# In vivo genome-wide CRISPR screen reveals breast cancer vulnerabilities and synergistic mTOR/Hippo targeted combination therapy

**DOI:** 10.1038/s41467-021-23316-4

**Published:** 2021-05-24

**Authors:** Meiou Dai, Gang Yan, Ni Wang, Girija Daliah, Ashlin M. Edick, Sophie Poulet, Julien Boudreault, Suhad Ali, Sergio A. Burgos, Jean-Jacques Lebrun

**Affiliations:** 1 Department of Medicine, McGill University Health Center, Cancer Research Program, Montreal, QC Canada; 2 Department of Animal Science, McGill University, Sainte-Anne-de-Bellevue, QC Canada; 3 Department of Medicine, McGill University Health Center, Metabolic Disorders and Complications Program, Montreal, QC Canada

**Keywords:** Breast cancer, Cancer genomics

## Abstract

Triple negative breast cancer (TNBC) patients exhibit poor survival outcomes and lack effective targeted therapies. Using unbiased in vivo genome-wide CRISPR screening, we interrogated cancer vulnerabilities in TNBC and identified an interplay between oncogenic and tumor suppressor pathways. This study reveals tumor regulatory functions for essential components of the mTOR and Hippo pathways in TNBC. Using in vitro drug matrix synergy models and in vivo patient-derived xenografts, we further establish the therapeutic relevance of our findings and show that pharmacological inhibition of mTORC1/2 and oncoprotein YAP efficiently reduces tumorigenesis in TNBC. At the molecular level, we find that while verteporfin-induced YAP inhibition leads to apoptosis, torin1-mediated mTORC1/2 inhibition promotes macropinocytosis. Torin1-induced macropinocytosis further facilitates verteporfin uptake, thereby greatly enhancing its pro-apoptotic effects in cancer cells. Overall, our study underscores the power and robustness of in vivo CRISPR genome-wide screens in identifying clinically relevant and innovative therapeutic modalities in cancer.

## Abstract (web summary)

Triple negative breast cancer (TNBC) lack effective therapies. Here, through an in vivo genome-wide CRISPR screen in TNBCs, the authors identify tumorigenic functions for components of the mTORC1/2 complex and of the YAP/Hippo pathway, and demonstrate that pharmacological inhibition of mTOR and YAP reduces tumour growth in vivo.

## Introduction

Breast cancer affects women worldwide with morbidity and mortality rates that continue to rise^1^. In particular, triple-negative breast cancer (TNBC), defined by the lack of expression of estrogen receptor, progesterone receptor, and human epidermal growth factor receptor-2, has a frequent onset in younger patients and accounts for about 15% of all breast cancer cases^2,3^. TNBC is associated with aggressive pathologic features such as high histology grade and mitotic index, higher rate of metastasis and relapse, lack of targeted therapy, and poor patient outcomes^4–8^. TNBC is a heterogeneous disease, comprised of several subgroups including basal-like, mesenchymal, mesenchymal stem-like, immunomodulatory, and luminal androgen receptor^9^. Despite this classification, our limited understanding of TNBC pathogenesis due to disease heterogeneity has made the development of effective therapeutic strategies a daunting challenge^10,11^. The complex nature of TNBC is further exemplified at the genomic level. While large-scale genomic landscape studies have revealed frequent genomic alteration occurrences within top cancer-driver genes (e.g., *TP53*, *MYC*, *PTEN*, *PI3K/AKT,* and *RB1*)^12,13^, most somatic mutations arise in tumor suppressor genes (74% *TP53*, 5.6% *PTEN*, and 5.6% *RB1*), further complicating efforts to efficiently target TNBC from a therapeutic perspective. Thus, a more comprehensive genome-wide approach to identify cancer vulnerabilities/dependencies in TNBC may provide a better rationalized and personalized method to discover treatment modalities for this disease.

Genome-wide genetic screens using genome editing systems such as clustered regularly interspaced short palindromic repeats (CRISPR)/Cas9 have emerged as advanced tools to systematically characterize cancer vulnerabilities^14–16^. Several large-scale CRISPR loss-of-function studies performed in vitro using cell passaging in 2D culture have allowed for identification of common essential genes and other cancer type-dependent survival genes^14–16^. By contrast, in vivo CRISPR screening at the genome-wide level, using preclinical models that better recapitulate and more closely resemble the patient 3D tumor micro-environment, has remained challenging. While a few recent studies have revealed the power of in vivo genome-wide CRISPR screens in non-small cell lung cancer and leukemia^17,18^, their proven utility in solving unmet medical needs remains unknown.

We applied an unbiased pooled CRISPR knockout (KO) screen in vivo using a TNBC xenograft model to interrogate key cancer vulnerabilities at a genome-wide level. Identification of high-confidence gene datasets from both positive and negative selections uncovers critical cancer vulnerabilities and tumor suppressor gene hypersensitivity in TNBC. We identify the oncogenic mTOR and tumor-suppressive Hippo signaling pathways as central regulators of tumorigenesis in TNBC. We further define novel roles for mTORC2/RICTOR and Sestrin3/GATOR2/WDR59 in promoting TNBC tumor growth. We also show that activation of the Hippo pathway blocks tumor growth through inhibition of the oncoprotein YAP and uncover tumor regulatory functions for Hippo pathway members (*SAV1* and *FRMD6*). We next establish the therapeutic relevance of our findings and show that pharmacological inhibition of mTORC1/2 (torin1) and of the YAP oncoprotein (verteporfin) efficiently reduces tumor growth in a preclinical orthotopic breast cancer transplantation model. Furthermore, using a drug matrix pharmacological approach and in vivo patient-derived xenograft, we find the combination drug treatment to exert synergistic effects in TNBC cell and animal models. At the mechanistic level, we find verteporfin to induce apoptosis and torin1 to promote macropinocytosis, an endocytic process that leads to engulfment of the extracellular fluid and catastrophic cell death^19^. Furthermore, torin1-induced macropinocytosis facilitates the verteporfin uptake and enhances its pro-apoptotic effects in cancer cells. Finally, using in vivo cell-derived and patient-derived xenograft models, we show that the proposed targeted combination therapy efficiently blocks tumor growth and development in TNBC preclinical settings.

## Results

### In vivo genome-wide CRISPR knockout screen in TNBC

To start deciphering cancer vulnerabilities and tumor suppressor gene hypersensitivity in TNBC, we performed a pooled genome-wide CRISPR loss-of-function screen using a lentiviral knockout library (GeCKOv2). The library contains 65,383 single guide RNAs (sgRNAs) including three for each of 19,050 targets and 1,000 non-targeting control sgRNAs^20^. The genome-wide screen was performed in the highly tumorigenic TNBC cell line SUM159PT (hereafter referred to as SUM159). SUM159 cells carry mutations in both oncogene (*PIK3CA* and *HRAS*) and tumor suppressor (*TP53*)^21,22^. While *HRAS* is not commonly mutated in TNBC, *PIK3CA*and *TP53* are the two most frequently mutated genes in breast cancer, particularly in TNBC patients^23^. Indeed, as shown in Supplementary Fig. 1a, analysis of the METABRIC dataset revealed high *TP53* and *PIK3CA*mutation frequency rates in TNBC patients (74.4% and 16.6%, respectively). Most patients with tumors containing the PIK3CA mutation also carried the TP53 mutations, accounting for 12% of all TNBC patients in the dataset. The PIK3CA mutation found in SUM159 cells is located on residue H1047 and leads to constitutive activation of the PI3 kinase^24,25^. H1047 mutation is also the most common PI3K mutation in over 2,500 breast cancer patients, as shown in the METABRIC dataset (Supplementary Fig. 1b)^26,27^. PIK3CA mutated patients showed poor survival outcome compared with PIK3CA non-mutated patients, while TP53 mutation was not predictive of TNBC patient survival (Supplementary Fig. 1c and d, respectively). Strikingly, TNBC patients harboring mutations in both genes had the worst overall survival outcomes with the lowest median survival (Supplementary Fig. 1e and f). As such, the SUM159 cancer model represents the most aggressive genetic features of TNBCs.

**Fig. 1:**
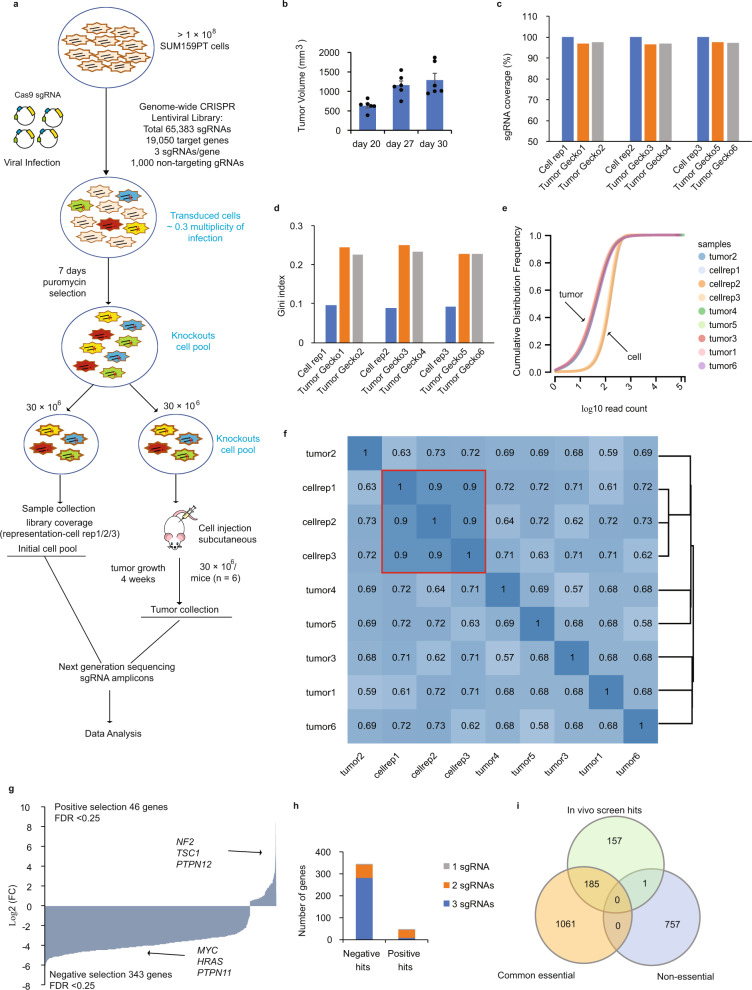
In vivo genome-wide CRISPR knockout screen in TNBC. **a** Schematic representation of the loss-of-function genome-wide screen using the human lentiviral CRISPR/Cas9 library (GeCKOv2) in triple negative breast cancer (TNBC) SUM159PT (SUM159) cells. **b** Tumor volume of NSG mice subcutaneously transplanted with 30 million of GeCKOv2 lentiviral library infected SUM159 cells with three independent infection replicate experiments (*n* = 6, 2 mice per biological replicate). Data are represented as mean±SEM. **c** The mapped percentage of sgRNAs in the library in cells before transplantation (*n* = 3), and tumor samples (*n* = 6) at day 30. **d** Gini index measures the evenness of sgRNA read depth within samples from cells and tumor replicates. **e** Cumulative distribution function of library sgRNAs in the three transduced cell replicates and six tumor replicates. Shift in tumor samples indicate the altered read counts in a subset of sgRNAs. **f** Pearson correlation of the sgRNA reads between all samples from in vitro and in vivo. The red box highlights the three biological replicates from the in vitro cell representation samples, showing high correlation. The black lines on the right side of the graph represent dendrograms of sample hierarchical clustering based on the distance between samples (calculated on the Pearson Correlation Coefficient). **g** Log2 (fold change) of top ranked genes in both positive and negative profiles (false discovery rate (FDR) < 0.25) in tumor samples normalized by transduced cell replicates. **h** Number of genes with 0, 1, 2, or 3 significantly enriched or depleted sgRNAs (FDR < 0.25) targeting that gene in both positive and negative profiles. **i** Venn diagram of negative selected genes overlapping with either common essential or non-essential gene lists. Source data are provided as a Source Data file.

As illustrated in Fig. 1a, three independent biological infections of the GeCKOv2 KO lentiviral library were performed in SUM159 cells, with a minimum coverage of 400 times per sgRNA and an average multiplicity of infection of 0.3 (estimated to result in one integrant per cell). Following puromycin selection, 30 million cells were subcutaneously transplanted into immunodeficient NOD scid gamma (NSG) mice. A separate 30 million cells were snap frozen to serve as an initial representation of the pooled library sgRNA in subsequent amplicon sequencing (referred to as Cell Rep1/2/3). Thirty days post transplantation, tumor development reached the humane endpoint (>1 cm^3^) at which time they were collected as representative of late-stage tumors (Fig. 1b). Tumor samples were processed through next-generation sequencing and the pooled sgRNA abundance and distribution, following in vivo selective pressure, were quantified using the MAGeCK Robust Rank Aggregation algorithm^28^. sgRNA read counts for each gene were compared before and after in vivo selective pressure (Cell Rep1/2/3 vs late-stage tumor samples). As shown in Fig. 1c, all in vitro (Cell Rep1/2/3) and in vivo tumor (Gecko1-6) samples contained an average sgRNA library representation of 99 and 97%, respectively, indicating sufficient library coverage in both conditions. Of note, all in vitro cell samples displayed an even and unbiased sgRNA distribution, as indicated by the low Gini index of 0.1, while in vivo tumor samples displayed a higher Gini index as a result of selective pressure (Fig. 1d). This is further illustrated by the shift in the log10^(readcounts)^ cumulative sgRNAs distribution curves observed between all Cell Rep and tumor samples (Fig. 1e). These results indicate that the in vivo selective pressure led to functional selection and altered distribution of the sgRNAs within the tumors, validating the functionality of our in vivo genome-wide CRISPR/Cas9 screen. Additionally, the biological replicates showed an average correlation of 0.98 within pre-transplantation in vitro control samples and of 0.66 within tumor replicates (Fig. 1f), further reflecting the high reproducibility of our screen. Importantly, the in vivo screen generated both positive and negative gene profiles (FDR < 0.25) with respective enrichment or depletion of at least two sgRNAs per gene, while none of the 1000 non-targeting sgRNAs ranked in either positive or negative selections (Fig. 1g and h, Supplementary Table 1).

Positive selection uncovered 46 enriched genes, likely to exert tumor-suppressive functions as their respective knockout promotes tumor growth in vivo. The gene list included several well-established tumor suppressors (*NF2*^29^, *TSC1*^30^, and *PTPN12*^31^), validating the specificity of the screen to reveal only those genes altered by and relevant to the in vivo selection pressure. Negative selection revealed 343 depleted genes that bear essential survival or tumor-promoting functions. As a proof of concept, the list included several well-known oncogenes such as *MYC*^32^, *HRAS*^33^, and *PTPN11*^34^ (Fig. 1g). To further assess the overall quality of the in vivo negative selection, we used a gold-standard approach whereby the 343-negative gene list was overlapped with both common essential and non-essential reference gene sets. Essentiality gene sets were derived from large-scale in vitro screening, available in the Achilles dataset 20Q1 from the DepMap portal^16,35^. The common essential gene set was created by selecting top genes responsible for cancer cell proliferation in over 90% of cancer cell lines screened while the non-essential gene list comprised genes whose individual deletion causes no substantial growth defect upon testing in multiple screens^35^. Around 50% of the in vivo negative screen hits were attributed an “essential” phenotype (185 out of total 343 hits) with only one hit classified as “non-essential” (Fig. 1i). Importantly, when analyzing mRNA expression of the 343 hits in a TCGA dataset of over 1200 breast cancer patients using the UCSC Xena and cBioPortal platforms^26,27^, we found a large number of these genes (117) to be specifically overexpressed in TNBC compared to non-TNBC tumors or normal tissues (Supplementary Fig. 2a). Further analysis of a large cohort of 3,593 breast cancer patients from METABRIC and TCGA pancancer datasets revealed these genes to be frequently amplified and exhibit increased gene alteration frequencies in basal-like (mostly comprised of TNBC patients) breast cancer compared with other subtypes (Supplementary Fig. 2b and c). These gene alterations also correlated with frequent TP53 mutation, higher tumor grade, and predicted poorer overall survival outcomes (Supplementary Fig. 2d and f, respectively). These results highlight the stringency, quality, and efficiency of our screen and define the shortlisted negative hits as potential tumor-promoting genes for TNBCs. Altogether, our in vivo genome-wide CRISPR screen displayed adequate library coverage, provided high-confidence level gene hit datasets from both positive and negative selections and further defined potential cancer vulnerabilities and tumor suppressor gene hypersensitivity in TNBC.

### Activation of the mTOR pathway promotes tumorigenesis in TNBC

**Fig. 2:**
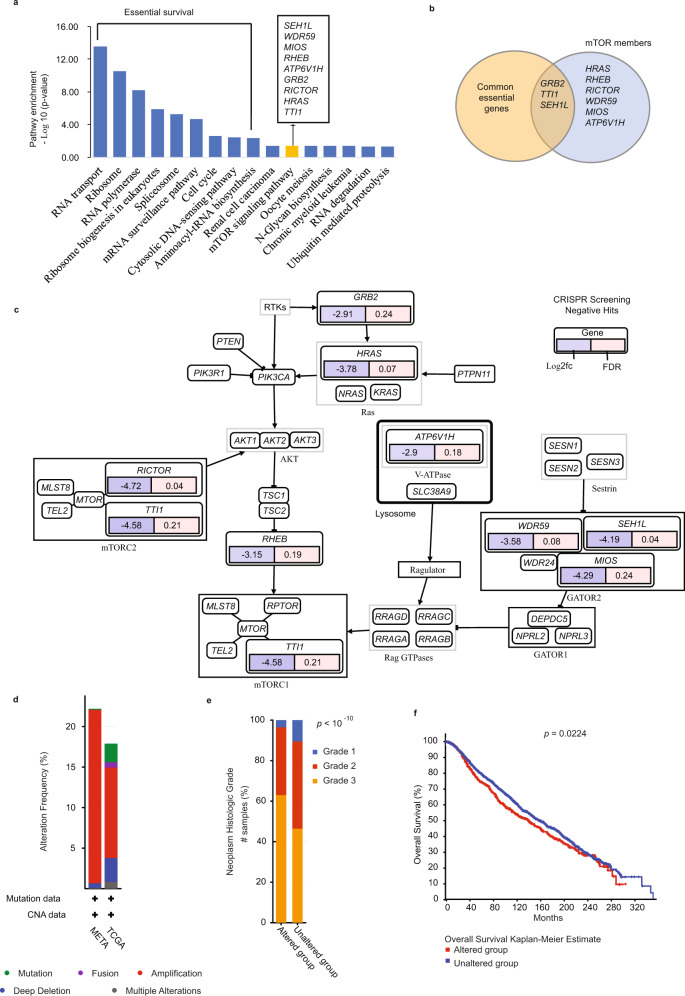
Activation of the mTOR pathway promotes tumorigenesis in TNBC. **a** Top-ranked pathways (*p* < 0.05) from KEGG pathway enrichment analysis of the negative selection gene list (343 genes) in tumors. In addition to essential survival pathways, the mTOR signaling pathway significantly ranked  with nine identified targets (labeled in the box). **b** Venn diagram of nine mTOR members overlapping with either common essential or non-essential gene lists. **c** Mapping of the nine mTOR signaling pathway members from in vivo screen . The identified target genes are labeled below with Log2 (fc) (blue color) and FDR (pink color) from in vivo screen analysis. **d** Percentage of genomic alteration frequency of nine combined mTOR hits in 3,593 breast cancer patients using METABRIC and TCGA pancancer datasets. The colors in the bar graph indicate specific mutation and copy number alterations (CNA). **e** Percentage of altered and unaltered groups of the nine mTOR hits in all grades (grade 1, 2, and 3) of breast cancer. *p* value was derived from Chi-squared test. **f** Kaplan Meier survival analysis of altered and unaltered groups of the nine mTOR hits in overall survival of breast cancer patients. *p* value was derived from Logrank test. Source data are provided as a Source Data file.

To identify oncogenic signaling pathways driving tumor growth in TNBC, we performed pathway enrichment analysis on the negative selection gene set using EnrichR^36,37^. As shown in Fig. 2a, the negative hits mostly exhibited essential survival functions such as RNA processing and cell cycle. Besides gene essentiality, nine members of the PI3K/AKT/mTOR (PAM) signaling pathway (*RICTOR*, *SEH1L*, *HRAS*, *WDR59*, *ATP6V1H*, *RHEB*, *TTI1*, *MIOS*, and *GRB2*) were identified, suggesting a prominent role for this pathway in promoting TNBC tumor growth. With the exception of three genes, *GRB2*, *TTI1*, and *SEH1L*, all the other genes were not found in the common essential list, suggesting they may have cancer type-specific functions (Fig. 2b). However, except for the well-characterized upstream mTOR activators (PI3K^25^, Grb2^38^, and HRAS^39^), a direct function for most mTOR components, including the ones found in our hit list, in the context of TNBC tumor development remains unknown. Individual PAM-related hits from our gene list were mapped and nodes were overlaid with fold changes (Log2fc) and FDR values (Fig. 2c) using PathwayMapper^40^. Hits included an upstream activator of PI3K (GRB2), two mTORC1/2 core components (RICTOR and TTI1), a direct activator of mTORC1 (RHEB), and HRAS, which can stimulate mTORC1 by inhibiting the TSC2-TSC1 complex^41^. In addition to responding to mitogens and growth factors, mTORC1 senses specific amino acid levels through the GATOR complexes^42^. In the presence of amino acids, GATOR2 inhibits GATOR1, a GAP protein that reduces mTORC1 activation by acting on Rag GTPases. Interestingly, three members of the GATOR2 complex (WDR59, MIOS, SEH1L) were found in our negative hits, underscoring the importance of these characterized GATOR2 components in promoting tumor growth in TNBC.

To further investigate the clinical relevance of the identified PAM hits, we examined their genetic alteration and expression levels in association with 3,593 breast cancer patients using METABRIC and TCGA pancancer datasets^26,27^. We found that most gene alterations were due to copy number amplifications and, to a much lower extent, to mutations/deletions (Fig. 2d). We then grouped these patients based on the ER/PR/HER2 status, and as shown in Supplementary Fig. 3a, the copy number amplifications of the nine PAM members were found in both TNBC and non-TNBC subtypes. In addition, gene alteration was increased in basal-like subtype (representing most of TNBC), but also occurred in HER2, luminal B subtypes (Supplementary Fig. 3b). We also compared gene alterations in different tumor stages. As shown in Supplementary Fig. 3c and d, gene alteration more frequently occurred in lymph node positive and stage II tumors, defined as large-sized tumors that spread to surrounding tissue. Our results also indicate that these alterations are more likely to occur in high-grade breast tumor phenotype and to predict for poor overall survival outcome (Figs. 2e, f). Overall, these gene alterations and correlations with poor prognostic features are not limited to TNBC, but also apply to non-TNBC patients. We then further compared the mRNA expression between TNBC and non-TNBC. Interestingly, TNBC patients exhibited higher expression of two members of the GATOR2 complex, *WDR59* and *SEH1L*, as well as a direct mTOR activator *RHEB,* compared to non-TNBC patients (Supplementary Fig. 3e). Together, these results highlight multiple components of the PAM signaling pathway as potent tumor growth regulators. Importantly, they also define a role for a previously uncharacterized PAM signaling circuitry (i.e., mTORC2 and GATOR2) in TNBC.

### Characterization of mTORC2 and GATOR2 function on TNBC tumor growth

**Fig. 3:**
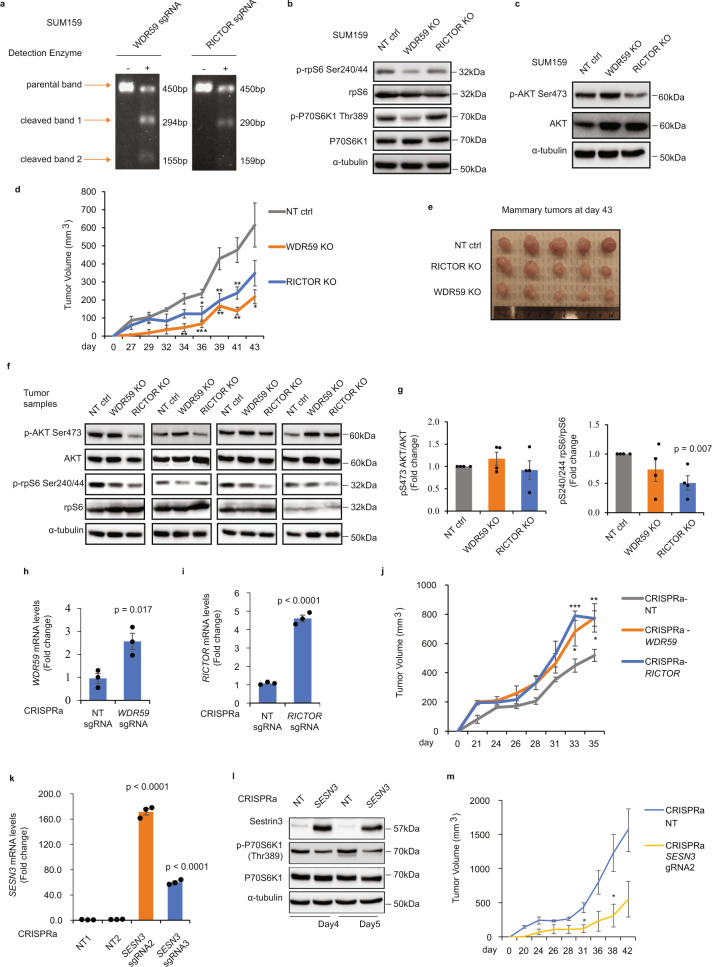
Characterization of mTORC2 and GATOR2 function on TNBC tumor growth. **a** Genomic modifications of lentiviral CRISPR/Cas9 sgRNA targeting WDR59 and RICTOR were examined using genomic cleavage assays in SUM159 (*n* = 2 independent experiments with similar results). **b**, **c** The effect of WDR59 and RICTOR knockout on mTOR signaling pathway in SUM159 cells was assessed at the protein level by immunoblotting using the  indicated antibodies. The non-targeting (NT) sgRNA was used as a control (*n* = 2 independent experiments with similar results). **d** Orthotopic mammary fat pad transplantation of NT, WDR59, and RICTOR KO SUM159 cells in NSG mice (*n* = 5 per group). Mammary tumor growth was assessed by measuring tumor volume every two days. Data are represented as mean±SEM. *p* values are comparing each KO group vs. NT control (NT ctrl) by two-sided unpaired *t* test at the same day. *P < 0.05, **P < 0.01 or ***P < 0.001. **e** Representative images of the mammary tumors (NT ctrl, WDR59 KO, and RICTOR KO) were collected at day 43 and shown. **f** Protein lysates derived from day 43 tumor samples (NT ctrl, WDR59 KO, and RICTOR KO) were assessed for mTOR signaling pathway by immunoblotting with the indicated antibodies (*n* = 4 tumor samples/group). **g** Quantification of the ratio of pS240/244rpS6/rpS6 and pS473AKT/AKT from different KO tumor samples by densitometry analysis from 16 animals (*n* = 4 tumor samples/group). Data are represented as mean±SEM and *p* values by two-sided unpaired *t* test are indicated. **h**, **i** Induction of mRNA expression of WDR59 and RICTOR using specific lentiSAM CRISPR sgRNAs was shown in SUM159. Data are represented as mean±SEM and *p* values by two-sided unpaired *t* test are indicated (*n* = 3 cells per group). **j** CRISPR activation NT ctrl, WDR59, and RICTOR transduced SUM159 were orthotopically transplanted in NSG mice (*n* = 6 mice per group). Mammary tumor volumes are represented as mean±SEM. *p* values are comparing each activation group vs. NT ctrl by two-sided unpaired *t* test at the same day. *P < 0.05, **P < 0.01, or ***P < 0.001. **k** Induction of mRNA expression of sestrin3 using two specific lentiSAM CRISPR sgRNAs in SUM159. Data are represented as mean±SEM and *p* values by two-sided unpaired *t* test are indicated (*n* = 3 cells *p*er group). **l** Protein levels were examined in CRISPR activation ctrl, and sestrin3 transduced SUM159 by immunoblotting with the indicated antibodies (*n* = 2 independent experiments with similar results). **m** Ctrl and sestrin3 activation transduced SUM159 were orthotopically transplanted in NSG mice (*n* = 3 mice per group). Primary mammary tumor growth was assessed by measuring tumor volume. Data are represented as mean±SEM and *p* values by two-sided unpaired *t* test are indicated. *P < 0.05. Source data are provided as a Source Data file.

As indicated above, the direct functions of mTORC2 or GATOR2 complex in primary mammary tumor development have not yet been characterized in TNBC. Thus, to assess their functional relevance and contribution to TNBC tumor formation, we used a CRISPR/Cas9 KO approach to specifically block expression of one identified representative hit for each of the mTORC2 and GATOR2 complexes (RICTOR and WDR59, respectively) in the SUM159 cell line. The presence of proper indel mutations in the bulk KO cells was verified using genomic cleavage assays (Fig. 3a). The ability of WDR59 KO to block GATOR2 signaling was verified through reduced phosphorylation levels of the two downstream mTORC1 targets, rpS6 and p70S6K1 (Fig. 3b). Functional validation of the RICTOR KO in regulating mTORC2 activity was confirmed by decreased phosphorylation of its substrate AKT on Ser473 (Fig. 3c).

To then assess the role and contribution of RICTOR and WDR59 to breast cancer formation, individual KOs were orthotopically transplanted into the mammary fat pad of NSG mice and tumor volume was measured every two days. Strikingly, individual RICTOR and WDR59 genomic deletion significantly blocked primary mammary tumor growth and reduced tumor size in NSG mice, highlighting these two proteins as potent pro-oncogenic factors (Fig. 3d and e). Moreover, as shown in Fig. 3f, immunoblot analysis of resected tumor tissues revealed that in the absence of RICTOR, we observe a consistent decrease in the ratio (p-AKT Ser473 relative to total AKT). However, this decrease was not statistically significant (Fig. 3g), possibly due to the heterogeneity between animals and numbers of resected tumor samples and/or to the fact that AKT phosphorylation is a very transient event that is likely not sustained by the time the tumors are resected. On the contrary and as expected, the WDR59 KO had no effect on p-AKT Ser473, as this phosphorylation event is specifically regulated by the mTORC2 complex. Interestingly, WDR59 and RICTOR KOs showed strong decrease in rpS6 phosphorylation levels, compared with non-targeting tumor samples (Fig. 3f, g).

To further address the tumor-promoting function of RICTOR and WDR59 in the mammary gland, we used a CRISPR activation (CRISPRa) approach (see methods for details)^43,44^. Using CRISPRa sgRNAs specifically targeting RICTOR and WDR59 gene promoters, we could significantly increase endogenous RICTOR and WDR59 gene expression in SUM159 cells (Fig. 3h, i). Importantly, activation of both RICTOR and WDR59 gene expression led to a significant increase in mammary tumor growth (Fig. 3j). Together, these results clearly define a direct tumor-promoting function for RICTOR in the mammary gland. Moreover, we identified a function for WDR59 as a potent inducer of mammary tumor growth, defining the nutrient-sensing GATOR2 complex as a pro-oncogenic pathway in TNBC.

The GATOR2 complex is negatively regulated by a family of stress-related proteins called Sestrins. In particular, Sestrin3 is known to complex with and inhibit GATOR2 activity independently of intracellular leucine levels^45^. To gain further insights into GATOR2 role in tumor development and examine whether Sestrin3 could act as an upstream regulator of GATOR2 to regulate mammary tumor growth in TNBC, we used the CRISPRa system to specifically induce endogenous Sestrin3 expression. Using two specific sgRNAs targeting different domains of the Sestrin3 promoter, we could significantly induce endogenous Sestrin3 gene expression (*SESN3*) by 64- and 170-fold in SUM159 TNBC cells (Fig. 3k). This was followed by an increase in Sestrin3 protein levels and further inhibition of mTOR signaling, as illustrated by the reduced p70S6K phosphorylation levels (Fig. 3l). The effect of increased Sestrin3 expression on tumor growth was then assessed in vivo, through orthotopic transplantation of the SUM159-SESN3 cells in NSG mice. As shown in Fig. 3m, increasing Sestrin3 expression levels led to a significant reduction of the mammary tumor burden over time. These results clearly define a function for the GATOR2 complex as a tumor promoter in TNBC and highlight the negative regulator Sestrin3 as a potential tumor suppressor. Altogether, these results underscore the critical role played by the oncogenic mTOR pathway in TNBC tumor development. Our study also revealed multiple PAM signaling components as important regulators of the tumorigenic process, further defining roles for mTORC2/RICTOR and GATOR2/WDR59 in TNBC mammary tumor growth and suggesting that Sestrin3-mediated negative regulation of GATOR2 may prevent these effects.

### Blocking the Hippo pathway promotes tumorigenesis in TNBC

**Fig. 4:**
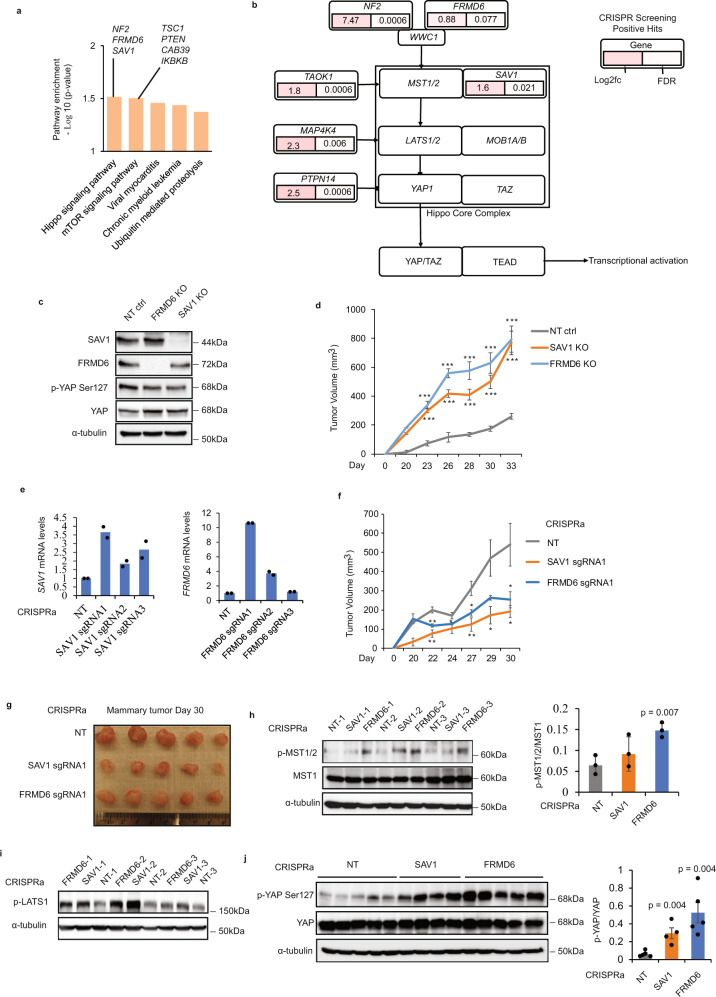
Blocking the Hippo pathway promotes tumorigenesis in TNBC. **a** Top-ranked pathways (*p* < 0.05) from KEGG pathway enrichment analysis of the positive selection list (46 genes) in tumors. **b** Individual Hippo-related hits from our gene list were mapped using PathwayMapper and nodes were overlaid with fold changes (Log2fc) and FDR values. **c** The effects of SAV1 and FRMD6 knockouts on SAV1 and FRMD6 expression as well as YAP phosphorylation and total protein levels in SUM159 cells were assessed by immunoblotting with the indicated antibodies (*n* = 2 independent experiments with similar results). **d** Orthotopic mammary fat pad transplantation of NT, SAV1, and FRMD6 KO SUM159 cells in NSG mice (*n* = 6 mice per group). Mammary tumor growth was assessed by measuring tumor volume every two days. Data are represented as mean±SEM. *p* values are two-sided unpaired *t* test, ***P < 0.001. **e** Activation of the endogenous SAV1 and FRMD6 gene promoters using three different sgRNAs lentiviral CRISPR/dcas9 SAM constructs for each gene in SUM159 cells. Induction of SAV1 and FRMD6 mRNA expression were examined by RT-PCR in transduced SUM159 cells (*n* = 2 cells from three sgRNAs/gene). **f** Transduced SUM159 cells with CRISPR activation ctrl, SAV1, and FRMD6 constructs were orthotopically transplanted in NSG mice (*n* = 5 mice per group). Primary mammary tumor growth was assessed by measuring tumor volume. Data are represented as mean±SEM. *p* values are two-sided unpaired *t* test, *P < 0.05, **P < 0.01 or ***P < 0.001. **g** Representative images of the mammary tumors were collected at day 30. **h** Protein lysates derived from day 30 tumor samples (LentiCRISPRa NT, SAV1, and FRMD6) were assessed for Hippo/YAP signaling pathway by immunoblotting with  the indicated antibodies. Quantification of the ratio of p-MST1/2/MST1 was performed to compare the control samples with SAV1 and FRMD6 activation samples (*n* = 3 tumor samples/group). Data are represented as mean±SEM and *p* values by two-sided unpaired *t* test are indicated. **i** Immunoblotting of p-LATS1 and a-tubulin in NT, SAV1, and FRMD6 activation tumor samples (*n* = 3 tumor samples). **j** Immunoblot and quantification of the ratio of p-YAP/YAP was performed to compare the control samples (*n* = 5) with SAV1 (*n* = 4) and FRMD6 (*n* = 5) activation samples. Data are represented as mean±SEM and *p* values by two-sided unpaired *t* test are indicated. Source data are provided as a Source Data file.

Tumorigenesis is a complex process that can result from the activation of oncogenic pathways but also from the loss of tumor suppressor activity. Identification of the positively-selected genes from genome-wide CRISPR screens provide a unique opportunity to uncover such functional suppressor pathways. To identify predominant pathways from our screen positive selection gene set (46 hits), we performed gene pathway enrichment analysis using EnrichR. As shown in Fig. 4a, the tumor suppressor Hippo signaling pathway emerged as the top candidate. Additionally, we found negative regulators of the mTOR signaling pathway to be also highly ranked. Individual Hippo pathway hits (*FRMD6*, *NF2*, *SAV1*, *TAOK1*, *MAP4K4*, *PTPN14*) and their downstream regulators were integrated with their associated FDR and fold change values using PathwayMapper (Fig. 4b). The Hippo pathway is comprised of four core components, MST1/2 and LATS1/2, and is activated by high cell density, extracellular matrix stiffness, and lack of nutrients^46^. SAV1 (coding for Salvador) is a MST1/2 coregulatory protein. NF2 (coding for Merlin) and FRMD6 (coding FERM domain-containing protein 6) are upstream regulators of the core cascades which activate the Hippo pathway by phosphorylating MST1/2 and LATS1/2. The tumor-suppressive function of the Hippo pathway is mediated through inhibition of the YAP/TAZ regulatory complex through phosphorylation of YAP (Ser 127), nuclear export, and subsequent ubiquitin degradation of YAP protein, further preventing TEAD-mediated gene transcription of its multiple oncogenic targets^46^. To characterize the potential tumor-suppressive function of the Hippo-related hits, we used both CRISPR/Cas9 KO and CRISPRa systems described above in SUM159 cells. Two hits (SAV1 and FRMD6) were selected as proof of principle, as neither was previously functionally characterized in the context of tumorigenesis. As shown in Fig. 4c, specific CRISPR-SAV1 and -FRMD6 KOs were generated. Both KOs led to drastically reduced protein levels of their respective targets, Salvador and FRMD6. The ratio of phosphorylation of YAP Ser127/total YAP is reduced in the SAV1 and FRMD6 KOs compared to control cells, suggesting that Salvador and FRMD6 could inhibit the YAP oncogene in TNBC. Importantly, when tested in preclinical models of TNBC orthotopic transplantation, the two individual SAV1 and FRMD6 CRISPR-KO were able to significantly facilitate primary mammary tumor growth (Fig. 4d). By contrast, activation of SAV1 and FRMD6 gene expression using the CRISPRa system led to significant inhibition of mammary tumor growth and strongly reduced tumor size (Fig. 4e–g). Importantly, immunoblot analysis of the resected tumors showed that activation of SAV1 and FRMD6 by CRISPRa system leads to activation of Hippo core cascades as measured by p-MST1/2 and p-LATS1 (Figs. 4h, i). Moreover, the activation of Hippo pathway by overexpression of Salvador and FRMD6 results in an increase of YAP phosphorylation at Ser127, an inhibitory site of YAP oncoprotein (Fig. 4j). Altogether, these results not only validate the positive hit selection from our screen but also uncover tumor-suppressive functions for both Salvador and FRMD6 in TNBC, through inhibition of the YAP oncogene.

### Pharmacological mTOR and YAP inhibitors synergistically block tumor growth

Having defined the mTOR and Hippo/YAP pathways as critical to TNBC tumor development, we next explored the therapeutic value of our findings. Pharmaceutical mTORC1 inhibitors (rapalogues) have failed to prevent tumor progression in clinical trials of various cancer types due to mTORC1-dependent negative feedback effect on mTORC2/AKT^47,48^. To overcome this limitation, second-generation inhibitors have been developed to simultaneously block both mTORC1 and mTORC2 and are currently undergoing clinical trials in various types of cancer patients^49^. To block the oncogenic mTORC1/2 pathways, we used the ATP-competitive selective mTOR inhibitor, torin1^50^. To activate the tumor-suppressive Hippo pathway, we used verteporfin, a YAP inhibitor that prevents YAP/TEAD binding and transcriptional activity^51^. Verteporfin is a FDA-approved drug used to treat macular degeneration and was recently proposed to exert anti-tumorigenic activities in retinoblastoma and lung cancer^52,53^. Synergy studies with the BET bromodomain inhibitor (BBDI) JQ1 and verteporfin also showed the combination could inhibit in vitro cell cultures of BBDI-resistant derivatives cell lines^54^.

**Fig. 5:**
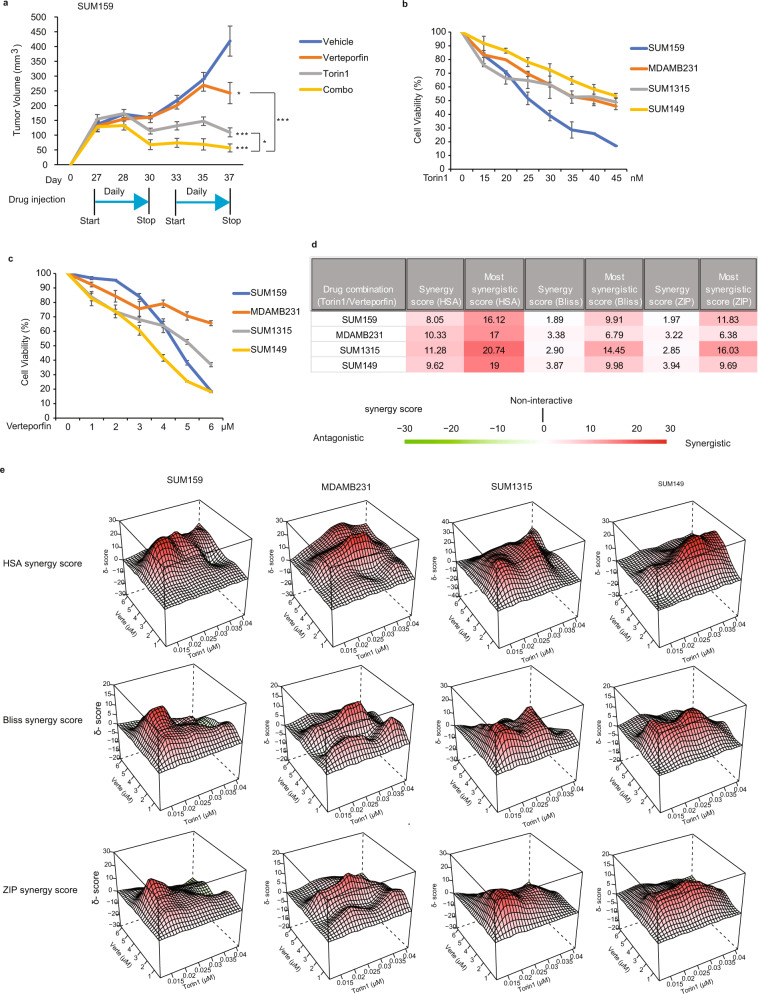
Pharmacological mTOR and YAP inhibitors synergistically block tumor growth. **a** SUM159 breast cancer cells were transplanted into the mammary fat pad of NSG mice and tumors allowed to develop for 27 days until reaching around 200 mm^3^ in tumor volume. Mice were split into four groups and subjected to vehicle (*n* = 6 mice), verteporfin (100 mg/kg) (*n* = 6 mice), torin1 (20 mg/kg) (*n* = 7 mice) and combination treatment (verteporfin/torin1, combo) (*n* = 7 mice) through daily intraperitoneal injections. Primary mammary tumor growth was assessed by measuring tumor volume. Data are represented as mean±SEM. *p* values are two-sided unpaired *t* test, *P < 0.05, or ***P < 0.001. **b**, **c** Percentage of cell viability in four TNBC cell lines (SUM159, MDA-MB231, SUM1315, SUM149) treated or not with torin1 or verteporfin for 3 days at the indicated doses, using Prestoblue staining. Data are represented as mean±SEM (*n* = 3 independent experiments for each cell line). **d**, **e** Four TNBC cell lines were treated with combinational drug matrix of verteporfin and torin1 at the indicated doses for 3 days. Cell viability was measured by PrestoBlue staining. Synergy scores and 3D surface plots of cell viability in four TNBC cell lines were quantified and analyzed with HSA, ZIP, and Bliss models using SynergyFinder (*n* = 3 independent experiments for each cell line). Source data are provided as a Source Data file.

The two specific inhibitors were first tested alone or in combination using the xenograft breast cancer model described above. Following orthotopic TNBC cell transplantation in NSG mice, tumors were allowed to grow until they reached 200 mm^3^. Mice bearing similar tumor volumes were then selected and divided into four separate groups (6 mice/group) for subsequent drug treatments: vehicle, verteporfin (100 mg/kg), torin1 (20 mg/kg), and combination treatment (verteporfin/torin1). Drugs were administered through daily intraperitoneal injections for 2 weeks (with a 5-h interval delivery between verteporfin and torin1 in the combination treatment group to prevent any potential interaction between the 2 formulations) and tumor volumes were measured through caliper measurements^55,56^. As shown in Fig. 5a, torin1 showed great efficacy in preventing primary tumor development. While verteporfin alone exhibited a weaker and later onset effect on tumor growth, compared to torin1, it did reach significant tumor growth inhibition at the end of the treatment course. Interestingly, the combination treatment torin1/verteporfin resulted in an accelerated and significantly improved effect on tumor volume reduction compared to either single drug administration. These results highlight the cooperative nature of these two pathways in promoting TNBC tumor development and the importance of the dual inhibition to attain greater tumor-suppressive effects and potential patient treatment benefits.

We next assessed and further characterized the proposed drug combination treatment efficacy using other TNBC models. For this, we used a pharmacological combinatory approach^57^ whereby we designed a drug matrix combining different doses of each inhibitor and quantified the resulting cell viability parameters. A panel of four TNBC cell lines (SUM159PT, MDA-MB231, SUM1315MO2, and SUM149PT) originated from different patients were used in the study^58^. Initial single drug dose-response treatments revealed that both drugs efficiently blocked cell viability in all TNBC cell lines, when used individually (Fig. 5b, c). We next measured and quantified drug efficacy and combination treatments using the drug matrix design (Supplementary Fig. 4a). For data analysis, we used several well-established pharmacological models (HSA, Bliss, and ZIP) with the SynergyFinder algorithm^57^. As shown in Fig. 5d and e, the observed combinatorial drug effects were stronger than individual effects in all TNBC cell lines tested. Interestingly, all three models (HSA, Bliss and ZIP) revealed positive synergistic scores between the two drugs in all TNBCs. Moreover, further detailed data analysis, using the two most stringent models (Bliss and ZIP) revealed that the highest synergy scores are observed in the areas that combine the lowest doses for each drug, highlighting additional potential clinical benefits (reduced toxicity) in using such a combination treatment (Fig. 5e and Supplementary Fig. 4b). Overall, our results show that pharmacological inhibition of mTORC1/2 (torin1) and YAP (verteporfin) efficiently block TNBC tumor growth in vitro and in vivo, revealing a synergism between the two drugs and highlighting the potential and benefits of such targeted combination therapy as a highly efficient indication for TNBC patients.

### Torin1 enhances verteporfin-induced apoptosis in TNBC

**Fig. 6:**
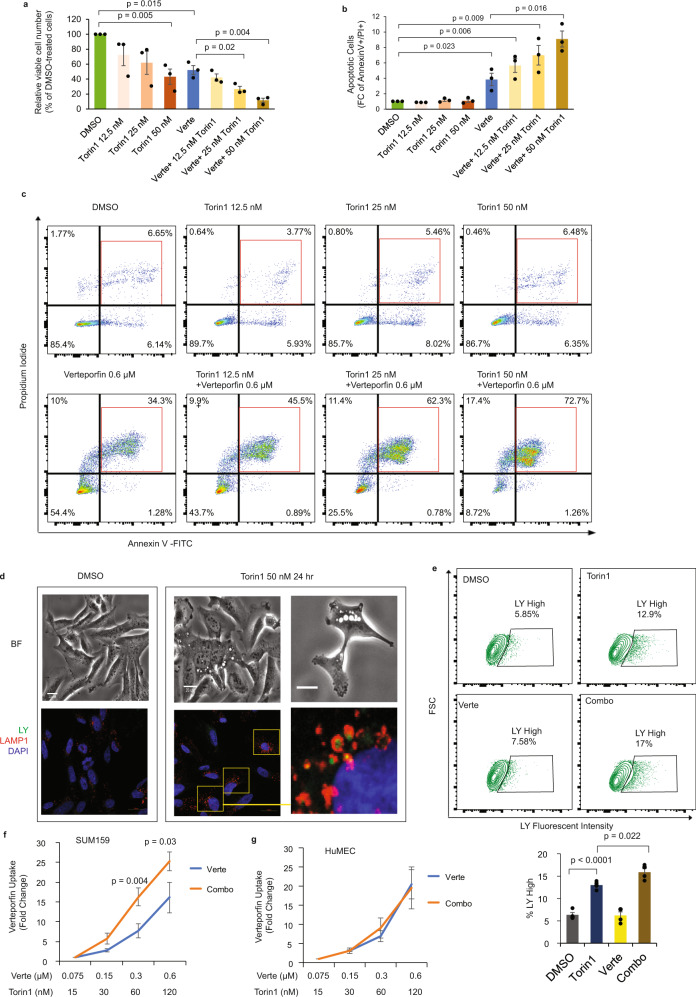
Torin1 enhances verteporfin-induced apoptosis in TNBC. **a** Drug response to torin1 and verteporin (verte) alone or in combination on SUM159 cell viability. Data are represented as mean±SEM and *p* values by two-sided unpaired *t* test are indicated (*n* = 3 independent experiments per treatment). **b**, **c** Percentage of apoptotic cells (Annexin V + /PI + ) in SUM159 treated with the indicated doses of individual drug or combination. Data are represented as mean±SEM and *p* values by two-sided unpaired *t* test are indicated (*n* = 3 independent experiments per treatment). **d** Bright field and fluorescence of LY (green) and LAMP1 (red) in SUM159 cells treated with 50 nM torin1 for 24 h (*n* = 3 independent experiments with similar results). Scale bar is 20 µm. **e** Flow cytometry analysis of LY in SUM159 cells treated with torin1 (50 nM) and verteporfin (0.6 µM) for 24 h. Quantification of LY high percentage cells (*n* = 4 biological replicates per treatment). *p* values by two-sided unpaired *t* test are indicated. Intracellular verteporfin fluorescence in SUM159 cells (**f**) and HuMEC (**g**) treated with verteporfin alone or in combination with torin1. Data are represented as mean±SEM and *p* values by two-sided paired *t* test are indicated (*n* = 3 biological replicates per treatment). Source data are provided as a Source Data file.

We next investigated the intracellular and molecular mechanisms by which torin1 and verteporfin relay their tumor-suppressive effects in TNBC. For this, we first assessed the drug effects on cancer cell death and survival. As shown in Fig. 6a, both torin1 and verteporfin significantly decreased cancer cell numbers. Interestingly, combination treatment using both drugs resulted in almost complete inhibition of cell viability, confirming the synergism between the two drugs and highlighting the potency of the combi-therapy modality. To gain further mechanistic insights, we next examined the drug effects on cell death. To address this, we assessed apoptosis by measuring Annexin V + /PI + population upon single and combined drug treatments. As shown in Fig. 6b, 6c, and Supplementary Fig. 5, verteporfin had a drastic pro-apoptotic effect after 3 days of treatment, while torin1 has no effect on cell apoptosis at various doses. Interestingly, the apoptotic effect of verteporfin was further enhanced significantly when combined with increased doses of torin1.

Examination of torin1-treated TNBC cells under bright field (BF) microscopy revealed the apparition of an endocytic process, characteristic of macropinocytosis (Fig. 6d). Macropinocytosis is characterized by engulfment of large amount of the extracellular fluid, which leads to the formation of membrane-bound large intracellular vacuoles, referred to as macropinosomes^59^. Macropinocytosis mostly occurs in cancer cells, contributing to their nutrient supplies under starving conditions^19,60^. However, the continuous accumulation of these large vacuoles has also been reported to lead to cell membrane rupture and subsequent non-apoptotic catastrophic cell death^19^. A recent study showed that high dose of mTORC1/2 inhibitors at a range of 5 to 25 µM induced catastrophic macropinocytosis and cell death in various cancer cells^61^. Macropinocytosis can be assessed by rapid incorporation of fluid-phase tracers such as lucifer yellow (LY), apparition of the late lysosomal marker LAMP1, and maturation of macropinosome fused with lysosome^61^. As shown in Fig. 6d, treatment of torin1 at final 50 nM concentration induced large vacuole formation, followed by accumulation of intracellular LY which co-localized with the late lysosomal marker LAMP1, thus indicating that torin1 can induce macropinocytosis in TNBC. Quantitative analysis of the LY uptake, using flow cytometry showed that torin1 significantly increased the percentage cells with high LY fluorescent content, compared with DMSO and verteporfin treated cells (Fig. 6e and Supplementary Fig. 6). Altogether, our results indicate that verteporfin and torin1 can induce cell death through distinct mechanisms in TNBC.

Having shown that the two drugs synergize their effects to reduce tumor growth both in vitro and in vivo and that the combination treatment led to much increased cell death, we next sought to investigate the molecular mechanism underlying the synergism. Having shown that torin1 could induce the extracellular fluid uptake, we hypothesized that torin1 could potentially facilitate the verteporfin entry to the cells, along with the extracellular fluid, when both drugs are administered together. Increased intracellular verteporfin concentrations would then result in increased apoptotic cell death and account for the synergism between the two drugs. To address this, we next assessed the intracellular level of verteporfin in the presence or the absence of torin1. Conveniently, intracellular verteporfin levels can be quantified through measuring verteporfin innate auto-fluorescence in the far-rad spectrum^62^. As shown in Fig. 6f, and as expected, the intracellular verteporfin fluorescent signal increased in a dose-dependent manner. Interestingly, combination treatments with torin1 resulted in a clear and significant increase in verteporfin intracellular levels, compared with verteporfin alone at the same doses (Fig. 6f). These results strongly suggest that torin1 enhances verteporfin uptake into the cells though macropinocytosis, further leading to enhanced verteporfin-mediated apoptotic cell death.

Moreover, additional analysis of the torin1/verteporfin combination treatments in normal primary human mammary epithelial cells (HuMEC) showed that the torin1 effect is specific to cancer cells. Indeed, HuMEC treatment with torin1 did not induce macropinocytosis, LY uptake, and LY/LAMP1 co-localization (Supplementary Fig. 7a, b), nor it increased the verteporfin intracellular uptake when the two drugs were administered simultaneously (Fig. 6g). This suggests that the combination treatment could appear be more selective and efficient in cancer cells, compared to normal cells, further highlighting its therapeutic value as a drug combination indication.

### Torin1/Verteporfin co-treatment blocks tumor growth in patient-derived xenografts

**Fig. 7:**
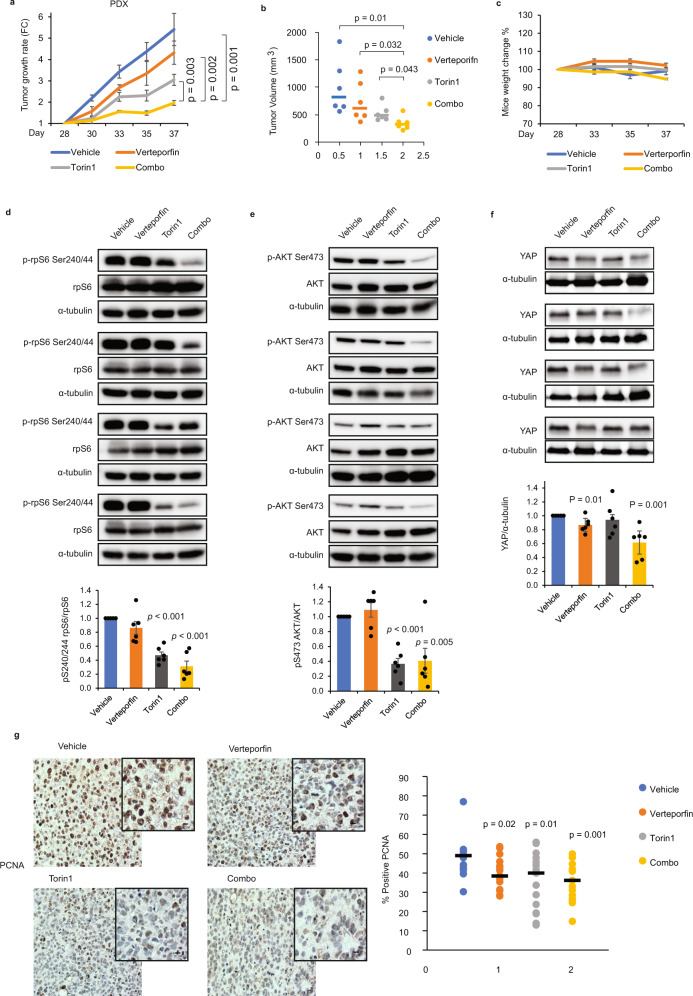
Torin1/Verteporfin co-treatment blocks tumor growth in patient-derived xenografts. **a** TNBC PDX tumors were subcutaneously transplanted into NSG mice and allowed to develop for 28 days. Mice were split into four groups (6 mice per group) with similar average tumor volume and subjected to vehicle, verteporfin (100 mg/kg), torin1 (8 mg/kg), and combination treatment (verteporfin/torin1, combo) by intraperitoneal injection for 2 weeks. Tumor growth rates from the initial measurement of individual mice are presented. Data are represented as mean±SEM and *p* values by two-sided unpaired *t* test are indicated (*n* = 6 mice per group). **b** Tumor volume of individual mice at day 37 was graphed for the treated PDX mice. Data are represented as dot plots with the middle line as the median. *p* values by two-sided unpaired *t* test are indicated (*n* = 6 mice per group). **c** Mice body weight was measured during the treatment. Changes in mice body weight during treatments are represented as mean±SEM (*n* = 6 mice per group). There is no significant difference between groups. **d**–**f** Representative images show that protein lysates derived from day 37 PDX tumor samples treated with vehicle, verteporfin, torin1 and combination were assessed for immunoblotting with the indicated antibodies. Quantification of the ratio of phosphor/total indicated proteins by densitometry analysis. Data are represented as mean±SEM and *p* values by two-sided unpaired *t* test are indicated (*n* = 6 tumor samples per group). **g** Representative images and quantification of PCNA positive cells in PDX tumor tissues from vehicle and drug-treated groups. Data are represented as dot plots with the middle line as the median value. *p* values by two-sided unpaired *t* test are indicated (*n* = 5 mice per group, 3 regions per tissue section). Scale bar is 20 µm. Source data are provided as a Source Data file.

To further emphasize the clinical relevance of the proposed drug combination treatment to TNBC patients, we next examined the drug effects (alone or in combination) in a TNBC patient-derived xenograft (PDX) model. PDX are more representative of human cancer biology and predictive of patient treatment responses, and thus better suited to test any combination therapy. The TM00096 PDX model used in our study was established from a grade 3 metastatic invasive ductal carcinoma (52-year old White/Hispanic woman). PDX tumors were maintained at low passage from initial patient tumor implantation. For expansion, tumors were harvested, minced, and transplanted subcutaneously into recipient NSG mice. Upon PDX tumor size reaching 200 mm^3^, mice were separated into four groups (6 mice per group) based on similar median tumor size. Animals were then treated or not with the inhibitors alone or in combination through daily i.p. injections at the indicated doses. Excitingly, while both verteporfin and torin1 showed a significant reduction in tumor growth rate and in final tumor volume when administered alone, the combination treatment resulted in a nearly maximal, sustained tumor growth inhibition throughout the entire duration of the treatment (Fig. 7a, b). We also monitored potential drug toxicity and did not observe any changes in body weight, food intake, and hair loss during the treatment course across the different groups, further supporting the benefits of the combination therapy (Fig. 7c).

To further examine the drug action efficacies within the tumors in the PDX model, resected tumors were analyzed to assess for mTOR/YAP signaling pathway activities through immunoblot analysis. As shown in Fig. 7d, torin1 alone or in combination significantly decreased downstream mTOR activity, as measured by the ratio of phosphorylation of rpS6 relative to total rpS6 within the tumor samples. Likewise, torin1 also inhibited AKT activation and reduced the ratio of phosphorylation of AKT at Ser473, relative to total AKT, consistent with inhibition of the mTORC2 complex (Fig. 7e). When assessing the verteporfin effects in tumor samples, we found that verteporfin alone or in combination showed a significant decrease of YAP protein expression (Fig. 7f). Finally, immunohistology of the resected tumors showed that daily injections of torin1 and verteporfin alone or in combination significantly reduced cell proliferation within the tumors as assessed by the expression levels of the proliferation marker, PCNA (Fig. 7g).

Overall, our study underscores the relevance and power of in vivo genome-wide CRISPR screens to interrogate cancer vulnerabilities, tumor suppressor hypersensitivity, and discover anti-cancer therapies. Our results also uncover an efficient approach to circumvent tumorigenesis and reduce the tumor burden through simultaneous targeting of pro-oncogenic and tumor suppressor pathways. Finally, our findings will help fill a much-needed medical gap in the metastatic breast cancer market, with the development of a potential first-line targeted therapy for the treatment of TNBC and the prospect of rapidly transitioning to clinical trials in humans.

## Discussion

The complex and heterogeneous nature of TNBC at the molecular and genomic levels makes this disease challenging to target with effective therapies. Classification of TNBCs into distinct subgroups based on their genomic, transcriptomic, and proteomic characteristics, has helped define comprehensive datasets, further revealing potential drug targets (receptor tyrosine kinases, PI3K/AKT, Ras/MAPK, JAK/STAT, and cell cycle regulators). However, while these elegant “omics” technologies are efficient to link gene alterations to clinical features and subtypes, they have limited capacity in identifying actionable ‘driver alterations’ in cancer. In fact, to date, no clinical benefits were achieved in TNBC by individually targeting such altered genes. This is in part due to the intricate interplay between these pathways and to the fact that tumorigenesis is multifactorial resulting not from one but multiple gene alterations. Thus, large-scale approaches, such as the in vivo genome-wide CRISPR screen described here, hold great promise to functionally identify clustered causal genes in cancer and allow for the development of suitable combinatorial therapies.

Large-scale CRISPR loss-of-function genomic screens are the optimal unbiased tools for identifying genes and pathways underlying tumorigenic processes. In this system, the Cas9 nuclease is targeted to the genomic locus of a specific gene, using a single gRNA to generate double-stranded DNA breaks, resulting in highly efficient gene knockouts. Several large-scale studies using this technology have been performed in vitro to interrogate common essentiality genes and other cancer type-dependent survival genes in over 700 cancer cell types^14–16^. While elegant and accurate, these in vitro settings also showed limitations concerning their ability to reflect tumorigenic processes from a 3D in vivo environment. In contrast, in vivo genome-wide CRISPR screens are better suited and more clinically relevant but remain technically challenging. In particular, proper tumor cell engraftment capable of maintaining a sufficient sgRNA representation remains a major challenge^18^. To circumvent this issue, the use of high-seeding capacity cancer cell lines is a prerequisite for suitable in vivo functional CRISPR screening. In this study, we used a TNBC cell line (SUM159) highly enriched in cancer stem cells and with a high tumor initiation capacity^63^. Proof of concept, validation of the experimental design, and high confidence of the obtained datasets were demonstrated by the high sgRNA representation in the tumor samples, identification of multiple specific sgRNAs for any given target gene and the identification of several well-known tumor suppressors and oncogenes in the screen (i.e., NF2, TSC1, myc, and Hras). Interestingly, datasets generated from our negative and positive selections uncovered an oncogenic (mTOR) and a tumor suppressor (Hippo) signaling pathways as the main regulators of the tumorigenic process in TNBC. This approach can apply and extend to other types of cancer lineages, backgrounds, and status with the ultimate goal of uncovering actionable drug targets.

The mTOR pathway plays a central role in regulation of cell growth, metabolism, and nutrient sensing. Its complexity and essentiality are further emphasized by its multiple regulatory complexes and negative feedback signaling loops. PIK3CA activating mutations lead to hyperactive signaling downstream of mTORC1, and suggest a tumor-promoting function for mTOR signaling in TNBC^25^. Here, we identified 11 upstream mTOR regulators (activators and suppressors) as important cancer drivers in TNBC. Direct functions for mTORC2 and GATOR2 in TNBC tumorigenesis have not been addressed. Here, we functionally characterized and defined important functions for mTORC2/RICTOR and GATOR2/WDR59 in controlling mammary tumor growth in TNBC. GATOR2 is a multi-protein complex upstream of mTORC1 that is regulated by nutrient sensors, that include a family of stress-regulated proteins known as Sestrins. In the absence of amino acids, Sestrin1/2 bind to the GATOR2 complex to block its function and further inhibit mTORC1 lysosomal localization and activity^45^. In the presence of specific amino acids, particularly leucine, Sestrin1/2 dissociate from GATOR2 thereby relieving the GATOR1 inhibition of mTORC1. However, Sestrin3 binds constitutively to GATOR2, and is irresponsive to amino acids^45^, raising the possibility that it may function to mediate stress-related signals through changes in its expression. Indeed, p53 can block mTORC1 signaling through upregulation of Sestrins upon genotoxic stress in human cancer cells^64^. Considering the high frequency of TP53 mutations in TNBC, this implies that in these tumors the loss of p53 leads to a loss of Sestrins as gatekeepers of mTORC1 signaling. Using a CRISPR activation system we also showed that inducing sestrin3 expression which constitutively bind to and inhibit GATOR2 activity^45^, significantly blocked primary mammary tumor formation in TNBC in vivo, further highlighting the prominent role played by these gatekeepers in the control of tumorigenesis. PIK3CA and TP53 mutations are the two most frequent mutated genes in TNBCs. In silico METABRIC patient dataset analysis showed that more than 60% of TNBC patients carrying a PIK3CA mutation also harbor mutations in TP53. Moreover, patients harboring the double mutation phenotype exhibit the worst overall survival outcome. Thus, our results suggest that the double mutations provide cancer cells with a further selective advantage and increased tumorigenic potency through activation of mTORC1 signaling. While Hippo signaling is a classic tumor-suppressive pathway in many cancer types, genetic alterations in this pathway occur at a low frequency in cancer^65^. Furthermore, molecular classifications of TNBC have not identified any Hippo pathway member as a signature for this breast cancer subtype. Our results revealed a prominent role for Hippo signaling as a tumor-suppressive pathway in TNBC. In particular, we found the tumor-suppressive action of two Hippo members, Salvador and FRMD6, to be mediated through inhibition of the oncogene YAP and discovered a therapeutic application in targeting YAP in TNBC.

Our screen revealed two distinct signaling pathways as master regulators of tumorigenesis in TNBC. We applied our findings to develop a potential target combination therapy for TNBC by blocking the two druggable targets, YAP and mTOR1/2, using verteporfin and torin1, respectively. Importantly, and of clinical relevance, targeting the two pathways simultaneously resulted in a synergism between the 2 drugs and produced stronger anti-tumorigenic effects than targeting them individually. The potential clinical impact of this combination therapy is further illustrated in patient-derived xenograft where the two drugs also showed synergism in preventing tumor growth. From a mechanistic perspective, we found that blocking the mTORC2 and Hippo pathways resulted in a combination of macropinocytosis and apoptosis-mediated cell death and that torin1-induced macropinocytosis led to increased verteporfin intake by cancer cells, further enhancing the verteporfin apoptotic response in cancer cells.

Overall, these results underline the potential for combination treatments as better and more durable therapeutic options compared to monotherapy approaches. Finally, as no efficient target treatment currently exists for TNBC and while current clinical trials using PI3K inhibitors as monotherapy have not yielded significant improvement for patient outcomes^66–68^, our proposed combination therapeutic modality should prove especially beneficial for these TNBC patients.

## Methods

### Cell Lines

MDA-MB231 and HEK293FT were maintained in Dulbecco’s Modified Eagle Medium (DMEM) (Wisent Bio) with 10% fetal bovine serum (FBS) (Gibco). SUM159PT and SUM149PT were maintained in Ham’s F-12 media with 5% FBS, 5 μg/mL insulin and 1 μg/mL hydrocortisone (Wisent Bio). SUM1315MO2 was cultured in Ham’s F-12 media with 5% FBS, 5 μg/mL insulin and 10 ng/mL EGF. Primary human mammary epithelial cells (HuMEC) were cultured in HuMEC Basal Serum-Free Medium Supplemented with HuMEC Supplement and bovine pituitary extract (ThermoFisher). SUM cell lines were obtained from Dr. Stephen Ethier. Detailed information for these cell lines is available at Breast Cancer Cell Line Knowledge Base (www.sumlineknowledgebase.com). MDA-MB231 was obtained from ATCC. HEK293FT was obtained from Genhunter. HuMEC was purchased from ThermoFisher Scientific. All the cell lines were tested by PCR kit for mycoplasma by Diagnostic Laboratory from Comparative Medicine and Animal Resources Centre (McGill University). All cell lines are mycoplasma negative.

### CRISPR knockout and CRISPR activation plasmid cloning

For knockout genes, LentiCRISPR v2 backbone vector was a gift from Feng Zhang (Addgene plasmid #52961). Cloning was performed as described in the Addgene protocol^69^. Oligo sequences for sgRNAs KO targeting each gene (Non-targeting, *WDR59*, *RICTOR*, *SAV1*, *FRMD6*) listed in Supplementary Table 2. 5 µg LentiCRISPR v2 vector was digested and dephosphorylated by for 30 min at 37 °C. The digested plasmid was then gel-purified by QIAquick Gel Extraction Kit (Qiagen). The pair of oligos for each gene were phosphorylated and annealed using T4 PNK enzyme in a thermocycler by incubating 30 min at 37 °C and 5 min at 95 °C and ramp down to 25 °C. Annealed oligos were diluted at 1:200 and ligated together with digested vector using Quick ligase (NEB) for 20 min at room temperature. For CRISPRa, LentiSAMv2 (Addgene plasmid #75112) and LentiMPH v2 (Addgene plasmid #89308) were kindly provided by Dr. Feng Zhang^70^. The CRISPRa system, also known as CRISPR/dCas9 Synergistic Activation Mediator (SAM) system includes the LentiMPH v2 construct which encodes for the MS2-P65-HSF1 activator helper complex with a 2A hygromycin resistance marker and the LentiSAMv2 empty vector containing a modified Cas9-VP64, MS2 loops at tetraloop and stemloop 2, as well as a blasticidin resistance marker. Golden-Gate sgRNA cloning on LentiSAMv2 empty vector were performed as described in the protocol^70^. Oligo sequences for sgRNAs targeting each gene (*SESN3*, *WDR59*, *RICTOR*, *SAV1*, and *FRMD6*) listed in Supplementary Table 2. Briefly, oligo primers for each gene were phosphorylated and annealed using T4 PNK enzyme in a thermocycler by incubating 30 min at 37 °C and 5 min at 95 °C and ramp down to 25 °C. Annealed oligos were diluted at 1:10 and ligated together with lentiSAMv2 vector using T7 ligase (Enzymatics) Quick ligase (NEB) for 20 min at room temperature. The cloned vectors were then transformed into Stbl3 bacteria (Invitrogen) and streaking it onto an LB agar plate for ampicillin selection overnight at 33 °C. Plasmid Miniprep was prepared using Qiagen plasmid miniprep kit.

### Lentiviral production and infection

The HEK293T cell lines were transfected overnight at 37 °C using 15 µg cloned vector for each gene, 4.5 µg pMD2.G (Addgene #12259) and 12 µg psPAX2 (Addgene #12260). psPAX2 and pMD2.G were a gift from Didier Trono. After 24 h of virus production, the medium containing virus was collected, and cell debris was removed by centrifugation at 300 × *g* for 10 min. The virus in the supernatant was added to infect bulk SUM159 cells overnight in the medium with 8 µg/mL of polybrene. After 36 h infection, cells were then subjected to puromycin selection for 7 days.

For CRISPR activation, SUM159 cells were first transduced with LentiMPH v2 and then selected with hygromycin, and then transduced with LentiSAMv2 carried specific sgRNA for each gene, followed by blasticidin selection for 4 days.

### Genomic DNA cleavage assay

The genomic DNA cleavage assays for gene knockouts were performed using GeneArt Genomic Cleavage Detection Kit (Invitrogen) according to the manufacturer’s protocol. Briefly, genomic DNA was extracted from 5 × 10^5^ lentiCRISPRv2-knockout bulk cells. Primers were designed to amplify the specific Cas9/sgRNA genetically modified region by PCR. The primer sequences were listed in Supplementary Table 2. The modifications (the insertion, deletion, or mismatched DNA) of the interested region from the PCR products were then cleaved and detected by the Detection Enzyme from GeneArt Genomic Cleavage Detection Kit.

### Genome-wide library lentiviral production and infection

Human genome-wide CRISPR/cas9 knockout pooled library GeCKOv2 was a gift from Feng Zhang (Addgene#1000000048). The amplification and virus production of GeCKOv2 library A were performed as described in the Addgene protocol^69^. SUM159 cells were plated at a density of 3 × 10^6^ cells per well in 12 well plates and polybrene was added to a final concentration of 8 µg/mL. Viruses were titered and optimal virus concentrations allowing for 30% cell survival were used. Following spinfection at 800 × g for 2 h at 32 °C, cells were incubated overnight, trypsinized, pooled, and transferred in T225 flasks at a density of 3×10^6^ cells per flask. After 24 h, puromycin (2 µg/mL) was added for selection for 7 days. After 7 days, 30 million cells were frozen at −80 °C for genomic DNA extraction and deep-sequencing. The remaining cells were prepared for transplantation in animal model.

### Genomic DNA Extraction

Genomic DNA extraction for genome-wide knockout cells and tumor samples were performed as described in the study^18^. 30 million cells or 200 mg grinded tumor tissues from each sample were lysed in 6 mL of NK Lysis Buffer (50 mM Tris, 50 mM EDTA, 1% SDS, pH 8) and 30 µL of 20 mg/mL Proteinase K (Qiagen). Cell lysates were incubated at 55 °C for 1 h (cell pellet) and overnight for tumor tissues. RNAse A (Qiagen) was added at a final concentration of 0.05 mg/mL and incubated at 37 °C for 30 min. The samples were then cooled on ice for 10 min prior to adding 2 mL of ice-cold 7.5 M ammonium acetate (Sigma). The samples were vortexed at high speed for 20 s and centrifuged at 4000 × *g* for 10 min and supernatants were collected and mixed with isopropanol for DNA precipitation. Following centrifugation at 4000 × *g* for 10 min, supernatants were carefully decanted, and pellets washed in 70% cold ethanol, air-dried and resuspended in 500 µL 1× TE Buffer at 65 °C for 1 h. The gDNA concentration was measured using the Epoch Microplate Spectrometer (ThermoFisher).

### Library preparation and sequencing

All PCR reactions were performed using Herculase II Fusion DNA Polymerase (Agilent) and total number of reactions were based on extracted gDNA yields. PCR1 reactions were prepared by mixing 20 µL Herculase 5× Buffer, 1 µL of 100 mM dNTP, 2.5 µL of Adapter Primer F, 2.5 µL of Adapter Primer R, 1 µL Herculase II Fusion Enzyme, 10 µg of the gDNA extracted and PCR grade water to a final 100 µL volume. PCR1 reactions were performed using a thermocycler (98 °C for 2 min, 98 °C for 10 s, 60 °C for 20 s, 72 °C for 30 s, and 72 °C for 2 min for 18 cycles). All PCR1 reactions were then pooled and kept at −20 °C. For PCR2, 8 reactions were performed for each sample in a total 100 µL volume (20 µL Herculase 5× Buffer, 1 µL of 100 mM dNTP, 2.5 µL of Adapter Primer F, 2.5 µL of Adapter Primer R, 1 µL Herculase II Fusion Enzyme, 5 µL of PCR1 amplicon and 68 µL of PCR grade water). PCR2 reactions were performed as described for PCR1. Final PCR products were run on a 2% gel and extracted and purified using the QIAquick PCR & Gel Cleanup Kit (Qiagen) and subjected to next generation sequencing by Quebec Genome Center. 80 cycle and 20 million reads for each sample were performed by Hiseq 2500.

### Bioinformatics

MAGeCK-VISPR (0.5.3)^71^ was used for mapping back the reads to sgRNA CRISPR library. Non-biological experimental variation (batch effect) was adjusted using ComBat^72^. Log_2_ fold change (LogFC) was calculated to determine the change in abundance of each guide in each sample. Robust Rank Aggregation values (*p* values) were determined using the MAGeCK algorithm (version 0.5.3), as described in Li *et al*^71^.

### Western Blot

Upon reaching 80% confluency, cells were washed twice with ice-cold PBS (Wisent Bio), collected, and lysed in ice-cold cell buffer (50 mM Tris-HCl, 150 mM NaCl, 1% Triton X-100, 1 mM EDTA, 100 mM Na_3_VO_4_, and  1× protease inhibitors, phosphatase inhibitor cocktails). The tumor was grinded with a mortar and pestle on dry ice. 50 mg tissue was then homogenized in cold lysis buffer and incubated on ice for 1 h. The lysate was then centrifuged at 16,000 × *g* for 20 min at 4 °C. The protein concentration of the supernatant was determined using the BCA Kit (ThermoFisher). Samples were boiled at 95 °C for 5 min in loading buffer (10% SDS, 0.313 M Tris-HCl, pH 6.8, 50% glycerol, 0.5% Bromophenol Blue, and 0.5 M DL-Dithiothreitol) prior to loading on gel. Following electrophoresis, proteins were transferred onto nitrocellulose and blocked for 1 h (5% non-fat dry milk) at room temperature. Incubation with primary antibodies listed in Supplementary Table 3 was performed overnight at 4 °C. Following 1 h incubation with specific secondary antibodies, membranes were washed, revealed by ECL, and data analyzed using the ChemiDoc Touch Instrument (Bio-Rad).

### Drug matrix design and cell viability assay

Cells (1,000 per well) were seeded in 96 well plates and treated or not for 72 h with different doses of torin1 and verteporfin (Selleckchem) as well as in combination, as indicated in the figures. Cells were incubated with 100 µL of 10% PrestoBlue Reagent (ThermoFisher), at 37 °C for 2 h and cell viability was assessed through fluorescence measurement (excitation/emission at 560/590 nm). The percentage inhibition was calculated from the cell viability upon treatments. The percentage inhibition was used to quantify the synergy score using SynergyFinder https://synergyfinder.fimm.fi/.

### Apoptosis assay

SUM159 cells were treated with or without torin1, verteporfin, and in combination for 3 days and subjected to stain with Annexin V FITC and PI using Annexin V Apoptosis Detection Kit (Santa Cruz) for 15 min at room temperature according to the manufacturer’s protocol. Percentage of Annexin V + /PI + (late apoptosis) was measured by flow cytometry FACSCanto II and quantified by FlowJo v10 software.

### Macropinocytosis

SUM159 and HuMEC cells were plated in coverslips for 2 days and treated with 50 nM torin1 for 24 h. 25 µg/uL lucifer yellow was added at the beginning of treatment. After 24 h, cells were fixed by 3.7% formaldehyde for 10 min and then permeabilized with 0.1% Triton X-100 for 15 min at room temperature. After blocking for 30 min in 2% BSA, cells were incubated with 1:300 LAMP1 primary antibody for overnight at 4 °C and then secondary anti-rabbit Alexa Fluor 568 (Invitrogen) for 1 h at room temperature. The cells were then mounted on the microscope slides and scanned using the confocal microscope (Zeiss LSM780) at 63×objective. LY was detected by Alexa Fluor 430 channel, and LAMP1 was detected by Alexa Fluor 568. Flow cytometry analysis of LY (detected at excitation /emission 430/536 nm) in drug-treated cells was measured by LSRFortessa. FlowJo v10 was used for analysis of LY high cells.

### Verteporfin uptake

SUM159 and HuMEC Cells (10000 cells/well) were seeded in 96 well black plates and treated or not for 72 h with different doses of torin1 and verteporfin as well as in combination, as indicated in the figures. After 72 h, medium was removed, and cells were washed with PBS for three times. Verteporfin autofluorescent signal was assessed by fluorescent plate reader (excitation/emission at 650/720 nm).

### In vivo Xenograft studies

All mice were housed and handled in accordance to the approved guidelines of the Canadian Council on Animal Care (CCAC) “Guide to the Care and Use of Experimental Animals”. All experiments were performed under the approved McGill University Animal Care protocol (AUP # 7497 to JJL). Housing condition for mice: Temperature = 21 C + /− 1 C; Humidity = 40–60%RH + /− 5%RH; Lighting = 12 h. ON / 12 h, OFF daily cycle; Genome-wide library infected SUM159 cells (30 × 10^6^/mouse) were subcutaneously injected into the right flank of NSG mice. For individual gene knockout or activation validation, transduced SUM159 knockout or activation cells (1 × 10^6^/mouse) were diluted 1:1 in Matrigel (BD Bioscience) and then inoculated in the mammary fat pads of 8-week old, female NSG mice to generate breast tumors. Tumor sizes were measured with a digital electronic caliper three times per week and allowed to reach maximum volume of 1000 mm^3^ prior to euthanasia. Tumor volumes were calculated according to the following formula: [4/3 × π × (length/2) × (width/2)2] to generate a growth curve.

### Patient-derived Xenograft (PDX) and drug treatment

The TNBC PDX model TM00096 was obtained from The Jackson Laboratory. Detailed patient information is available on the company website. PDX primary tumor tissues at passage four were minced and subcutaneously transplanted into NSG mice. Tumor sizes were monitored with a digital electronic caliper and allowed to develop close to 200 mm^3^. Mice were separated into four groups (6 mice per group) based on similar average size. Animals were subjected to torin1 and verteporfin alone or in combination through daily i.p. injections at the indicated doses for 2 weeks.

### Immunohistochemistry

PDX tumor tissues were fixed in 10% formalin for 48 h and then embedded and section into 5 µm per slide. The slides were then boiled with 10 mM citrate buffer (pH 6.0) at 95 °C for 20 min. The slides were stained with PCNA (1:300) for 1 h. HRP Polymer & DAB Plus Chromogen Kit (Thermo Scientific) was used for detection. The images were acquired using the ToupView software, from three random regions within each tumor sample (*n* = 5 tumor samples per group) at 20× objective. Quantification of PCNA positive tumor cells was performed using the ImageJ plugin ImmunoRatio^73^.

### Statistical analyses

All results are presented as the mean±SEM for at least three repeated individual experiments unless otherwise indicated. The difference between groups was analyzed using two-sided unpaired Student’s *t*-test unless otherwise indicated, and *P < 0.05 was considered statistically significant.

### Reporting summary

Further information on research design is available in the Nature Research Reporting Summary linked to this article.

## Supplementary information

Supplementary Information

Reporting Summary

## Data Availability

METABRIC and TCGA pancancer DATASETs is available at the following links (https://www.cbioportal.org/study/summary?id=brca_metabric;https://www.cbioportal.org/study/summary?id=brca_tcga_pan_can_atlas_2018; https://xenabrowser.net/heatmap) Source data are provided with this paper.

## Source data

Source Data
