# Supplementary material for: In vivo genome-wide CRISPR screen reveals breast cancer vulnerabilities and synergistic mTOR/Hippo targeted combination therapy
Source: Nat Commun. 2021 May 24;12:3055. doi: 10.1038/s41467-021-23316-4 (PMC8144221; doi:10.1038/s41467-021-23316-4)
Supplement: Supplementary file 1 — Supplementary Information [file 41467_2021_23316_MOESM1_ESM.pdf]

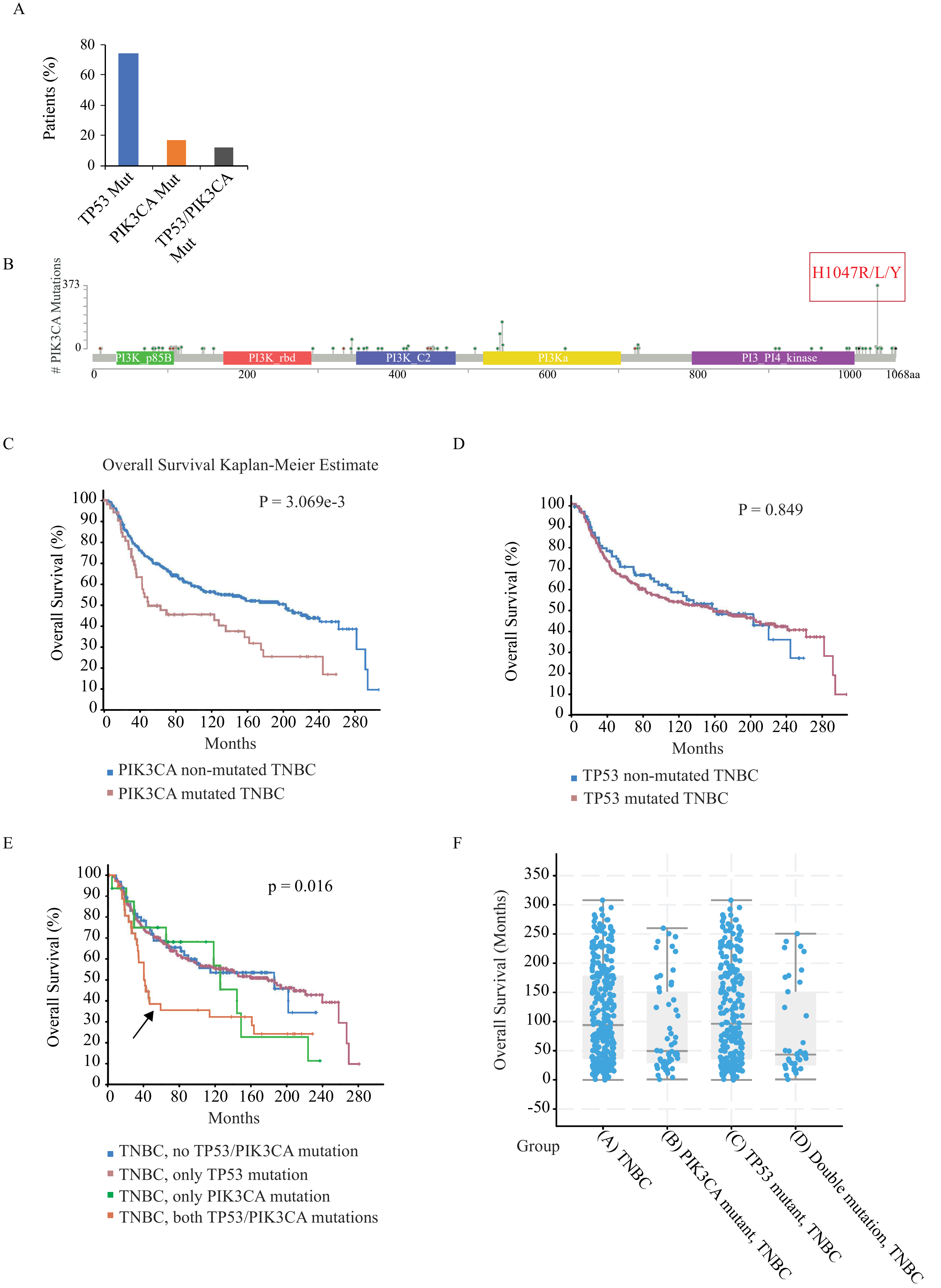

Suppl Figure 1

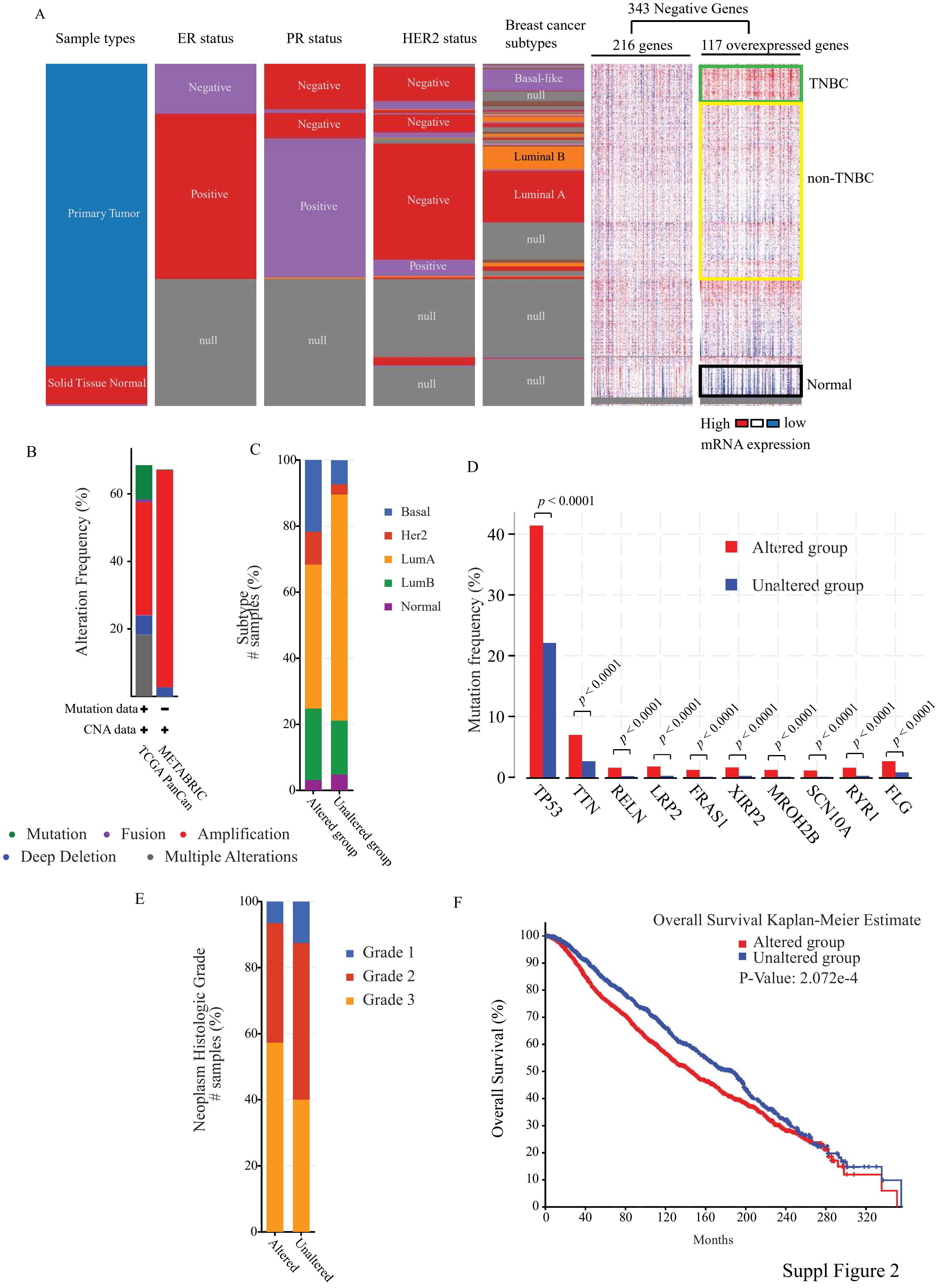

Suppl Figure 2

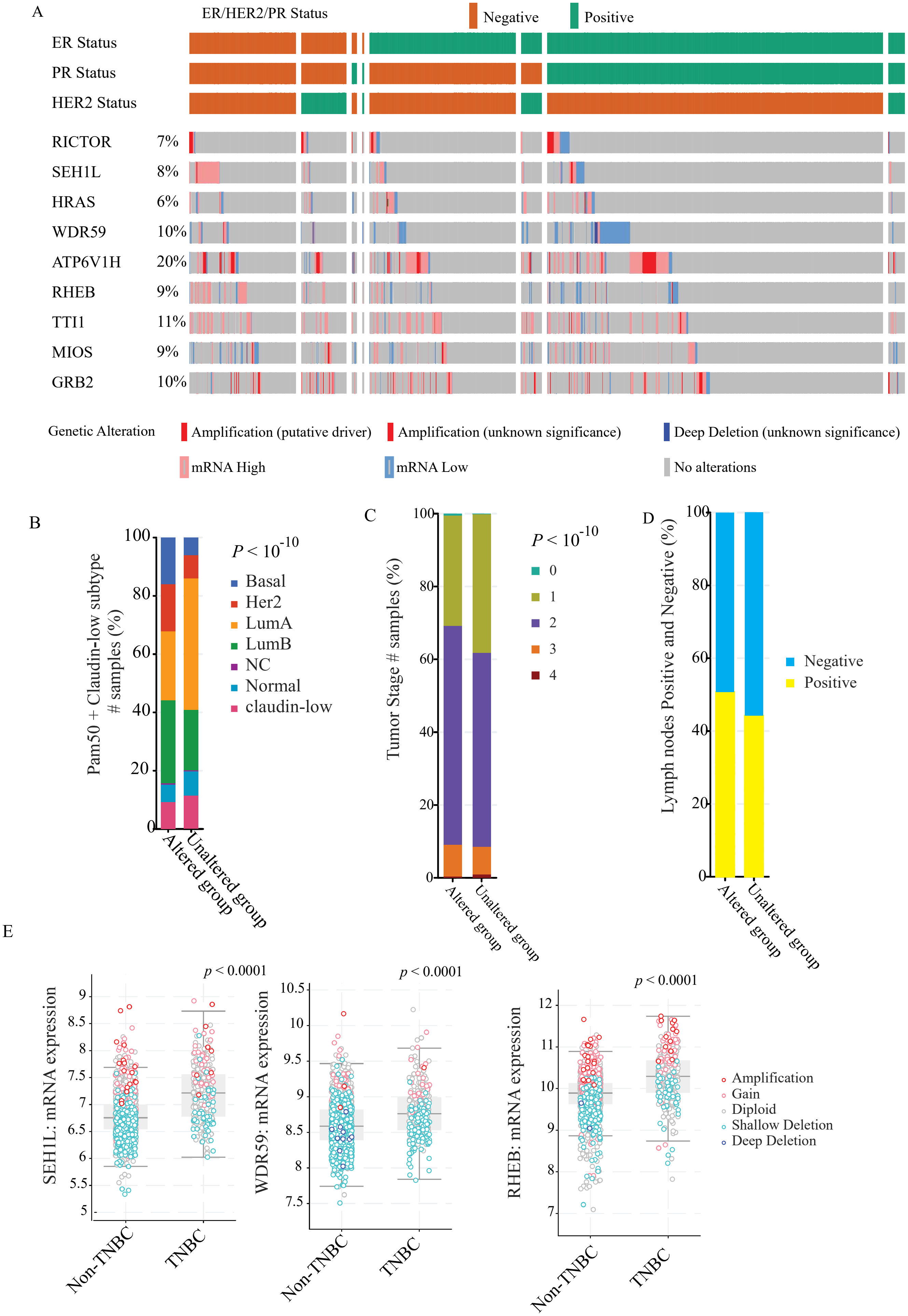

Suppl Figure 3

A

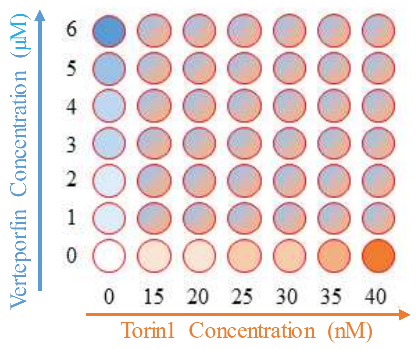

B

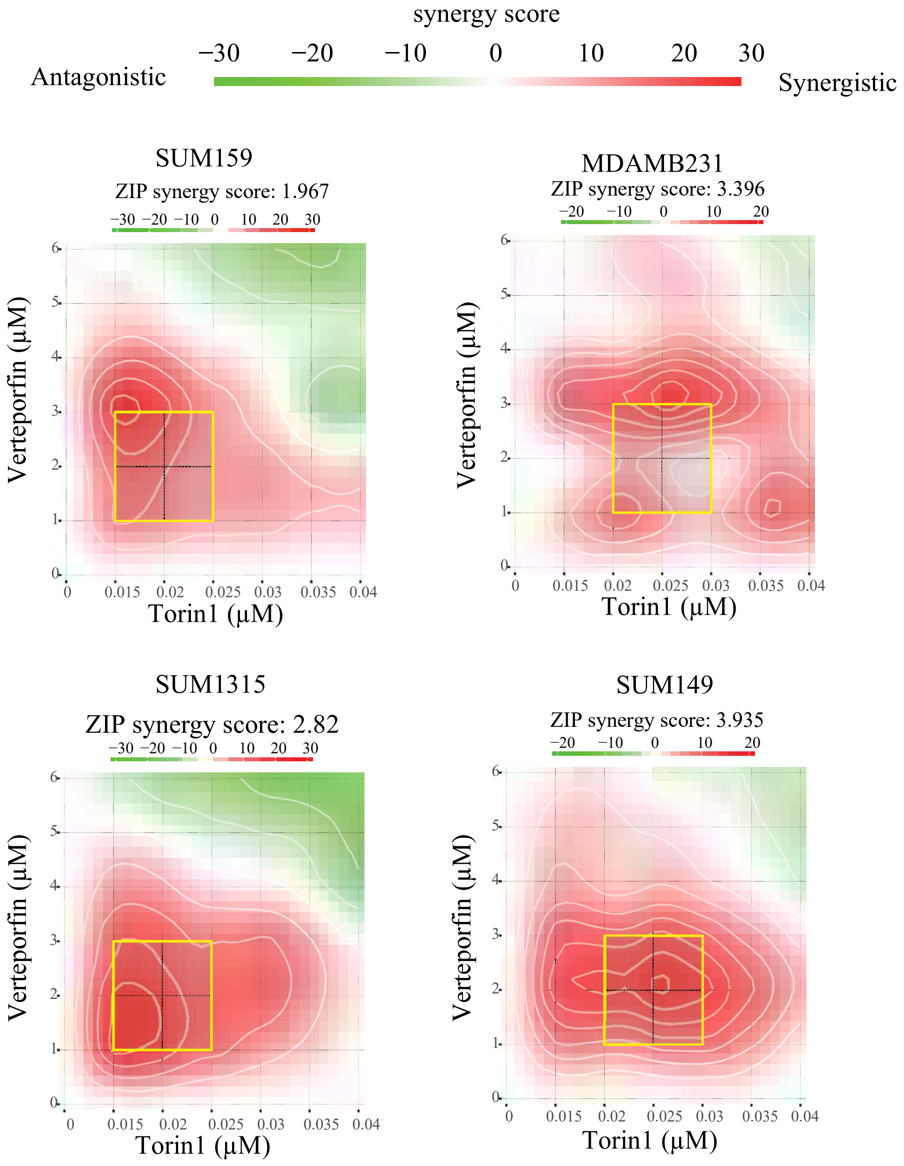

Suppl Figure 4

Suppl Figure 5

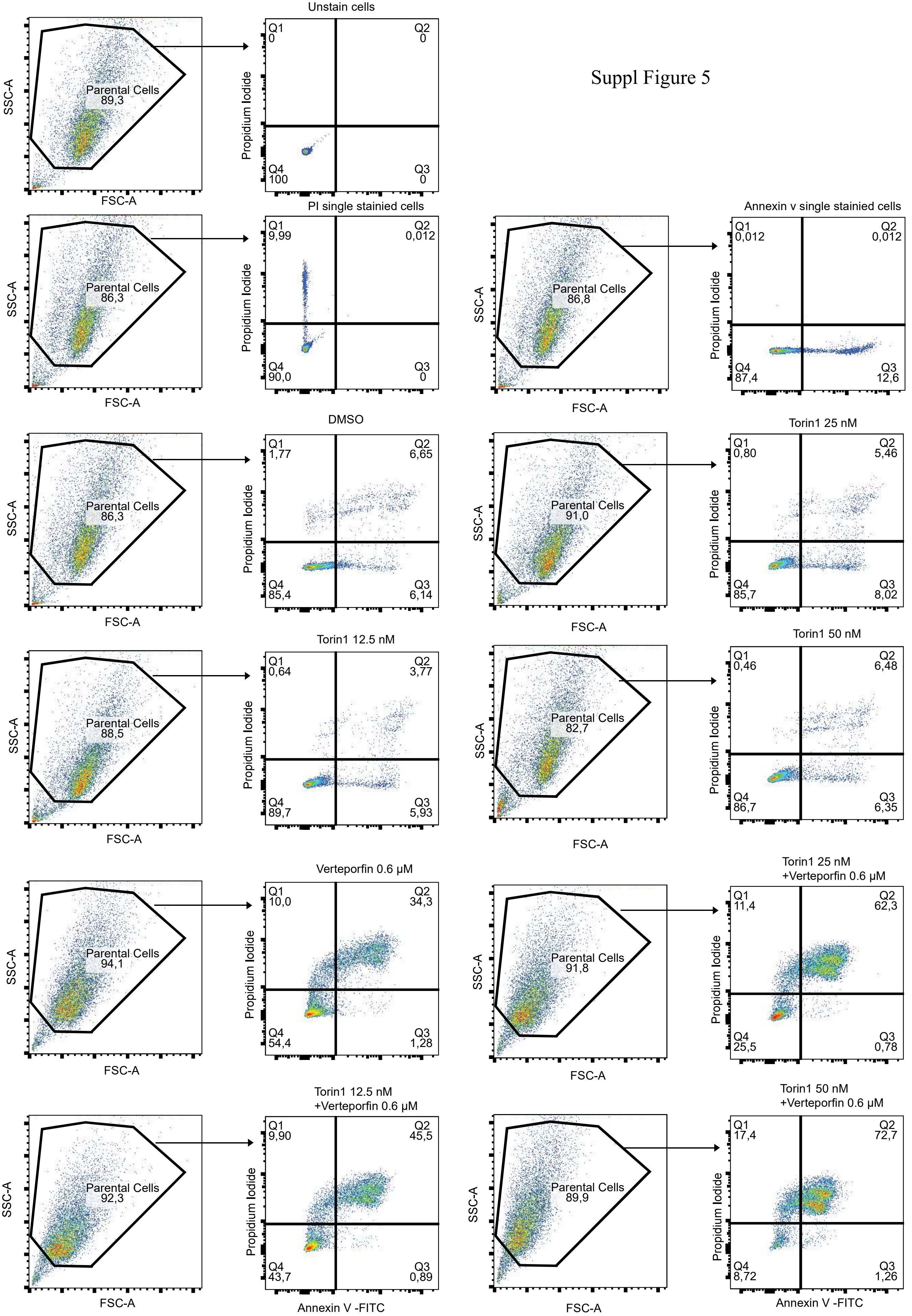

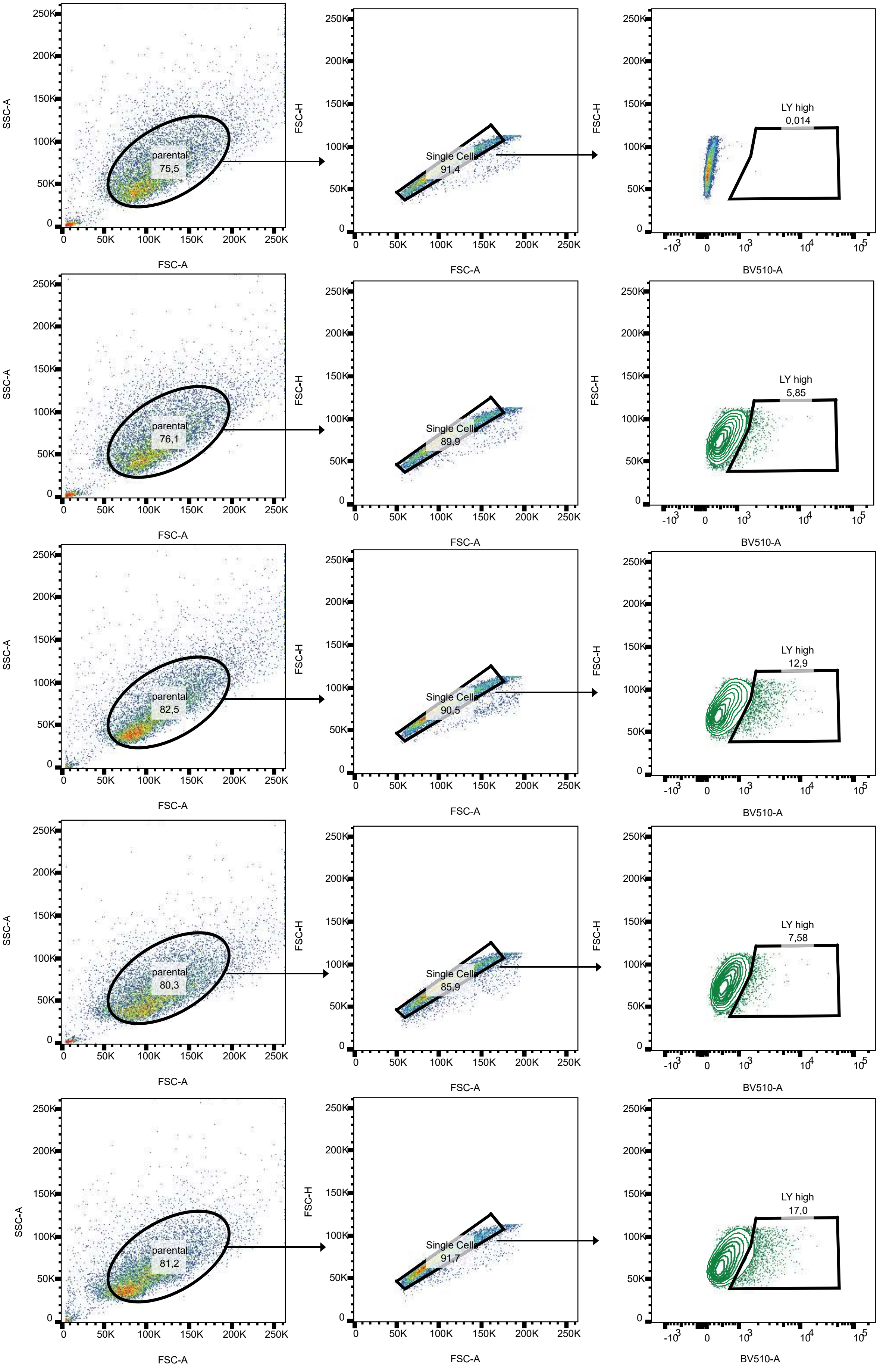

Suppl Figure 6

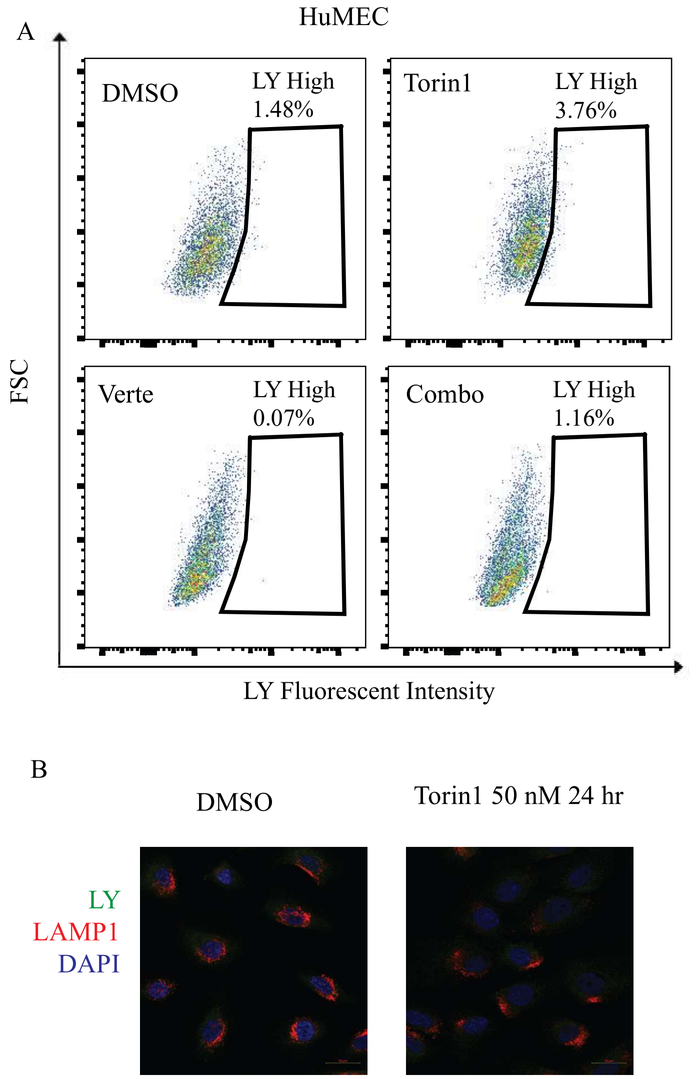

Suppl Figure 7

Positive Hit Rank

id	num	neg|score	neg|p-value	neg|fdr	neg|rank	neg|goodsgrna	neg|lfc	pos|score	pos|p-value	pos|fdr	pos|rank	pos|goodsgrna	pos|lfc
UBE2M	3	1.000	1.000	1.000	21668	0	8.418	0.000	0.000	0.001	1	3	8.418
PTPN14	3	1.000	1.000	1.000	21667	0	2.540	0.000	0.000	0.001	2	3	2.540
SOX4	3	1.000	1.000	1.000	21666	0	1.841	0.000	0.000	0.001	3	3	1.841
TAOK1	3	1.000	1.000	1.000	21665	0	1.796	0.000	0.000	0.001	4	3	1.796
PTPN12	3	1.000	1.000	1.000	21664	0	1.470	0.000	0.000	0.001	5	3	1.470
PPP4R2	3	1.000	1.000	1.000	21663	0	2.232	0.000	0.000	0.001	6	3	2.232
CMTR2	3	1.000	1.000	1.000	21662	0	1.825	0.000	0.000	0.001	7	3	1.825
NF2	3	0.969	0.970	1.000	20788	0	7.471	0.000	0.000	0.001	8	2	7.471
CAND1	3	0.970	0.970	1.000	20808	0	5.469	0.000	0.000	0.001	9	2	5.469
AFAP1	3	1.000	1.000	1.000	21661	0	2.251	0.000	0.000	0.001	10	3	2.251
KIRREL	3	0.995	0.995	1.000	21467	0	3.356	0.000	0.000	0.001	11	2	3.356
KEAP1	3	1.000	1.000	1.000	21653	0	3.164	0.000	0.000	0.001	12	2	3.164
ABL1	3	1.000	1.000	1.000	21622	0	2.694	0.000	0.000	0.003	13	2	2.694
ABL2	3	1.000	1.000	1.000	21660	0	1.111	0.000	0.000	0.004	14	3	1.111
MAP4K4	3	0.998	0.998	1.000	21590	0	2.287	0.000	0.000	0.006	15	2	2.287
TIPRL	3	0.879	0.879	1.000	18618	0	2.081	0.000	0.000	0.006	16	2	2.081
AMBRA1	3	0.966	0.966	1.000	20683	0	2.056	0.000	0.000	0.007	17	2	2.056
PCBP2	3	1.000	1.000	1.000	21658	0	1.932	0.000	0.000	0.007	18	2	1.932
TSC1	3	0.934	0.934	1.000	19921	0	1.610	0.000	0.000	0.013	19	2	1.610
SAV1	3	0.859	0.859	1.000	18198	1	1.598	0.000	0.000	0.021	20	2	1.598
FKBP1A	3	1.000	1.000	1.000	21652	0	1.429	0.000	0.000	0.024	21	2	1.429
STK40	3	1.000	1.000	1.000	21651	0	0.936	0.000	0.000	0.063	22	2	0.936
RNF7	3	0.948	0.948	1.000	20274	0	0.987	0.000	0.000	0.063	23	2	0.987
ZEB2	3	0.985	0.985	1.000	21195	0	0.968	0.000	0.000	0.077	24	2	0.968
FRMD6	3	0.993	0.993	1.000	21418	0	0.882	0.000	0.000	0.077	25	2	0.882
SPTAN1	3	0.991	0.991	1.000	21357	0	0.824	0.000	0.000	0.081	26	2	0.824
LILRA1	3	0.826	0.831	1.000	17521	1	0.987	0.000	0.000	0.081	27	2	0.987
SPR	3	0.882	0.882	1.000	18682	0	0.727	0.000	0.000	0.089	28	2	0.727
IL1R1	3	0.992	0.992	1.000	21399	0	0.754	0.000	0.000	0.101	29	2	0.754
PTEN	3	0.974	0.974	1.000	20912	0	1.130	0.000	0.000	0.101	30	1	1.130
ZSWIM6	3	1.000	1.000	1.000	21645	0	0.730	0.000	0.000	0.104	31	2	0.730
TXNDC17	3	1.000	1.000	1.000	21655	0	0.749	0.000	0.000	0.105	32	2	0.749
PDCD10	3	0.998	0.998	1.000	21572	0	0.698	0.000	0.000	0.109	34	2	0.698
SOBP	3	0.611	0.702	1.000	14482	1	0.749	0.000	0.000	0.116	35	2	0.749
CLRN2	3	0.910	0.910	1.000	19343	0	0.580	0.000	0.000	0.132	36	2	0.580
COCH	3	0.715	0.757	1.000	15807	1	0.662	0.000	0.000	0.132	37	2	0.662
FBXL5	3	0.328	0.482	1.000	9662	1	0.622	0.000	0.000	0.132	38	2	0.622
CCND1	3	0.999	0.999	1.000	21603	0	0.618	0.000	0.000	0.134	39	2	0.618
UBE2N	3	1.000	1.000	1.000	21659	0	0.629	0.000	0.000	0.145	40	2	0.629
ARHGAP35	3	1.000	1.000	1.000	21657	0	0.632	0.000	0.000	0.145	41	2	0.632
NFXL1	3	0.964	0.964	1.000	20634	0	0.589	0.000	0.000	0.158	42	2	0.589
PAX8	3	0.168	0.314	1.000	6235	1	0.561	0.000	0.000	0.160	43	2	0.561
EHMT1	3	0.767	0.788	1.000	16504	1	0.583	0.000	0.000	0.169	44	2	0.583
AP2S1	3	0.998	0.998	1.000	21585	0	0.519	0.000	0.000	0.211	45	2	0.519
IKBKB	3	0.999	0.999	1.000	21618	0	0.516	0.000	0.000	0.211	46	2	0.516
CAB39	3	1.000	1.000	1.000	21649	0	0.423	0.000	0.001	0.244	48	2	0.423

Negative Hit Rank

id	num	neg|score	neg|p-value	neg|fdr	neg|rank	neg|goodsgrna	neg|lfc	pos|score	pos|p-value	pos|fdr	pos|rank	pos|goodsgrna	pos|lfc
IARS2	3	1.23E-07	2.28E-07	0.001238	3	3	-5.7167	1	1	1	21666	0	-5.7167
MAD2L1	3	1.75E-07	6.85E-07	0.00297	4	3	-5.345	1	1	1	21665	0	-5.345
ALG11	3	3.21E-07	1.14E-06	0.004125	6	3	-5.6395	1	1	1	21664	0	-5.6395
YARS	3	7.15E-07	2.51E-06	0.006807	7	3	-5.2722	1	1	1	21663	0	-5.2722
RPL14	3	1.67E-06	5.71E-06	0.012151	10	3	-4.9663	1	1	1	21660	0	-4.9663
POLR3H	3	1.79E-06	6.17E-06	0.012151	11	3	-5.507	1	1	1	21659	0	-5.507
RPS17	3	2.48E-06	8.91E-06	0.016089	12	3	-4.6846	1	1	1	21658	0	-4.6846
DHX36	3	2.81E-06	1.03E-05	0.016172	13	3	-5.2056	1	1	1	21657	0	-5.2056
ARIH1	3	3.22E-06	1.12E-05	0.016172	15	3	-4.9727	1	1	1	21655	0	-4.9727
POLR3A	3	3.34E-06	1.12E-05	0.016172	16	3	-5.0513	1	1	1	21654	0	-5.0513
ELMO2	3	3.72E-06	1.30E-05	0.016227	18	3	-4.5182	1	1	1	21653	0	-4.5182
ATP6V0C	3	4.44E-06	1.53E-05	0.017457	19	3	-4.6834	1	1	1	21652	0	-4.6834
CDIPT	3	4.94E-06	1.80E-05	0.019554	20	3	-5.2382	1	1	1	21651	0	-5.2382
RPL3	3	5.62E-06	1.92E-05	0.019802	21	3	-4.6195	0.99999	1	1	21650	0	-4.6195
SNRPF	3	6.48E-06	2.26E-05	0.021246	22	3	-4.5447	0.99999	0.99999	1	21649	0	-4.5447
NIP7	3	6.62E-06	2.26E-05	0.021246	23	3	-4.381	0.99999	0.99999	1	21648	0	-4.381
WASH1	3	7.05E-06	2.35E-05	0.021246	24	3	-4.3906	0.99999	0.99999	1	21647	0	-4.3906
MAK16	3	7.59E-06	2.54E-05	0.02198	26	3	-4.6482	0.99999	0.99999	1	21646	0	-4.6482
FAM96B	3	8.25E-06	2.67E-05	0.022277	28	3	-5.2476	0.99999	0.99999	1	21645	0	-5.2476
SMC6	3	8.70E-06	2.86E-05	0.022919	29	3	-4.4445	0.99999	0.99999	1	21644	0	-4.4445
DUT	3	9.62E-06	3.18E-05	0.022937	31	3	-4.3377	0.99999	0.99999	1	21642	0	-4.3377
RPS15A	3	1.05E-05	3.45E-05	0.024114	32	3	-4.4075	0.99999	0.99999	1	21641	0	-4.4075
FERMT2	3	1.23E-05	4.04E-05	0.026063	33	3	-4.9063	0.99999	0.99999	1	21640	0	-4.9063
RPL24	3	1.25E-05	4.09E-05	0.026063	34	3	-4.3664	0.99999	0.99999	1	21639	0	-4.3664
POLR3C	3	1.40E-05	4.55E-05	0.026609	35	3	-5.0999	0.99999	0.99999	1	21638	0	-5.0999
ANAPC5	3	1.41E-05	4.59E-05	0.026609	36	3	-3.3211	0.99993	0.99993	1	21602	0	-3.3211
YKT6	3	1.43E-05	4.64E-05	0.026609	37	3	-4.2006	0.99999	0.99999	1	21637	0	-4.2006
TBCD	3	1.50E-05	4.73E-05	0.026609	38	3	-4.8475	0.99999	0.99999	1	21636	0	-4.8475
PDCD2	3	1.52E-05	4.82E-05	0.026609	39	3	-4.103	0.99998	0.99999	1	21635	0	-4.103
CUL2	3	1.57E-05	4.91E-05	0.026609	40	3	-4.7907	0.99998	0.99999	1	21634	0	-4.7907
RSL24D1	3	1.65E-05	5.09E-05	0.026926	41	3	-4.332	0.99998	0.99999	1	21633	0	-4.332
SS18L2	3	1.87E-05	5.73E-05	0.029585	43	3	-5.0882	0.99998	0.99999	1	21631	0	-5.0882
SPDYE2	3	2.12E-05	6.56E-05	0.032291	44	3	-3.5153	0.99998	0.99998	1	21630	0	-3.5153
AARS	3	2.16E-05	6.56E-05	0.032291	45	2	-5.0867	0.62501	0.62497	1	13225	0	-5.0867
GOLGA8O	3	2.34E-05	7.11E-05	0.0329	46	3	-4.8825	0.99998	0.99998	1	21629	0	-4.8825
DIS3	3	2.35E-05	7.20E-05	0.0329	47	3	-2.2548	0.92913	0.92922	1	19691	0	-2.2548
CENPM	3	2.41E-05	7.29E-05	0.0329	48	3	-4.0902	0.99998	0.99998	1	21628	0	-4.0902
TUBGCP4	3	2.61E-05	7.88E-05	0.034856	49	3	-4.1539	0.99997	0.99998	1	21627	0	-4.1539
HARS	3	2.72E-05	8.16E-05	0.03532	50	3	-4.2252	0.99997	0.99998	1	21626	0	-4.2252
RICTOR	3	2.82E-05	8.38E-05	0.03532	51	3	-4.7206	0.99997	0.99998	1	21625	0	-4.7206

NOP16	3	2.83E-05	8.48E-05	0.03532	52	3	-4.6498	0.99997	0.99998	1	21624	0	-4.6498
VARS	3	2.92E-05	9.02E-05	0.036004	54	3	-6.2664	0.99525	0.99535	1	21275	0	-6.2664
GTF3C4	3	2.95E-05	9.14E-05	0.036004	55	3	-4.6249	0.99997	0.99998	1	21623	0	-4.6249
SEH1L	3	3.11E-05	9.71E-05	0.037571	57	3	-4.1895	0.99997	0.99998	1	21621	0	-4.1895
ALDOA	3	3.34E-05	0.00010213	0.038153	59	3	-4.5669	0.99997	0.99998	1	21619	0	-4.5669
PPIL4	3	3.57E-05	0.00011127	0.040863	60	3	-4.0493	0.99996	0.99997	1	21618	0	-4.0493
DCPS	3	3.66E-05	0.00011401	0.041172	61	3	-3.9313	0.99996	0.99997	1	21617	0	-3.9313
MOCS3	3	3.91E-05	0.00012132	0.042558	62	3	-4.0063	0.99996	0.99997	1	21616	0	-4.0063
HAUS1	3	4.03E-05	0.00012497	0.042983	63	2	-4.4594	0.74215	0.74205	1	15710	0	-4.4594
CCT2	3	4.18E-05	0.00012909	0.043704	64	3	-4.5895	0.99996	0.99997	1	21615	0	-4.5895
PUF60	3	4.38E-05	0.00013594	0.04523	65	3	-3.9295	0.99995	0.99996	1	21613	0	-3.9295
YTHDF2	3	4.47E-05	0.00013777	0.04523	66	3	-4.0333	0.99996	0.99996	1	21614	0	-4.0333
MYC	3	4.93E-05	0.00015011	0.048122	67	3	-4.9128	0.99995	0.99996	1	21612	0	-4.9128
RPL7	3	5.00E-05	0.00015102	0.048122	68	3	-4.2442	0.99995	0.99996	1	21611	0	-4.2442
TIMM23	3	5.15E-05	0.00015467	0.048572	70	3	-3.8901	0.99995	0.99995	1	21610	0	-3.8901
GTF3C6	3	6.21E-05	0.00018712	0.057064	71	3	-4.4529	0.99994	0.99994	1	21609	0	-4.4529
PES1	3	6.27E-05	0.00018849	0.057064	72	3	-3.623	0.99994	0.99994	1	21608	0	-3.623
CCT8	3	6.38E-05	0.00019214	0.057064	73	3	-4.095	0.99994	0.99994	1	21607	0	-4.095
EIF3F	3	6.49E-05	0.00019489	0.057064	74	3	-4.0343	0.99994	0.99994	1	21606	0	-4.0343
CTDP1	3	6.69E-05	0.00019991	0.057126	76	3	-4.0856	0.99993	0.99993	1	21604	0	-4.0856
NSL1	3	6.73E-05	0.00020037	0.057126	77	3	-3.8938	0.99993	0.99993	1	21603	0	-3.8938
IMP4	3	7.19E-05	0.00021636	0.060885	78	3	-3.7259	0.99993	0.99993	1	21601	0	-3.7259
SYMPK	3	7.45E-05	0.0002255	0.062351	79	3	-3.9847	0.99993	0.99993	1	21600	0	-3.9847
SART3	3	7.51E-05	0.00022733	0.062351	80	3	-4.4305	0.99992	0.99993	1	21599	0	-4.4305
QARS	3	7.90E-05	0.00023692	0.06399	81	2	-5.7025	0.64127	0.64123	1	13562	0	-5.7025
RPL38	3	8.00E-05	0.00023921	0.06399	82	3	-4.9074	0.99992	0.99992	1	21598	0	-4.9074
RPL18	3	8.32E-05	0.00024698	0.065072	83	3	-3.9918	0.99992	0.99992	1	21597	0	-3.9918
PTPN11	3	8.44E-05	0.00024926	0.065072	84	3	-4.2565	0.99992	0.99992	1	21596	0	-4.2565
PKMYT1	3	8.56E-05	0.00025246	0.065123	85	3	-4.5125	0.99991	0.99991	1	21595	0	-4.5125
ENO1	3	9.01E-05	0.00026571	0.066947	86	3	-4.2606	0.99991	0.99991	1	21594	0	-4.2606
DCAF13	3	9.27E-05	0.00027211	0.067113	87	3	-3.6473	0.99991	0.99991	1	21593	0	-3.6473
SNRNP27	3	9.29E-05	0.00027257	0.067113	88	3	-3.6789	0.99991	0.99991	1	21592	0	-3.6789
HRAS	3	9.63E-05	0.00028079	0.067932	89	3	-3.7801	0.9999	0.9999	1	21591	0	-3.7801
POLE2	3	9.66E-05	0.00028216	0.067932	90	3	-4.8234	0.95979	0.95996	1	20399	0	-4.8234
UNC45A	3	0.0001028	0.00030912	0.073605	92	3	-4.0488	0.9999	0.9999	1	21588	0	-4.0488
TAF1B	3	0.0001054	0.00031826	0.073994	93	3	-4.2044	0.99989	0.99989	1	21587	0	-4.2044
SNRPD3	3	0.0001059	0.00031917	0.073994	94	3	-4.3866	0.99944	0.99944	1	21500	0	-4.3866
MMS22L	3	0.0001064	0.000321	0.073994	95	3	-3.8493	0.99989	0.99989	1	21586	0	-3.8493
DYRK1A	3	0.0001101	0.00033334	0.076011	96	3	-4.6138	0.99989	0.99989	1	21585	0	-4.6138
TOMM20	3	0.0001114	0.00033677	0.076011	97	3	-3.4555	0.99989	0.99989	1	21584	0	-3.4555
PRPF38B	3	0.0001142	0.00034248	0.076503	98	3	-4.8193	0.99989	0.99989	1	21583	0	-4.8193
NCAPD3	3	0.0001154	0.00034613	0.076531	99	3	-3.7606	0.99988	0.99989	1	21582	0	-3.7606

UBE2I	3	0.0001182	0.00035436	0.077558	101	3	-3.9528	0.99988	0.99988	1	21581	0	-3.9528
WDR59	3	0.0001202	0.00036258	0.077885	102	3	-3.5765	0.99988	0.99988	1	21580	0	-3.5765
SFPQ	3	0.0001203	0.00036304	0.077885	103	3	-5.0866	0.99988	0.99988	1	21579	0	-5.0866
TBCC	3	0.0001231	0.00036944	0.078006	104	3	-5.3977	0.99988	0.99988	1	21578	0	-5.3977
EIF2B2	3	0.0001235	0.00037081	0.078006	105	3	-3.6384	0.99988	0.99988	1	21577	0	-3.6384
RPP38	3	0.0001329	0.00039731	0.082778	106	3	-3.529	0.99987	0.99987	1	21576	0	-3.529
HJURP	3	0.0001356	0.00040279	0.082991	107	3	-4.0084	0.99986	0.99987	1	21575	0	-4.0084
BRF1	3	0.0001402	0.00041513	0.083471	108	3	-4.3946	0.99986	0.99986	1	21574	0	-4.3946
KDSR	3	0.0001404	0.00041604	0.083471	109	3	-3.1635	0.99986	0.99986	1	21573	0	-3.1635
CSTF3	3	0.0001437	0.00043341	0.085675	110	3	-5.2445	0.99961	0.99961	1	21523	0	-5.2445
ZCCHC14	3	0.0001445	0.00043889	0.085675	111	3	-3.8447	0.99986	0.99986	1	21572	0	-3.8447
ATP6V0D1	3	0.0001446	0.00043889	0.085675	112	3	-3.7754	0.99986	0.99986	1	21571	0	-3.7754
PSMB1	3	0.00015	0.0004494	0.086943	113	3	-3.4749	0.99985	0.99985	1	21570	0	-3.4749
EIF2S1	3	0.0001597	0.00047179	0.090021	114	3	-4.1773	0.99984	0.99984	1	21569	0	-4.1773
SRP54	3	0.0001655	0.0004887	0.091627	115	3	-3.5811	0.99983	0.99984	1	21568	0	-3.5811
EXOC3	3	0.0001672	0.00049053	0.091627	116	3	-3.1961	0.99983	0.99983	1	21567	0	-3.1961
PFN1	3	0.0001716	0.00050835	0.094143	117	3	-3.4723	0.99983	0.99983	1	21566	0	-3.4723
UTP18	3	0.0001737	0.00051383	0.094143	118	3	-4.2617	0.99983	0.99983	1	21565	0	-4.2617
CABIN1	3	0.0001753	0.00051703	0.094143	119	3	-3.6491	0.99982	0.99983	1	21564	0	-3.6491
LOC1001322	3	0.0001773	0.00052297	0.094431	120	3	-2.7023	0.99982	0.99983	1	21563	0	-2.7023
MARK3	3	0.0001809	0.00053211	0.095287	121	3	-3.5667	0.99982	0.99982	1	21562	0	-3.5667
PSMG4	3	0.0001866	0.00054582	0.09694	122	2	-5.2071	0.38739	0.38735	1	8248	0	-5.2071
RPL34	3	0.0001913	0.00055907	0.097069	123	3	-5.1044	0.99981	0.99981	1	21561	0	-5.1044
C9orf114	3	0.0001915	0.00055952	0.097069	124	3	-5.2171	0.99981	0.99981	1	21560	0	-5.2171
C3orf17	3	0.0001918	0.00055998	0.097069	125	2	-5.242	0.24896	0.24859	1	5326	0	-5.242
ANAPC4	3	0.0002007	0.00058374	0.100385	126	3	-3.5944	0.9998	0.9998	1	21559	0	-3.5944
ATP2A2	3	0.0002029	0.00059242	0.100814	127	3	-5.1233	0.9998	0.9998	1	21558	0	-5.1233
SNRNP40	3	0.0002039	0.00059562	0.100814	128	3	-3.6656	0.9998	0.9998	1	21557	0	-3.6656
MED6	3	0.0002057	0.00060019	0.100814	129	3	-3.7537	0.99979	0.99979	1	21556	0	-3.7537
RBM14	3	0.0002125	0.00062258	0.10377	130	3	-4.2262	0.99943	0.99944	1	21497	0	-4.2262
TIMM10	3	0.0002188	0.00064726	0.107059	131	3	-4.5855	0.93924	0.93938	1	19901	0	-4.5855
CIRH1A	3	0.0002267	0.0006701	0.109998	132	3	-4.4801	0.99977	0.99977	1	21555	0	-4.4801
ALG2	3	0.0002375	0.00070026	0.113233	133	3	-4.4271	0.98888	0.98901	1	21104	0	-4.4271
DNM1L	3	0.0002376	0.00070026	0.113233	134	3	-3.5952	0.99976	0.99976	1	21554	0	-3.5952
LAS1L	3	0.0002427	0.00071123	0.114116	135	3	-4.6031	0.99976	0.99975	1	21553	0	-4.6031
PTPMT1	3	0.0002438	0.00071625	0.114116	136	3	-4.1972	0.99976	0.99975	1	21552	0	-4.1972
BYSL	3	0.0002467	0.00072219	0.114223	137	3	-5.1504	0.99245	0.99254	1	21193	0	-5.1504
SAFB	3	0.0002514	0.00073499	0.11425	138	3	-3.3528	0.99975	0.99974	1	21551	0	-3.3528
DDX19A	3	0.000252	0.00073682	0.11425	139	3	-3.3543	0.99975	0.99974	1	21550	0	-3.3543
BCAS2	3	0.0002525	0.00073819	0.11425	140	3	-3.5284	0.99975	0.99974	1	21549	0	-3.5284
WDR70	3	0.0002581	0.00075875	0.116476	141	2	-3.9947	0.85115	0.85111	1	18005	0	-3.9947
BCL2L2-PABI	3	0.0002603	0.00076332	0.116476	142	3	-3.4992	0.99974	0.99973	1	21548	0	-3.4992

TRAIP	3	0.0002667	0.00078068	0.117677	143	3	-3.1708	0.99973	0.99973	1	21547	0	-3.1708
HYPK	3	0.000267	0.00078205	0.117677	144	3	-4.4604	0.99973	0.99973	1	21546	0	-4.4604
AIFM1	3	0.0002713	0.00079165	0.1183	146	3	-4.9224	0.99973	0.99972	1	21545	0	-4.9224
KIF4A	3	0.0002793	0.00081907	0.121558	147	3	-3.7276	0.99972	0.99972	1	21544	0	-3.7276
PSMA3	3	0.0002829	0.00082912	0.122213	149	3	-4.0336	0.99972	0.99971	1	21543	0	-4.0336
VPS25	3	0.0002854	0.00083552	0.122324	150	2	-5.1449	0.4788	0.47861	1	10171	0	-5.1449
YY1	3	0.0002913	0.00084877	0.12343	151	3	-3.8956	0.99971	0.99971	1	21542	0	-3.8956
RBM39	3	0.0002946	0.00085608	0.123663	152	3	-3.3829	0.99971	0.99971	1	21541	0	-3.3829
SUMO2	3	0.0003021	0.00087801	0.125992	153	3	-4.2002	0.9997	0.99969	1	21540	0	-4.2002
ELAC2	3	0.000305	0.00088441	0.126075	154	2	-2.402	0.37995	0.37989	1	8079	0	-2.402
RRP9	3	0.0003076	0.00089126	0.126221	155	3	-5.1105	0.99875	0.99876	1	21447	0	-5.1105
CCDC115	3	0.0003146	0.00092096	0.128492	156	3	-3.4438	0.99969	0.99968	1	21539	0	-3.4438
DDX6	3	0.0003161	0.00092782	0.128492	157	3	-2.9451	0.99968	0.99968	1	21538	0	-2.9451
PSMA6	3	0.0003162	0.00092782	0.128492	158	2	-3.538	0.87391	0.87404	1	18512	0	-3.538
TNFSF9	3	0.0003171	0.00093102	0.128492	159	3	-2.5697	0.99002	0.99015	1	21129	0	-2.5697
CCT4	3	0.0003287	0.00096437	0.131422	160	3	-5.0425	0.99961	0.99961	1	21525	0	-5.0425
RAPGEF2	3	0.0003291	0.00096437	0.131422	161	3	-3.3915	0.99967	0.99967	1	21537	0	-3.3915
POLR2K	3	0.0003317	0.00097306	0.131776	162	3	-5.0993	0.96717	0.96726	1	20551	0	-5.0993
NAA50	3	0.0003336	0.00097991	0.13188	165	3	-4.95	0.8931	0.89314	1	18943	0	-4.95
IRF2	3	0.0003425	0.0010073	0.132586	166	3	-3.4257	0.99966	0.99966	1	21535	0	-3.4257
NOP58	3	0.0003445	0.0010151	0.132586	167	2	-5.0518	0.77198	0.772	1	16354	0	-5.0518
IRX2	3	0.0003455	0.0010183	0.132586	168	3	-4.862	0.99965	0.99965	1	21534	0	-4.862
CENPI	3	0.0003485	0.0010288	0.132586	169	2	-4.9932	0.82022	0.82007	1	17358	0	-4.9932
GFER	3	0.0003487	0.0010293	0.132586	170	3	-4.83	0.99965	0.99965	1	21533	0	-4.83
KRTAP4-9	3	0.0003515	0.0010389	0.132586	171	2	-1	0.38685	0.38683	1	8239	0	-1
EXOC7	3	0.0003519	0.0010398	0.132586	172	3	-3.9095	0.96049	0.96068	1	20417	0	-3.9095
NAA25	3	0.000352	0.0010402	0.132586	173	3	-3.7499	0.99965	0.99965	1	21532	0	-3.7499
EIF3D	3	0.000355	0.0010471	0.132679	174	3	-4.5722	0.99964	0.99964	1	21531	0	-4.5722
PCNA	3	0.0003629	0.0010704	0.134843	175	3	-3.9416	0.99964	0.99963	1	21530	0	-3.9416
CCDC84	3	0.0003699	0.00109	0.135082	176	3	-5.0186	0.99936	0.99936	1	21489	0	-5.0186
PDCD11	3	0.000374	0.0011019	0.135082	177	3	-4.2746	0.99963	0.99962	1	21529	0	-4.2746
WDR26	3	0.0003762	0.0011088	0.135082	178	2	-4.8304	0.46667	0.46643	1	9915	0	-4.8304
YARS2	3	0.0003766	0.0011092	0.135082	179	3	-3.2818	0.99962	0.99962	1	21528	0	-3.2818
CYFIP1	3	0.0003769	0.0011097	0.135082	180	3	-4.0813	0.99962	0.99962	1	21527	0	-4.0813
TP53RK	3	0.0003814	0.0011216	0.135595	181	3	-3.511	0.99818	0.99819	1	21410	0	-3.511
HEATR1	3	0.0003848	0.001133	0.135595	182	3	-3.4548	0.99962	0.99961	1	21526	0	-3.4548
PHAX	3	0.0003856	0.0011348	0.135595	183	2	-4.7877	0.64982	0.64974	1	13748	0	-4.7877
DGCR6	3	0.0003883	0.0011389	0.135595	184	3	-3.3653	0.99961	0.99961	1	21524	0	-3.3653
GEMIN5	3	0.000392	0.0011485	0.13599	185	2	-4.8849	0.5285	0.52832	1	11210	0	-4.8849
PRDM9	3	0.0003952	0.0011567	0.136125	186	3	-3.6185	0.91322	0.91322	1	19366	0	-3.6185
EIF3I	3	0.0003973	0.0011622	0.136125	187	3	-4.8355	0.93811	0.93824	1	19875	0	-4.8355
TBCA	3	0.0004022	0.0011778	0.136451	188	3	-4.1223	0.9996	0.9996	1	21522	0	-4.1223

NUP155	3	0.0004068	0.0011887	0.136451	190	3	-4.3678	0.99959	0.99959	1	21520	0	-4.3678
CHEK1	3	0.0004081	0.0011947	0.136451	191	3	-3.8072	0.99959	0.99959	1	21519	0	-3.8072
SRP9	3	0.0004091	0.0011965	0.136451	192	3	-3.4777	0.99959	0.99959	1	21518	0	-3.4777
C5orf38	3	0.0004151	0.0012171	0.13807	193	3	-2.9559	0.99958	0.99958	1	21517	0	-2.9559
PRIM2	3	0.0004195	0.0012331	0.138588	194	3	-3.5986	0.99958	0.99958	1	21516	0	-3.5986
PPP4C	3	0.0004201	0.0012344	0.138588	195	3	-4.4677	0.99958	0.99958	1	21515	0	-4.4677
PACSIN2	3	0.0004222	0.0012427	0.138792	196	3	-3.7153	0.99958	0.99957	1	21514	0	-3.7153
SLC39A7	3	0.0004415	0.0012975	0.14271	198	3	-4.5268	0.99956	0.99956	1	21513	0	-4.5268
PPP2R1A	3	0.0004458	0.0013121	0.142868	199	3	-2.5896	0.92863	0.92872	1	19683	0	-2.5896
CSTF1	3	0.0004459	0.0013121	0.142868	200	3	-4.8403	0.99563	0.99572	1	21288	0	-4.8403
RNGTT	3	0.0004515	0.0013336	0.144385	201	2	-4.4553	0.64772	0.64765	1	13704	0	-4.4553
FNTB	3	0.0004561	0.0013427	0.144385	202	3	-3.0371	0.99954	0.99954	1	21512	0	-3.0371
EIF4G1	3	0.0004614	0.0013569	0.144385	204	3	-4.3093	0.99954	0.99954	1	21510	0	-4.3093
INTS8	3	0.000463	0.0013656	0.144385	205	3	-4.6318	0.91908	0.91911	1	19498	0	-4.6318
KPNB1	3	0.0004633	0.001366	0.144385	206	3	-4.0255	0.99954	0.99953	1	21509	0	-4.0255
CHMP7	3	0.0004659	0.0013756	0.144617	207	3	-3.4057	0.99953	0.99953	1	21508	0	-3.4057
SKP1	3	0.0004676	0.0013816	0.144617	208	3	-3.6032	0.99953	0.99953	1	21507	0	-3.6032
CDC16	3	0.0004784	0.001403	0.146159	209	3	-3.1211	0.99952	0.99952	1	21506	0	-3.1211
DNLZ	3	0.0004865	0.0014231	0.147544	210	3	-4.6466	0.99587	0.99594	1	21299	0	-4.6466
IPO5	3	0.0005077	0.0014789	0.152593	212	3	-3.3088	0.99949	0.99949	1	21505	0	-3.3088
GLE1	3	0.0005167	0.0014995	0.153862	213	3	-3.2275	0.99948	0.99948	1	21504	0	-3.2275
ARPC3	3	0.000519	0.0015054	0.153862	214	2	-4.6401	0.52144	0.52124	1	11058	0	-4.6401
DCTN3	3	0.0005276	0.0015287	0.155511	215	3	-3.1825	0.99947	0.99947	1	21503	0	-3.1825
TOP2A	3	0.0005338	0.0015465	0.15586	217	2	-4.7868	0.71754	0.71736	1	15172	0	-4.7868
DHX33	3	0.0005375	0.0015561	0.156101	218	2	-4.8816	0.794	0.79389	1	16798	0	-4.8816
MYH9	3	0.0005403	0.0015639	0.156157	219	3	-3.3812	0.99946	0.99946	1	21502	0	-3.3812
MGEA5	3	0.0005465	0.0015826	0.157303	220	3	-3.0683	0.99945	0.99946	1	21501	0	-3.0683
CCDC113	3	0.0005595	0.001621	0.159698	221	3	-3.134	0.99944	0.99944	1	21499	0	-3.134
POLR1B	3	0.0005598	0.0016215	0.159698	222	3	-3.1043	0.99944	0.99944	1	21498	0	-3.1043
ABCB7	3	0.0005675	0.0016448	0.160534	223	3	-3.191	0.99943	0.99944	1	21496	0	-3.191
OSGEP	3	0.0005714	0.0016562	0.160924	224	3	-3.372	0.99943	0.99943	1	21495	0	-3.372
NFYB	3	0.0005865	0.0017014	0.164582	225	2	-3.7319	0.7517	0.75165	1	15901	0	-3.7319
C22orf28	3	0.0005897	0.0017092	0.164598	226	2	-4.6533	0.83978	0.83973	1	17784	0	-4.6533
TBC1D3F	2	0.0006059	0.0011769	0.136451	227	2	-4.0749	0.99939	0.9994	1	21494	0	-4.0749
RPL21	3	0.000606	0.0017485	0.166992	228	3	-3.8342	0.99939	0.9994	1	21493	0	-3.8342
SPDL1	3	0.0006068	0.0017503	0.166992	229	3	-3.015	0.99319	0.9933	1	21205	0	-3.015
CDC45	3	0.0006094	0.0017572	0.166992	230	3	-4.7434	0.99866	0.99868	1	21433	0	-4.7434
GAPDH	3	0.0006148	0.0017809	0.168511	231	2	-4.67	0.091989	0.091642	1	1928	0	-4.67
RPL36	3	0.0006214	0.0018024	0.169802	232	2	-4.8301	0.81967	0.81952	1	17347	0	-4.8301
NOC4L	3	0.0006291	0.001823	0.170411	233	3	-4.5623	0.99937	0.99938	1	21492	0	-4.5623
RPS11	3	0.0006322	0.0018307	0.170411	234	3	-4.7419	0.9912	0.99126	1	21166	0	-4.7419
RPS18	3	0.0006347	0.0018353	0.170411	235	3	-3.2018	0.99937	0.99937	1	21491	0	-3.2018

SHC1	3	0.0006357	0.0018403	0.170411	236	3	-3.7046	0.99936	0.99937	1	21490	0	-3.7046
AGAP4	3	0.0006423	0.001855	0.171034	237	3	-2.5734	0.99936	0.99936	1	21488	0	-2.5734
SNRNP35	3	0.0006746	0.00195	0.17828	238	3	-3.3476	0.99933	0.99933	1	21487	0	-3.3476
LSM11	3	0.0006866	0.0019783	0.179882	239	3	-3.5919	0.99931	0.99931	1	21486	0	-3.5919
CHORDC1	3	0.0006921	0.0019943	0.179882	240	3	-4.0204	0.99931	0.99931	1	21485	0	-4.0204
ATP6V1H	3	0.0006962	0.0020007	0.179882	241	3	-2.8968	0.9993	0.99931	1	21484	0	-2.8968
SFSWAP	3	0.0006984	0.0020112	0.180079	242	3	-3.1061	0.9993	0.99931	1	21483	0	-3.1061
PPIL2	3	0.0007186	0.0020683	0.183676	243	2	-4.463	0.74152	0.74142	1	15700	0	-4.463
RHEB	3	0.0007379	0.0021232	0.187775	245	3	-3.1525	0.99926	0.99927	1	21482	0	-3.1525
TMEM27	3	0.0007476	0.0021515	0.189101	246	2	-4.0184	0.32009	0.31989	1	6818	0	-4.0184
RPL27	3	0.0007494	0.0021556	0.189101	247	3	-3.3913	0.99925	0.99925	1	21481	0	-3.3913
CASC5	3	0.0007564	0.0021766	0.189451	249	3	-4.3409	0.93958	0.93972	1	19910	0	-4.3409
PHF5A	3	0.0007564	0.0021771	0.189451	250	3	-3.9909	0.99924	0.99925	1	21480	0	-3.9909
POLR3E	3	0.0007623	0.0021963	0.190032	251	3	-2.5028	0.99793	0.99795	1	21400	0	-2.5028
SMNDC1	3	0.0007653	0.0022013	0.190032	252	2	-4.6205	0.48588	0.48568	1	10308	0	-4.6205
NAPA	3	0.0007832	0.0022516	0.1936	253	3	-4.5228	0.99877	0.99878	1	21449	0	-4.5228
ALG13	3	0.0007922	0.0022767	0.193917	254	2	-4.1279	0.85658	0.85652	1	18128	0	-4.1279
UTP15	3	0.0007949	0.0022826	0.193917	255	3	-3.2279	0.99921	0.99921	1	21478	0	-3.2279
TTC27	3	0.0007973	0.002289	0.193917	256	3	-3.3022	0.9992	0.9992	1	21477	0	-3.3022
STT3B	3	0.0008013	0.0022996	0.193917	257	3	-3.5417	0.9992	0.9992	1	21476	0	-3.5417
GSG2	3	0.0008017	0.0023	0.193917	258	3	-3.2979	0.9992	0.9992	1	21475	0	-3.2979
UTP14A	3	0.0008089	0.0023201	0.194854	259	3	-4.5786	0.99701	0.99703	1	21355	0	-4.5786
TRA2B	3	0.0008209	0.0023535	0.194864	261	3	-3.9956	0.99918	0.99918	1	21474	0	-3.9956
FRMD5	3	0.0008211	0.0023535	0.194864	262	2	-1.9387	0.44895	0.44877	1	9543	0	-1.9387
RNPS1	3	0.0008211	0.0023535	0.194864	263	3	-4.559	0.98886	0.98899	1	21103	0	-4.559
CRKL	3	0.0008225	0.0023562	0.194864	264	3	-3.4203	0.99918	0.99918	1	21473	0	-3.4203
ZNHIT6	3	0.000835	0.0023896	0.196872	265	3	-4.5012	0.99845	0.99846	1	21420	0	-4.5012
SRPR	3	0.0008475	0.0024234	0.198524	267	3	-3.3894	0.99915	0.99915	1	21472	0	-3.3894
PRIM1	3	0.000849	0.002428	0.198524	268	3	-4.0482	0.98599	0.98611	1	21024	0	-4.0482
TLN1	3	0.0008631	0.0024686	0.199138	269	3	-4.357	0.99872	0.99873	1	21443	0	-4.357
SMC5	3	0.0008647	0.0024732	0.199138	271	3	-4.1685	0.99003	0.99015	1	21130	0	-4.1685
HSPA9	3	0.0008678	0.0024814	0.199138	272	2	-4.4357	0.30832	0.30807	1	6569	0	-4.4357
SSRP1	3	0.0008679	0.0024814	0.199138	273	3	-3.8786	0.99913	0.99913	1	21471	0	-3.8786
ATXN7L2	3	0.000868	0.0024814	0.199138	274	2	-1.9245	0.4119	0.41189	1	8775	0	-1.9245
YBX1	3	0.000873	0.002497	0.199646	275	3	-3.5406	0.99913	0.99912	1	21470	0	-3.5406
MED9	3	0.0008821	0.0025216	0.200877	276	3	-4.5405	0.99655	0.9966	1	21327	0	-4.5405
NUP88	3	0.0008869	0.0025344	0.201037	277	3	-4.5771	0.8807	0.88075	1	18657	0	-4.5771
SPATA5	3	0.0009055	0.0025851	0.203455	278	3	-2.9817	0.99909	0.99909	1	21469	0	-2.9817
XRCC6	3	0.0009091	0.0025915	0.203455	280	3	-3.2378	0.99909	0.99909	1	21468	0	-3.2378
ALDH3B1	3	0.0009149	0.0026158	0.204306	281	2	-2.3512	0.42372	0.42362	1	9021	0	-2.3512
SRSF10	3	0.0009179	0.0026212	0.204306	282	3	-3.6409	0.99908	0.99909	1	21467	0	-3.6409
POLR3K	3	0.0009223	0.0026345	0.204603	283	3	-4.3261	0.99908	0.99908	1	21466	0	-4.3261

SAP18	3	0.0009321	0.0026624	0.206029	284	2	-4.3509	0.74266	0.74258	1	15720	0	-4.3509
THOC3	3	0.0009354	0.0026724	0.206071	285	2	-3.4464	0.1355	0.13502	1	2863	0	-3.4464
DNAJB11	3	0.0009561	0.00273	0.209228	286	3	-3.1926	0.99904	0.99905	1	21465	0	-3.1926
MRPL41	3	0.0009618	0.0027423	0.209228	287	3	-2.6192	0.98218	0.98229	1	20930	0	-2.6192
NDUFA4L2	3	0.0009693	0.0027615	0.209445	288	3	-2.8097	0.99903	0.99904	1	21464	0	-2.8097
C12orf45	3	0.0009717	0.0027661	0.209445	290	3	-4.4433	0.89076	0.89079	1	18886	0	-4.4433
TTI1	3	0.0009801	0.0027889	0.209445	291	3	-4.5769	0.9913	0.99137	1	21167	0	-4.5769
DHX9	3	0.0009827	0.0027935	0.209445	292	3	-3.8493	0.99902	0.99902	1	21463	0	-3.8493
ASH2L	3	0.0009986	0.0028424	0.212376	293	2	-4.0956	0.58767	0.58751	1	12462	0	-4.0956
ACTR3	3	0.0010047	0.0028579	0.212803	294	3	-4.0283	0.999	0.999	1	21462	0	-4.0283
C21orf59	3	0.0010259	0.0029269	0.216166	296	2	-4.4638	0.86341	0.86335	1	18278	0	-4.4638
RPP21	3	0.0010327	0.0029438	0.216166	298	2	-4.2452	0.79881	0.79866	1	16900	0	-4.2452
RPL37	3	0.0010337	0.0029452	0.216166	299	3	-3.5205	0.99897	0.99897	1	21460	0	-3.5205
C15orf41	3	0.0010362	0.0029525	0.216166	300	2	-3.6753	0.86834	0.86837	1	18377	0	-3.6753
ARFRP1	3	0.0010366	0.002953	0.216166	301	3	-3.5482	0.99896	0.99897	1	21459	0	-3.5482
PAK2	3	0.0010472	0.0029891	0.217937	302	3	-3.4038	0.99895	0.99896	1	21458	0	-3.4038
RBM18	3	0.0010556	0.0030101	0.217937	303	3	-2.9179	0.93922	0.93937	1	19900	0	-2.9179
JAK1	3	0.0010564	0.0030115	0.217937	304	3	-2.9235	0.99894	0.99895	1	21457	0	-2.9235
MED8	3	0.0010657	0.0030352	0.218496	305	3	-4.5713	0.89346	0.89351	1	18950	0	-4.5713
NMD3	3	0.0010692	0.0030476	0.218658	306	2	-4.4386	0.14111	0.14063	1	2973	0	-4.4386
SNRPC	2	0.0010694	0.0020629	0.183676	307	2	-4.1328	0.99893	0.99893	1	21456	0	-4.1328
SUPT5H	3	0.0010938	0.0031152	0.222626	308	3	-3.2034	0.99891	0.99891	1	21455	0	-3.2034
CHMP2A	3	0.0011144	0.0031632	0.22472	309	3	-3.0008	0.99889	0.99889	1	21454	0	-3.0008
DYNC1H1	3	0.0011205	0.0031819	0.225312	310	2	-4.229	0.34207	0.34179	1	7286	0	-4.229
WDR74	3	0.0011403	0.0032404	0.227514	312	3	-4.182	0.94319	0.94334	1	19992	0	-4.182
ELP6	3	0.0011421	0.0032445	0.227514	313	2	-4.0444	0.52453	0.52429	1	11127	0	-4.0444
MSTO1	3	0.0011803	0.0033528	0.233597	315	2	-4.4736	0.61883	0.61881	1	13100	0	-4.4736
SSB	3	0.0011881	0.0033725	0.234045	317	3	-3.8519	0.99881	0.99882	1	21453	0	-3.8519
DPY30	3	0.0011932	0.0033843	0.234045	318	3	-4.0648	0.99579	0.99587	1	21296	0	-4.0648
EIF4A3	3	0.0011962	0.0033916	0.234045	319	3	-2.2801	0.93689	0.93701	1	19851	0	-2.2801
COG3	3	0.0012099	0.0034314	0.23556	320	3	-4.4761	0.95653	0.95673	1	20321	0	-4.4761
EIF2S3	3	0.0012157	0.0034437	0.23556	321	3	-3.4626	0.99878	0.9988	1	21452	0	-3.4626
MRPS30	3	0.0012205	0.0034579	0.23556	322	3	-3.9098	0.99878	0.99879	1	21451	0	-3.9098
POP5	3	0.0012211	0.0034611	0.23556	323	3	-4.4749	0.98831	0.98846	1	21091	0	-4.4749
GMPPB	3	0.0012248	0.003468	0.23556	324	3	-3.2096	0.99878	0.99879	1	21450	0	-3.2096
POLR1A	3	0.0012361	0.0035004	0.235833	325	2	-3.6482	0.86347	0.86341	1	18279	0	-3.6482
HSPA14	3	0.0012431	0.0035182	0.235833	326	3	-2.4554	0.96138	0.96154	1	20442	0	-2.4554
SBDS	3	0.0012455	0.0035237	0.235833	328	3	-4.3004	0.97581	0.9759	1	20774	0	-4.3004
RPL6	3	0.0012557	0.0035516	0.235833	330	3	-3.0898	0.99874	0.99876	1	21446	0	-3.0898
FDXR	3	0.0012587	0.0035593	0.235833	331	2	-4.1861	0.50688	0.50667	1	10742	0	-4.1861
RIOK1	3	0.0012663	0.003579	0.235833	332	3	-4.412	0.9966	0.99667	1	21329	0	-4.412
POLR1C	3	0.0012672	0.0035799	0.235833	333	3	-3.6163	0.99873	0.99874	1	21445	0	-3.6163

CFLAR	3	0.0012739	0.0035996	0.235833	334	2	-4.3346	0.64947	0.6494	1	13741	0	-4.3346
SSU72	3	0.0012765	0.0036078	0.235833	335	3	-2.8111	0.99872	0.99874	1	21444	0	-2.8111
NUS1	3	0.0012835	0.0036256	0.235833	336	3	-4.4579	0.99	0.99012	1	21128	0	-4.4579
C3orf38	3	0.0012854	0.0036343	0.235833	337	3	-3.719	0.99871	0.99872	1	21442	0	-3.719
MIOS	3	0.0012873	0.0036393	0.235833	338	3	-4.2924	0.96253	0.96265	1	20468	0	-4.2924
SUPV3L1	3	0.0012909	0.0036512	0.235833	339	3	-2.7634	0.99871	0.99872	1	21441	0	-2.7634
SBNO1	3	0.001296	0.0036626	0.235833	340	3	-3.4729	0.9987	0.99871	1	21440	0	-3.4729
ACTL6A	3	0.0013027	0.0036768	0.235833	343	3	-4.0178	0.94972	0.94988	1	20151	0	-4.0178
CHMP6	3	0.0013032	0.0036795	0.235833	344	3	-3.7182	0.9987	0.99871	1	21438	0	-3.7182
TOP3A	3	0.0013085	0.0036928	0.235833	345	3	-2.7453	0.99171	0.9918	1	21176	0	-2.7453
GRB2	3	0.00131	0.0036978	0.235833	346	3	-2.9122	0.99869	0.9987	1	21437	0	-2.9122
NAALADL1	3	0.0013111	0.0037056	0.235833	347	3	-3.207	0.99869	0.9987	1	21436	0	-3.207
CENPE	3	0.001324	0.0037451	0.235833	349	2	-4.3467	0.74916	0.74913	1	15854	0	-4.3467
B3GNT5	3	0.0013269	0.0037503	0.235833	350	3	-3.0691	0.99867	0.99869	1	21434	0	-3.0691
CLN5	3	0.0013369	0.0037805	0.235833	351	1	-1.3366	0.38315	0.38311	1	8156	0	-1.3366
RPL39	3	0.0013435	0.0037942	0.235833	352	2	-4.4515	0.8679	0.8679	1	18369	0	-4.4515
PELO	3	0.0013474	0.0038052	0.235833	354	3	-3.0132	0.99865	0.99867	1	21432	0	-3.0132
NBPF15	3	0.0013494	0.0038107	0.235833	355	3	-4.2577	0.99185	0.99193	1	21183	0	-4.2577
SRP14	3	0.001352	0.0038175	0.235833	356	3	-4.3575	0.99865	0.99866	1	21431	0	-4.3575
DAD1	3	0.0013612	0.0038408	0.236428	358	3	-4.083	0.9706	0.97067	1	20640	0	-4.083
FDX1L	3	0.001377	0.0038783	0.237805	359	3	-3.9805	0.98786	0.98801	1	21076	0	-3.9805
RAB25	3	0.0013838	0.0038956	0.237805	360	2	-2.9887	0.70155	0.70135	1	14846	0	-2.9887
FNTA	3	0.0013867	0.0039062	0.237805	361	3	-3.2607	0.99861	0.99863	1	21430	0	-3.2607
TPI1	3	0.0013873	0.0039071	0.237805	362	3	-2.8506	0.99861	0.99863	1	21429	0	-2.8506
BRIX1	3	0.0013978	0.0039336	0.238162	363	3	-3.1219	0.9986	0.99861	1	21428	0	-3.1219
TRIO	3	0.0013996	0.0039377	0.238162	364	3	-3.1024	0.9986	0.99861	1	21427	0	-3.1024
POLR1E	3	0.0014029	0.0039459	0.238162	365	3	-3.9279	0.99692	0.99694	1	21349	0	-3.9279
LENG8	3	0.0014238	0.004003	0.24016	366	3	-3.1486	0.99858	0.99859	1	21426	0	-3.1486
NUBP2	3	0.001425	0.0040062	0.24016	367	3	-3.9783	0.9902	0.99031	1	21134	0	-3.9783
UBXN1	3	0.0014307	0.0040254	0.24016	368	2	-2.393	0.86962	0.86968	1	18410	0	-2.393
GLIS1	3	0.0014327	0.0040286	0.24016	369	3	-3.1438	0.99857	0.99858	1	21425	0	-3.1438
HAUS7	3	0.0014371	0.0040391	0.24016	370	2	-4.1724	0.66686	0.66653	1	14112	0	-4.1724
RPS19	3	0.0014391	0.0040455	0.24016	371	3	-4.4174	0.99238	0.99246	1	21192	0	-4.4174
CHIC2	3	0.0014483	0.0040752	0.241262	372	3	-3.1311	0.99855	0.99856	1	21424	0	-3.1311
NUDT12	3	0.0014537	0.0040944	0.241662	373	3	-3.2705	0.99855	0.99856	1	21423	0	-3.2705
RABGGTB	3	0.0014594	0.0041086	0.241662	374	3	-3.1803	0.97448	0.97456	1	20740	0	-3.1803
CRCP	3	0.0014615	0.0041154	0.241662	375	2	-4.2413	0.79742	0.79727	1	16873	0	-4.2413
PSMB4	3	0.0014776	0.0041547	0.241937	376	2	-2.6683	0.79503	0.79489	1	16830	0	-2.6683
HIST2H3A	3	0.0014778	0.0041547	0.241937	377	2	-3.717	0.73665	0.73654	1	15585	0	-3.717
UTP3	3	0.001481	0.004163	0.241937	378	3	-3.4346	0.99852	0.99853	1	21422	0	-3.4346
EXOC1	3	0.0014975	0.0042205	0.24452	379	3	-3.2987	0.9985	0.99852	1	21421	0	-3.2987
RPL35A	3	0.0015129	0.0042644	0.245747	381	2	-4.3673	0.87021	0.87027	1	18422	0	-4.3673

TMEM184A	3	0.0015171	0.0042767	0.245788	382	2	-2.8762	0.79992	0.79974	1	16923	0	-2.8762
FCF1	3	0.0015233	0.0042959	0.245788	383	3	-4.1263	0.99748	0.99748	1	21379	0	-4.1263
MUM1	3	0.0015244	0.0042991	0.245788	384	2	-2.2079	0.68115	0.68083	1	14414	0	-2.2079
RPS25	3	0.0015442	0.0043604	0.24733	385	2	-3.9618	0.55082	0.55067	1	11703	0	-3.9618
UBE2H	3	0.0015547	0.0043869	0.248184	386	2	-4.3762	0.78902	0.78893	1	16699	0	-4.3762
COMMD3	3	0.0015737	0.004444	0.249731	387	2	-3.8434	0.53997	0.53983	1	11466	0	-3.8434
ELP3	3	0.0015758	0.0044488	0.249731	388	3	-4.2386	0.99672	0.99677	1	21338	0	-4.2386
KAAG1	2	0.0018763	0.0036553	0.235833	420	2	-1.6206	0.83628	0.83623	1	17715	0	-1.6206
TMEM48	2	0.0022757	0.0044358	0.249731	470	2	-3.7817	0.99772	0.99769	1	21389	0	-3.7817

Supplementary Table 1

CRISPR KO primers	Up primer	Bottom primer
WDR59 KO gRNA cloning	caccgATATCCGCACATCGCCGTCA	aaacTGACGGCGATGTGCGGATATc
WDR59 KO genomic cleavage assay	GCCAGAACTCATTATCCAGCTCC	CGGTGGCTTACCTGGTGTCA
RICTOR KO gRNA cloning	caccgCCATCTGAATAACTTTACTA	aaacTAGTAAAGTTATTCAGATGGc
RICTOR KO genomic cleavage	ATACACTGGACAAAATCCATGAGAAGCAGG	GGGTACAGGGGAGTAGAGATG
SAV1 KO gRNA cloning	caccgGGAGGTGGTTGATCATACCG	aaacCGGTATGATCAACCACCTCCc
FRMD6 KO gRNA cloning	caccgCTTCCGTGTGCAGTACTATG	aaacCATAGTACTGCACACGGAAGc
**CRISPR Activation primers**	**Up primer**	**Bottom primer**
WDR59 act gRNA cloning	caccgAGGCGCGGTGTAGCAATTGG	aaacCCAATTGCTACACCGCGCCTc
RICTOR act gRNA cloning	caccgCATTTGGACGACGGCTTCCG	aaacCGGAAGCCGTCGTCCAAATGc
SAV1 act gRNA1 cloning	caccgCCTGCCGACTGAGAAGATGA	aaacTCATCTTCTCAGTCGGCAGGc
SAV1 act gRNA2 cloning	caccgCTTCTCGCTGAGGATGAGTG	aaacCACTCATCCTCAGCGAGAAGc
SAV1 act gRNA3 cloning	caccgGACTCGGGTGCCGGCGCTCT	aaacAGAGCGCCGGCACCCGAGTCc
FRMD6 act gRNA1 cloning	caccgGGAGCTGCGCGCTGAGCTCG	AAACcgagctcagcgcgcagctccC
FRMD6 act gRNA2 cloning	caccgGGAGGGGTGCGGCCACTTGG	AAACccaagtggccgcacccctccC
FRMD6 act gRNA3 cloning	caccgGGACCCAACCAAGCGTCCCG	AAACcgggacgcttggttgggtccC
SESN3 gRNA2 cloning	caccgACAACAACCCTGGTTTCCTT	aaacAAGGAAACCAGGGTTGTTGTc
SESN3 gRNA3 cloning	caccgTTCGTAGAATGAAATCTATG	aaacCATAGATTTCATTCTACGAAc
**Primer for gene expression**	**Forward**	**Reverse**
SESN3	AGTGCCGCTAGCTTTCAGG	TTCCGGCAGTTGGTACAGAG
SAV1	GCGGGGAAAGTTTACGGGAT	CAGTCGCTGGTCAGTTCCTT
FRMD6	GAGTCAGAGGGGTGACCAGA	GGCAATTCCTCAGGTTCCCA
WDR59	TGATTCTCTGGCTGTGTGGG	TTCCACTGCACAGCTCCAAT
RICTOR	GAGTACGAGGGCGGAATGAC	CTGCTCGCACTTCTTTTGCT

Supplementary Table 2

Antibody	Cat no	Lot #	Company	Dilution	Source
YAP (D8H1X) XP	14074	4	Cell Signaling	1:4000	Rabbit
Phospho-YAP (Ser127) (D9W2I)	13008	5	Cell Signaling	1:2000	Rabbit
MST1	3682	5	Cell Signaling	1:3000	Rabbit
Phospho-MST1 (Thr183)/MST2 (Thr180) (E7U1D)	49332	1	Cell Signaling	1:1000	Rabbit
Akt (pan) (40D4)	2920	8	Cell Signaling	1:3000	Mouse
Phospho-Akt (Ser473)	9271	13	Cell Signaling	1:4000	Rabbit
S6 Ribosomal Protein (5G10)	2217	5	Cell Signaling	1:8000	Rabbit
Phospho-S6 Ribosomal Protein (Ser240/244)	5364	3	Cell Signaling	1:4000	Rabbit
p70 S6 Kinase (49D7)	2708	8	Cell Signaling	1:2000	Rabbit
Phospho-p70 S6 Kinase (Thr389) (108D2)	9234	7	Cell Signaling	1:3000	Mouse
SAV1	3507	2	Cell Signaling	1:2000	Rabbit
FRMD6 (D8X3R)	14688	1	Cell Signaling	1:1000	Rabbit
SESN3 Polyclonal	PA5-71710	UB2712225A	Invitrogen	1:1000	Rabbit
α-Tubulin (11H10)	2125S	11	Cell Signaling	1:10000	Rabbit
LAMP1 (D401S)	15665	1	Cell Signaling	1:300	Mouse
Phospho-LATS1 (Ser909)	9157	2	Cell Signaling	1:1000	Rabbit
PCNA (D3H8P) XP ®	13110	4	Cell Signaling	1:300	Rabbit

Secondary Antibody	Cat #	Lot #	Company	Dilution
Alexa Flour 568 goat anti-rabbit IgG (H+L)	A11011	2017252	Invitrogen	1:600
Alexa fFlour 468 goat anti-mouse IgG (H+L)	A11001	2051236	Invitrogen	1:600
Anti-mouse IgG, HRP-Linked Antibody	7076	35	Cell Signaling	1:3000
Rabbit IgG (H+L) HRP	AP307P	3185610	EMD Millipore	1:3000

supplementary Table 3

Supplementary Legends

Supplementary Figure 1. Double mutation of PIK3CA and TP53 correlate poor survival outcome. (A) Percentage patients carried either single mutation (mut) of TP53/PIK3CA or both mutations in 320 TNBC patients derived from METABRIC dataset. (B) Number of patients for different sites of PIK3CA mutations in 2,500 breast cancer patients from METABRIC dataset. The red box highlights the H1047 mutation as the top mutated site for breast cancer patients. (C) Kaplan Meier overall survival analysis of PIK3CA mutated and non-mutated TNBC patients. *p*-value was derived from Logrank test. (D) Kaplan Meier overall survival analysis of TP53 mutated and non-mutated TNBC patients. (E) and (F) Kaplan Meier overall survival and individual patient survival month of 320 TNBC patients including 238 patients with single TP53 mutation, 53 patients with single PIK3CA mutation, and 37 patients with double mutation. *p*-value was derived from Logrank test. The box is drawn from the 25th percentile to the 75th percentile, with the horizontal line in between representing the median. Whiskers are drawn independently above and below the box, and extend to the maximum or minimum data values, unless there are outlier values, in which case the whisker will extend to 1.5 interquartile range. Outliers are defined as values that extend beyond 1.5 IQR.

Supplementary Figure 2. Genetic alteration and expression of negative selected hits in breast cancer subtypes and their correlations with clinical outcomes. (A) Gene expressions of 343 negative selected hits in TNBC and non-TNBC from TCGA dataset. (B) Percentage of genomic alteration frequency of 117 TNBC overexpressed hits in with 3,593 breast cancer patients using METABRIC and TCGA pancancer datasets. The colors in the bar graph indicate specific mutation and copy number variance (CNA). (C) Percentage of altered and unaltered groups of 117 TNBC overexpressed hits in all breast cancer subtypes of 3,593 patients using METABRIC and TCGA

pancancer datasets. (D) Association of altered and unaltered groups of 117 TNBC overexpressed hits with indicated gene mutation status. *p*-values by one-sided Fisher Extract test are indicated. (E) Percentage of altered and unaltered groups of 117 TNBC overexpressed hits in all grades (grade 1, 2, 3) of breast cancer. (F) Kaplan Meier overall survival analysis of altered and unaltered groups of 117 TNBC overexpressed hits. *p*-value was derived from Logrank test.

Supplementary Figure 3. Genetic alteration and expression of nine PAM hits in breast cancer subtypes and their correlations with clinical status. (A) Analysis of genomic alteration frequency of nine combined mTOR hits based on positive and negative status of ER, PR, or HER2 expressions in with 1,904 breast cancer patients using METABRIC dataset. The colors in the bar graph indicate copy number variance (CNA). (B) Percentage of altered and unaltered groups of nine mTOR hits in all breast cancer subtypes in METABRIC dataset. NC referred as non-classified. p-value is derived from Chi-Squared Test (C) Percentage of altered and unaltered groups of nine mTOR hits in tumor stages. (D) Percentage of altered and unaltered groups of nine mTOR hits in lymph node positive and negative patients. (E) mRNA expression level of SEH1L, WDR59, RHEB in TNBC and Non-TNBC patients derived from METABRIC dataset. The box is drawn from the 25th percentile to the 75th percentile, with the horizontal line in between representing the median. Whiskers are drawn independently above and below the box, and extend to the maximum or minimum data values, unless there are outlier values, in which case the whisker will extend to 1.5 interquartile range. Outliers are defined as values that extend beyond 1.5 IQR. *p*-values by two-sided unpaired *t* test are indicated.

Supplementary Figure 4. Drug synergy of combination treatment of verteporfin and Torin1. (A) Four TNBC cell lines were treated with combinational drug matrix of verteporfin and Torin1

at the indicated low dose for 3 days. (B) Cell viability was measured by PrestoBlue staining. 2D surface plots of cell viability in four TNBC cell lines were graphed by ZIP model.

Supplementary Figure 5. Torin1 enhances verteporfin apoptotic effect in TNBC. Gating strategy for percentage of apoptotic cells (Annexin V+/PI+) in SUM159 treated with the indicated dose of individual drug or in combination .

Supplementary Figure 6. Gating strategy for flow cytometry analysis of LY in SUM159 cells treated with torin1 (50 nM) and verteporfin (0.6 μM) for 24 hr.

Supplementary Figure 7. Torin1 and verteporfin effect on macropinocytosis in normal primary human mammary epithelial cells. (A) Flow cytometry analysis of LY in HuMEC cells treated with torin1 (50 nM) and verteporfin (0.6 μM) for 24 hr. (B) Fluorescence of LY (green) and LAMP1 (red) in HuMEC cells treated with 50 nM torin1 for 24 hrs. Scale bar is 20 μm.

Supplementary Table 1: In vivo genome-wide CRISPR screen positive and negative gene hits. Log2 (Fold change) of top ranked genes in both positive and negative profiles (false discovery rate (FDR) <0.25) in tumor samples normalized by transduced cell replicates. p-value was derived from a permutation test and FDR was computed from the empirical permutation p-values using Benjamini-Hochberg procedure.

Supplementary Table 2: Primer sequences for CRISPR KO and activation.

Supplementary Table 3: Antibody list and resources
