# Supplementary material for: In vivo genome-wide CRISPR screen reveals breast cancer vulnerabilities and synergistic mTOR/Hippo targeted combination therapy
Source: Nat Commun. 2021 May 24;12:3055. doi: 10.1038/s41467-021-23316-4 (PMC8144221; doi:10.1038/s41467-021-23316-4)
Supplement: Supplementary file 2 — Reporting Summary [file 41467_2021_23316_MOESM2_ESM.pdf]

nature research

Corresponding author(s): Lebrun Jean-Jacques

Last updated by author(s): Apr 16, 2021

Reporting Summary

Nature Research wishes to improve the reproducibility of the work that we publish. This form provides structure for consistency and transparency in reporting. For further information on Nature Research policies, see our Editorial Policies and the Editorial Policy Checklist.

Statistics

For all statistical analyses, confirm that the following items are present in the figure legend, table legend, main text, or Methods section.

n/a	Confirmed	
☐	☒	The exact sample size (*n*) for each experimental group/condition, given as a discrete number and unit of measurement
☐	☒	A statement on whether measurements were taken from distinct samples or whether the same sample was measured repeatedly
☐	☒	The statistical test(s) used AND whether they are one- or two-sided<br>*Only common tests should be described solely by name; describe more complex techniques in the Methods section.*
☒	☐	A description of all covariates tested
☒	☐	A description of any assumptions or corrections, such as tests of normality and adjustment for multiple comparisons
☐	☒	A full description of the statistical parameters including central tendency (e.g. means) or other basic estimates (e.g. regression coefficient) AND variation (e.g. standard deviation) or associated estimates of uncertainty (e.g. confidence intervals)
☐	☒	For null hypothesis testing, the test statistic (e.g. *F*, *t*, *r*) with confidence intervals, effect sizes, degrees of freedom and *P* value noted<br>*Give P values as exact values whenever suitable.*
☒	☐	For Bayesian analysis, information on the choice of priors and Markov chain Monte Carlo settings
☒	☐	For hierarchical and complex designs, identification of the appropriate level for tests and full reporting of outcomes
☐	☒	Estimates of effect sizes (e.g. Cohen's *d*, Pearson's *r*), indicating how they were calculated

Our web collection on statistics for biologists contains articles on many of the points above.

Software and code

Policy information about availability of computer code

Data collection	Essentiality gene sets were derived from large-scale in vitro screening, available in the Achilles dataset 20Q1 from the DepMap portal.
Data analysis	MAGeCK-VISPR 0.5.3 was used for analysis of genome-wide CRISPR screening samples; Non-biological experimental variation (batch effect) was adjusted using ComBat; Individual screen hits were mapped using PathwayMapper; FlowJo V10 was used for flow cytometry analysis; Synergy score of combinational drug was calculated by SynergyFinder; Gene alteration and expression as well as their survival outcomes from breast cancer patients were analyzed by UCSC Xena and cBioPortal; EnrichR was used for Pathway enrichment analysis; ToupView was used for acquiring PCNA staining in tissue samples. ImageJ plugin ImmunoRatio was used for IHC quantification.

For manuscripts utilizing custom algorithms or software that are central to the research but not yet described in published literature, software must be made available to editors and reviewers. We strongly encourage code deposition in a community repository (e.g. GitHub). See the Nature Research guidelines for submitting code & software for further information.

Data

Policy information about availability of data

All manuscripts must include a data availability statement. This statement should provide the following information, where applicable:

- Accession codes, unique identifiers, or web links for publicly available datasets
- A list of figures that have associated raw data
- A description of any restrictions on data availability

DATA AVAILABILITY
Source data are provided with this paper. METABRIC and TCGA pancancer DATASETs is available with the accessible links (https://www.cbioportal.org/study/summary?id=brca_metabric;https://www.cbioportal.org/study/summary?id=brca_tcga_pan_can_atlas_2018; https://xenabrowser.net/heatmap)

Field-specific reporting

Please select the one below that is the best fit for your research. If you are not sure, read the appropriate sections before making your selection.

- [x] Life sciences
- [ ] Behavioural & social sciences
- [ ] Ecological, evolutionary & environmental sciences

For a reference copy of the document with all sections, see nature.com/documents/nr-reporting-summary-flat.pdf

Life sciences study design

All studies must disclose on these points even when the disclosure is negative.

Sample size	No sample-size calculation was performed. For in vitro experiments, a minimum of three biological replicates were used for statistical analysis based on our previous publication when using cell lines. For in vivo experiments, we choose 5-8 mice per groups. This was based on pilot studies which revealed this number of animals to be sufficient to observe significant differences between groups.
Data exclusions	No data were excluded.
Replication	At least three biological replicates were performed with successful outcomes.
Randomization	For all experiments, samples were randomly allocated into experimental groups. Except for drug treatment, Mice were separated into control and treatment groups based on similar median tumor size.
Blinding	While investigators were not blinded to group allocation during data collection and analysis, all tumor volume measurements were performed in a blinded manner all across the different animal groups.

Reporting for specific materials, systems and methods

We require information from authors about some types of materials, experimental systems and methods used in many studies. Here, indicate whether each material, system or method listed is relevant to your study. If you are not sure if a list item applies to your research, read the appropriate section before selecting a response.

Materials & experimental systems

n/a	Involved in the study	
[ ]	[x]	Antibodies
[ ]	[x]	Eukaryotic cell lines
[x]	[ ]	Palaeontology and archaeology
[ ]	[x]	Animals and other organisms
[x]	[ ]	Human research participants
[x]	[ ]	Clinical data
[x]	[ ]	Dual use research of concern

Methods

n/a	Involved in the study	
[x]	[ ]	ChIP-seq
[ ]	[x]	Flow cytometry
[x]	[ ]	MRI-based neuroimaging

Antibodies

Antibodies used	rabbit anti-YAP (D8H1X) XP (Cat# 14074; lot#4) Cell Signaling; rabbit anti-Phospho-YAP (Ser127) (D9W2I) (Cat# 13008; lot#5) Cell Signaling; mouse anti-Akt (pan) (40D4) (Cat# 2920; lot#8) Cell Signaling; rabbit anti-Phospho-Akt (Ser473) (Cat# 9271; lot#13) Cell Signaling, rabbit anti-S6 Ribosomal Protein (5G10) (Cat# 2217; lot#5) Cell Signaling; rabbit anti-Phospho-S6 Ribosomal Protein (Ser240/244) (Cat# 5364; lot#3) Cell Signaling; rabbit anti-p70 S6 Kinase (49D7) (Cat# 2708; lot#8) Cell Signaling; mouse anti-Phospho-p70 S6 Kinase (Thr389) (108D2) (Cat# 9234; lot#7) Cell Signaling; rabbit anti-SESN3 Polyclonal (Cat# PA5-71710; lot#UB2712225A) Invitrogen; rabbit anti-α-Tubulin (11H10) (Cat# 2125S; lot#11) Cell Signaling; mouse anti-LAMP1 (Cat# 15665T; lot#1) Cell Signaling; rabbit anti-MST1 (Cat# 3682; lot#5) Cell Signaling; rabbit anti-Phospho-MST1(Thr183, E7U1D)/MST2(Thr180) (Cat# 49332; lot#1) Cell signaling; rabbit anti-Phospho-LATS1 (Ser909) (Cat# 9157; lot#2) Cell Signaling; rabbit anti-SAV1 (Cat# 3507; lot#2) Cell Signaling; rabbit anti-FRMD6 (D8X3R) (Cat# 14688; lot#1) Cell Signaling; rabbit anti-PCNA (Cat# 13110; lot#4) Cell Signaling;<br>Alexa Flour 568 goat anti-rabbit IgG (H+L) (cat#A11011, lot# 2017252) Invitrogen; Alexa fFlour 468 goat anti-mouse IgG (H+L) (cat# A11001; lot# 2051236) Invitrogen; Anti-mouse IgG, HRP-Linked Antibody (cat#7076; lot# 35) Cell Signaling;<br>anti-Rabbit IgG (H+L) HRP (cat#AP307P; lot#3185610) EMD Millipore
Validation	All antibodies were validated in this study using knockout or activation cells and for their associated applications by the Suppliers and validated by literature citations available on the company website.<br>https://www.cellsignal.com//products/primary-antibodies/yap-d8h1x-xp-rabbit-mab/14074<br>https://www.cellsignal.com//products/primary-antibodies/phospho-yap-ser127-d9w2i-rabbit-mab/13008<br>https://www.cellsignal.com//products/primary-antibodies/akt-pan-40d4-mouse-mab/2920

https://www.cellsignal.com//products/primary-antibodies/phospho-akt-ser473-antibody/9271
https://www.cellsignal.com//products/primary-antibodies/s6-ribosomal-protein-5g10-rabbit-mab/2217
https://www.cellsignal.com//products/primary-antibodies/phospho-s6-ribosomal-protein-ser240-244-d68f8-xp-rabbit-mab/5364
https://www.cellsignal.com//products/primary-antibodies/p70-s6-kinase-49d7-rabbit-mab/2708
https://www.cellsignal.com//products/primary-antibodies/phospho-p70-s6-kinase-thr389-108d2-rabbit-mab/9234
https://www.cellsignal.com/products/primary-antibodies/a-tubulin-11h10-rabbit-mab/2125?site-search-type=Products&N=4294956287&Ntt=2125s&fromPage=plp&_requestid=2011598
https://www.cellsignal.com/products/primary-antibodies/lamp1-d4o1s-mouse-mab/15665?site-search-type=Products&N=4294956287&Ntt=15665t&fromPage=plp&_requestid=2011688
https://www.cellsignal.com//products/primary-antibodies/mst1-antibody/3682
https://www.cellsignal.com//products/primary-antibodies/phospho-mst1-thr183-mst2-thr180-e7u1d-rabbit-mab/49332
https://www.cellsignal.com//products/primary-antibodies/phospho-lats1-ser909-antibody/9157
https://www.cellsignal.com//products/primary-antibodies/sav1-antibody/3507
https://www.cellsignal.com//products/primary-antibodies/frmd6-d8x3r-rabbit-mab/14688
https://www.cellsignal.com//products/primary-antibodies/pcna-d3h8p-xp-rabbit-mab/13110

Eukaryotic cell lines

Policy information about cell lines

Cell line source(s): SUM159PT, SUM149PT, SUM1315MO2 cell lines were obtained from Dr. Stephen Ethier. MDA-MB231 was obtained from ATCC. HEK293FT was obtained from Genhunter. HuMEC cell were purchased from ThermoFisher Scientific.

Authentication: SUM cell lines were obtained from orginal source. The authentication of MDA-MB231, HEK293FT, and HuMEC were performed by the suppliers. We didn't independently perform authentication.

Mycoplasma contamination: All the cell lines were tested by PCR kit for mycoplasma by Diagnostic Laboratory from Comparative Medicine and Animal Resources Centre (McGill University). All cell lines are mycoplasma negative.

Commonly misidentified lines (See ICLAC register): None of the cell lines used in this study are included in the ICLAC register.

Animals and other organisms

Policy information about studies involving animals; ARRIVE guidelines recommended for reporting animal research

Laboratory animals: immunodeficient NOD scid gamma (NSG), mice, female, 8-11 weeks old

Wild animals: The study did not involve wild animals.

Field-collected samples: *For laboratory work with field-collected samples, describe all relevant parameters such as housing, maintenance, temperature, photoperiod and end-of-experiment protocol OR state that the study did not involve samples collected from the field.*

Ethics oversight: All mice were housed and handled in accordance to the approved guidelines of the Canadian Council on Animal Care (CCAC) “Guide to the Care and Use of Experimental Animals”. All experiments were performed under the approved McGill University Animal Care protocol (AUP # 7497 to JJL).

Note that full information on the approval of the study protocol must also be provided in the manuscript.

Flow Cytometry

Plots

Confirm that:

- [x] The axis labels state the marker and fluorochrome used (e.g. CD4-FITC).
- [x] The axis scales are clearly visible. Include numbers along axes only for bottom left plot of group (a 'group' is an analysis of identical markers).
- [x] All plots are contour plots with outliers or pseudocolor plots.
- [x] A numerical value for number of cells or percentage (with statistics) is provided.

Methodology

Sample preparation: SUM159 cells were treated with or without Torin1, Verteporfin, and in combination for 3 days and subjected to stain with Annexin V FITC and PI using Annexin V Apoptosis Detection Kit (Santa Cruz) for 15 min at room temperature according to the manufacturer's protocol. Percentage of Annexin V+/PI+ (late apoptosis) was quantified by flow cytometry FACSCanto II.

Instrument: FACSCanto II

Software	FlowJo v10
Cell population abundance	Total 10000 cells per condition were acquired for analysis. Cell debris were excluded from gating and minimum 80% cells were subjected to further analysis.
Gating strategy	The preliminary FSC/SSC gates of the starting cell population exclude debris at the bottom left corner of the plot. Compensation was performed by single staining of mixed cells (50% alive/50% apoptotic). Unstained cells used as negative control for gating.

☒ Tick this box to confirm that a figure exemplifying the gating strategy is provided in the Supplementary Information.
