# Supplementary material for: In vivo genome-wide CRISPR screen reveals breast cancer vulnerabilities and synergistic mTOR/Hippo targeted combination therapy
Source: Nat Commun. 2021 May 24;12:3055. doi: 10.1038/s41467-021-23316-4 (PMC8144221; doi:10.1038/s41467-021-23316-4)
Supplement: Supplementary file 3 — Source Data [file 41467_2021_23316_MOESM3_ESM.zip › Fig 5d and 5e result_bliss.pdf]

Calculation and Visualization of synergy scores for Drug Combinations

Drug combinations:

Drug combination	Synergy score	Most synergistic area score	M
average 159 torin1 - average 159 verteporfin	1.89	9.91	
Replicate1 159 torin1 - Replicate 1 159 verteporfin	3.52	12.99	
Replicate2 159 torin1 - Replicate 2 159 verteporfin	1.08	8.69	
Replicate 3 159 torin1 - Replicate 3 159 verteporfin	0.96	7.98	
average MDAMB231 torin1 - average MDAMB231 verteporfin	3.38	6.79	
Replicate 1 MDAMB231 torin1 - Replicate 1 MDAMB231 verteporfin	1.51	5.15	
Replicate 2 MDAMB231 torin1 - Replicate 2 MDAMB231 verteporfin	2.29	12.20	
Replicate 3 MDAMB231 torin1 - Replicate 3 MDAMB231 verteporfin	5.66	11.98	
average SUM1315 torin1 - average SUM1315 verteporfin	2.90	14.45	
Replicate 1 SUM1315 torin1 - Replicate 1 SUM1315 verteporfin	4.90	16.20	
Replicate 2 SUM1315 torin1 - Replicate 2 SUM1315 verteporfin	6.34	19.71	
Replicate 3 SUM1315 torin1 - Replicate 3 SUM1315 verteporfin	-2.74	9.36	
average SUM149 torin1 - average SUM149 verteporfin	3.88	9.98	
Replicate 1 SUM149 torin1 - Replicate 1 SUM149 verteporfin	5.29	14.85	
Replicate 2 SUM149 torin1 - Replicate 2 SUM149 verteporfin	5.30	9.58	
Replicate 3 SUM149 torin1 - Replicate 3 SUM149 verteporfin	0.60	6.82	

Chosen parameters:

Readout: inhibition ; Baseline correction: Yes ;

average 159 torin1 & average 159 verteporfin

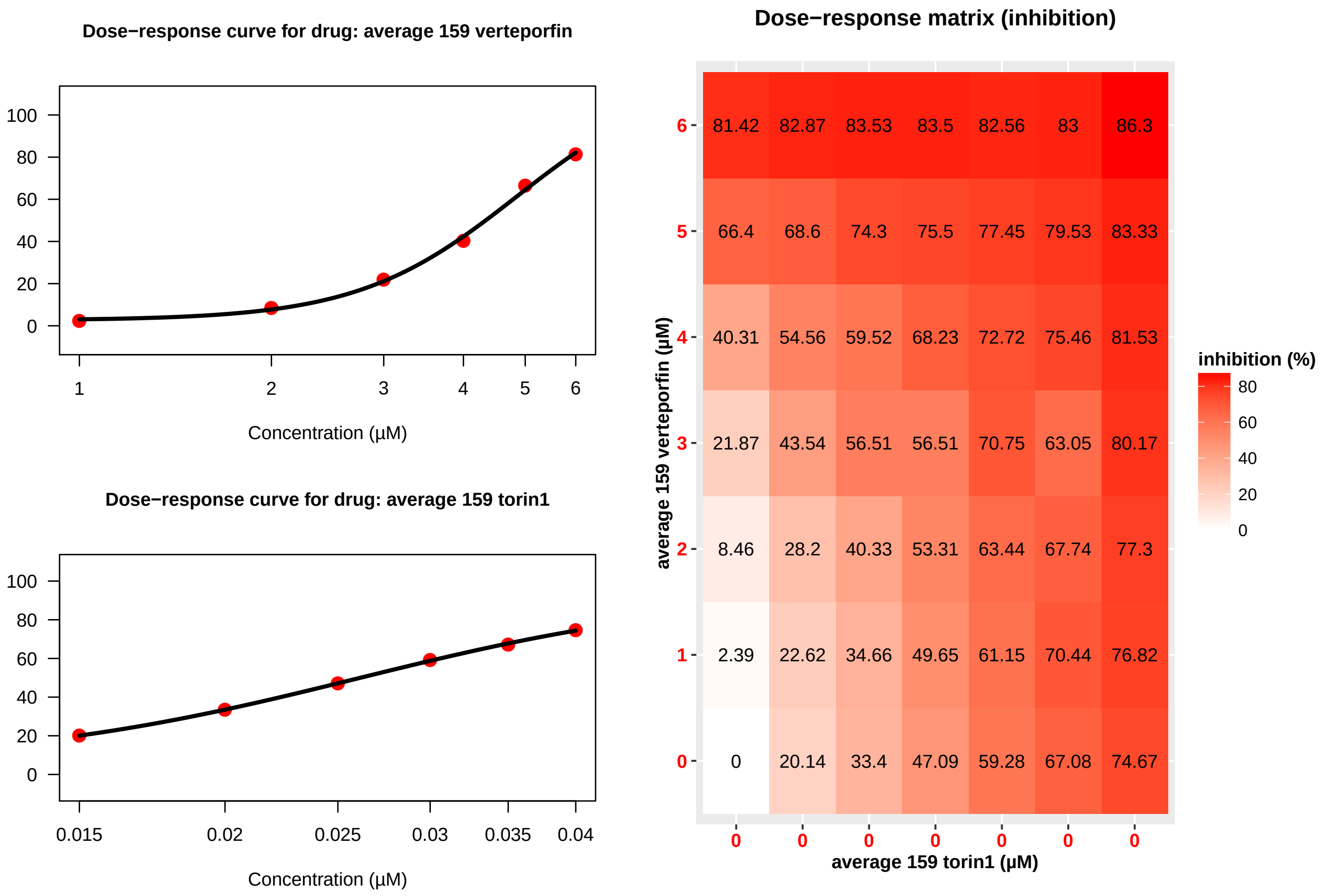

Replicate1 159 torin1 & Replicate 1 159 verteporfin

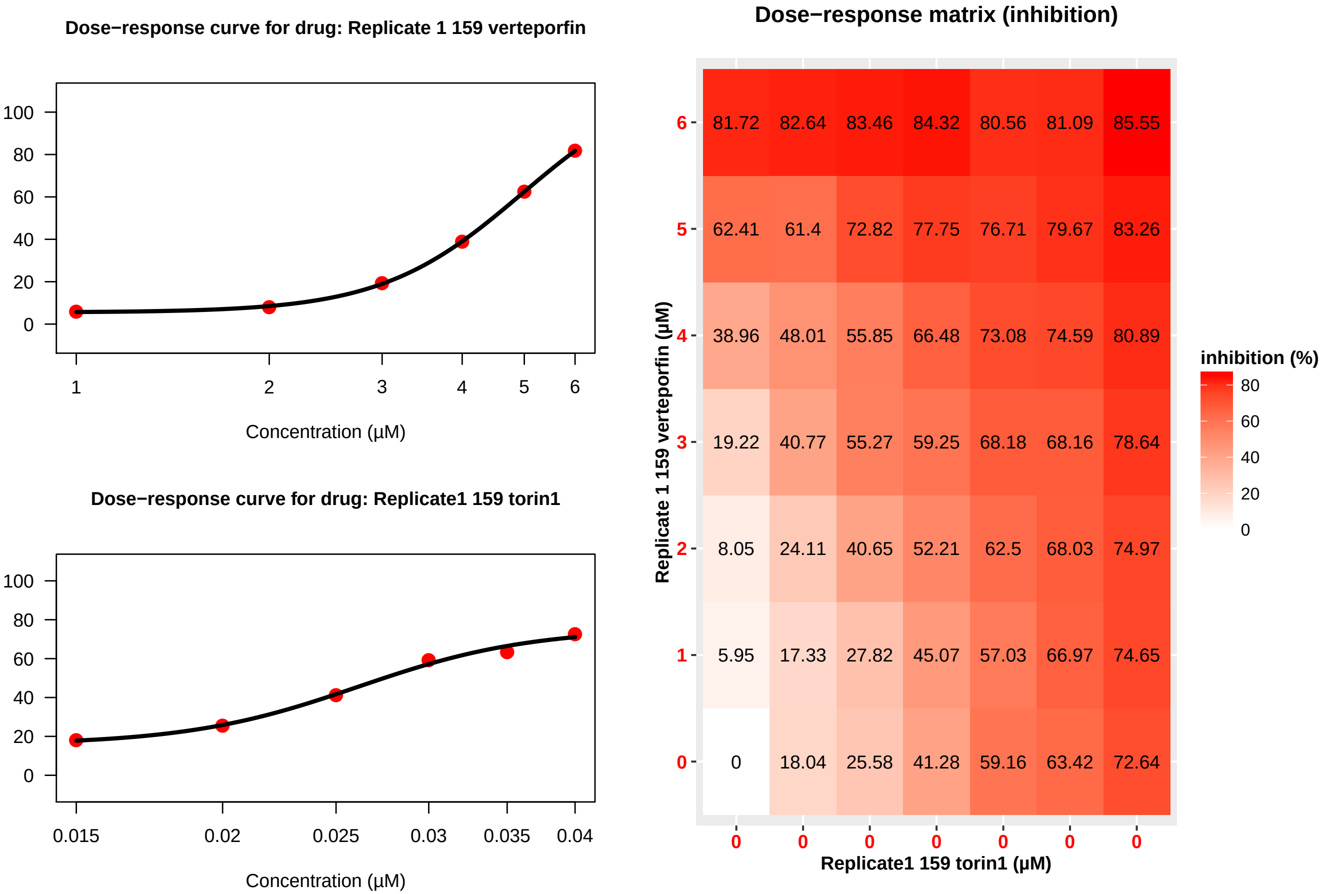

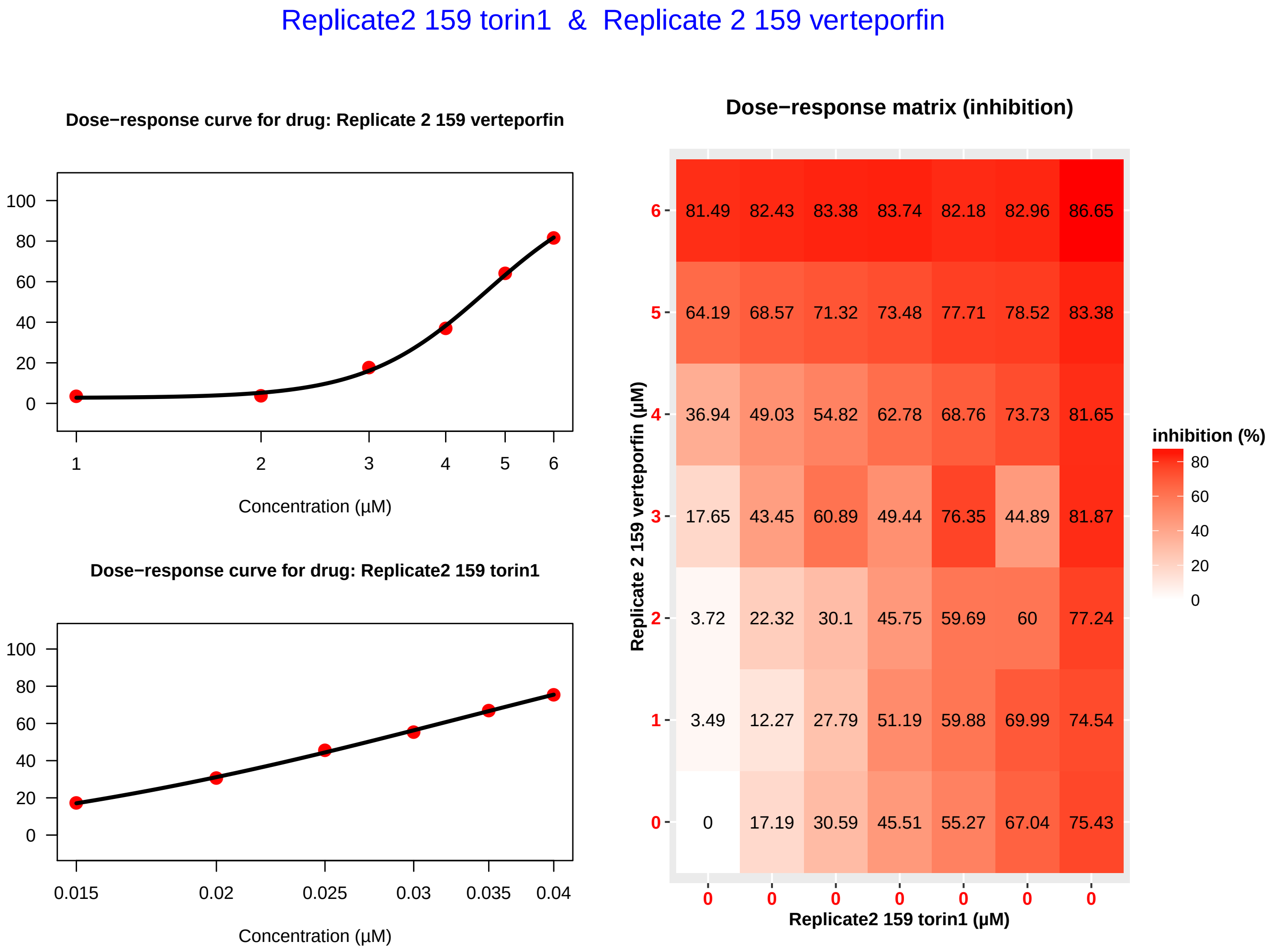

Replicate2 159 torin1 & Replicate 2 159 verteporfin
Dose−response curve for drug: Replicate 2 159 verteporfin
100
80
60
40
20
0
1 2 3 4 5 6
Concentration (µM)
Dose−response curve for drug: Replicate2 159 torin1
100
80
60
40
20
0
0.015 0.02 0.025 0.03 0.035 0.04
Concentration (µM)
Dose−response matrix (inhibition)
Replicate 2 159 verteporfin (µM)
6 81.49 82.43 83.38 83.74 82.18 82.96 86.65
5 64.19 68.57 71.32 73.48 77.71 78.52 83.38
4 36.94 49.03 54.82 62.78 68.76 73.73 81.65
3 17.65 43.45 60.89 49.44 76.35 44.89 81.87
2 3.72 22.32 30.1 45.75 59.69 60 77.24
1 3.49 12.27 27.79 51.19 59.88 69.99 74.54
0 0 17.19 30.59 45.51 55.27 67.04 75.43
0 0 0 0 0 0 0
Replicate2 159 torin1 (µM)
inhibition (%)
80
60
40
20
0

Replicate 3 159 torin1 & Replicate 3 159 verteporfin

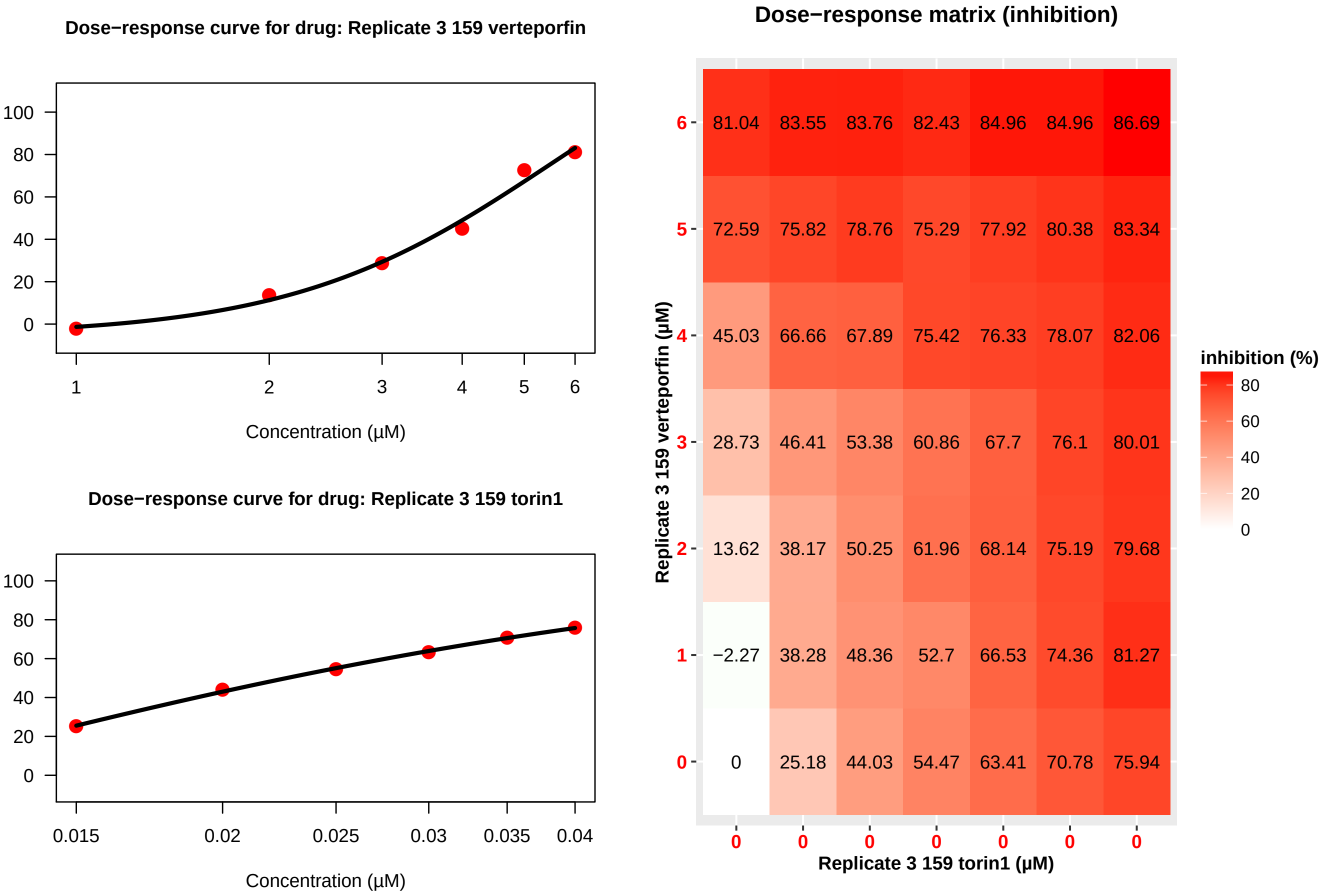

average MDAMB231 torin1 & average MDAMB231 verteporfin

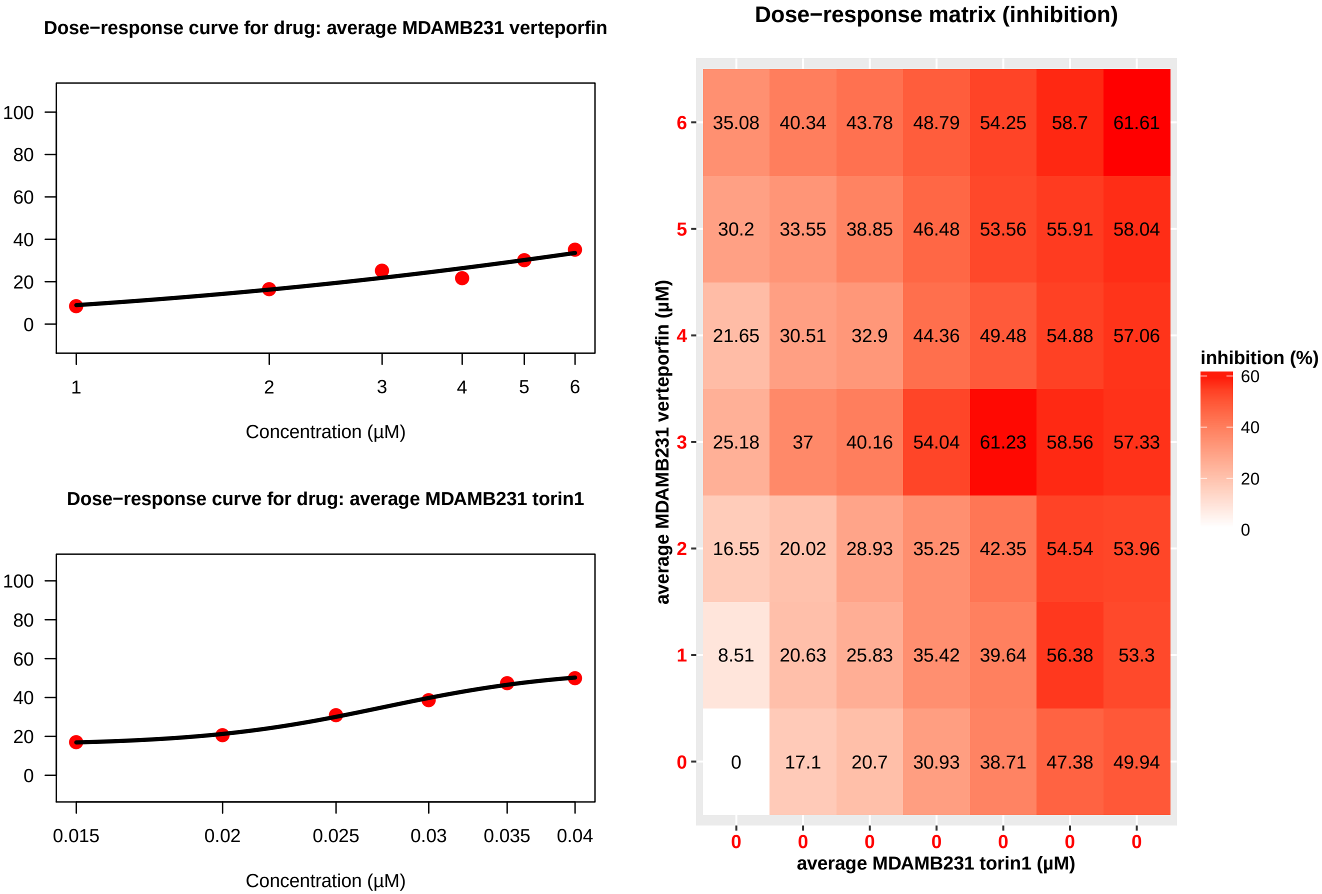

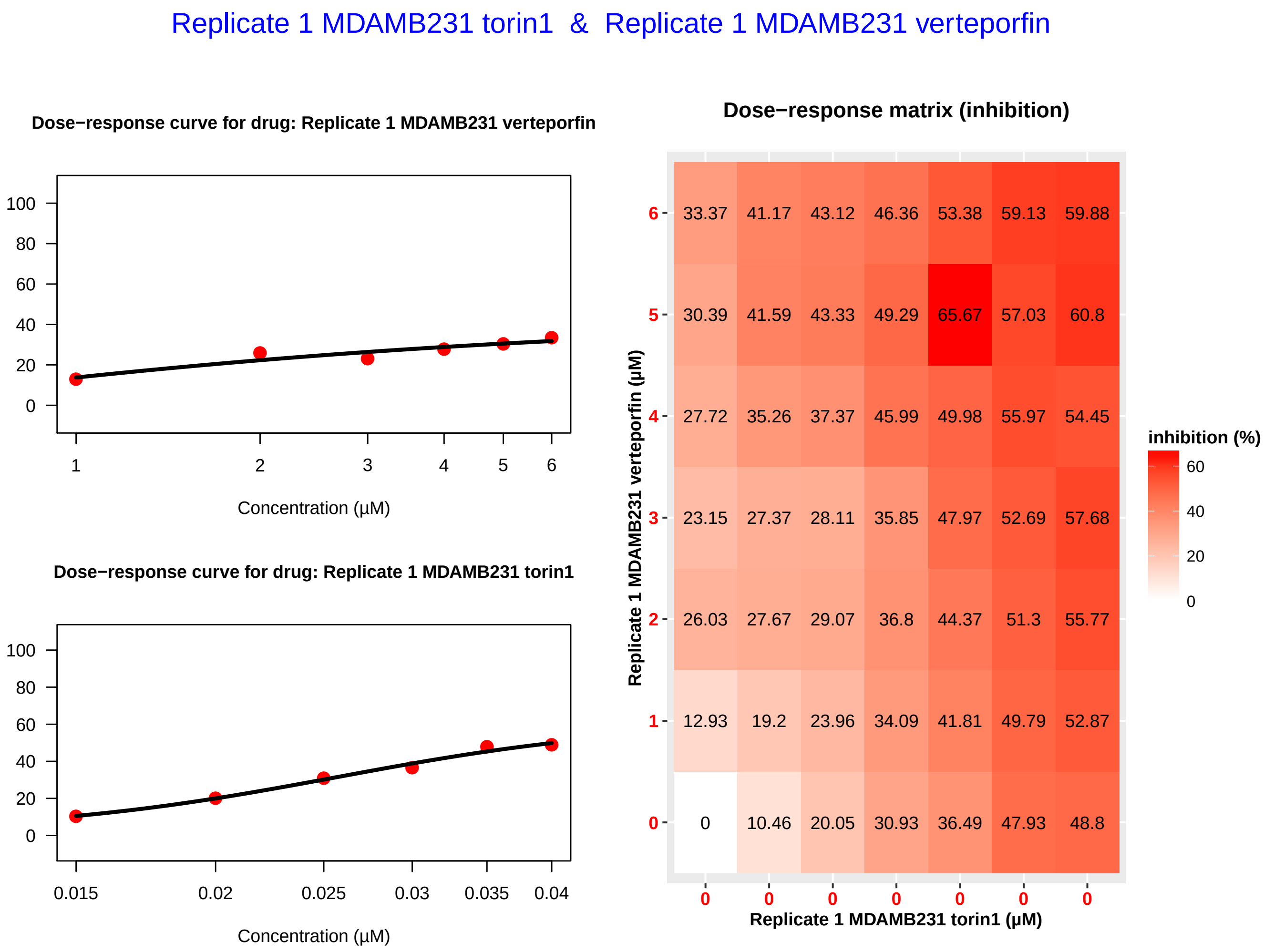
Replicate 1 MDAMB231 torin1 & Replicate 1 MDAMB231 verteporfin
Dose−response curve for drug: Replicate 1 MDAMB231 verteporfin
100
80
60
40
20
0
1
2
3
4
5
6
Concentration (µM)
Dose−response curve for drug: Replicate 1 MDAMB231 torin1
100
80
60
40
20
0
0.015
0.02
0.025
0.03
0.035
0.04
Concentration (µM)
Dose−response matrix (inhibition)
6
33.37 41.17 43.12 46.36 53.38 59.13 59.88
5
30.39 41.59 43.33 49.29 65.67 57.03 60.8
4
27.72 35.26 37.37 45.99 49.98 55.97 54.45
3
23.15 27.37 28.11 35.85 47.97 52.69 57.68
2
26.03 27.67 29.07 36.8 44.37 51.3 55.77
1
12.93 19.2 23.96 34.09 41.81 49.79 52.87
0
0 10.46 20.05 30.93 36.49 47.93 48.8
0 0 0 0 0 0 0
Replicate 1 MDAMB231 torin1 (µM)
Replicate 1 MDAMB231 verteporfin (µM)
inhibition (%)
60
40
20
0

Replicate 2 MDAMB231 torin1 & Replicate 2 MDAMB231 verteporfin

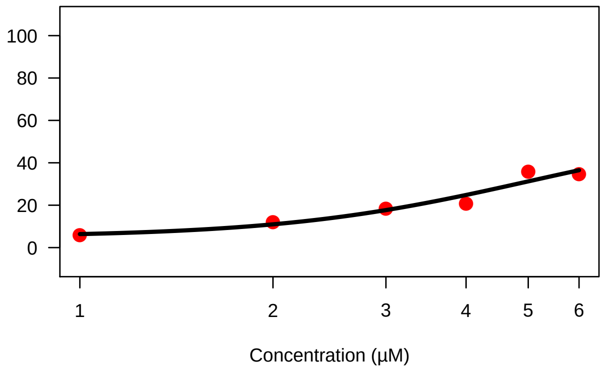

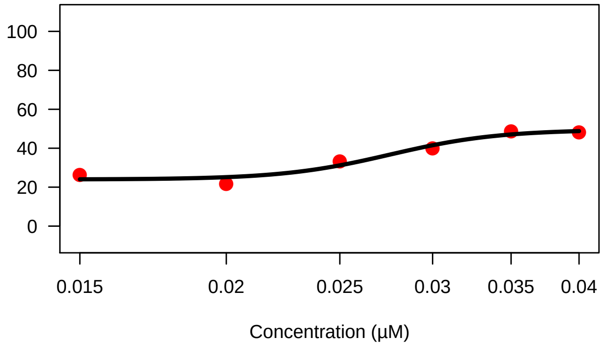

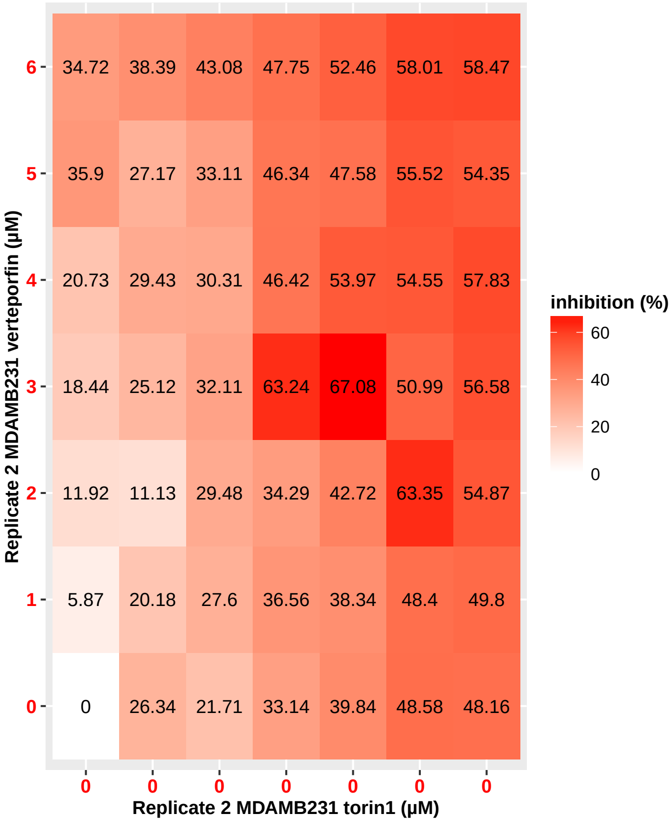

Replicate 3 MDAMB231 torin1 & Replicate 3 MDAMB231 verteporfin

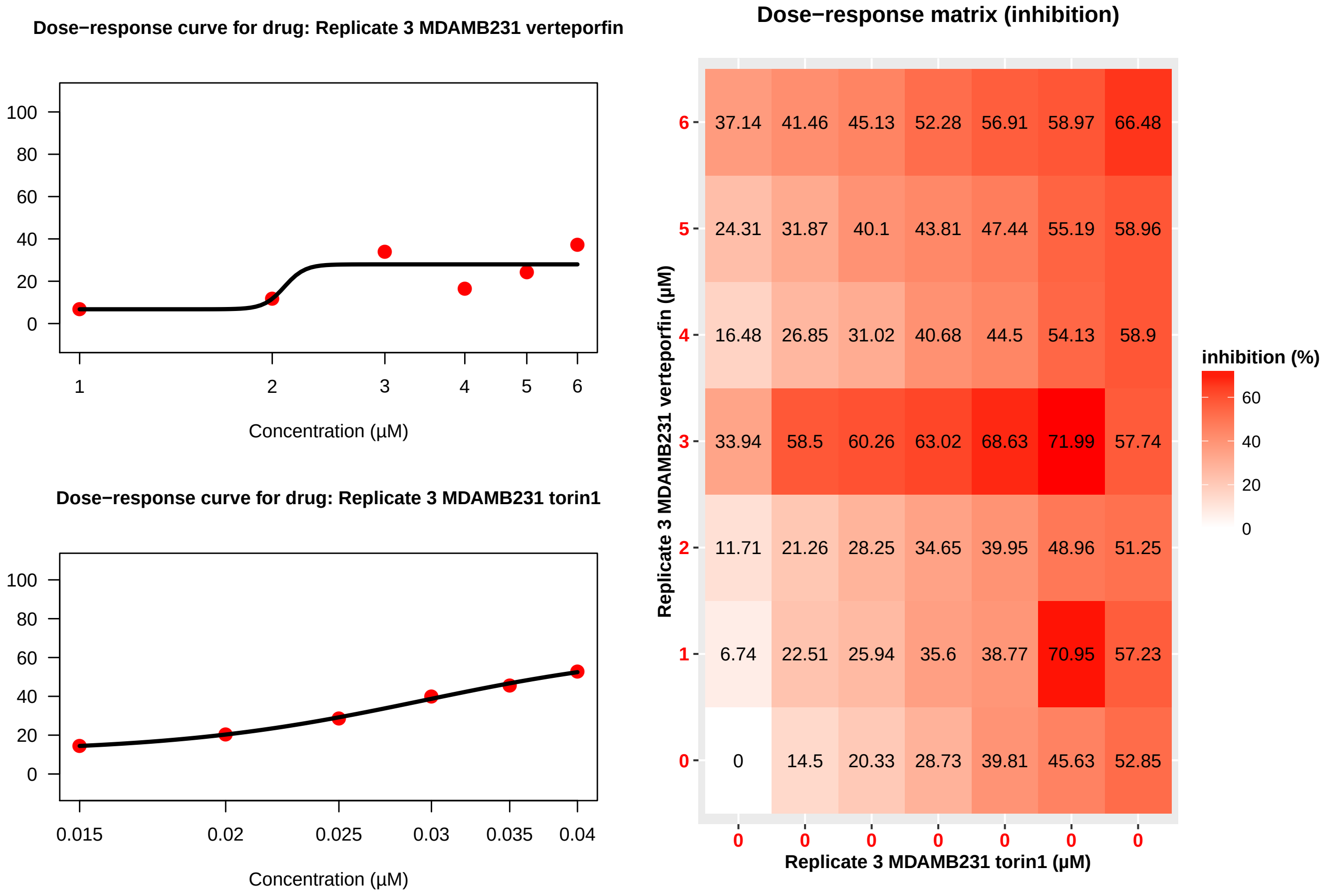

average SUM1315 torin1 & average SUM1315 verteporfin

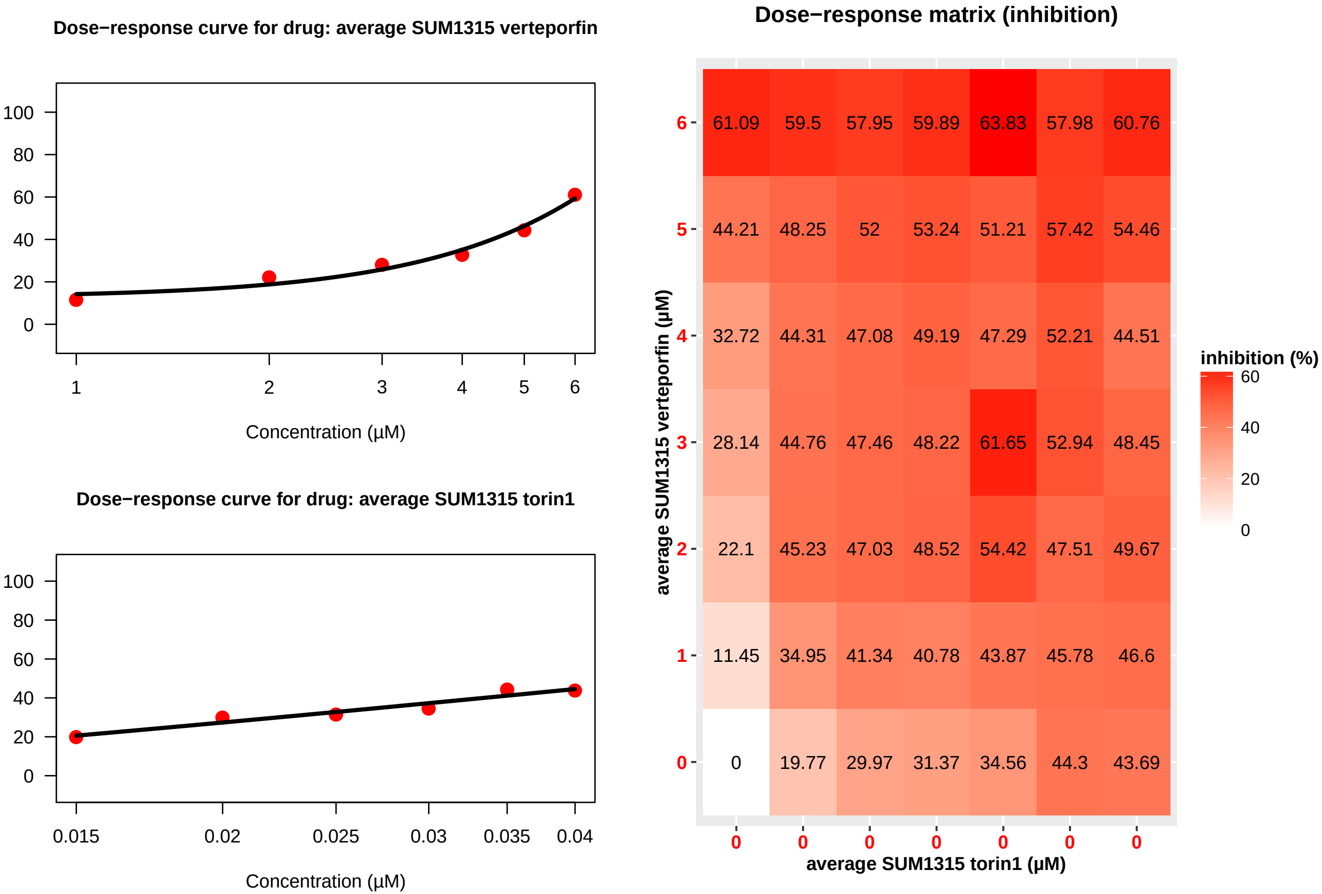

Replicate 1 SUM1315 torin1 & Replicate 1 SUM1315 verteporfin

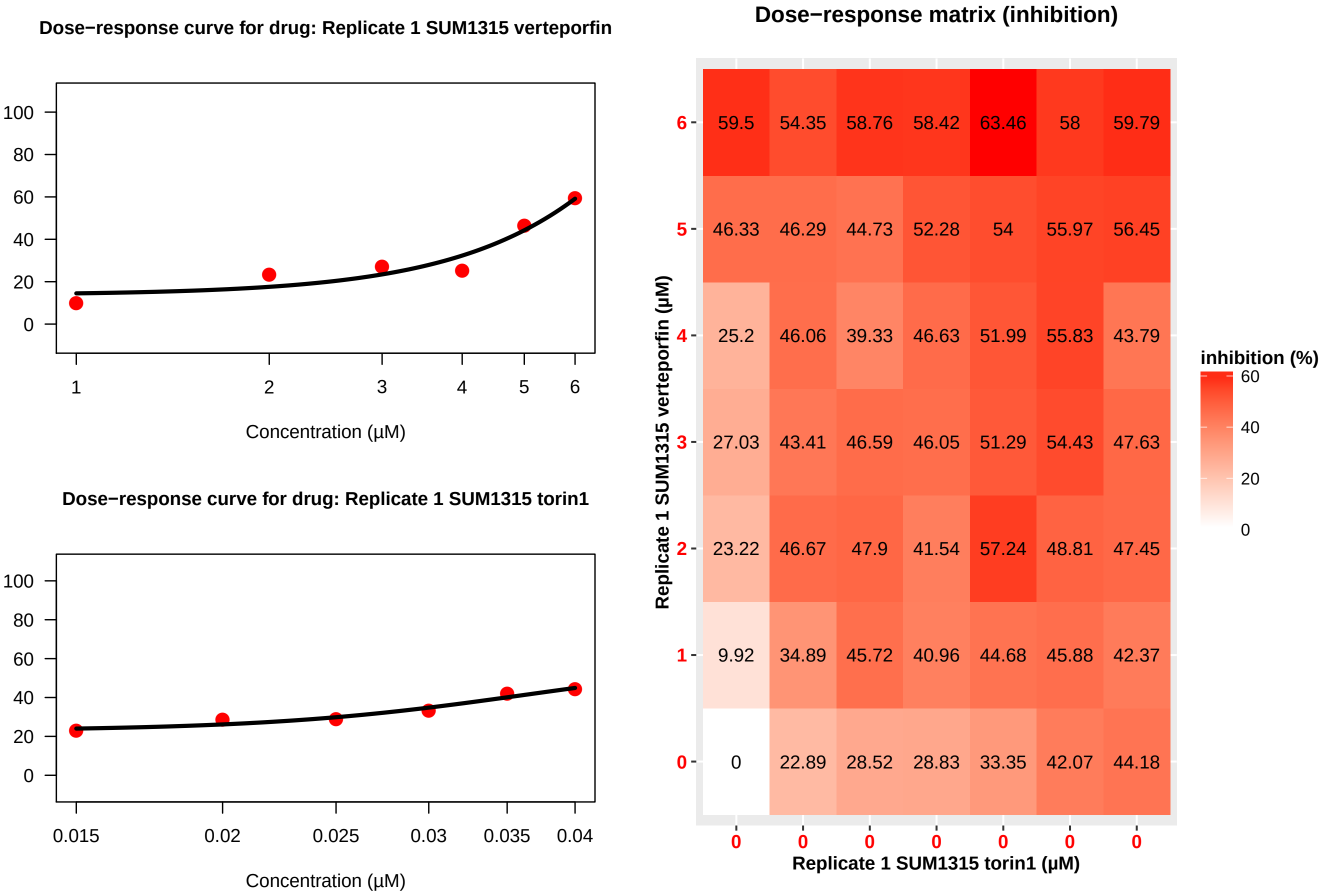

Replicate 2 SUM1315 torin1 & Replicate 2 SUM1315 verteporfin

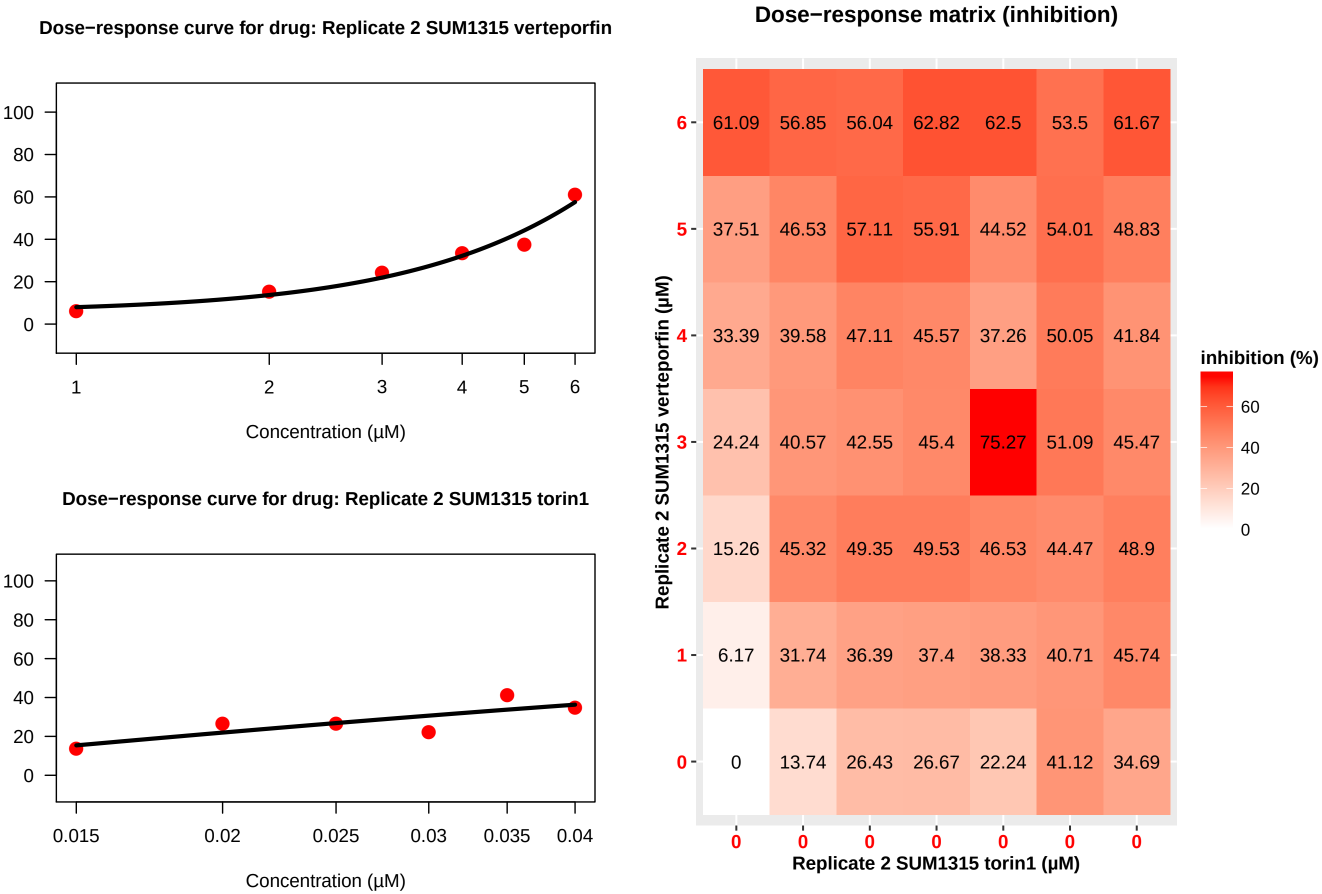

Replicate 3 SUM1315 torin1 & Replicate 3 SUM1315 verteporfin

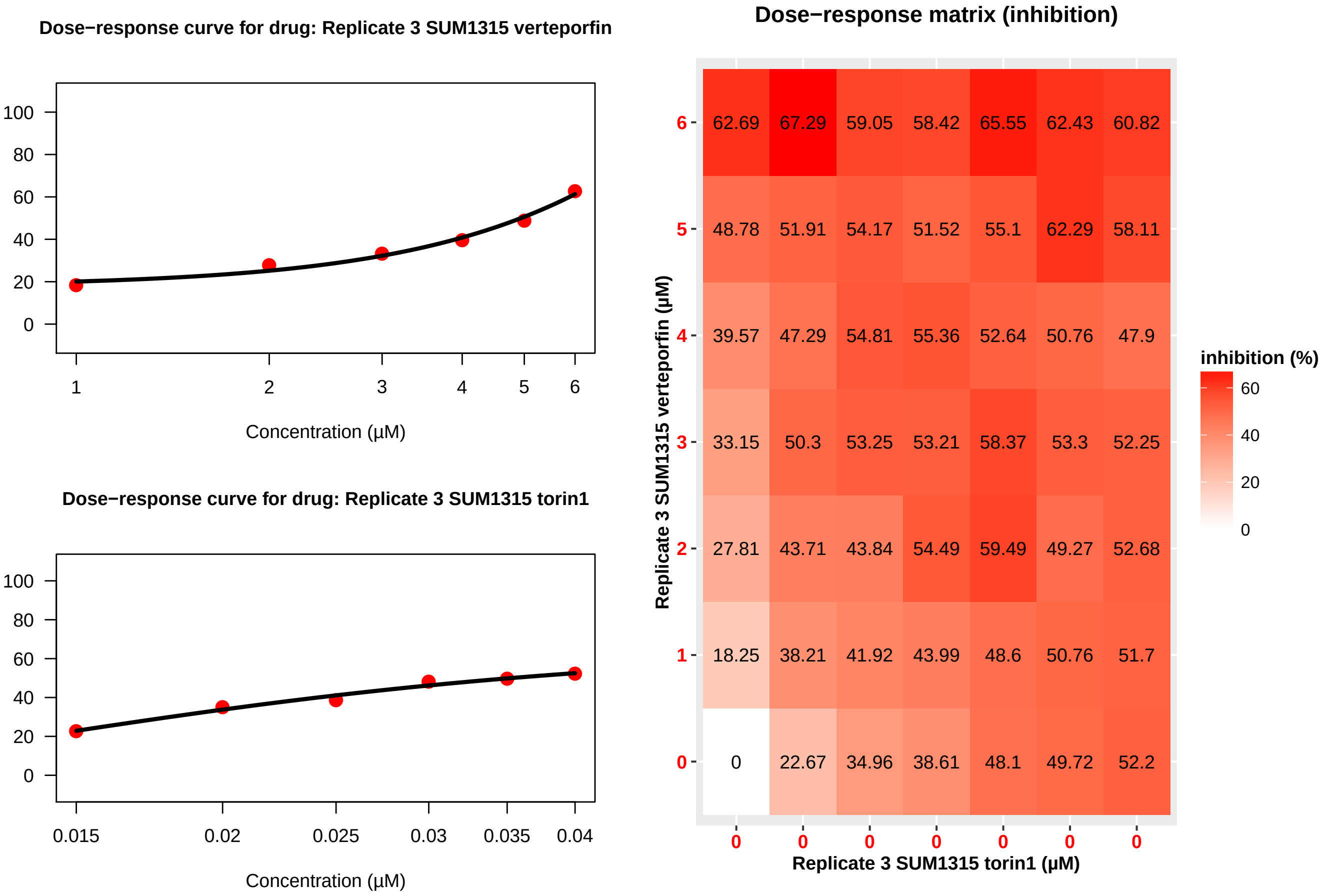

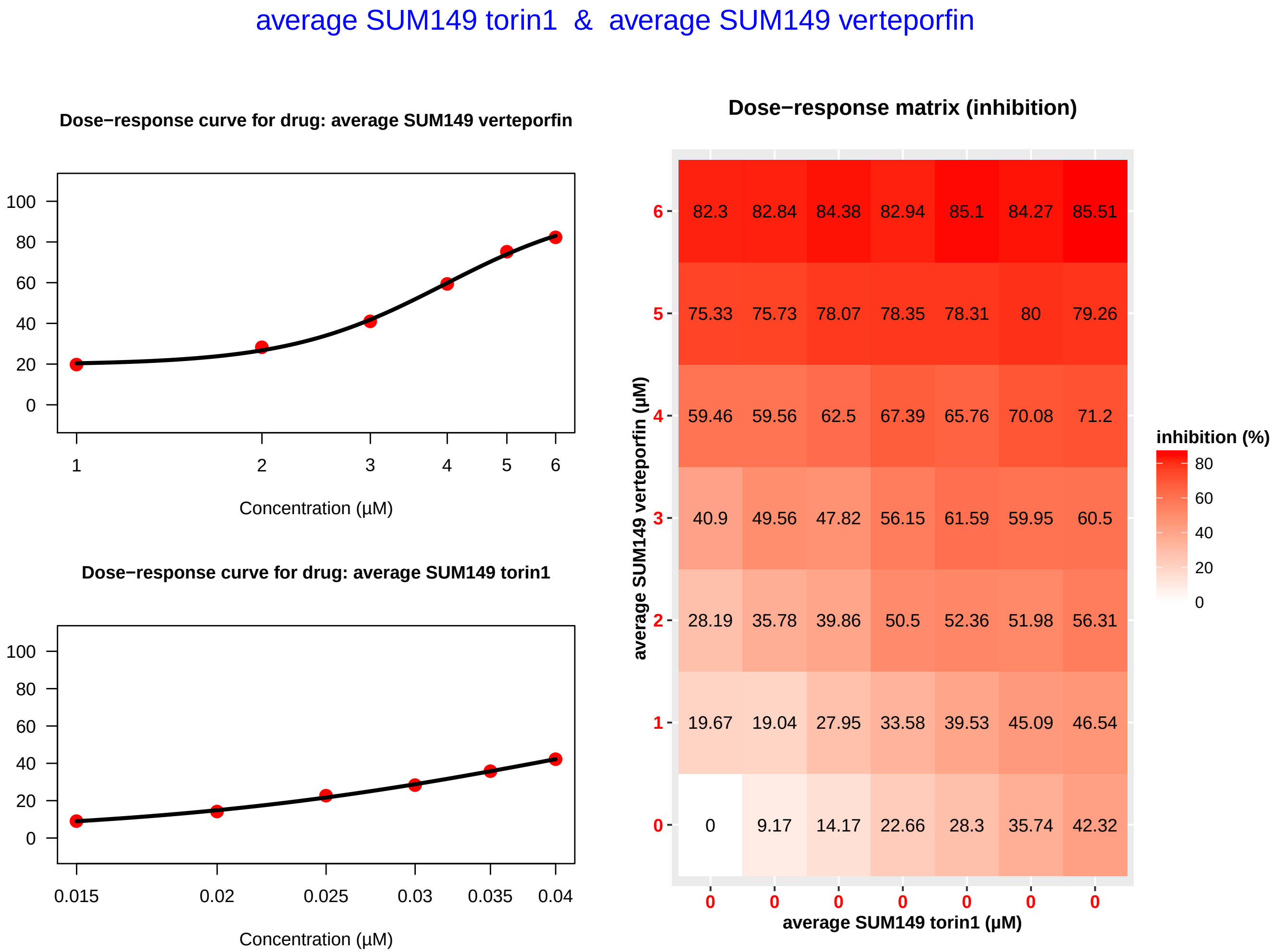

average SUM149 torin1 & average SUM149 verteporfin
Dose−response curve for drug: average SUM149 verteporfin
Concentration (µM)
Dose−response curve for drug: average SUM149 torin1
Concentration (µM)
Dose−response matrix (inhibition)
average SUM149 verteporfin (µM)
average SUM149 torin1 (µM)
inhibition (%)
6: 82.3 82.84 84.38 82.94 85.1 84.27 85.51
5: 75.33 75.73 78.07 78.35 78.31 80 79.26
4: 59.46 59.56 62.5 67.39 65.76 70.08 71.2
3: 40.9 49.56 47.82 56.15 61.59 59.95 60.5
2: 28.19 35.78 39.86 50.5 52.36 51.98 56.31
1: 19.67 19.04 27.95 33.58 39.53 45.09 46.54
0: 0 9.17 14.17 22.66 28.3 35.74 42.32
0 0 0 0 0 0 0

Replicate 1 SUM149 torin1 & Replicate 1 SUM149 verteporfin

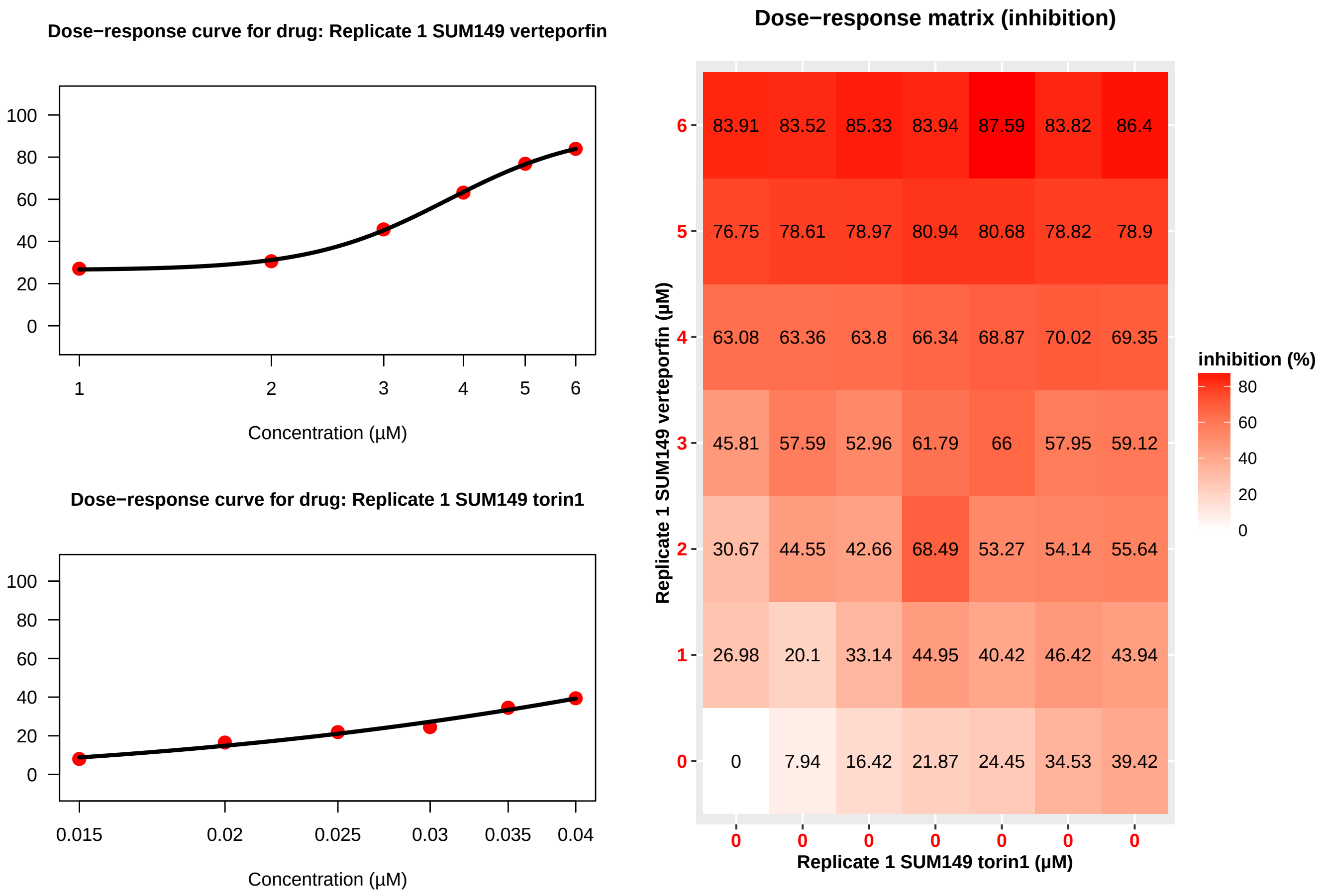

Replicate 2 SUM149 torin1 & Replicate 2 SUM149 verteporfin

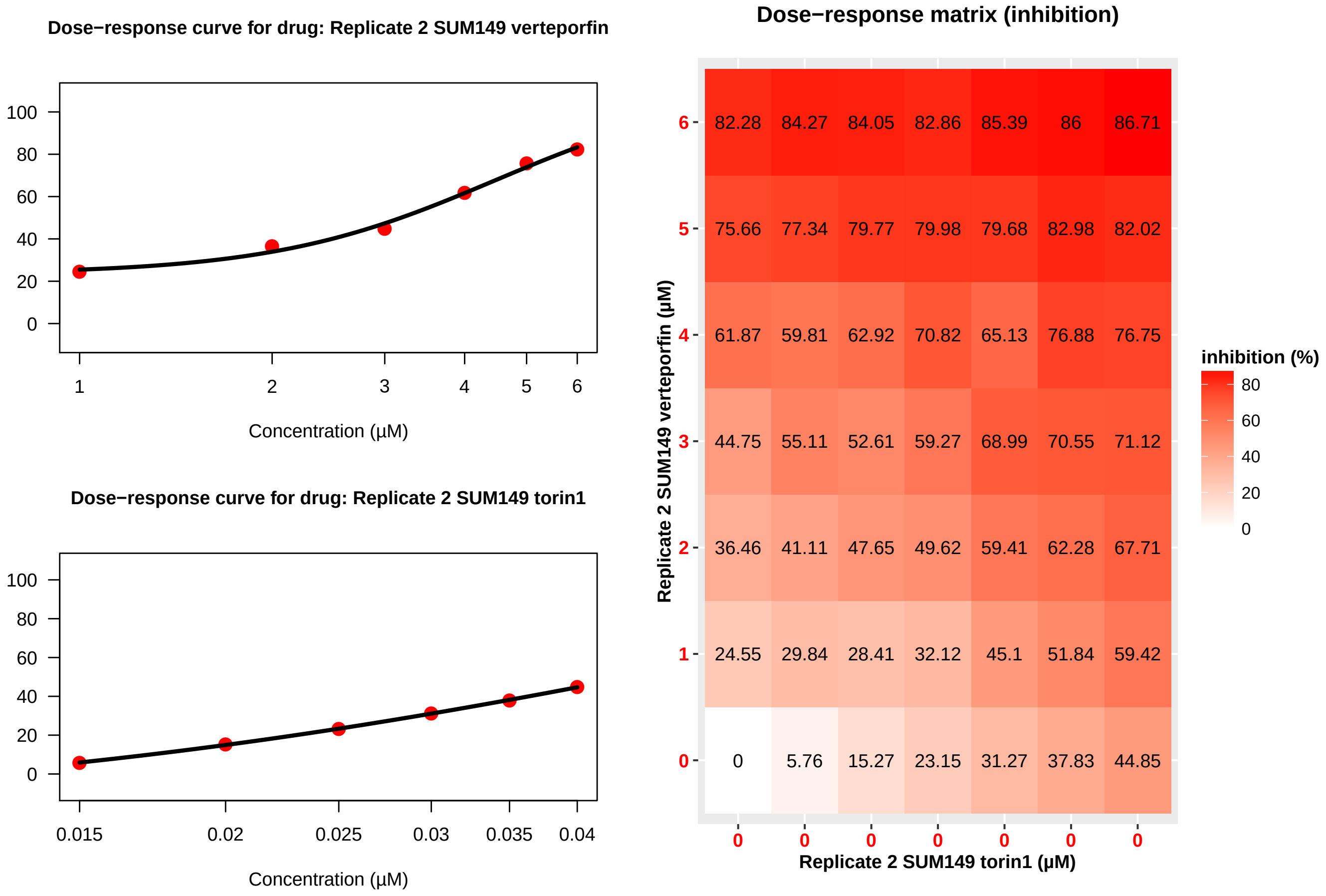

Replicate 3 SUM149 torin1 & Replicate 3 SUM149 verteporfin

Dose−response curve for drug: Replicate 3 SUM149 verteporfin

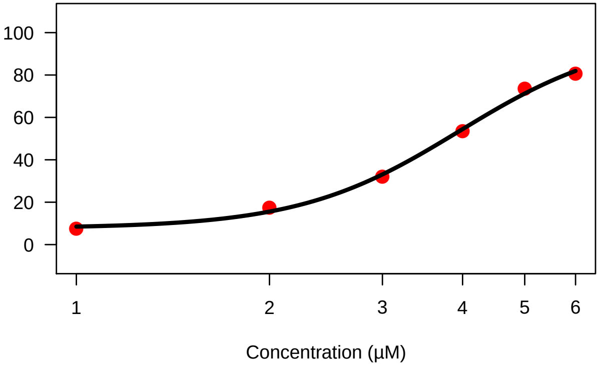

Dose−response curve for drug: Replicate 3 SUM149 torin1

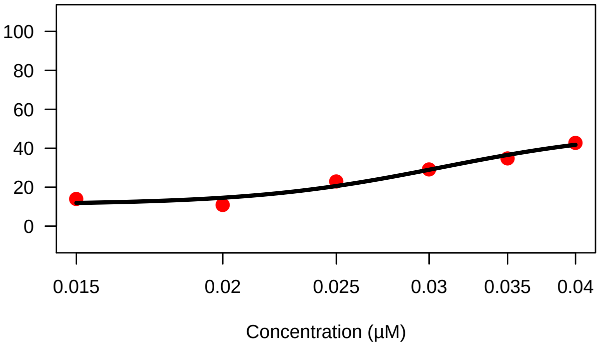

Dose−response matrix (inhibition)

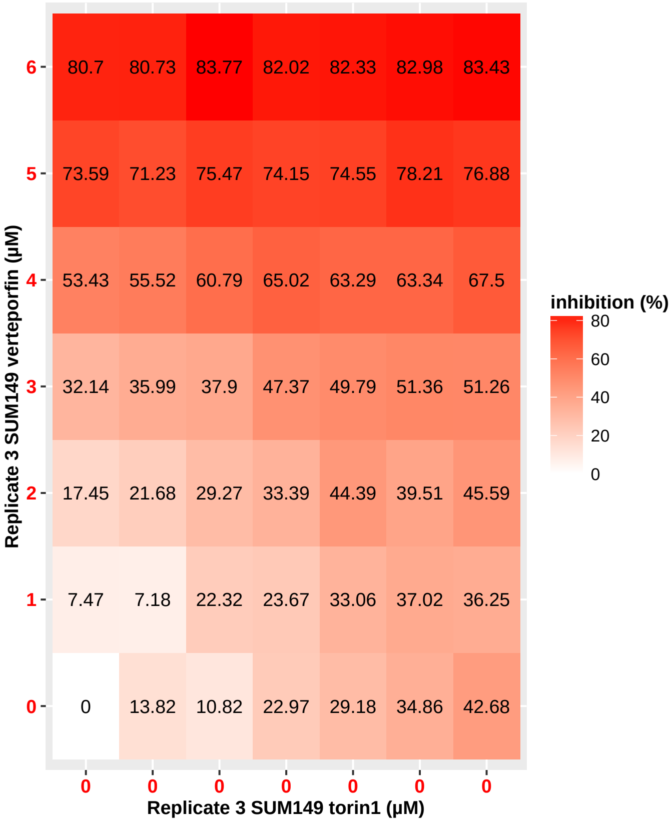

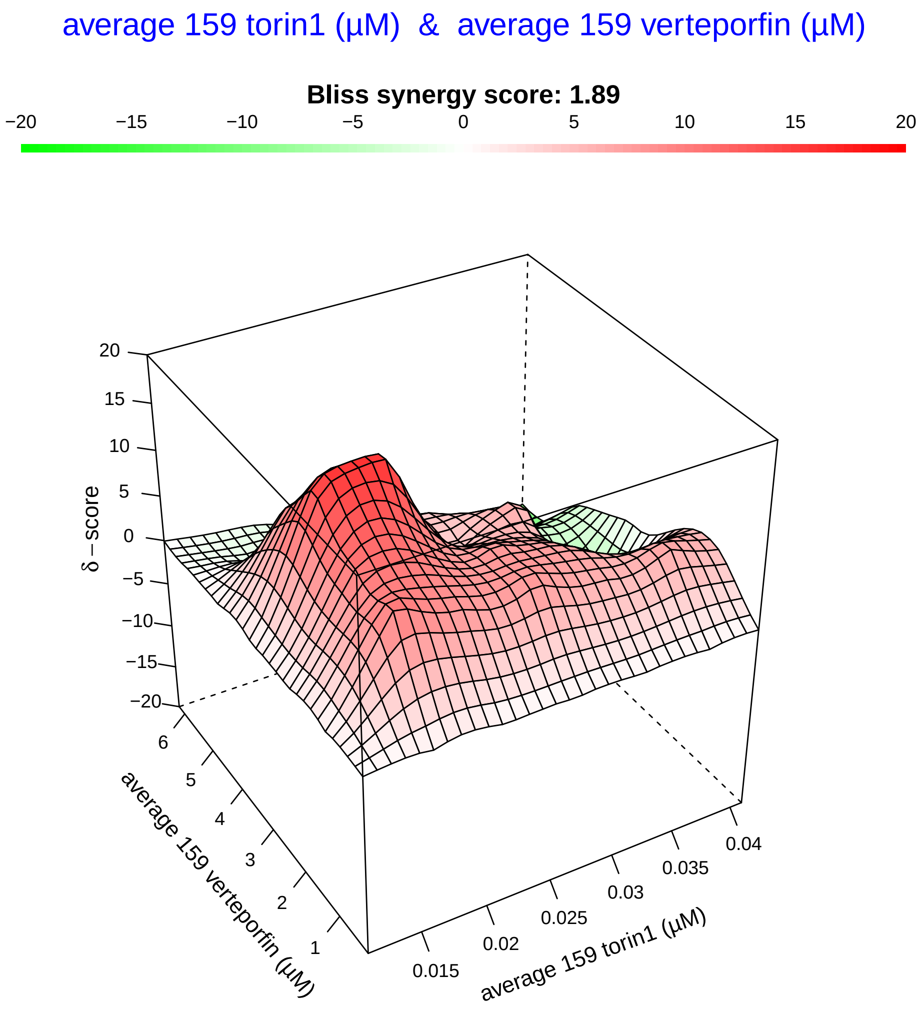
average 159 torin1 (µM) & average 159 verteporfin (µM)
Bliss synergy score: 1.89
−20
−15
−10
−5
0
5
10
15
20
δ − score
20
15
10
5
0
−5
−10
−15
−20
average 159 verteporfin (µM)
6
5
4
3
2
1
average 159 torin1 (µM)
0.015
0.02
0.025
0.03
0.035
0.04

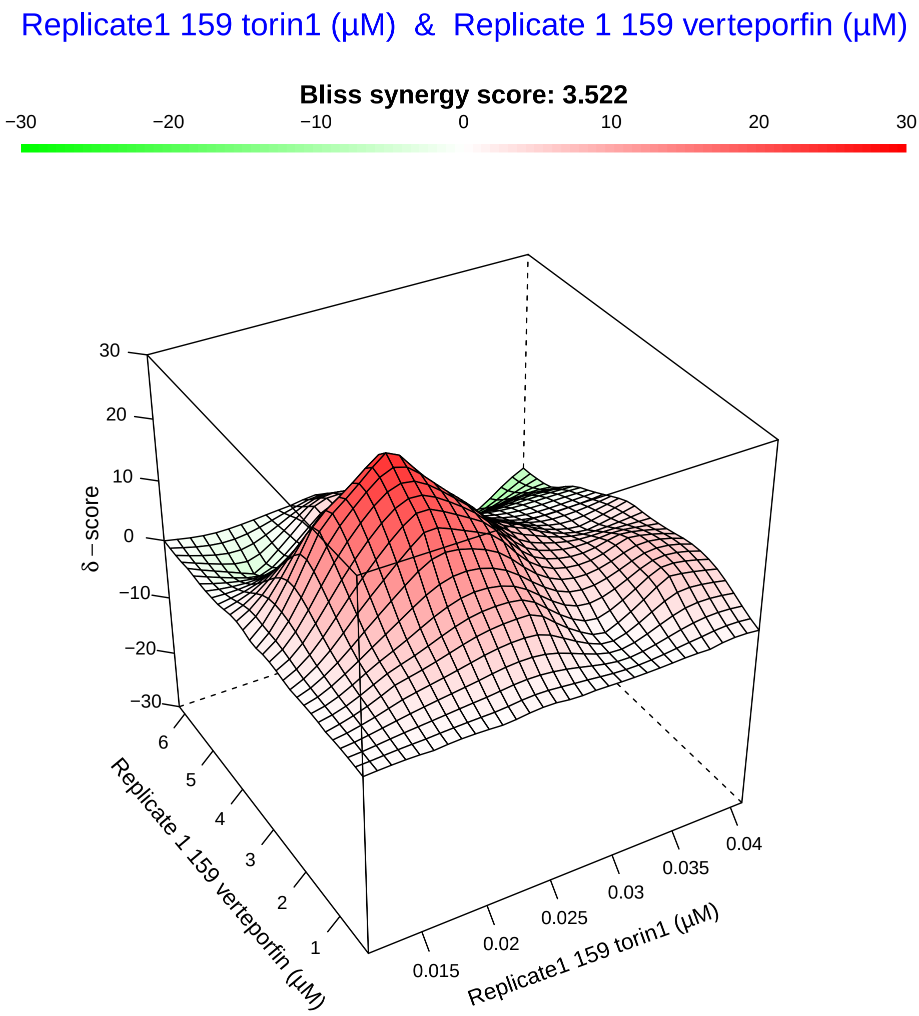
Replicate1 159 torin1 (µM) & Replicate 1 159 verteporfin (µM)
Bliss synergy score: 3.522
−30
−20
−10
0
10
20
30
δ − score
Replicate 1 159 verteporfin (µM)
Replicate1 159 torin1 (µM)
0.015
0.02
0.025
0.03
0.035
0.04
1
2
3
4
5
6

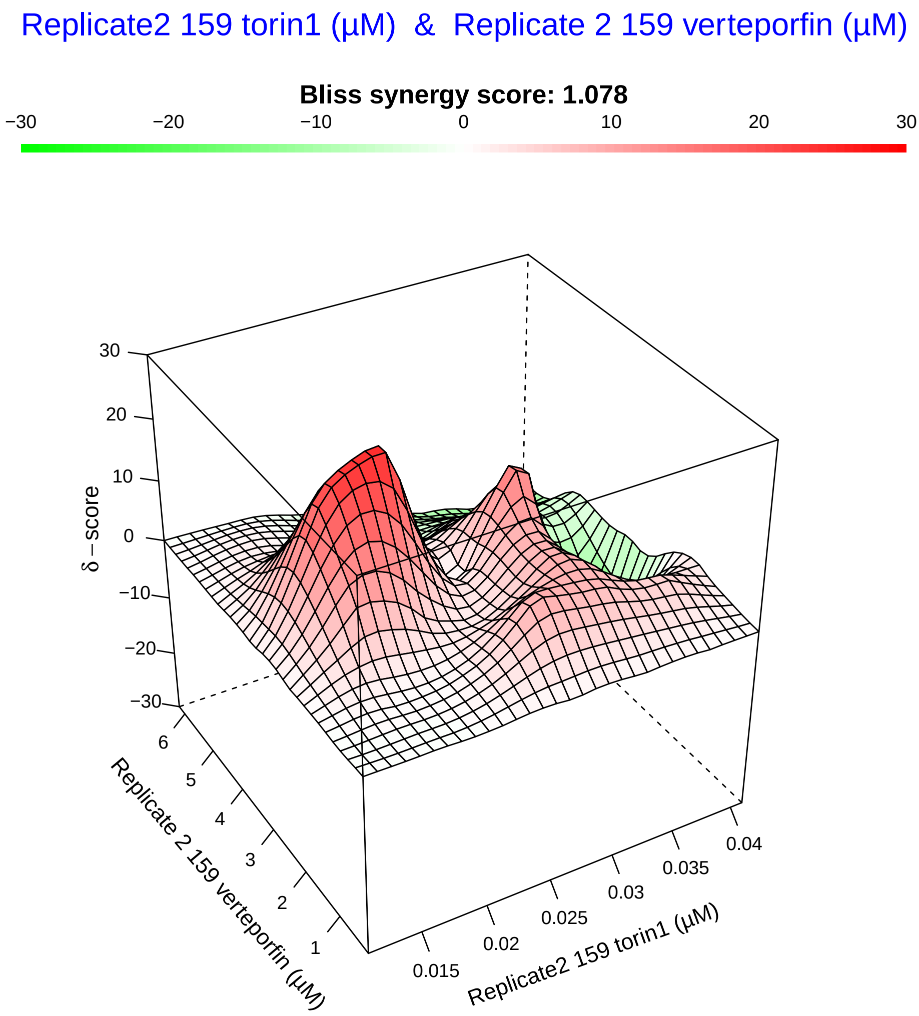

Replicate2 159 torin1 (µM) & Replicate 2 159 verteporfin (µM)
Bliss synergy score: 1.078
−30
−20
−10
0
10
20
30
δ − score
30
20
10
0
−10
−20
−30
6
5
4
3
2
1
Replicate 2 159 verteporfin (µM)
0.015
0.02
0.025
0.03
0.035
0.04
Replicate2 159 torin1 (µM)

Replicate 3 159 torin1 (µM) & Replicate 3 159 verteporfin (µM)

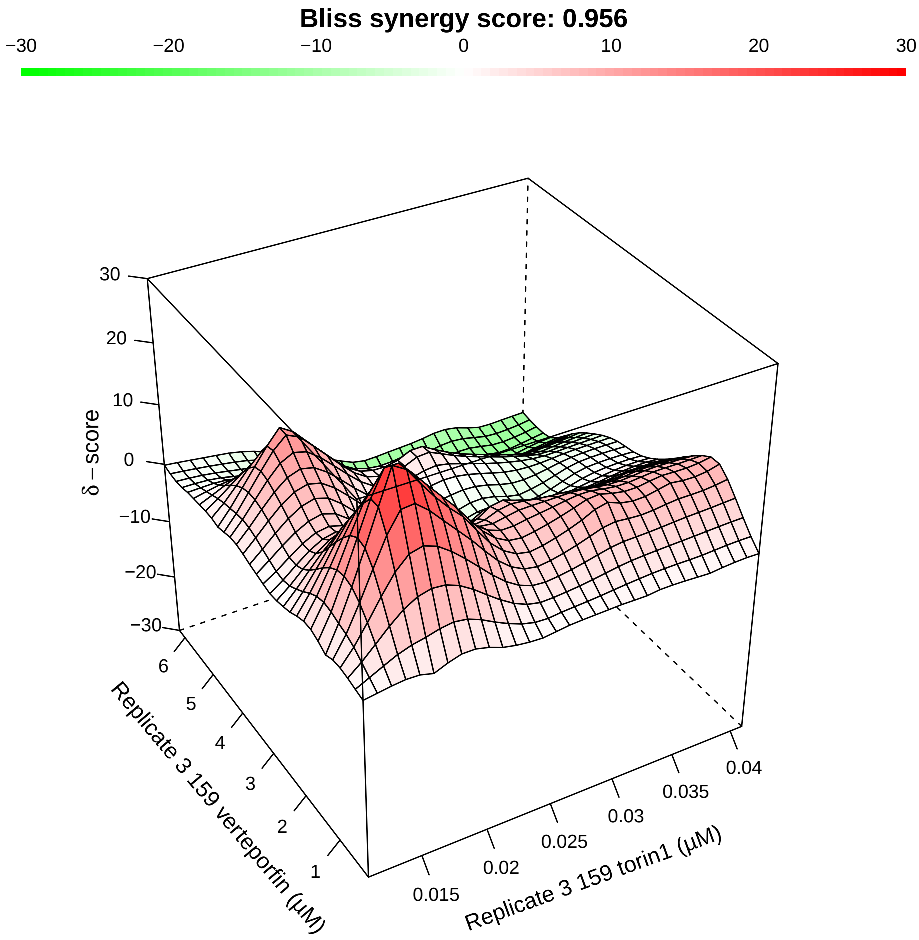

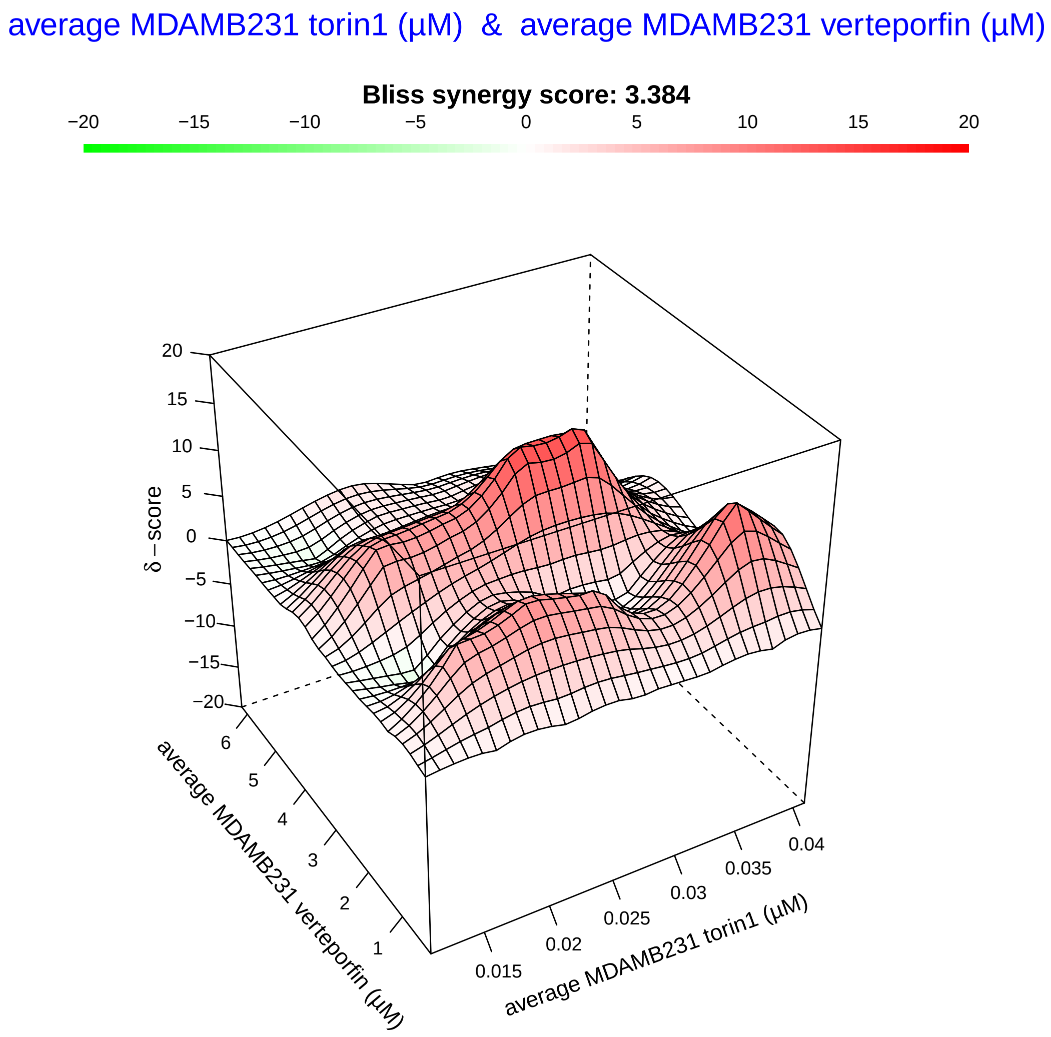

average MDAMB231 torin1 (µM) & average MDAMB231 verteporfin (µM)
Bliss synergy score: 3.384
−20
−15
−10
−5
0
5
10
15
20
δ − score
average MDAMB231 verteporfin (µM)
1
2
3
4
5
6
average MDAMB231 torin1 (µM)
0.015
0.02
0.025
0.03
0.035
0.04

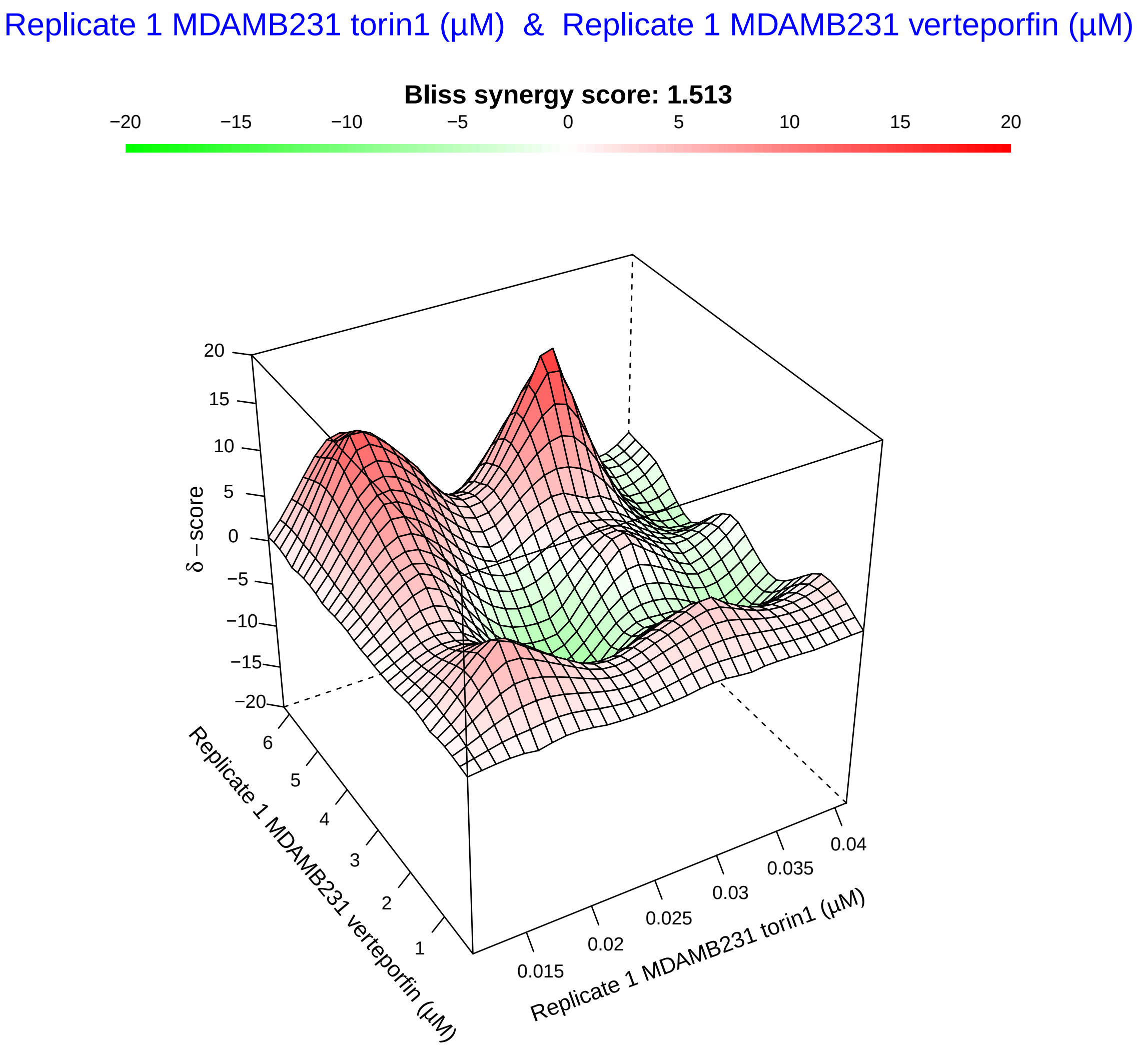
Replicate 1 MDAMB231 torin1 (µM) & Replicate 1 MDAMB231 verteporfin (µM)
Bliss synergy score: 1.513
−20
−15
−10
−5
0
5
10
15
20
δ − score
Replicate 1 MDAMB231 verteporfin (µM)
1
2
3
4
5
6
Replicate 1 MDAMB231 torin1 (µM)
0.015
0.02
0.025
0.03
0.035
0.04

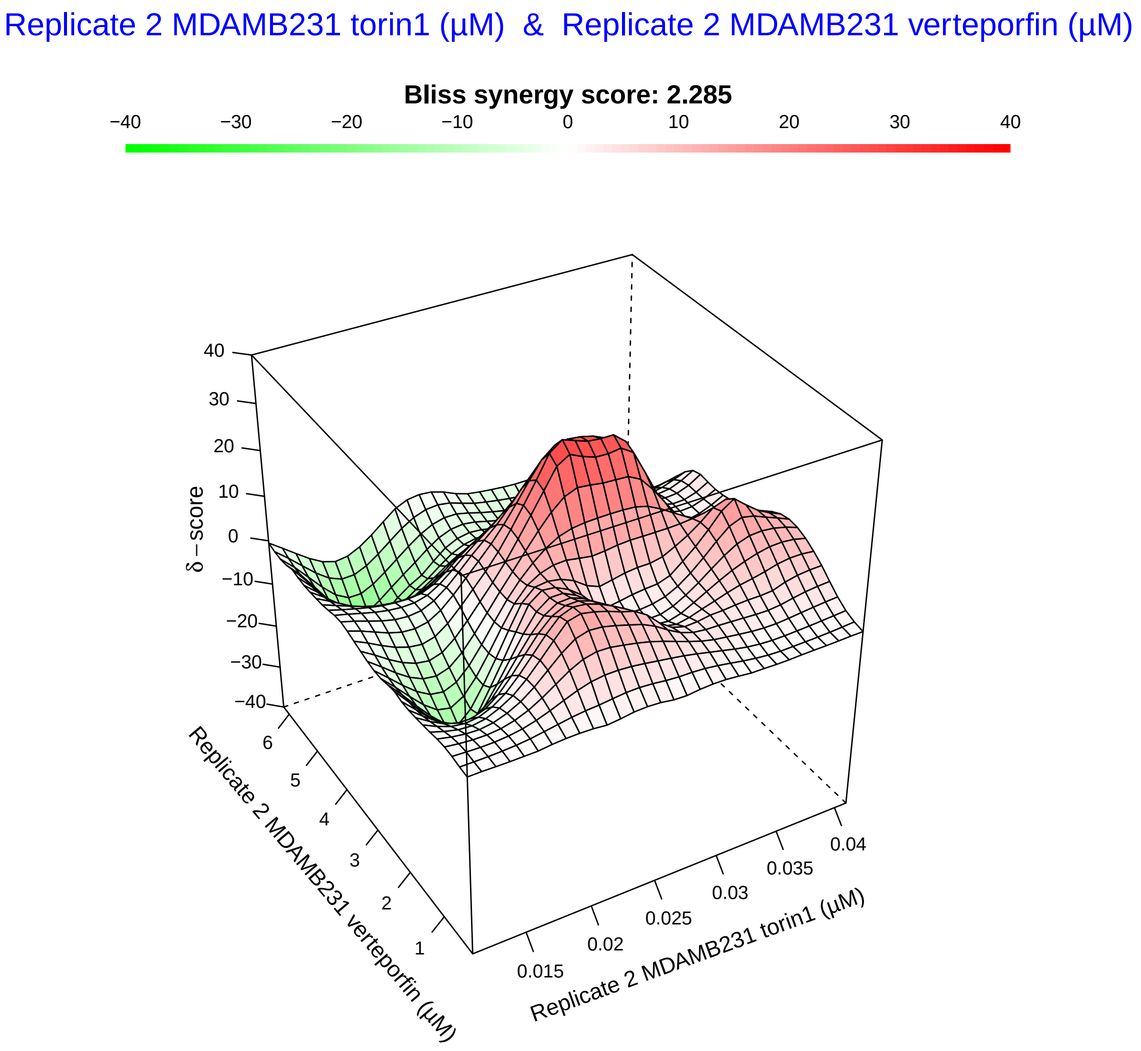
Replicate 2 MDAMB231 torin1 (µM) & Replicate 2 MDAMB231 verteporfin (µM)
Bliss synergy score: 2.285
−40
−30
−20
−10
0
10
20
30
40
δ − score
Replicate 2 MDAMB231 verteporfin (µM)
1
2
3
4
5
6
Replicate 2 MDAMB231 torin1 (µM)
0.015
0.02
0.025
0.03
0.035
0.04

Replicate 3 MDAMB231 torin1 (µM) & Replicate 3 MDAMB231 verteporfin (µM)

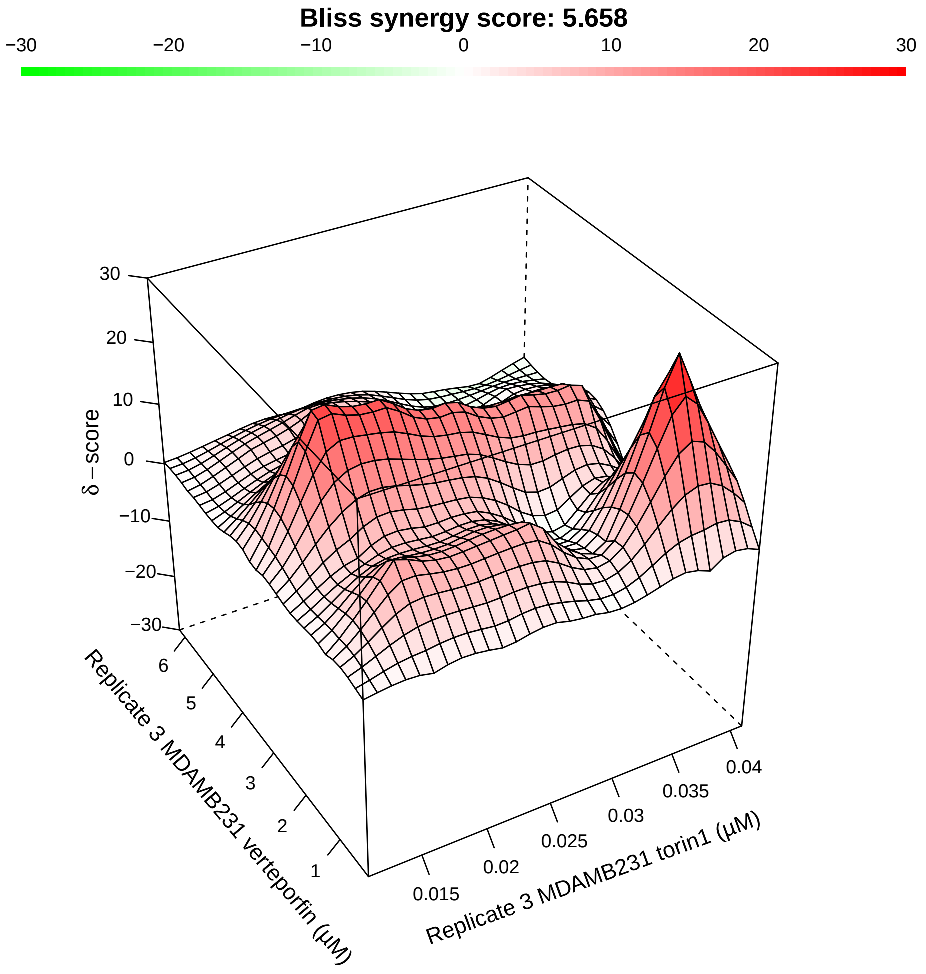

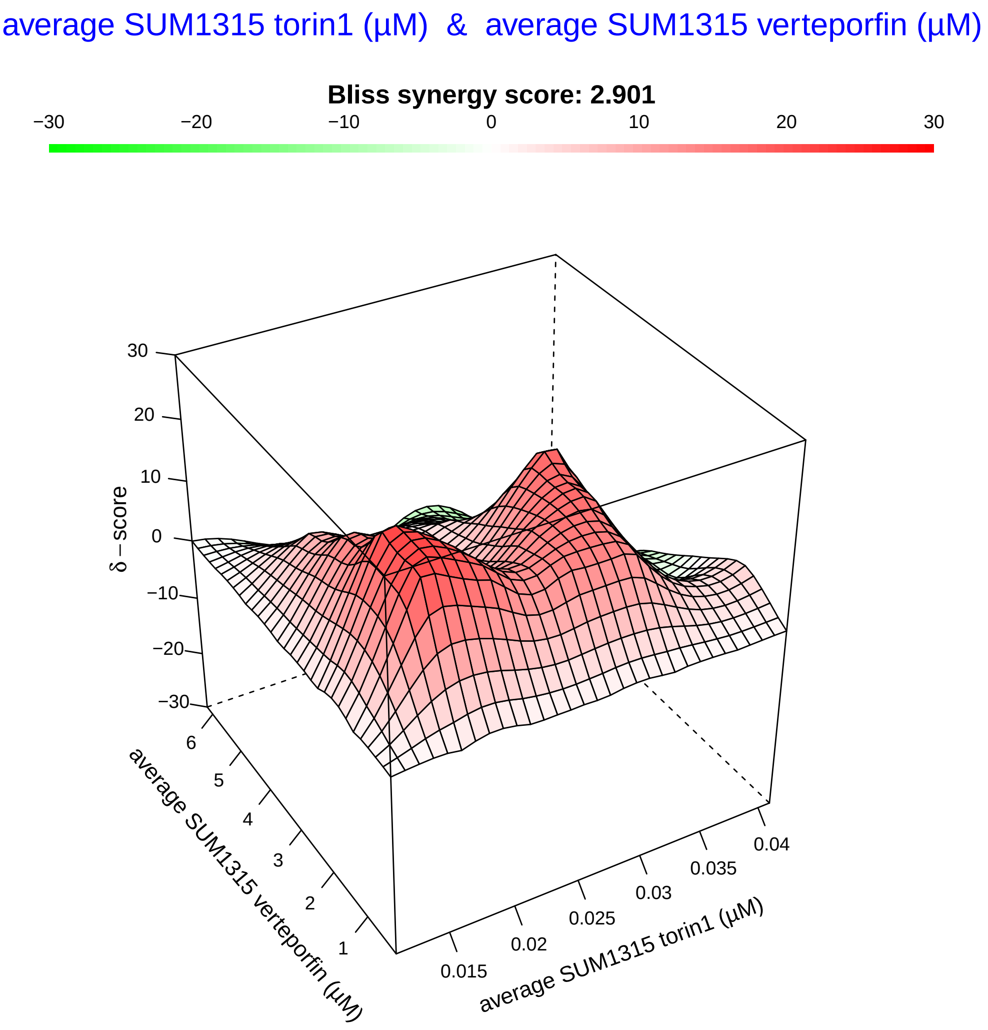
average SUM1315 torin1 (µM) & average SUM1315 verteporfin (µM)
Bliss synergy score: 2.901
−30
−20
−10
0
10
20
30
δ − score
average SUM1315 verteporfin (µM)
average SUM1315 torin1 (µM)
1
2
3
4
5
6
0.015
0.02
0.025
0.03
0.035
0.04

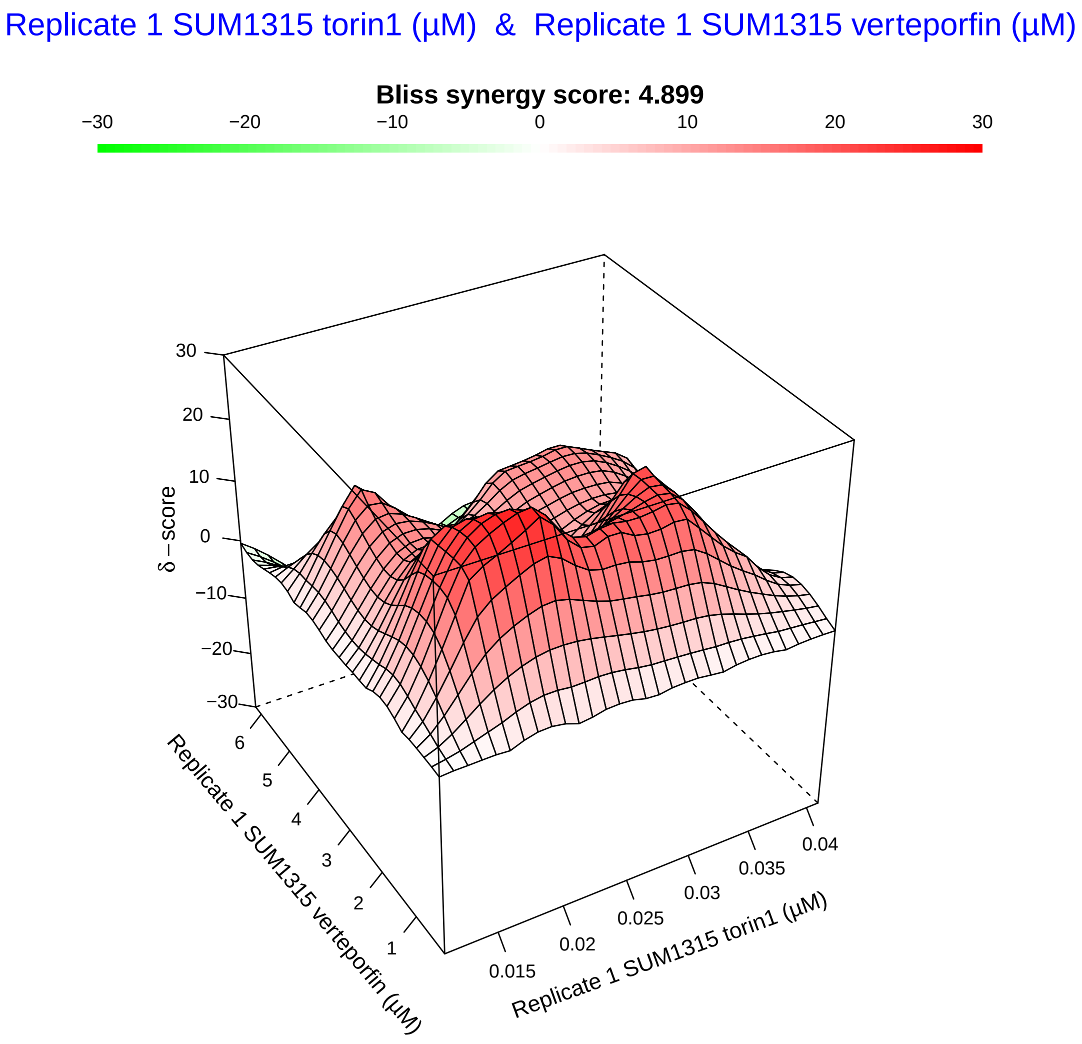

Replicate 1 SUM1315 torin1 (µM) & Replicate 1 SUM1315 verteporfin (µM)
Bliss synergy score: 4.899
−30
−20
−10
0
10
20
30
δ − score
30
20
10
0
−10
−20
−30
6
5
4
3
2
1
Replicate 1 SUM1315 verteporfin (µM)
0.015
0.02
0.025
0.03
0.035
0.04
Replicate 1 SUM1315 torin1 (µM)

Replicate 2 SUM1315 torin1 (µM) & Replicate 2 SUM1315 verteporfin (µM)

Bliss synergy score: 6.339

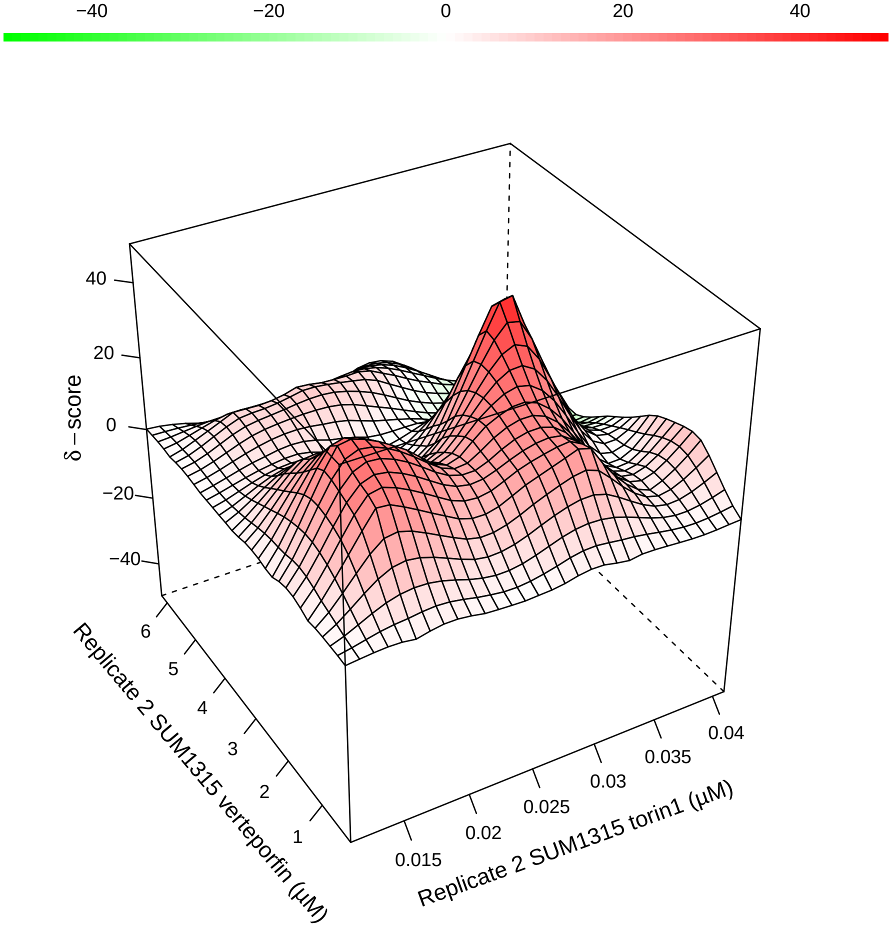

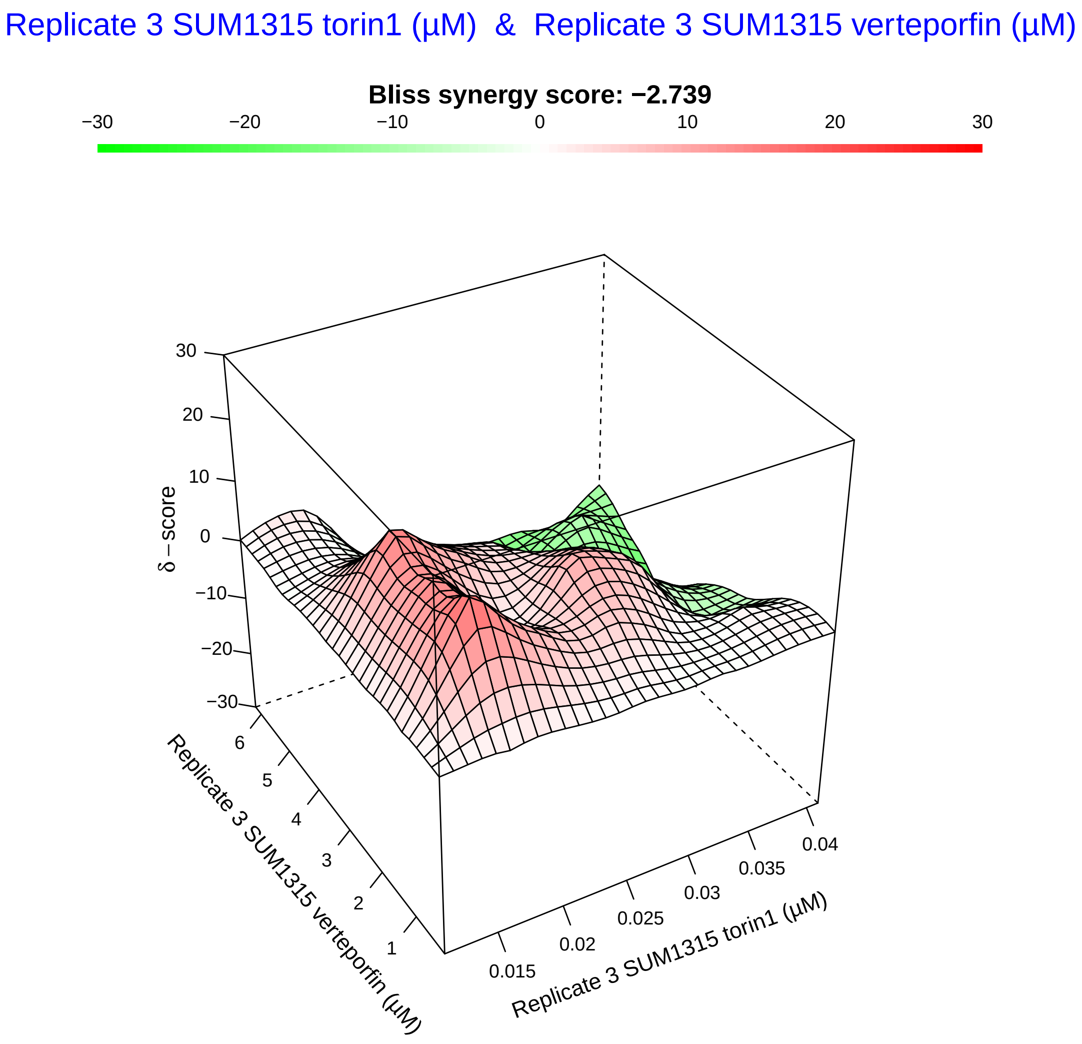

Replicate 3 SUM1315 torin1 (µM) & Replicate 3 SUM1315 verteporfin (µM)
Bliss synergy score: −2.739
−30
−20
−10
0
10
20
30
δ − score
Replicate 3 SUM1315 verteporfin (µM)
1
2
3
4
5
6
Replicate 3 SUM1315 torin1 (µM)
0.015
0.02
0.025
0.03
0.035
0.04

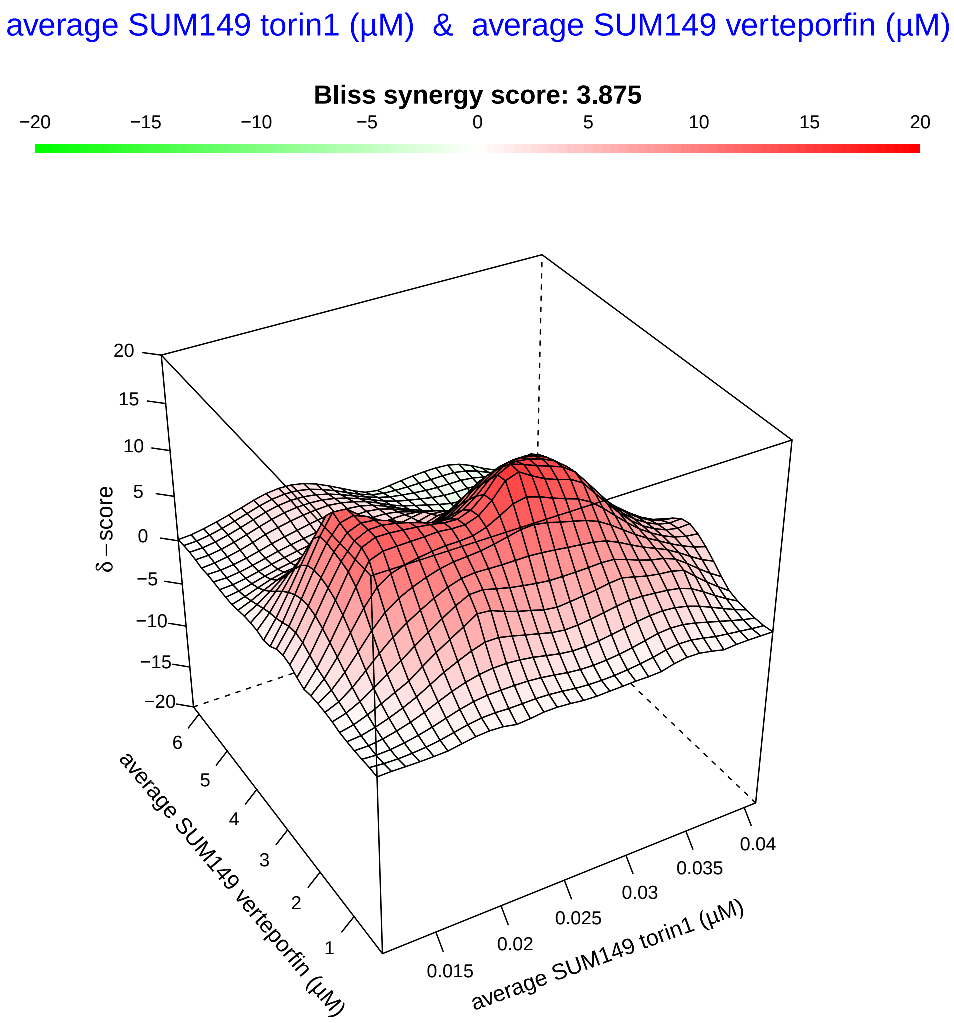
average SUM149 torin1 (µM) & average SUM149 verteporfin (µM)
Bliss synergy score: 3.875
−20
−15
−10
−5
0
5
10
15
20
δ − score
average SUM149 verteporfin (µM)
1
2
3
4
5
6
average SUM149 torin1 (µM)
0.015
0.02
0.025
0.03
0.035
0.04

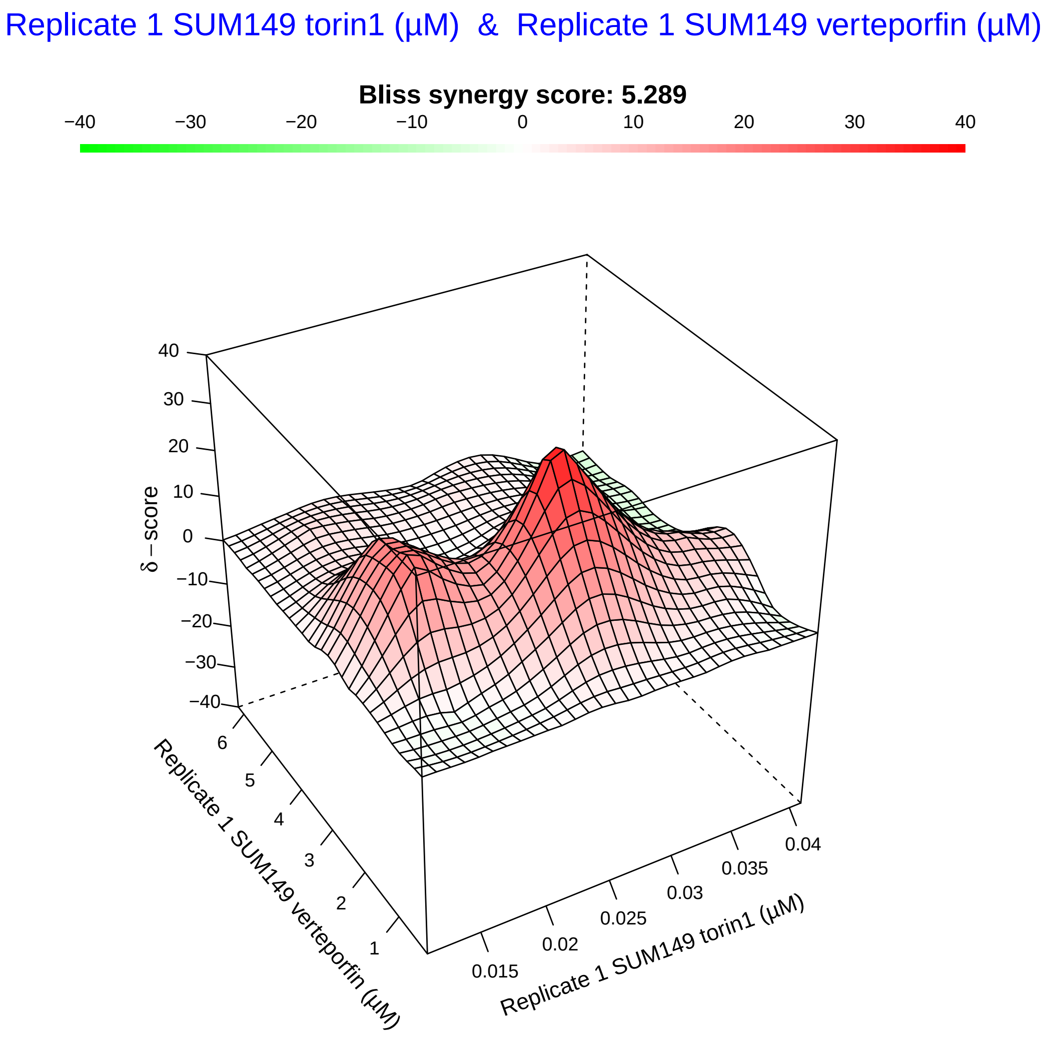
Replicate 1 SUM149 torin1 (µM) & Replicate 1 SUM149 verteporfin (µM)
Bliss synergy score: 5.289
−40
−30
−20
−10
0
10
20
30
40
δ − score
Replicate 1 SUM149 verteporfin (µM)
1
2
3
4
5
6
Replicate 1 SUM149 torin1 (µM)
0.015
0.02
0.025
0.03
0.035
0.04

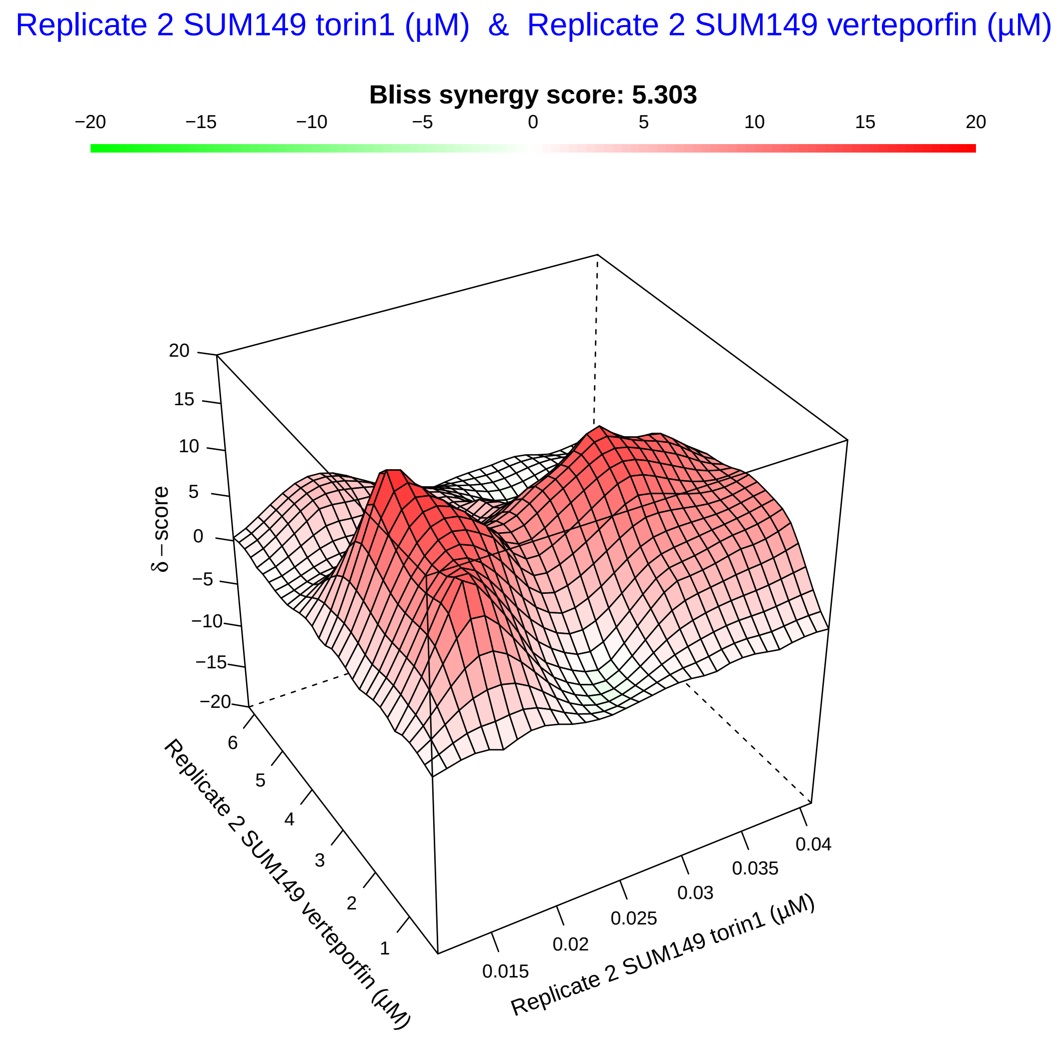
Replicate 2 SUM149 torin1 (µM) & Replicate 2 SUM149 verteporfin (µM)
Bliss synergy score: 5.303
−20
−15
−10
−5
0
5
10
15
20
δ − score
Replicate 2 SUM149 verteporfin (µM)
1
2
3
4
5
6
Replicate 2 SUM149 torin1 (µM)
0.015
0.02
0.025
0.03
0.035
0.04

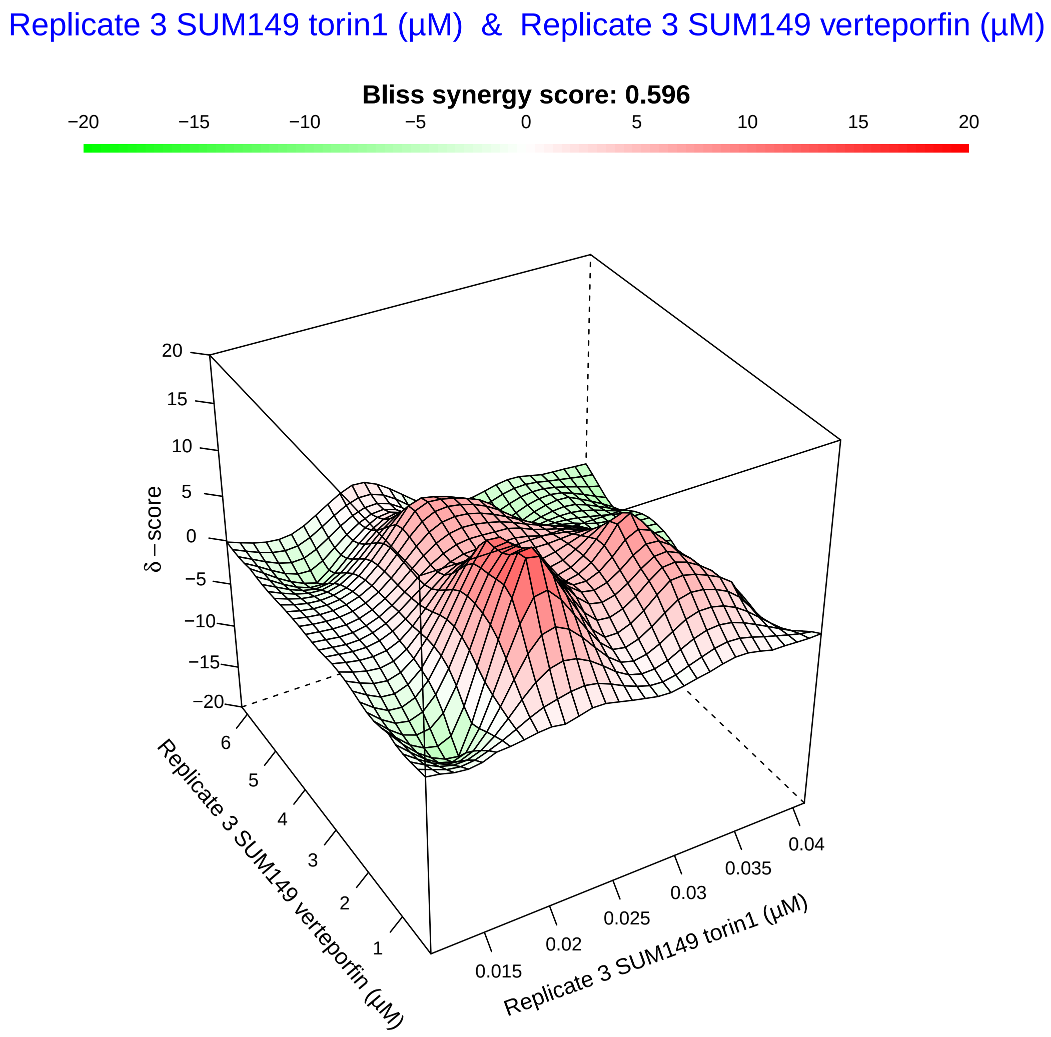

Replicate 3 SUM149 torin1 (µM) & Replicate 3 SUM149 verteporfin (µM)
Bliss synergy score: 0.596
−20
−15
−10
−5
0
5
10
15
20
δ − score
Replicate 3 SUM149 verteporfin (µM)
1
2
3
4
5
6
Replicate 3 SUM149 torin1 (µM)
0.015
0.02
0.025
0.03
0.035
0.04
