# Supplementary material for: In vivo genome-wide CRISPR screen reveals breast cancer vulnerabilities and synergistic mTOR/Hippo targeted combination therapy
Source: Nat Commun. 2021 May 24;12:3055. doi: 10.1038/s41467-021-23316-4 (PMC8144221; doi:10.1038/s41467-021-23316-4)
Supplement: Supplementary file 3 — Source Data [file 41467_2021_23316_MOESM3_ESM.zip › Fig 5d and 5e result_HSA.pdf]

Calculation and Visualization of synergy scores for Drug Combinations

Drug combinations:

Drug combination	Synergy score	Most synergistic area score	M
average 159 torin1 - average 159 verteporfin	8.05	16.12	
Replicate1 159 torin1 - Replicate 1 159 verteporfin	9.39	18.97	
Replicate2 159 torin1 - Replicate 2 159 verteporfin	6.55	16.35	
Replicate 3 159 torin1 - Replicate 3 159 verteporfin	8.11	15.64	
average MDAMB231 torin1 - average MDAMB231 verteporfin	10.33	16.95	
Replicate 1 MDAMB231 torin1 - Replicate 1 MDAMB231 verteporfin	9.72	17.23	
Replicate 2 MDAMB231 torin1 - Replicate 2 MDAMB231 verteporfin	7.25	15.25	
Replicate 3 MDAMB231 torin1 - Replicate 3 MDAMB231 verteporfin	12.12	18.60	
average SUM1315 torin1 - average SUM1315 verteporfin	11.28	20.75	
Replicate 1 SUM1315 torin1 - Replicate 1 SUM1315 verteporfin	11.52	20.76	
Replicate 2 SUM1315 torin1 - Replicate 2 SUM1315 verteporfin	13.48	23.91	
Replicate 3 SUM1315 torin1 - Replicate 3 SUM1315 verteporfin	7.65	17.47	
average SUM149 torin1 - average SUM149 verteporfin	9.62	19.00	
Replicate 1 SUM149 torin1 - Replicate 1 SUM149 verteporfin	9.89	22.46	
Replicate 2 SUM149 torin1 - Replicate 2 SUM149 verteporfin	11.49	24.29	
Replicate 3 SUM149 torin1 - Replicate 3 SUM149 verteporfin	6.11	13.60	

Chosen parameters:

Readout: inhibition ; Baseline correction: Yes ;

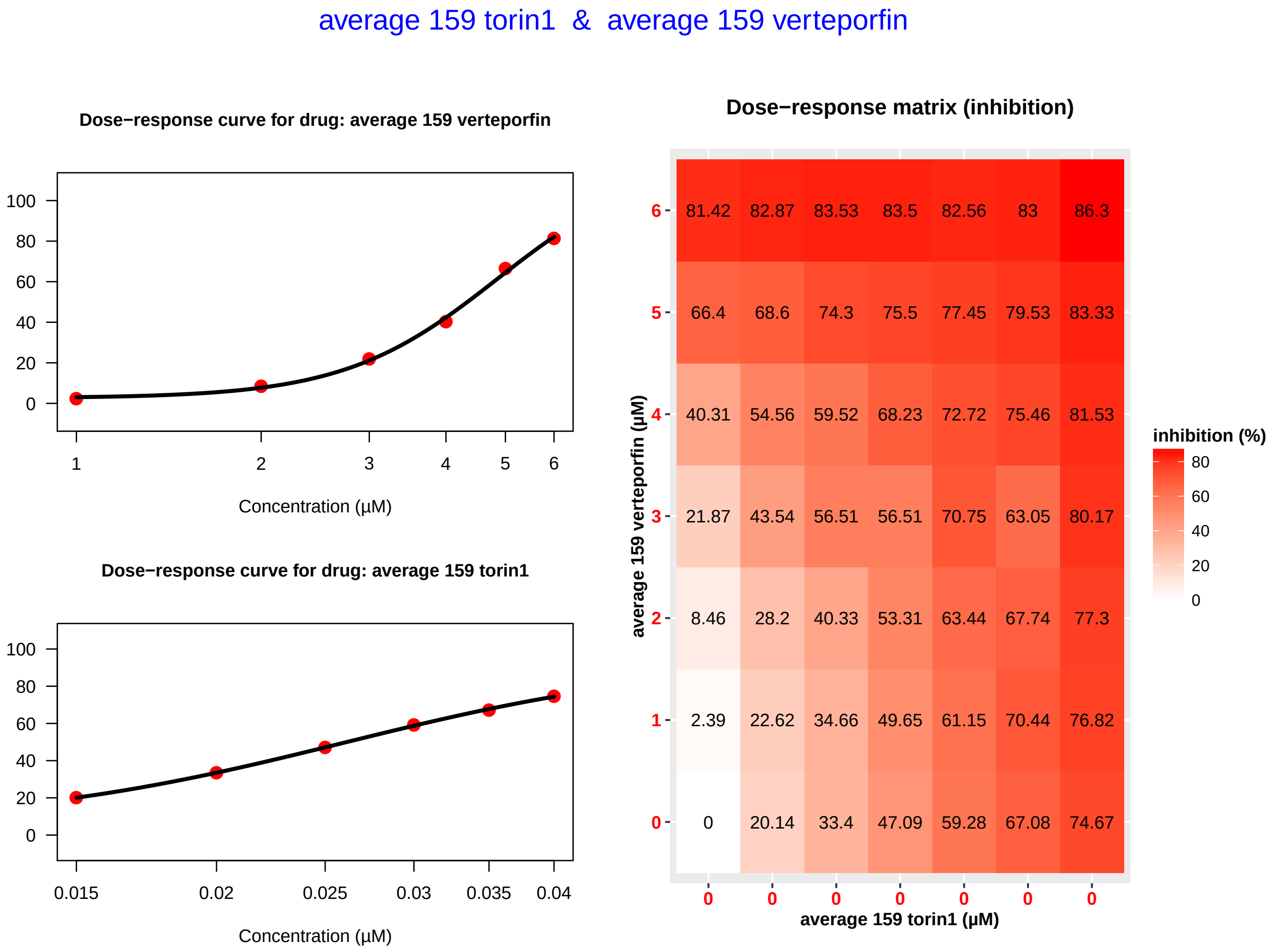
average 159 torin1 & average 159 verteporfin
Dose−response curve for drug: average 159 verteporfin
Concentration (µM)
Dose−response curve for drug: average 159 torin1
Concentration (µM)
Dose−response matrix (inhibition)
average 159 verteporfin (µM)
average 159 torin1 (µM)
inhibition (%)
81.42 82.87 83.53 83.5 82.56 83 86.3
66.4 68.6 74.3 75.5 77.45 79.53 83.33
40.31 54.56 59.52 68.23 72.72 75.46 81.53
21.87 43.54 56.51 56.51 70.75 63.05 80.17
8.46 28.2 40.33 53.31 63.44 67.74 77.3
2.39 22.62 34.66 49.65 61.15 70.44 76.82
0 20.14 33.4 47.09 59.28 67.08 74.67

Replicate1 159 torin1 & Replicate 1 159 verteporfin

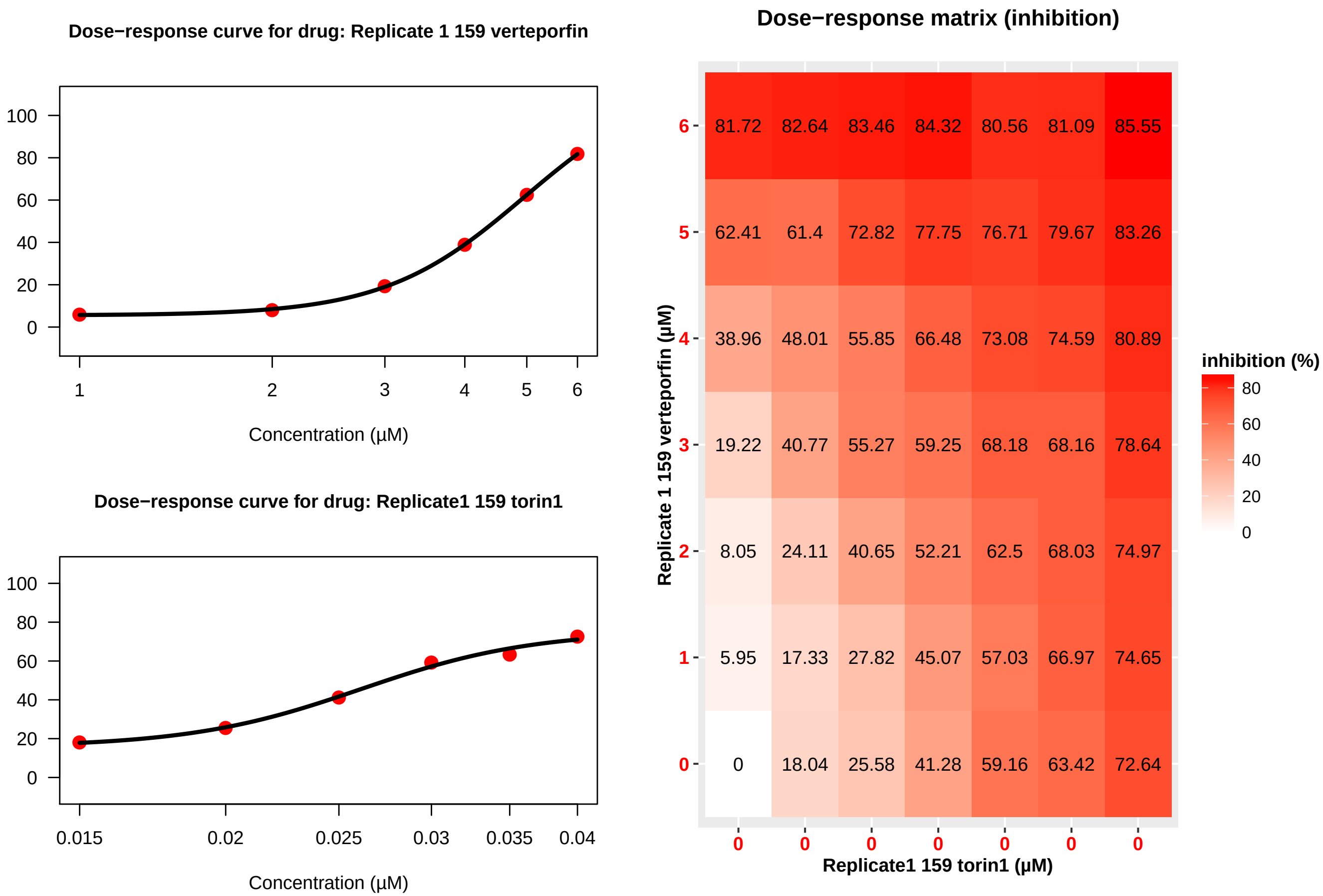

Replicate2 159 torin1 & Replicate 2 159 verteporfin

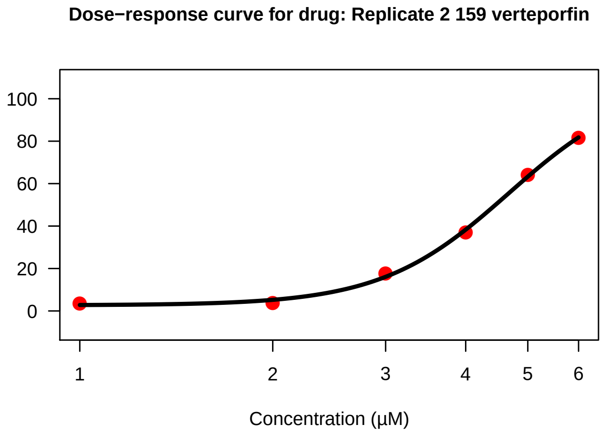

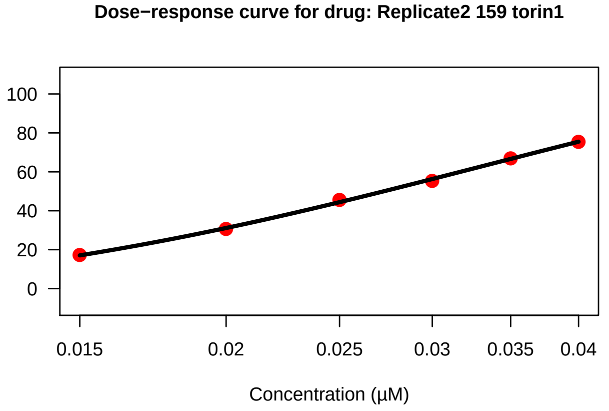

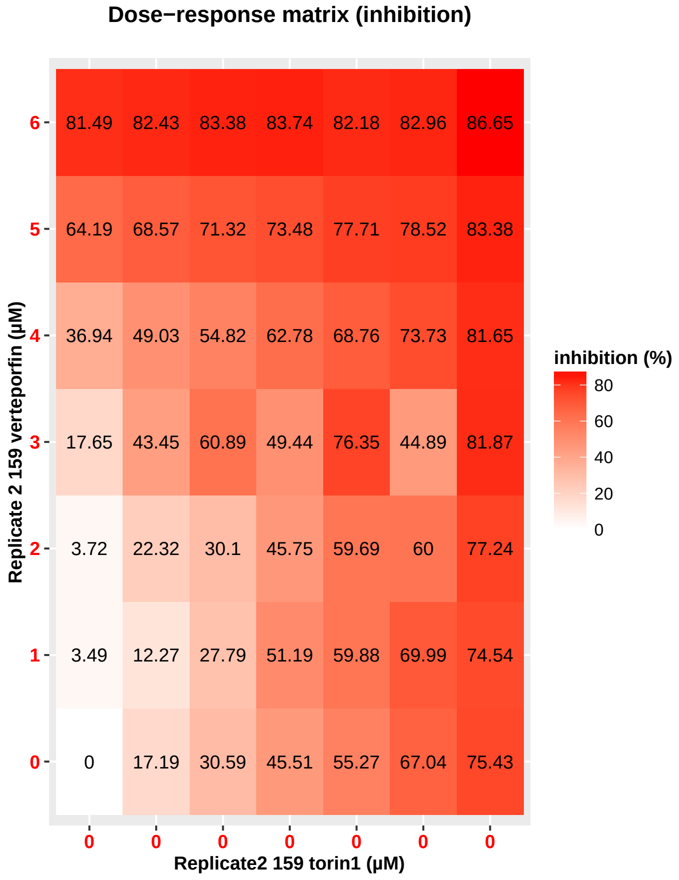

Replicate 3 159 torin1 & Replicate 3 159 verteporfin

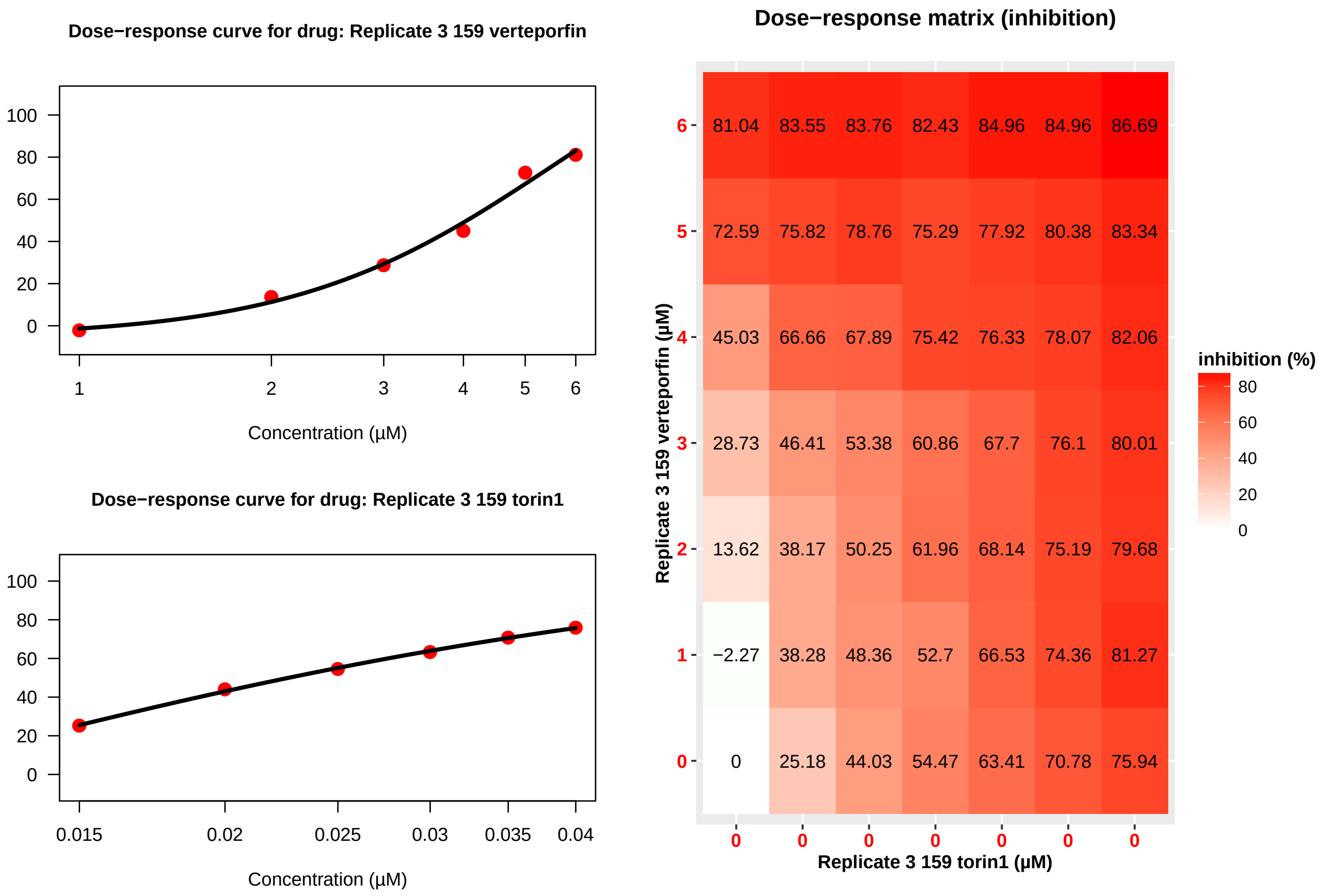

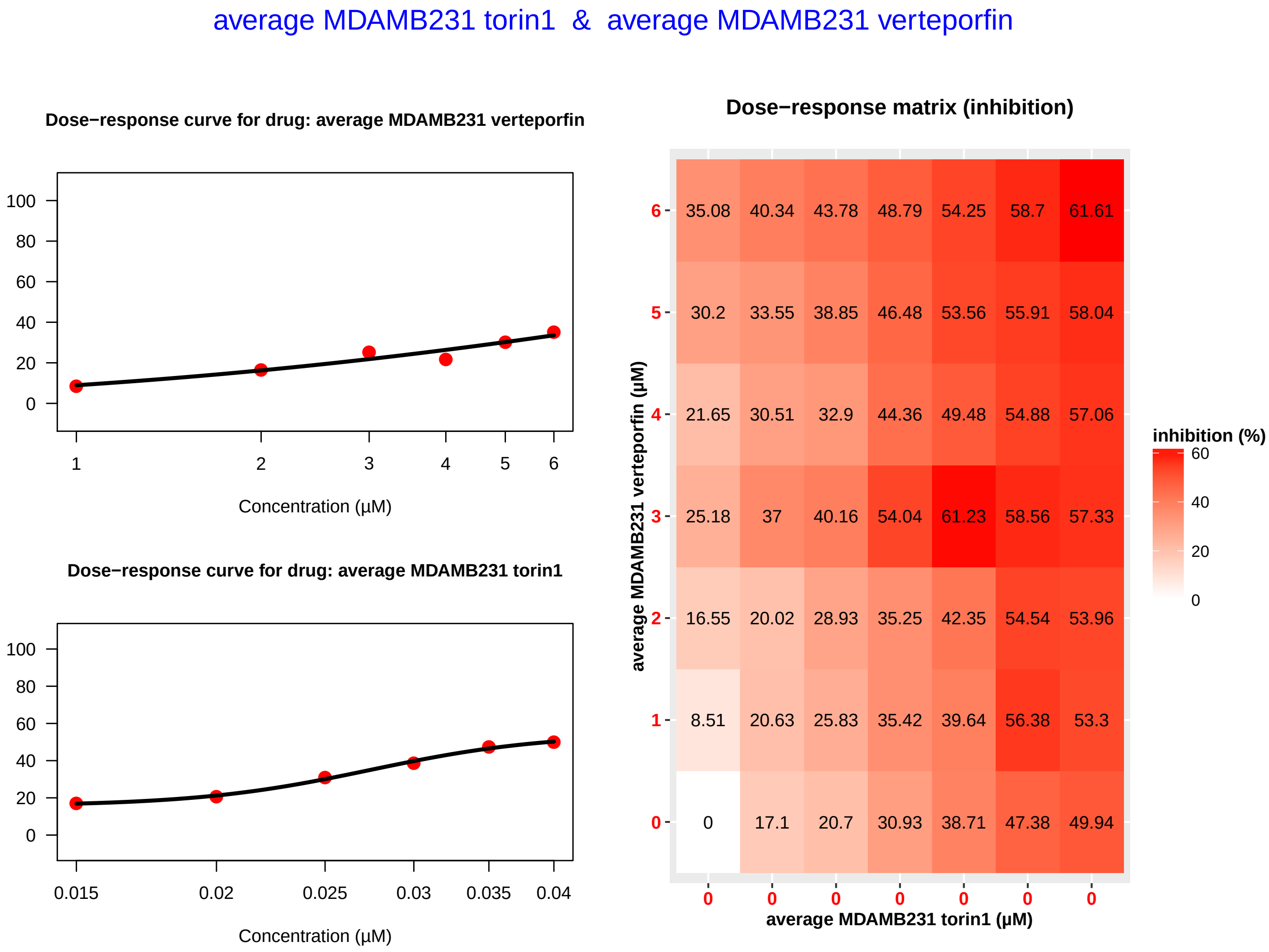
average MDAMB231 torin1 & average MDAMB231 verteporfin
Dose−response curve for drug: average MDAMB231 verteporfin
100
80
60
40
20
0
1
2
3
4
5
6
Concentration (µM)
Dose−response curve for drug: average MDAMB231 torin1
100
80
60
40
20
0
0.015
0.02
0.025
0.03
0.035
0.04
Concentration (µM)
Dose−response matrix (inhibition)
6 35.08 40.34 43.78 48.79 54.25 58.7 61.61
5 30.2 33.55 38.85 46.48 53.56 55.91 58.04
4 21.65 30.51 32.9 44.36 49.48 54.88 57.06
3 25.18 37 40.16 54.04 61.23 58.56 57.33
2 16.55 20.02 28.93 35.25 42.35 54.54 53.96
1 8.51 20.63 25.83 35.42 39.64 56.38 53.3
0 0 17.1 20.7 30.93 38.71 47.38 49.94
0 0 0 0 0 0 0
average MDAMB231 verteporfin (µM)
average MDAMB231 torin1 (µM)
inhibition (%)
60
40
20
0

Replicate 1 MDAMB231 torin1 & Replicate 1 MDAMB231 verteporfin

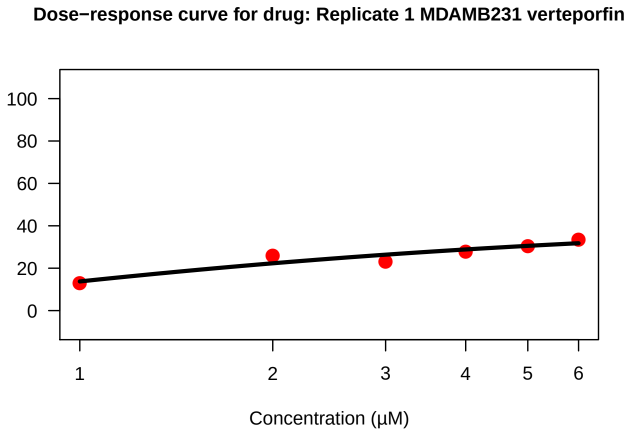

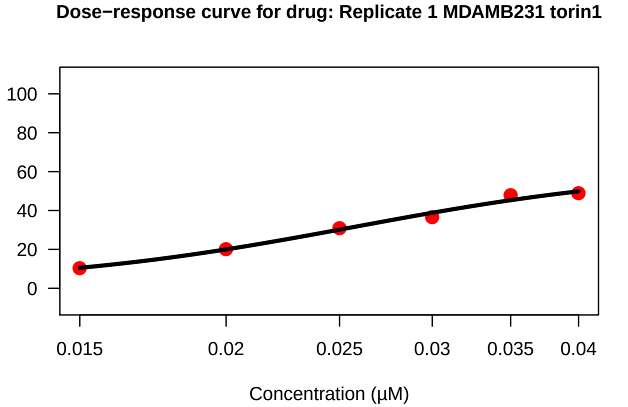

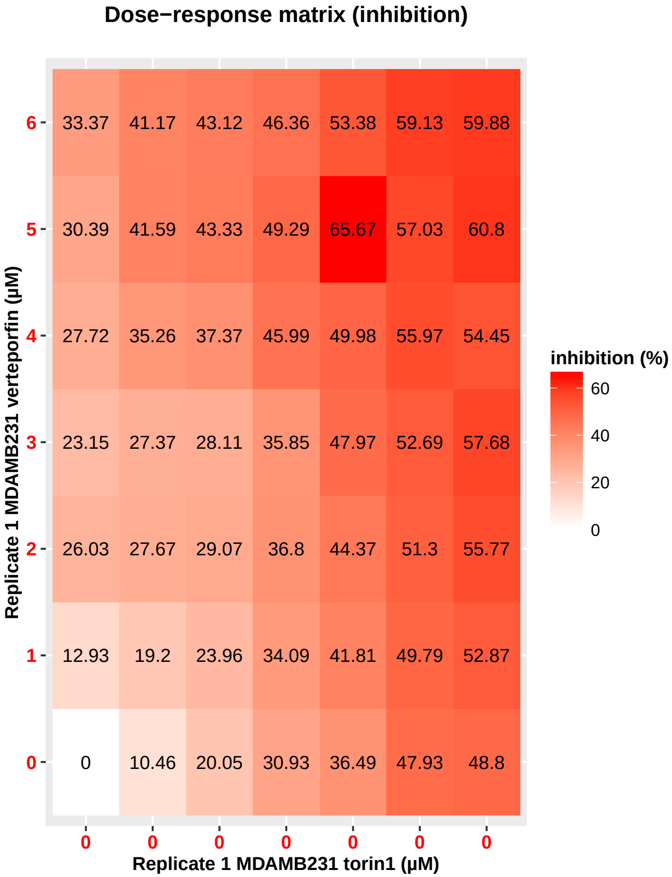

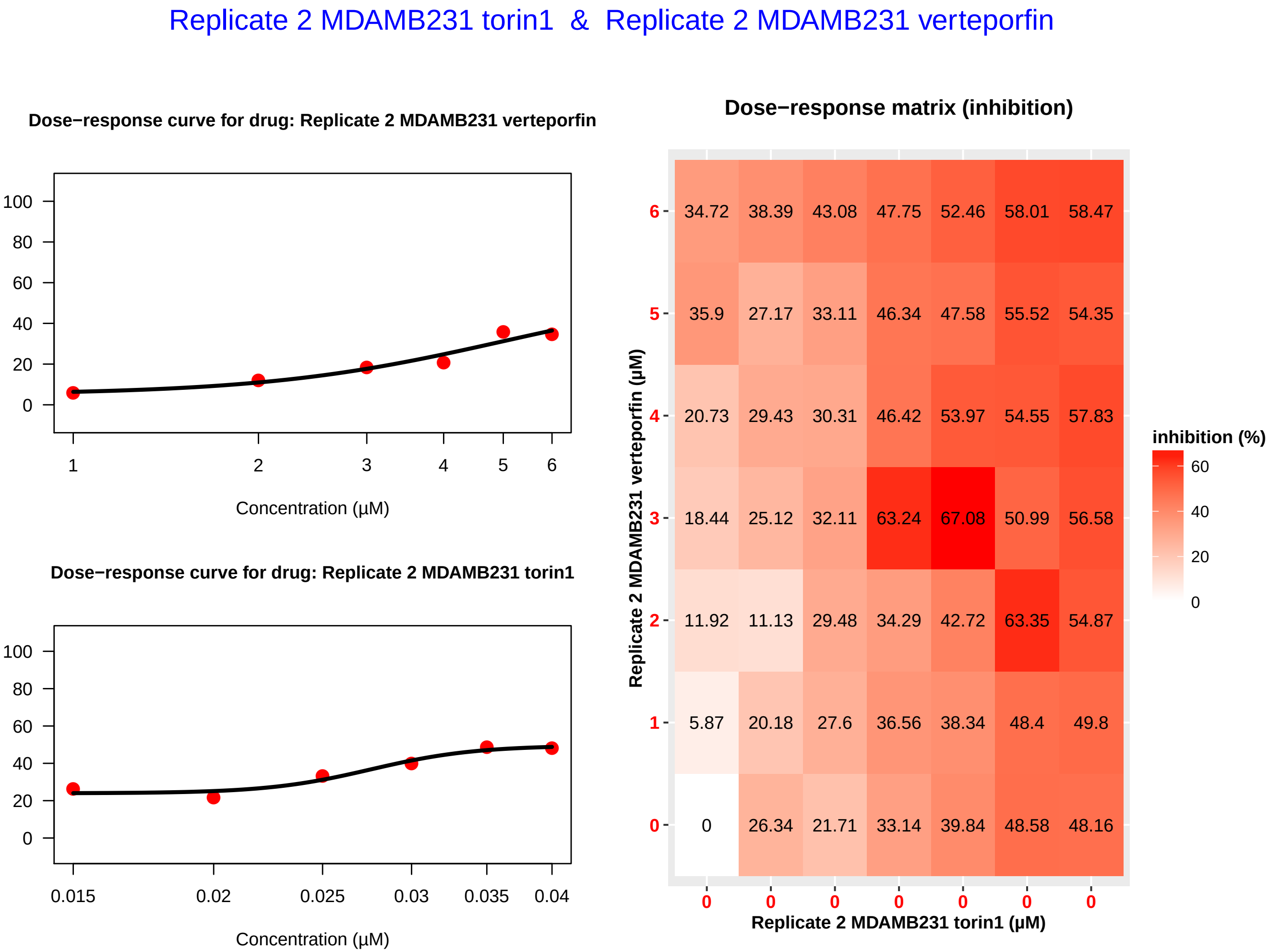
Replicate 2 MDAMB231 torin1 & Replicate 2 MDAMB231 verteporfin
Dose−response curve for drug: Replicate 2 MDAMB231 verteporfin
100
80
60
40
20
0
1
2
3
4
5
6
Concentration (μM)
Dose−response curve for drug: Replicate 2 MDAMB231 torin1
100
80
60
40
20
0
0.015
0.02
0.025
0.03
0.035
0.04
Concentration (μM)
Dose−response matrix (inhibition)
Replicate 2 MDAMB231 verteporfin (μM)
6 34.72 38.39 43.08 47.75 52.46 58.01 58.47
5 35.9 27.17 33.11 46.34 47.58 55.52 54.35
4 20.73 29.43 30.31 46.42 53.97 54.55 57.83
3 18.44 25.12 32.11 63.24 67.08 50.99 56.58
2 11.92 11.13 29.48 34.29 42.72 63.35 54.87
1 5.87 20.18 27.6 36.56 38.34 48.4 49.8
0 0 26.34 21.71 33.14 39.84 48.58 48.16
0 0 0 0 0 0 0
Replicate 2 MDAMB231 torin1 (μM)
inhibition (%)
60
40
20
0

Replicate 3 MDAMB231 torin1 & Replicate 3 MDAMB231 verteporfin

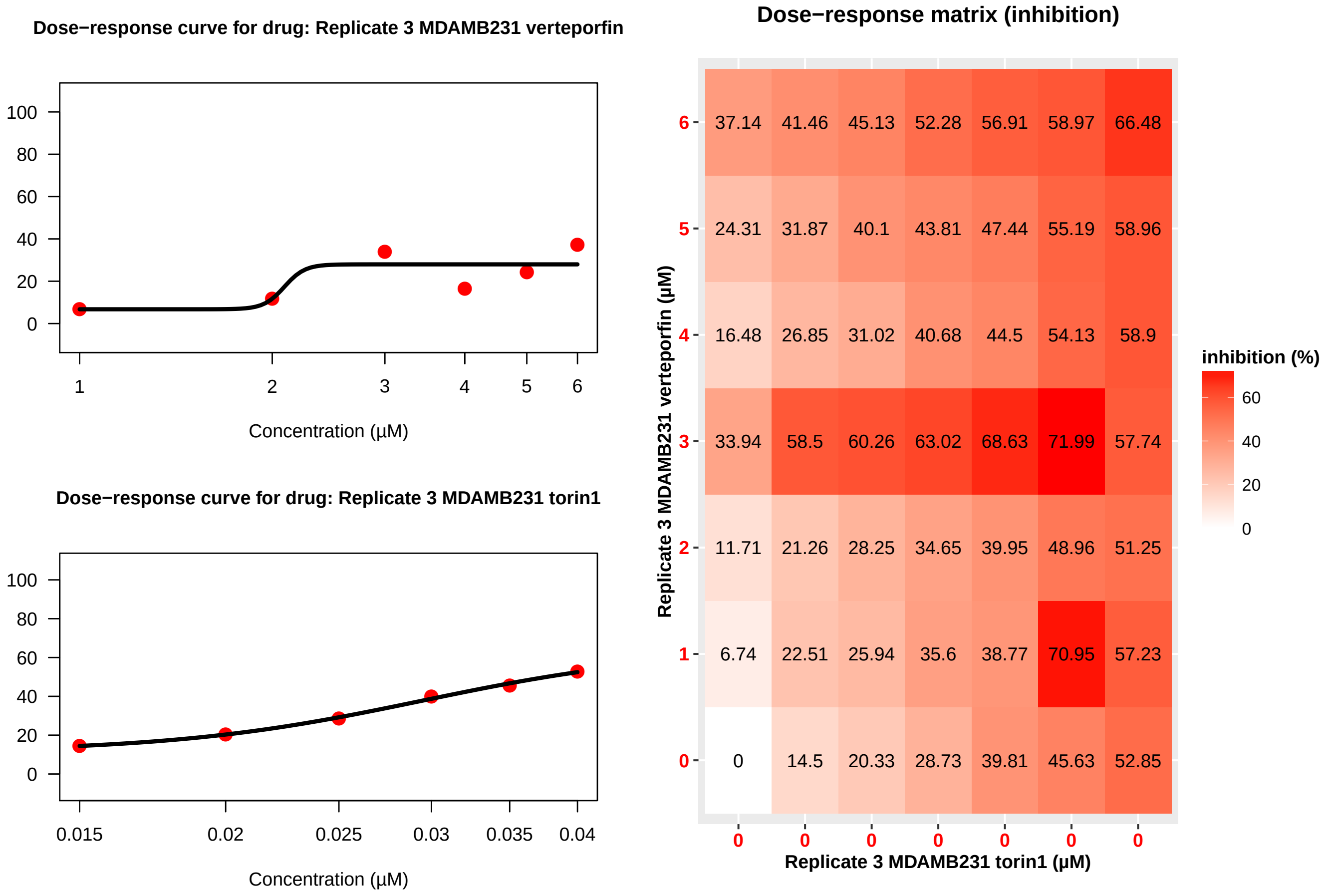

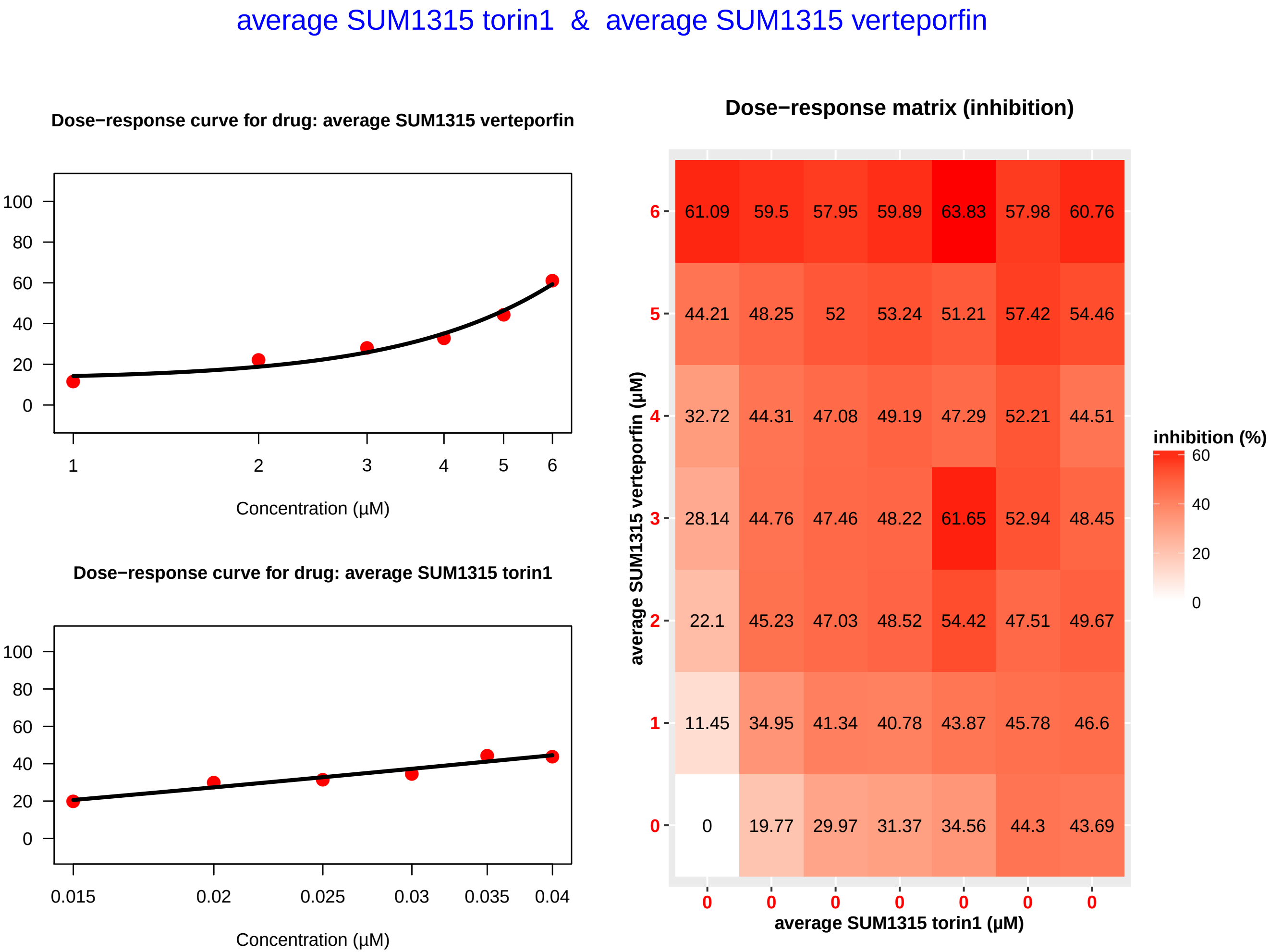
average SUM1315 torin1 & average SUM1315 verteporfin
Dose−response curve for drug: average SUM1315 verteporfin
100
80
60
40
20
0
1
2
3
4
5
6
Concentration (µM)
Dose−response curve for drug: average SUM1315 torin1
100
80
60
40
20
0
0.015
0.02
0.025
0.03
0.035
0.04
Concentration (µM)
Dose−response matrix (inhibition)
6 61.09 59.5 57.95 59.89 63.83 57.98 60.76
5 44.21 48.25 52 53.24 51.21 57.42 54.46
4 32.72 44.31 47.08 49.19 47.29 52.21 44.51
3 28.14 44.76 47.46 48.22 61.65 52.94 48.45
2 22.1 45.23 47.03 48.52 54.42 47.51 49.67
1 11.45 34.95 41.34 40.78 43.87 45.78 46.6
0 0 19.77 29.97 31.37 34.56 44.3 43.69
0 0 0 0 0 0 0
average SUM1315 verteporfin (µM)
average SUM1315 torin1 (µM)
inhibition (%)
60
40
20
0

Replicate 1 SUM1315 torin1 & Replicate 1 SUM1315 verteporfin

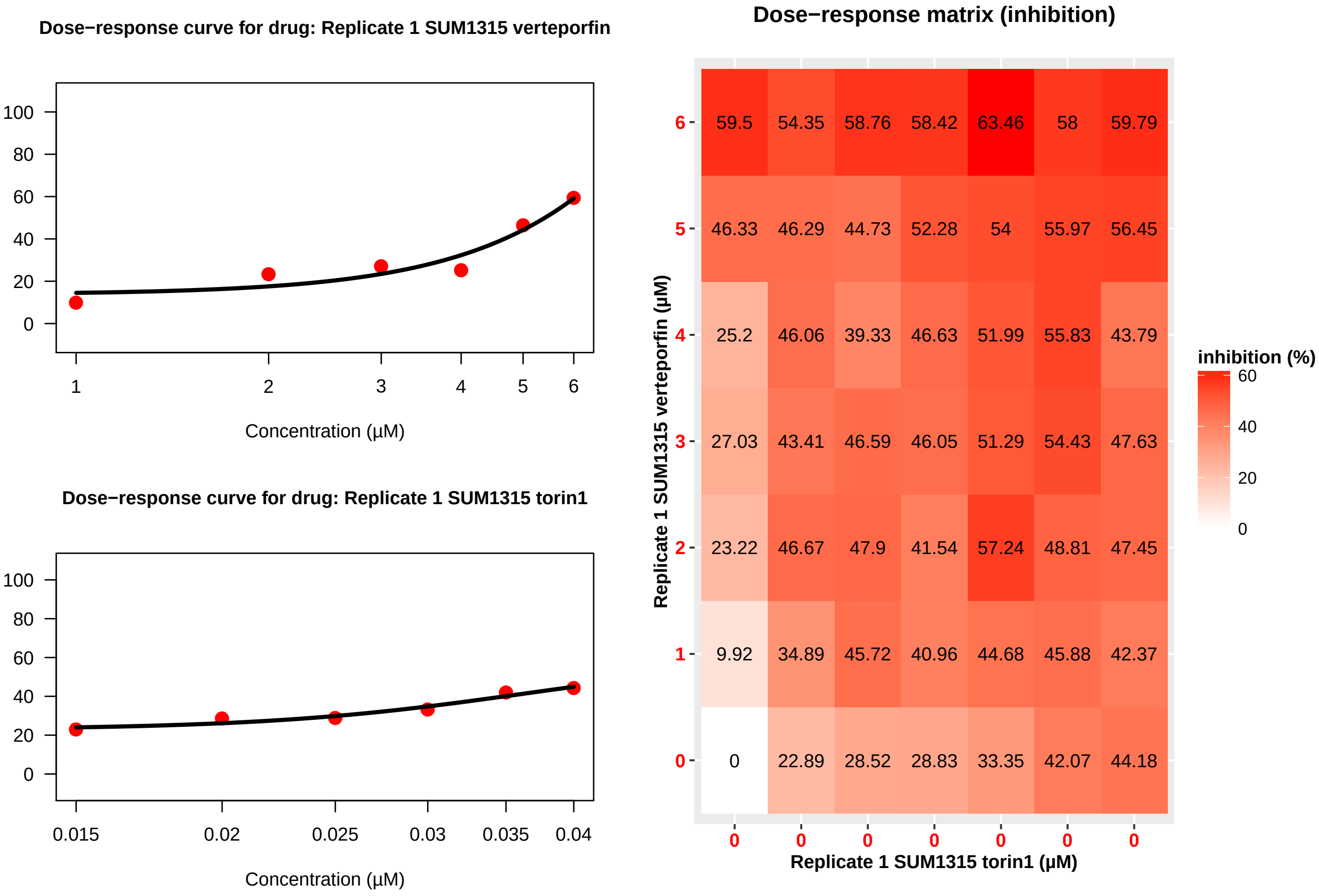

Replicate 2 SUM1315 torin1 & Replicate 2 SUM1315 verteporfin

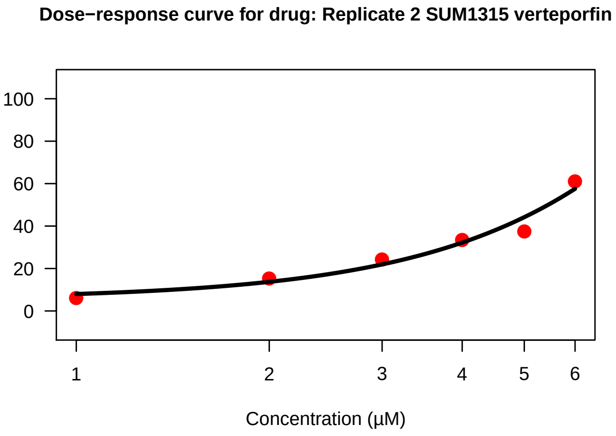

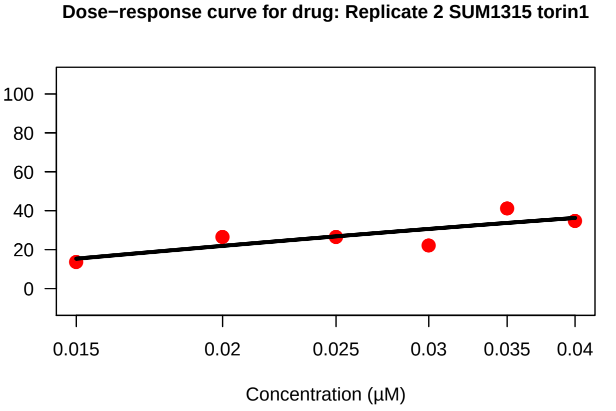

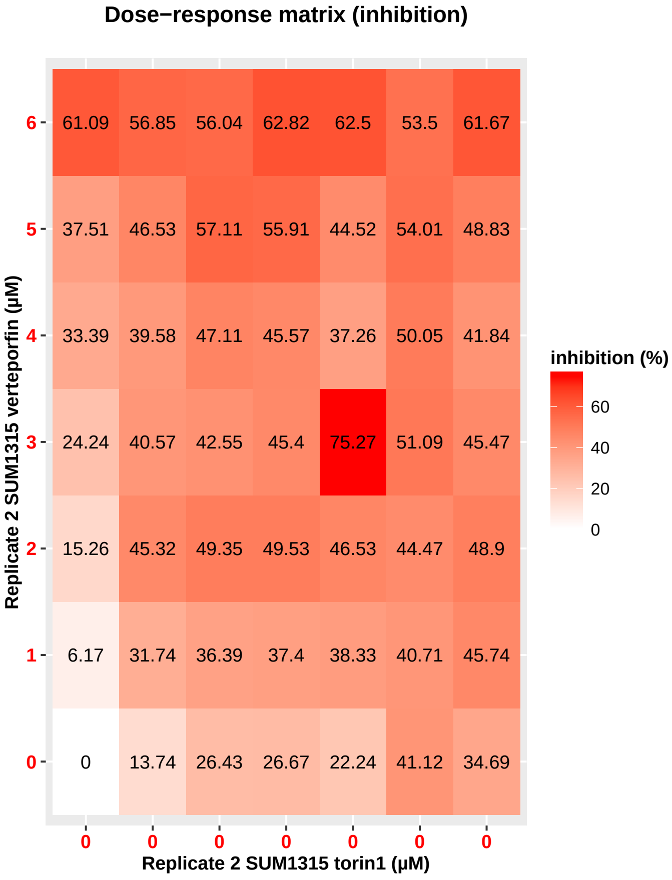

Replicate 3 SUM1315 torin1 & Replicate 3 SUM1315 verteporfin

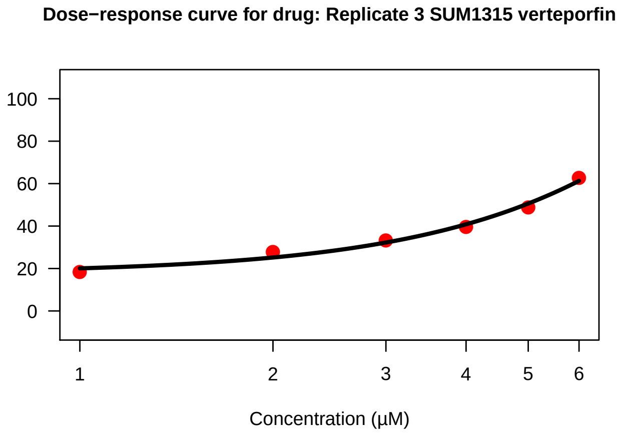

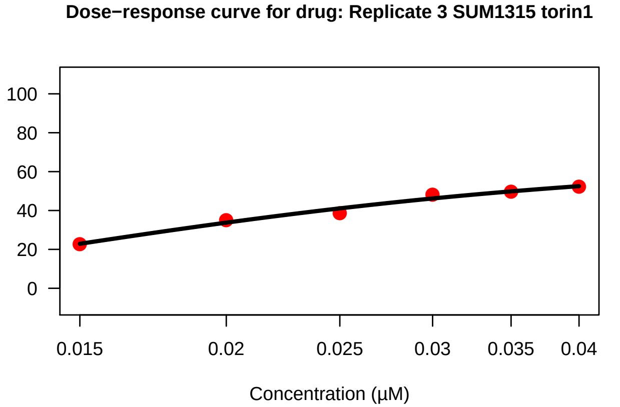

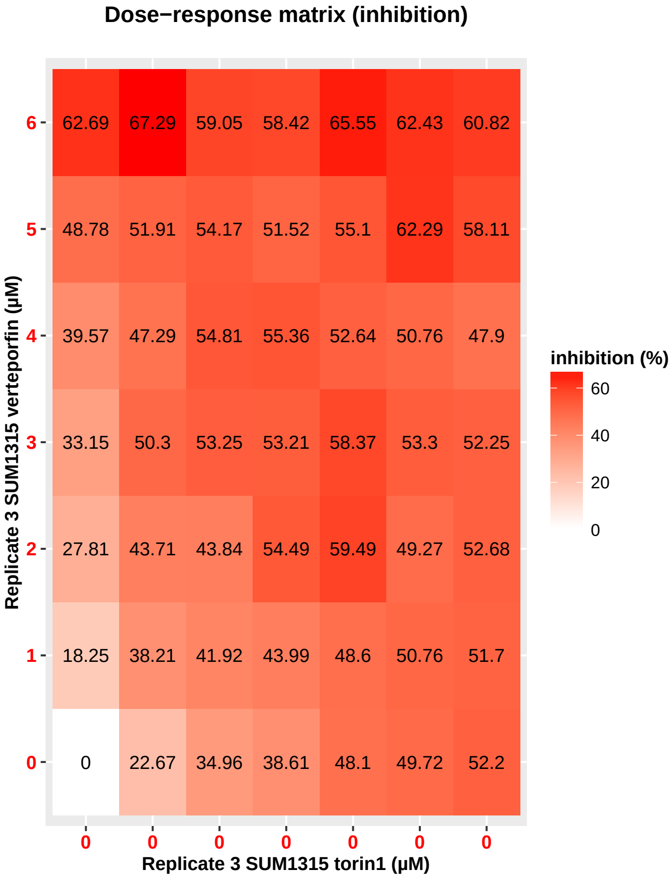

average SUM149 torin1 & average SUM149 verteporfin

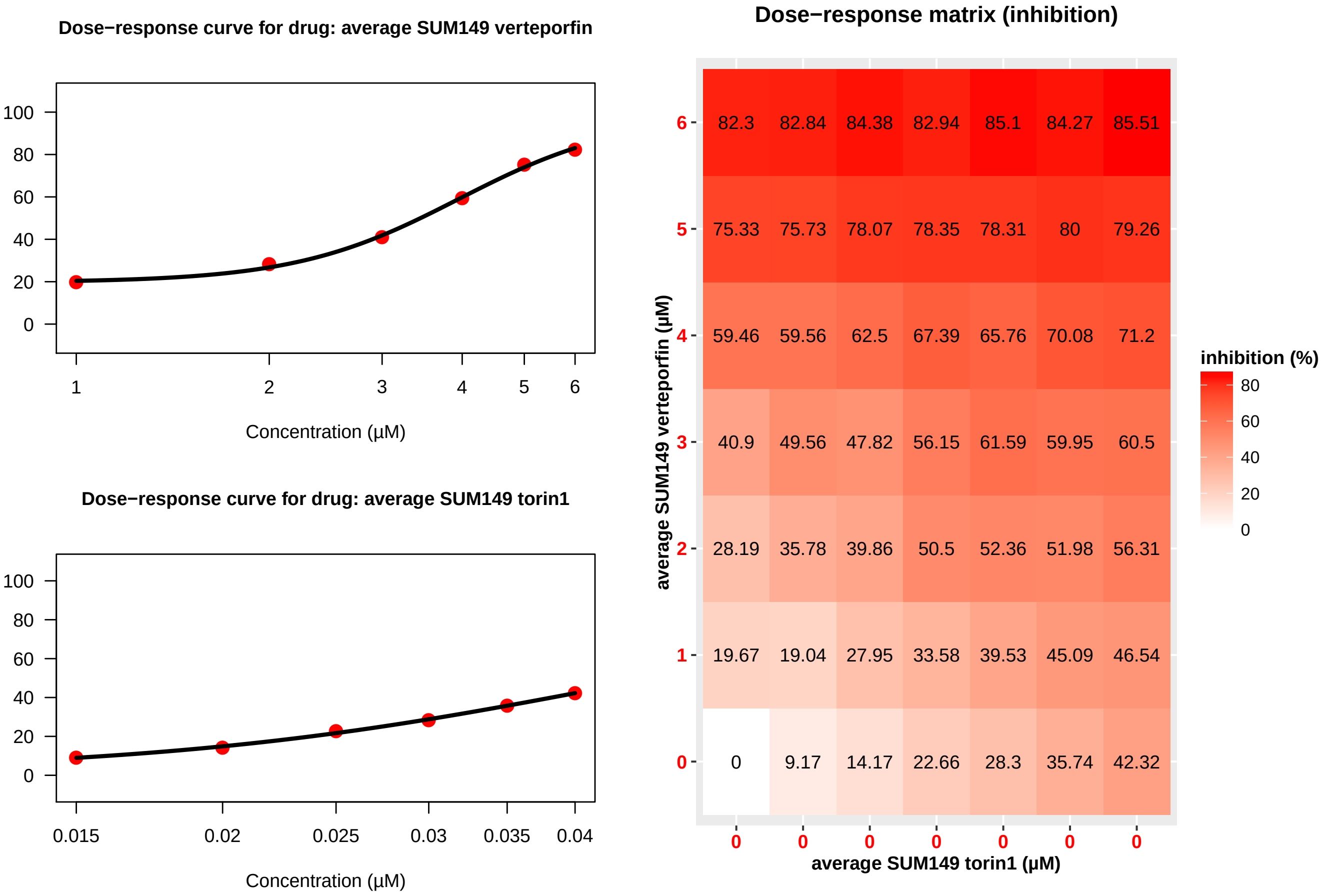

Replicate 1 SUM149 torin1 & Replicate 1 SUM149 verteporfin

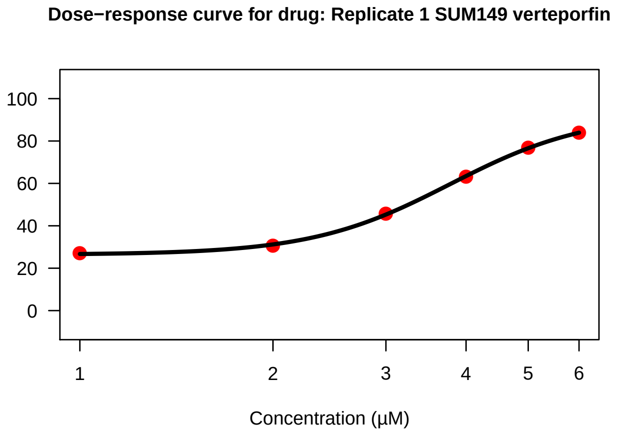

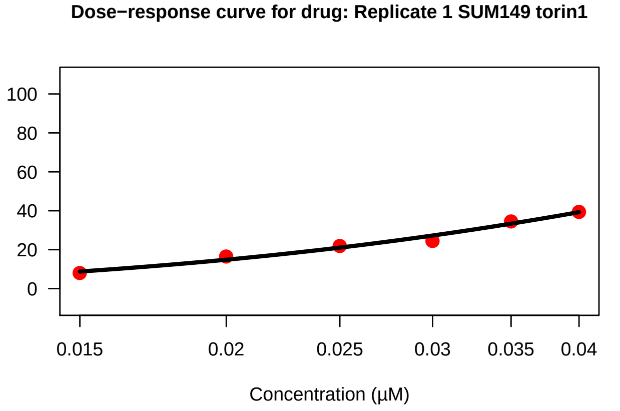

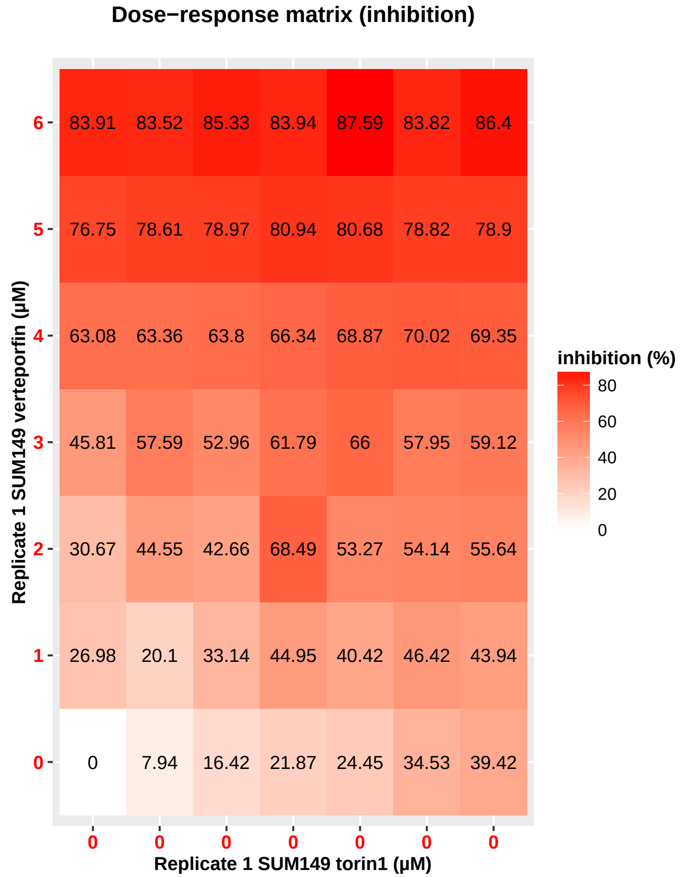

Replicate 2 SUM149 torin1 & Replicate 2 SUM149 verteporfin

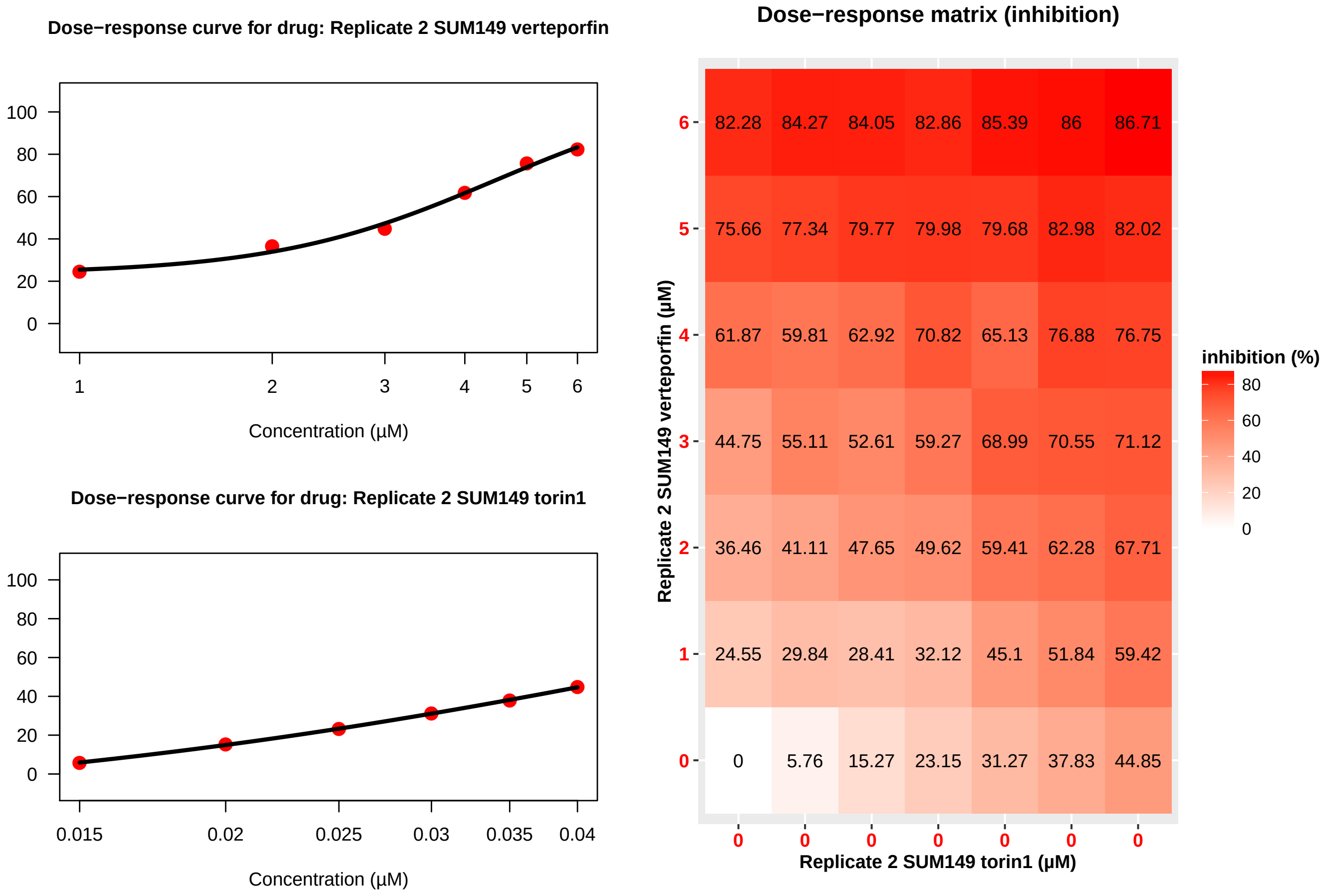

Replicate 3 SUM149 torin1 & Replicate 3 SUM149 verteporfin

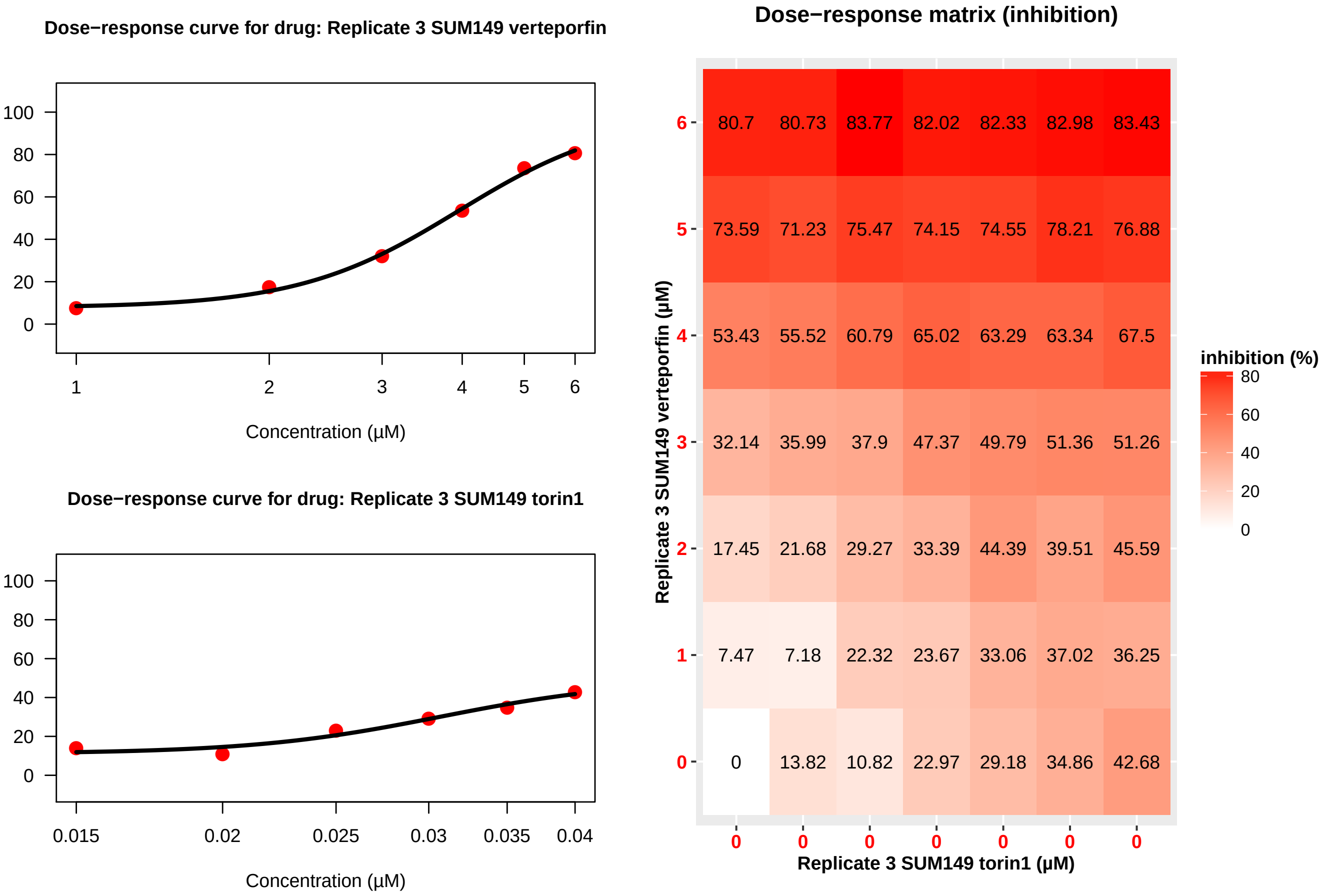

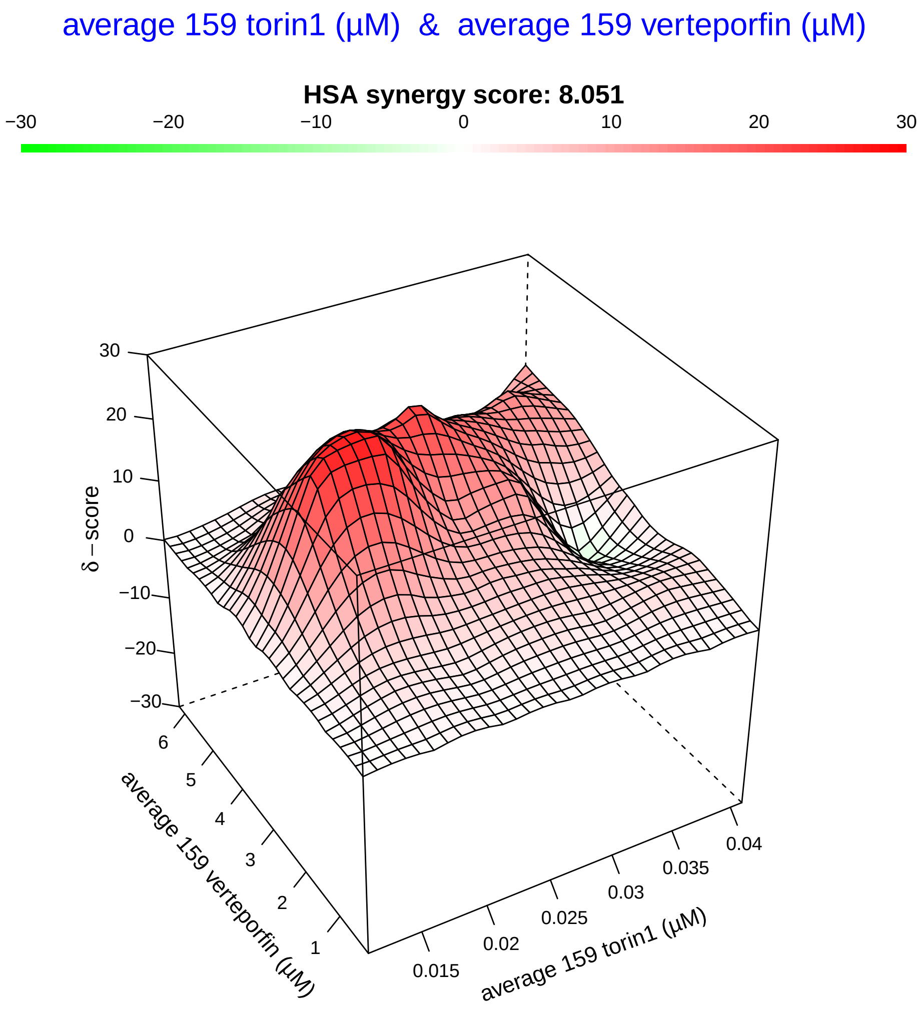

average 159 torin1 (µM) & average 159 verteporfin (µM)
HSA synergy score: 8.051
−30
−20
−10
0
10
20
30
δ − score
average 159 verteporfin (µM)
average 159 torin1 (µM)
1
2
3
4
5
6
0.015
0.02
0.025
0.03
0.035
0.04

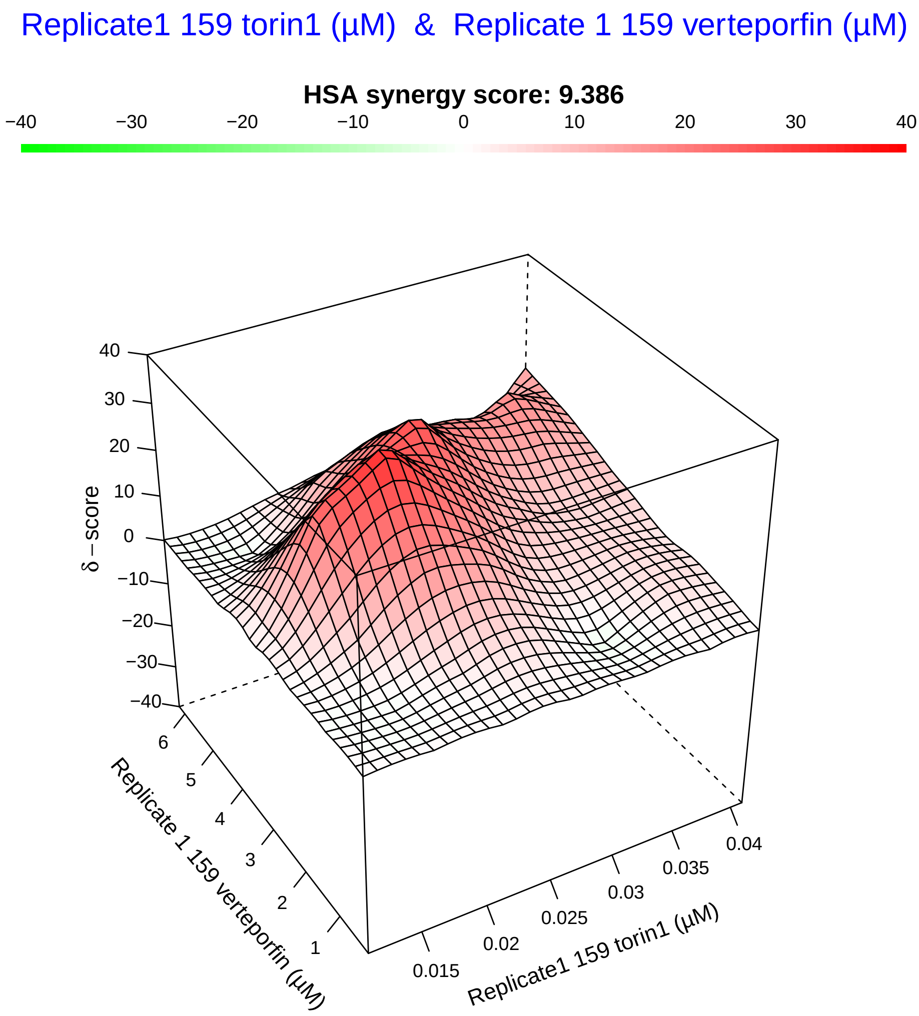

Replicate1 159 torin1 (µM) & Replicate 1 159 verteporfin (µM)
HSA synergy score: 9.386
−40
−30
−20
−10
0
10
20
30
40
δ − score
Replicate 1 159 verteporfin (µM)
1
2
3
4
5
6
Replicate1 159 torin1 (µM)
0.015
0.02
0.025
0.03
0.035
0.04

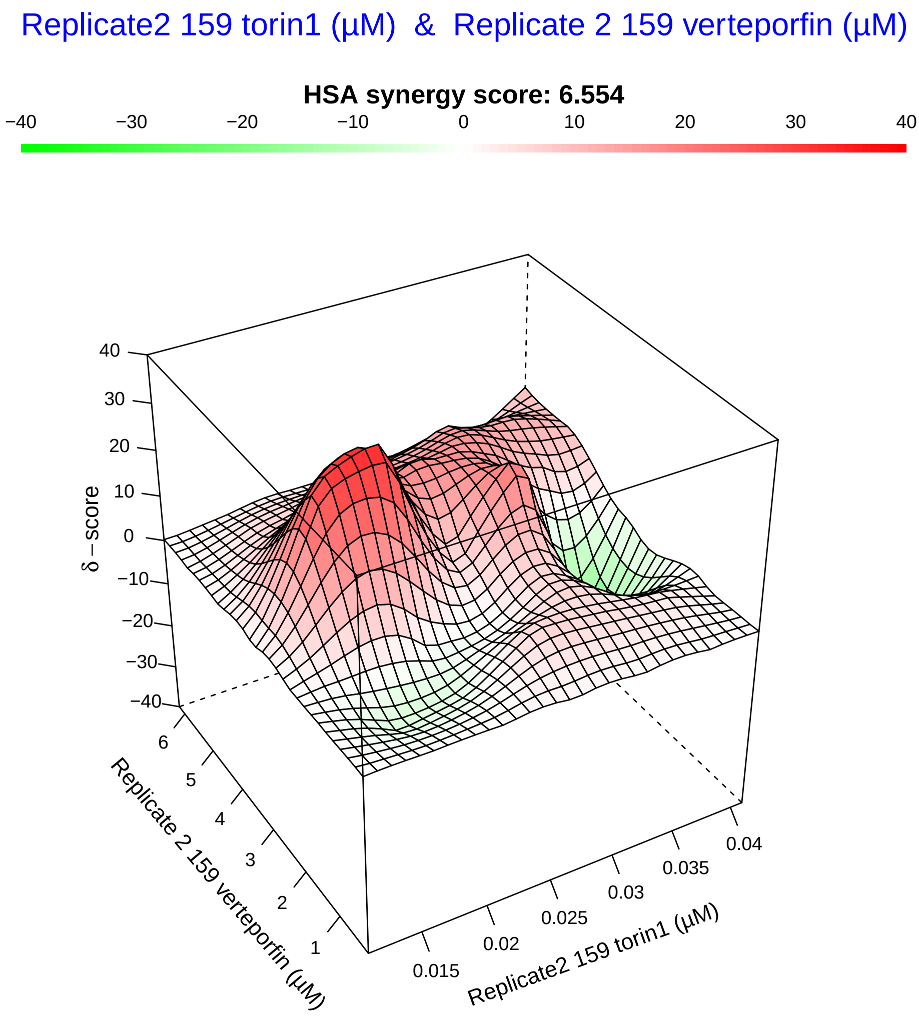
Replicate2 159 torin1 (µM) & Replicate 2 159 verteporfin (µM)
HSA synergy score: 6.554
−40
−30
−20
−10
0
10
20
30
40
δ − score
Replicate 2 159 verteporfin (µM)
1
2
3
4
5
6
Replicate2 159 torin1 (µM)
0.015
0.02
0.025
0.03
0.035
0.04

Replicate 3 159 torin1 (µM) & Replicate 3 159 verteporfin (µM)

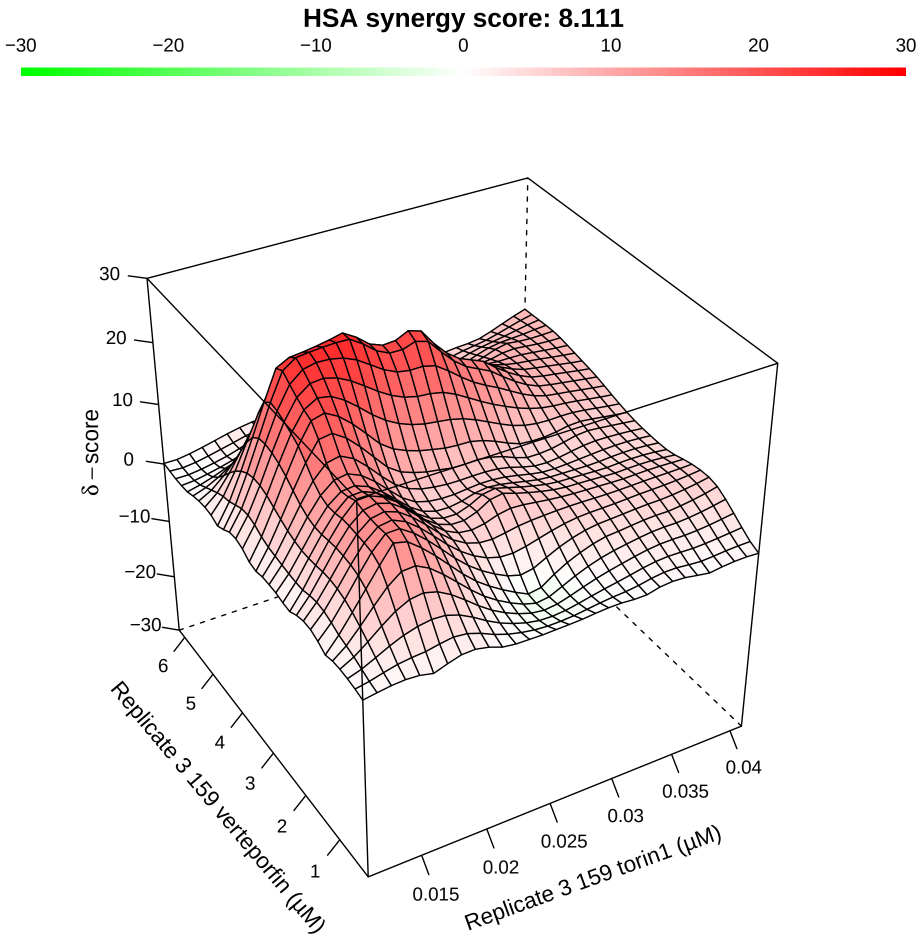

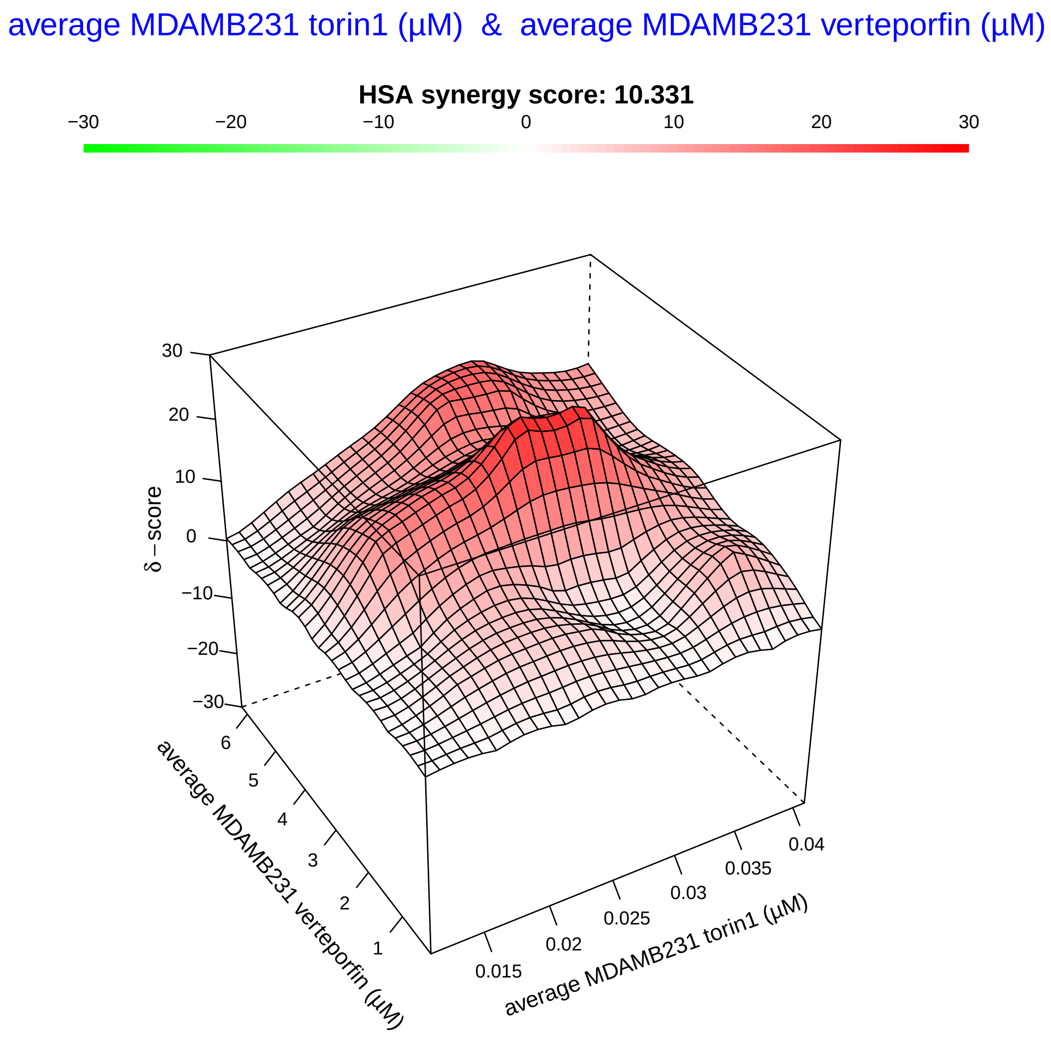
average MDAMB231 torin1 (µM) & average MDAMB231 verteporfin (µM)
HSA synergy score: 10.331
−30
−20
−10
0
10
20
30
δ − score
30
20
10
0
−10
−20
−30
average MDAMB231 verteporfin (µM)
6
5
4
3
2
1
average MDAMB231 torin1 (µM)
0.015
0.02
0.025
0.03
0.035
0.04

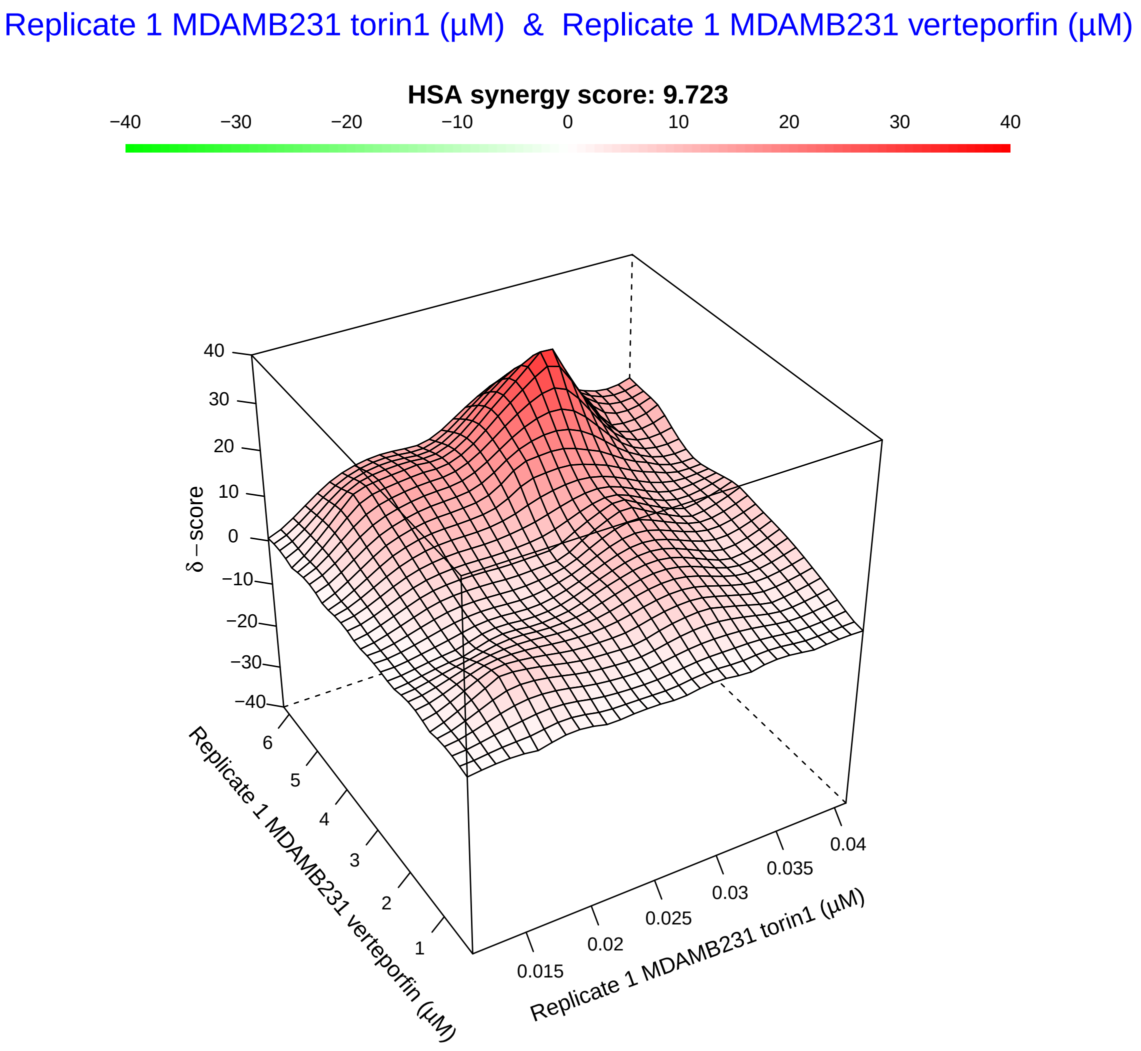
Replicate 1 MDAMB231 torin1 (µM) & Replicate 1 MDAMB231 verteporfin (µM)
HSA synergy score: 9.723
−40
−30
−20
−10
0
10
20
30
40
δ − score
Replicate 1 MDAMB231 verteporfin (µM)
1
2
3
4
5
6
Replicate 1 MDAMB231 torin1 (µM)
0.015
0.02
0.025
0.03
0.035
0.04

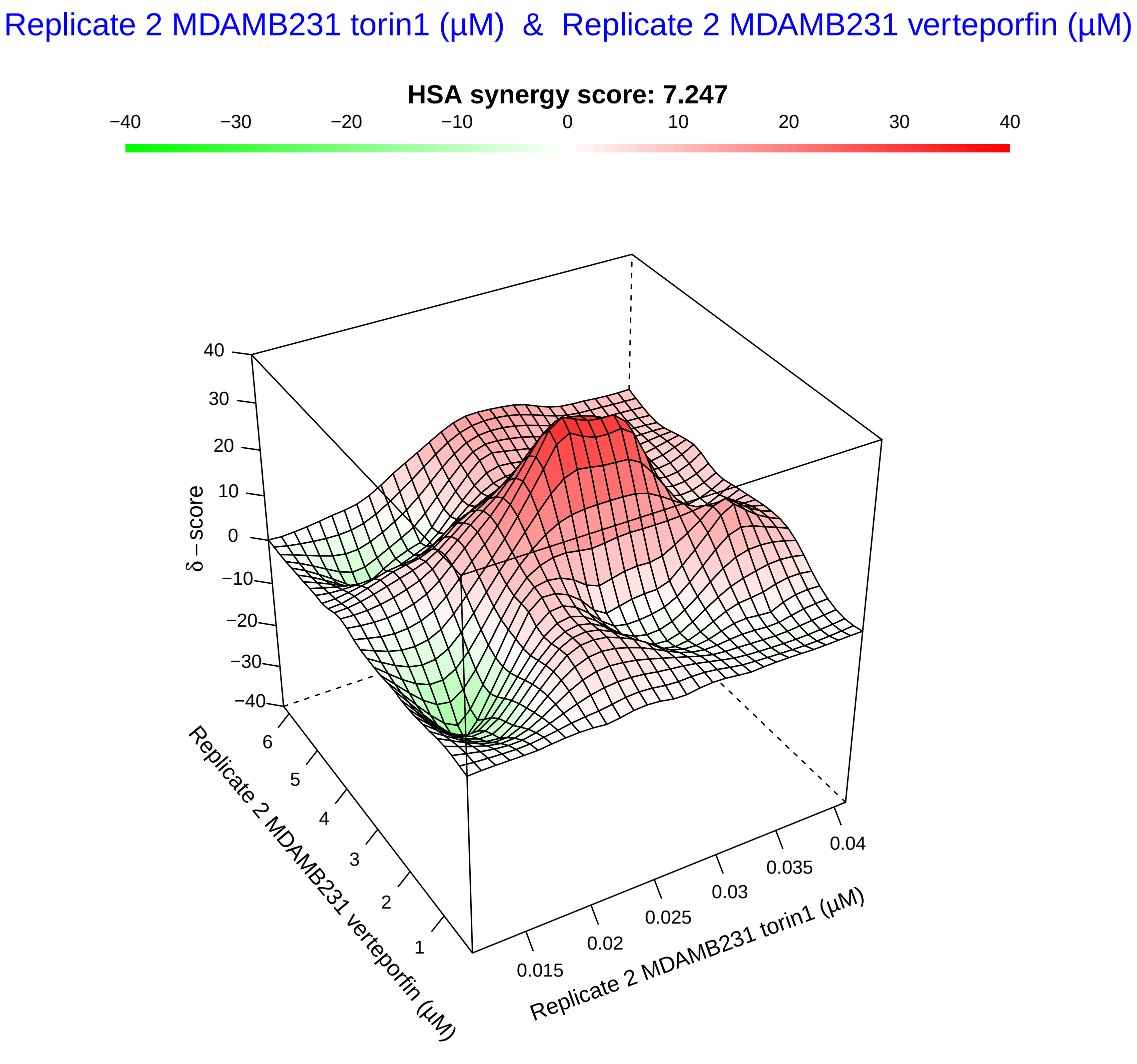
Replicate 2 MDAMB231 torin1 (µM) & Replicate 2 MDAMB231 verteporfin (µM)
HSA synergy score: 7.247
−40
−30
−20
−10
0
10
20
30
40
δ − score
Replicate 2 MDAMB231 verteporfin (µM)
1
2
3
4
5
6
Replicate 2 MDAMB231 torin1 (µM)
0.015
0.02
0.025
0.03
0.035
0.04

Replicate 3 MDAMB231 torin1 (µM) & Replicate 3 MDAMB231 verteporfin (µM)

HSA synergy score: 12.119

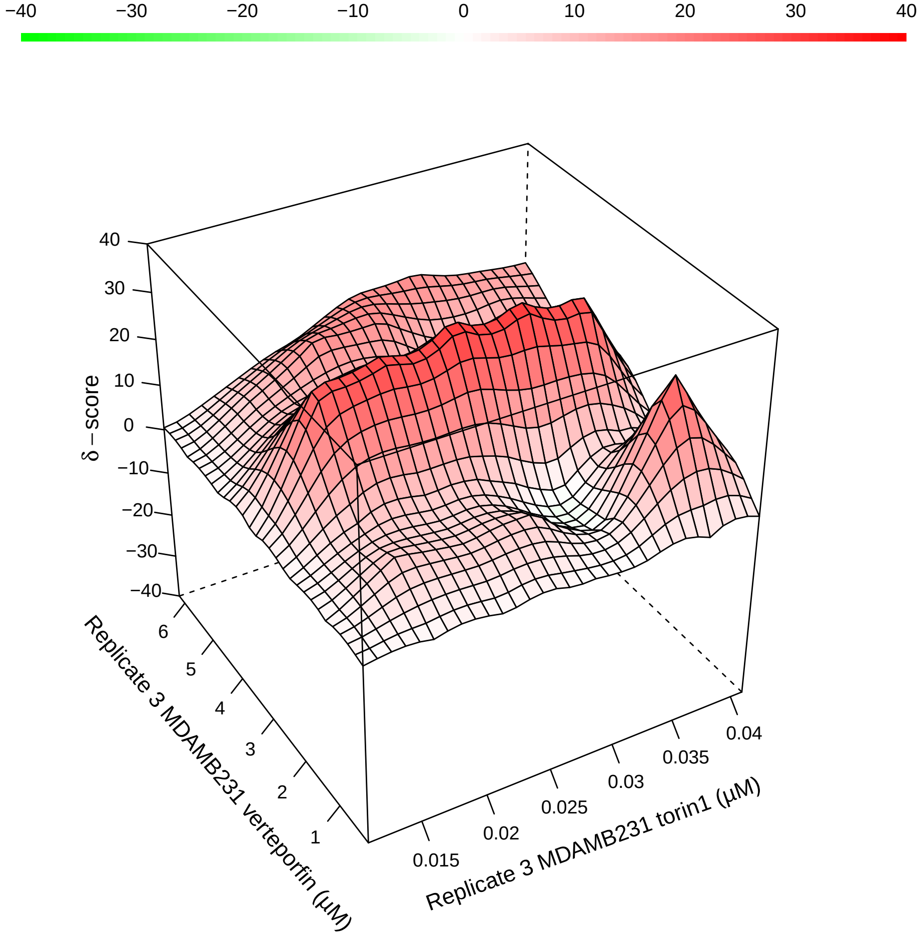

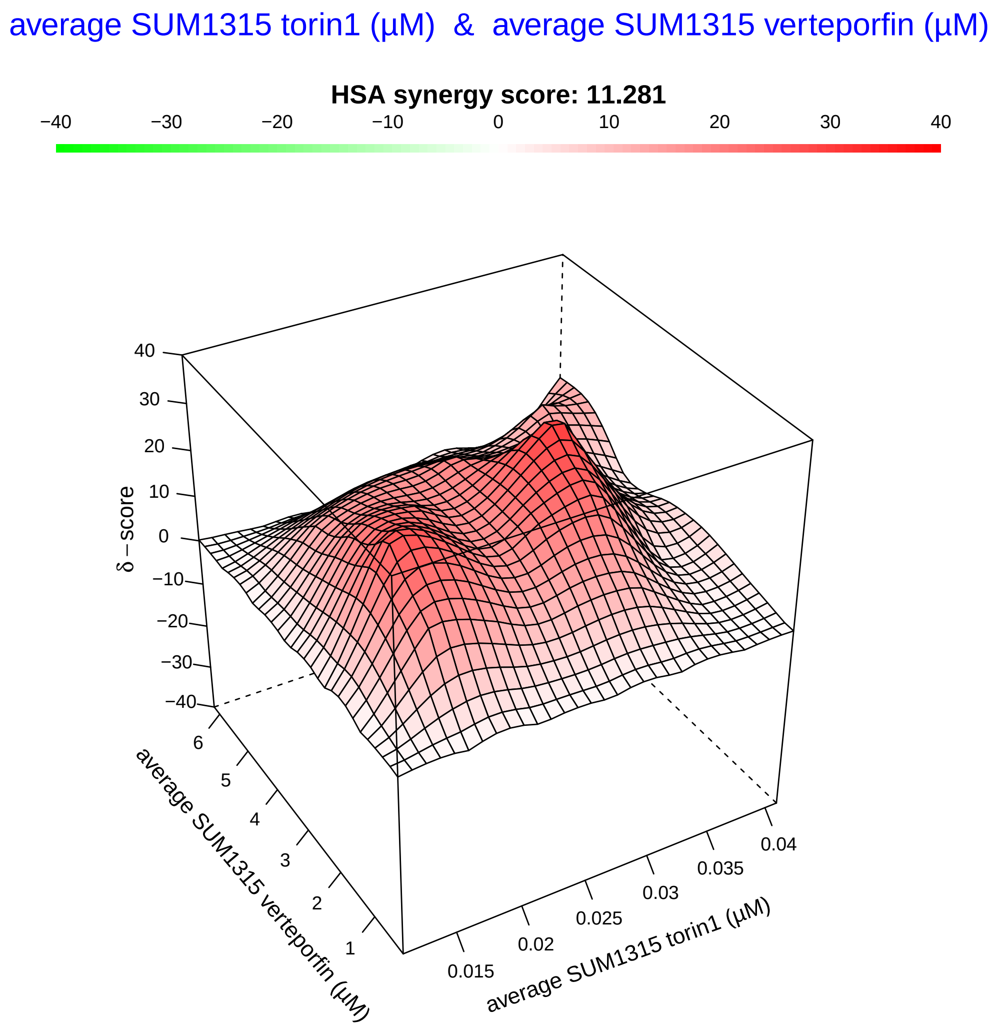
average SUM1315 torin1 (µM) & average SUM1315 verteporfin (µM)
HSA synergy score: 11.281
−40
−30
−20
−10
0
10
20
30
40
δ − score
average SUM1315 verteporfin (µM)
1
2
3
4
5
6
average SUM1315 torin1 (µM)
0.015
0.02
0.025
0.03
0.035
0.04

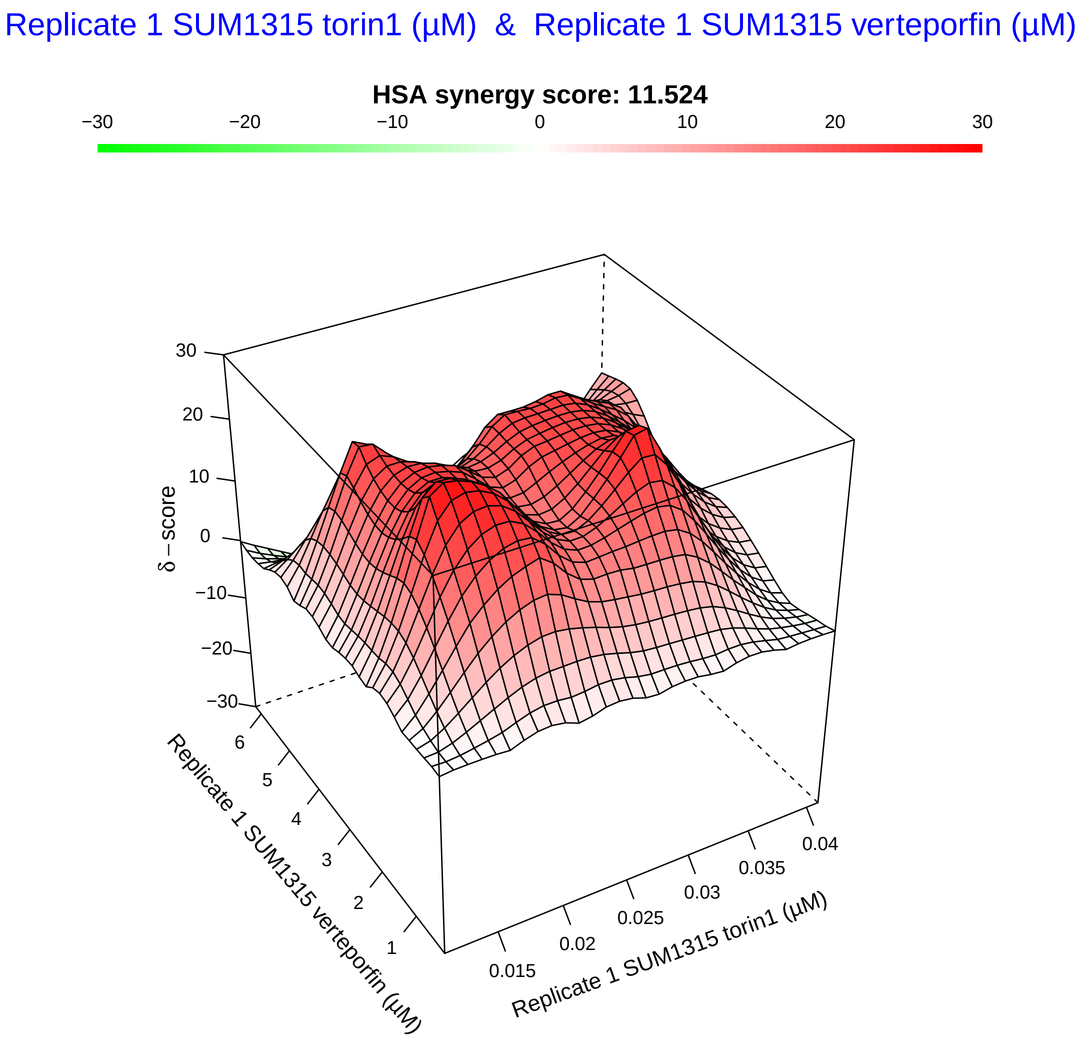
Replicate 1 SUM1315 torin1 (µM) & Replicate 1 SUM1315 verteporfin (µM)
HSA synergy score: 11.524
−30
−20
−10
0
10
20
30
δ − score
Replicate 1 SUM1315 verteporfin (µM)
1
2
3
4
5
6
Replicate 1 SUM1315 torin1 (µM)
0.015
0.02
0.025
0.03
0.035
0.04

Replicate 2 SUM1315 torin1 (µM) & Replicate 2 SUM1315 verteporfin (µM)

HSA synergy score: 13.485

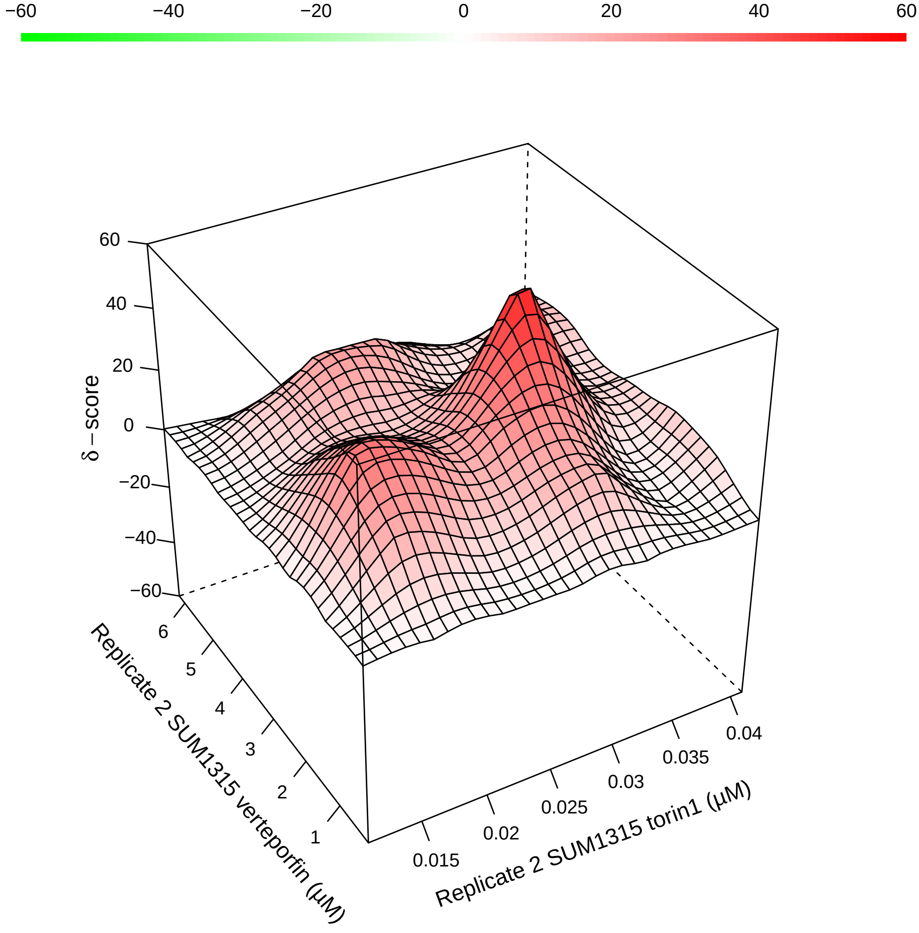

Replicate 3 SUM1315 torin1 (µM) & Replicate 3 SUM1315 verteporfin (µM)

HSA synergy score: 7.646

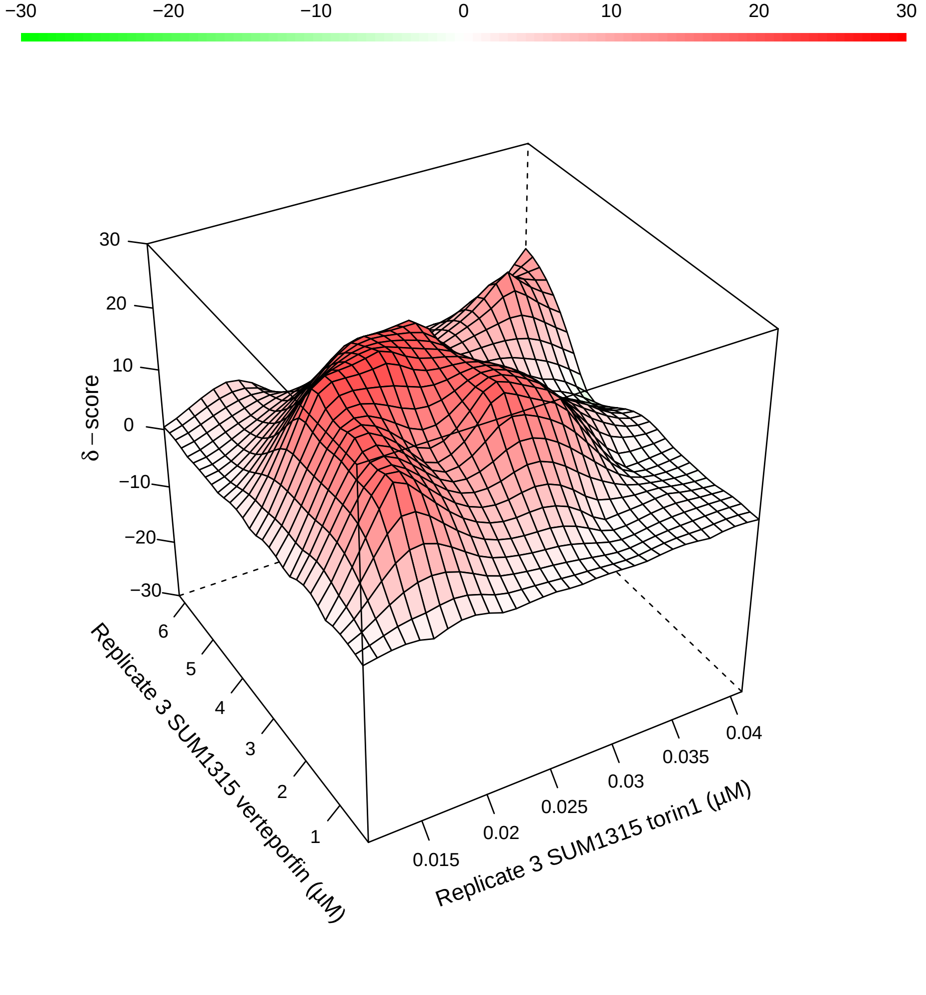

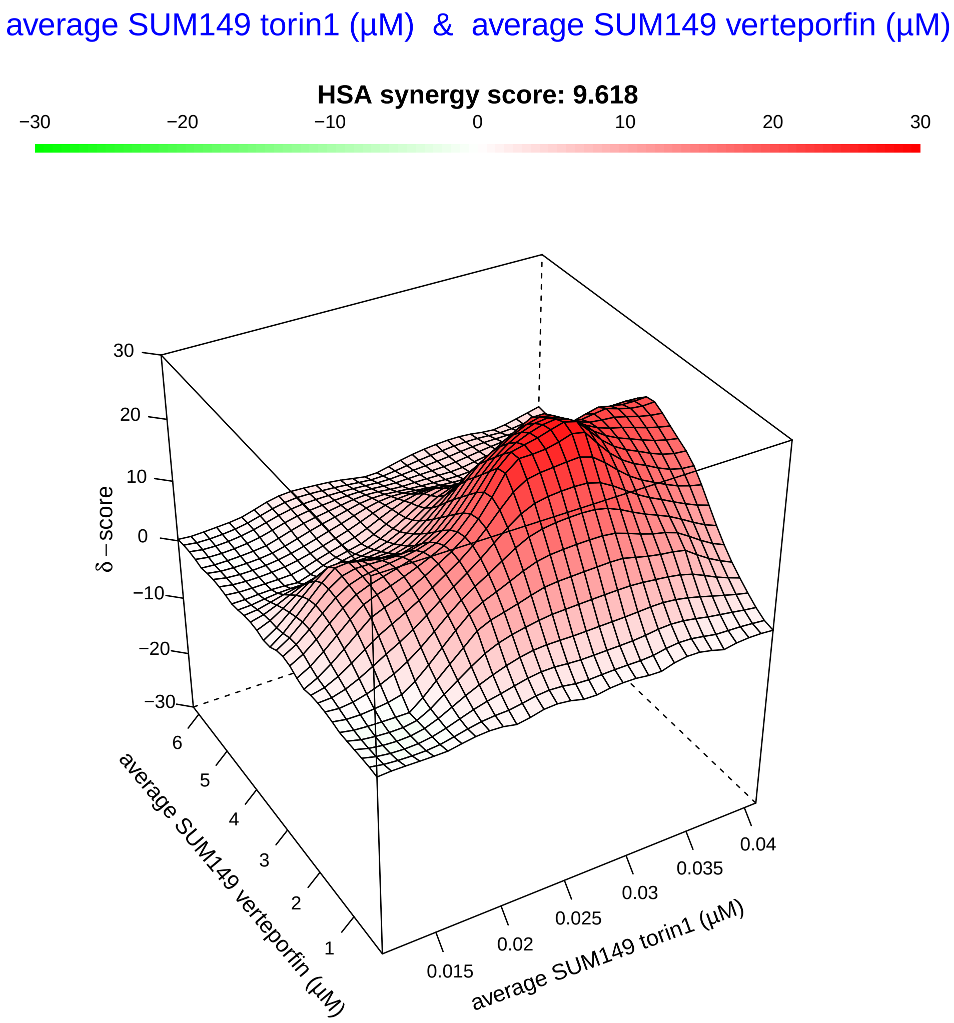
average SUM149 torin1 (µM) & average SUM149 verteporfin (µM)
HSA synergy score: 9.618
−30
−20
−10
0
10
20
30
δ − score
average SUM149 verteporfin (µM)
average SUM149 torin1 (µM)
0.015
0.02
0.025
0.03
0.035
0.04
1
2
3
4
5
6

Replicate 1 SUM149 torin1 (µM) & Replicate 1 SUM149 verteporfin (µM)

HSA synergy score: 9.893

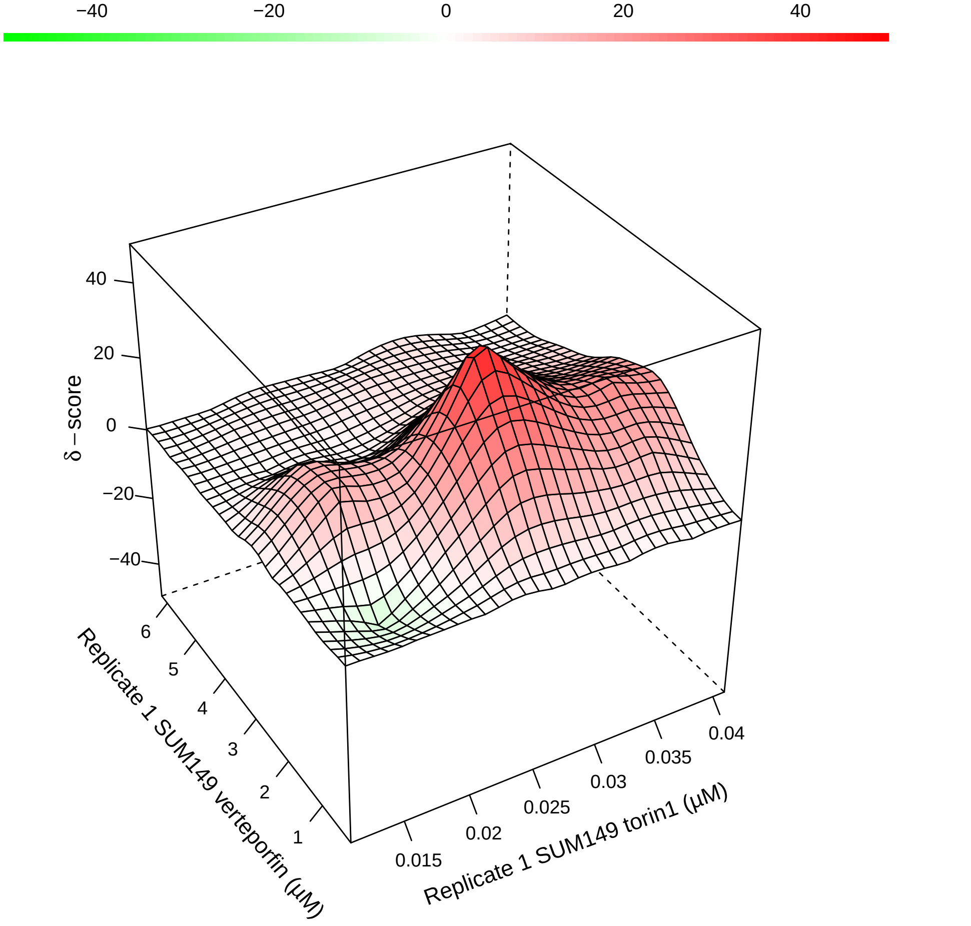

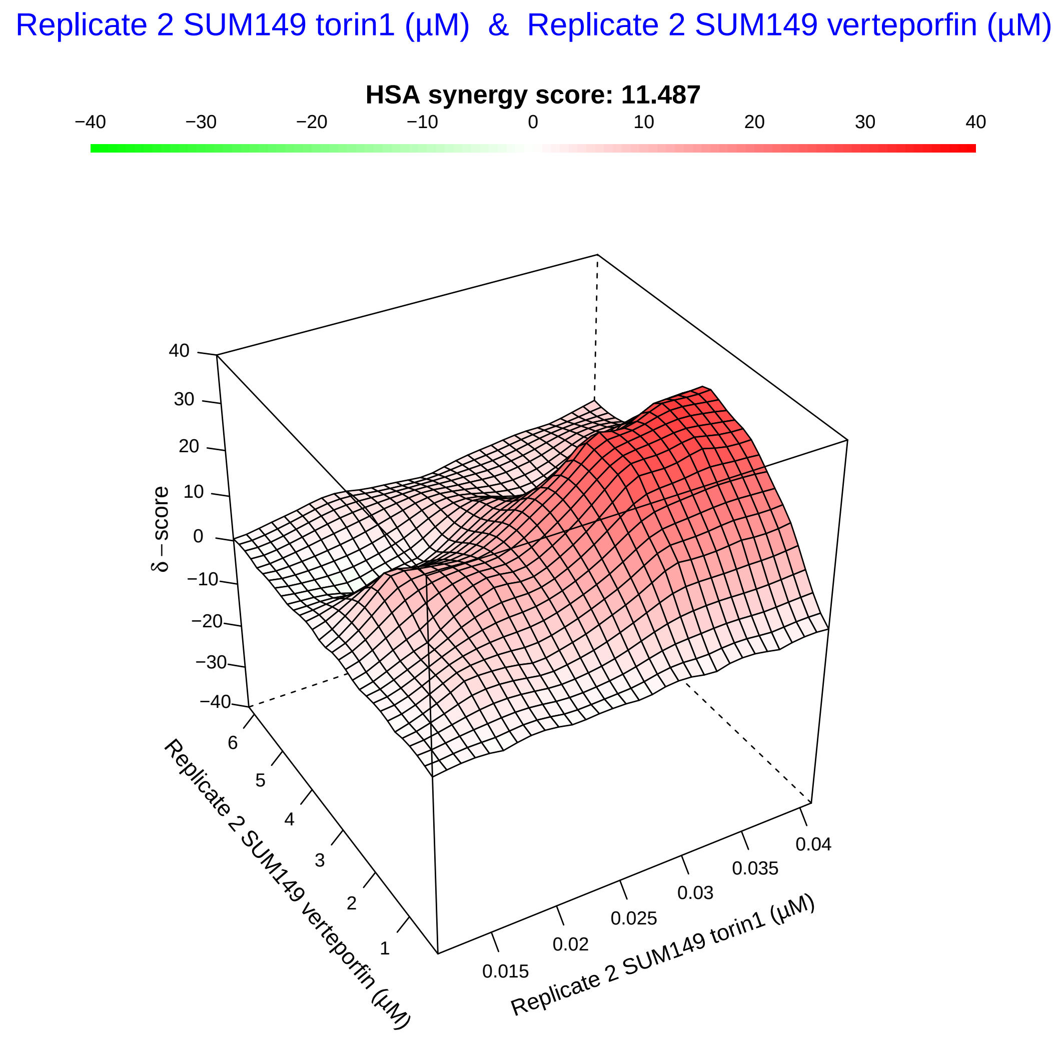
Replicate 2 SUM149 torin1 (µM) & Replicate 2 SUM149 verteporfin (µM)
HSA synergy score: 11.487
−40
−30
−20
−10
0
10
20
30
40
δ − score
Replicate 2 SUM149 verteporfin (µM)
1
2
3
4
5
6
Replicate 2 SUM149 torin1 (µM)
0.015
0.02
0.025
0.03
0.035
0.04

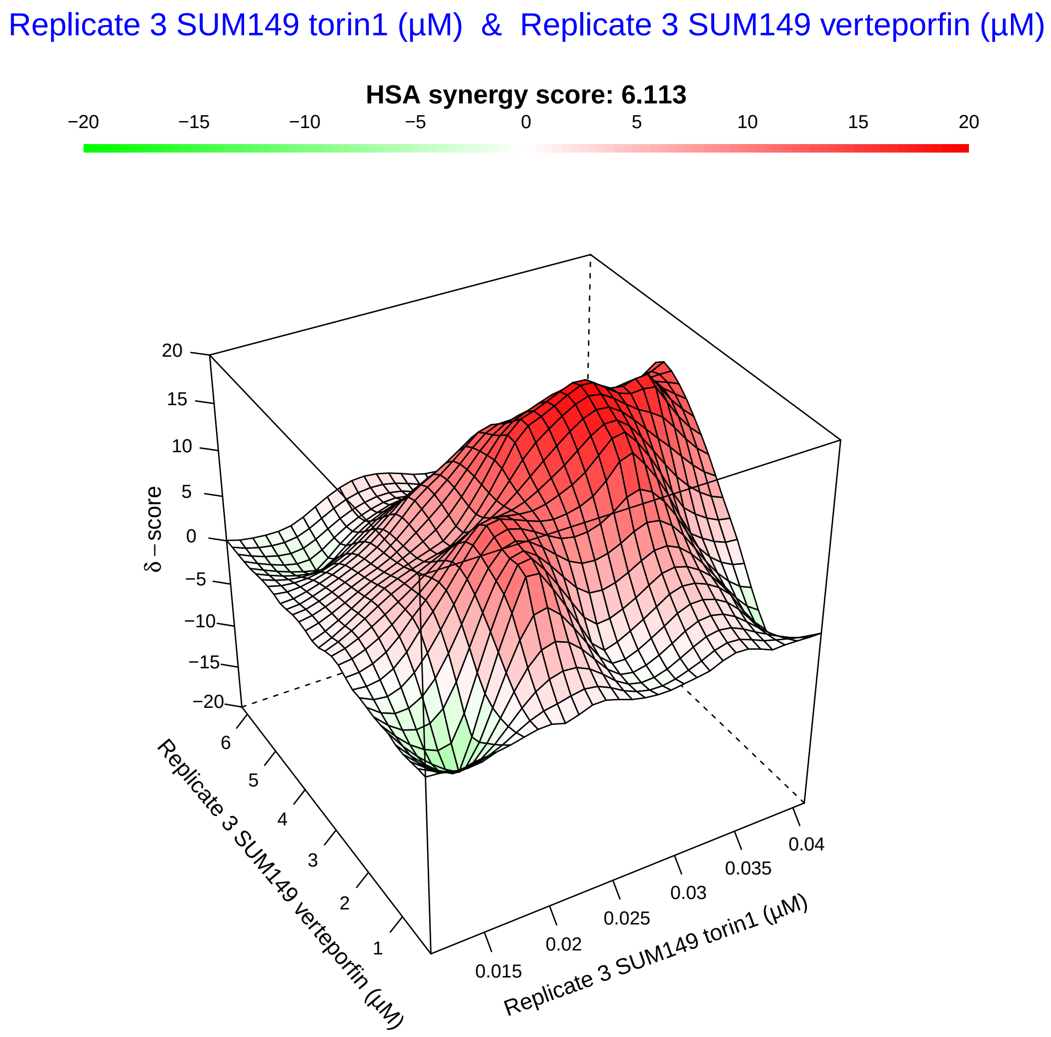
Replicate 3 SUM149 torin1 (µM) & Replicate 3 SUM149 verteporfin (µM)
HSA synergy score: 6.113
−20
−15
−10
−5
0
5
10
15
20
δ − score
Replicate 3 SUM149 verteporfin (µM)
1
2
3
4
5
6
Replicate 3 SUM149 torin1 (µM)
0.015
0.02
0.025
0.03
0.035
0.04
