# Supplementary material for: In vivo genome-wide CRISPR screen reveals breast cancer vulnerabilities and synergistic mTOR/Hippo targeted combination therapy
Source: Nat Commun. 2021 May 24;12:3055. doi: 10.1038/s41467-021-23316-4 (PMC8144221; doi:10.1038/s41467-021-23316-4)
Supplement: Supplementary file 3 — Source Data [file 41467_2021_23316_MOESM3_ESM.zip › Fig 5d and 5e result_ZIP.pdf]

Calculation and Visualization of synergy scores for Drug Combinations

Drug combinations:

Drug combination	Synergy score	Most synergistic area score	M
average 159 torin1 - average 159 verteporfin	1.97	11.83	
Replicate1 159 torin1 - Replicate 1 159 verteporfin	3.57	12.59	
Replicate2 159 torin1 - Replicate 2 159 verteporfin	1.10	8.96	
Replicate 3 159 torin1 - Replicate 3 159 verteporfin	0.93	8.44	
average MDAMB231 torin1 - average MDAMB231 verteporfin	3.22	6.38	
Replicate 1 MDAMB231 torin1 - Replicate 1 MDAMB231 verteporfin	1.42	5.39	
Replicate 2 MDAMB231 torin1 - Replicate 2 MDAMB231 verteporfin	2.42	10.05	
Replicate 3 MDAMB231 torin1 - Replicate 3 MDAMB231 verteporfin	5.21	9.57	
average SUM1315 torin1 - average SUM1315 verteporfin	2.85	16.03	
Replicate 1 SUM1315 torin1 - Replicate 1 SUM1315 verteporfin	4.88	18.20	
Replicate 2 SUM1315 torin1 - Replicate 2 SUM1315 verteporfin	6.38	19.47	
Replicate 3 SUM1315 torin1 - Replicate 3 SUM1315 verteporfin	-2.95	9.21	
average SUM149 torin1 - average SUM149 verteporfin	3.94	9.69	
Replicate 1 SUM149 torin1 - Replicate 1 SUM149 verteporfin	5.45	15.01	
Replicate 2 SUM149 torin1 - Replicate 2 SUM149 verteporfin	5.32	8.73	
Replicate 3 SUM149 torin1 - Replicate 3 SUM149 verteporfin	0.82	6.30	

Chosen parameters:

Readout: inhibition ; Baseline correction: Yes ;

average 159 torin1 & average 159 verteporfin

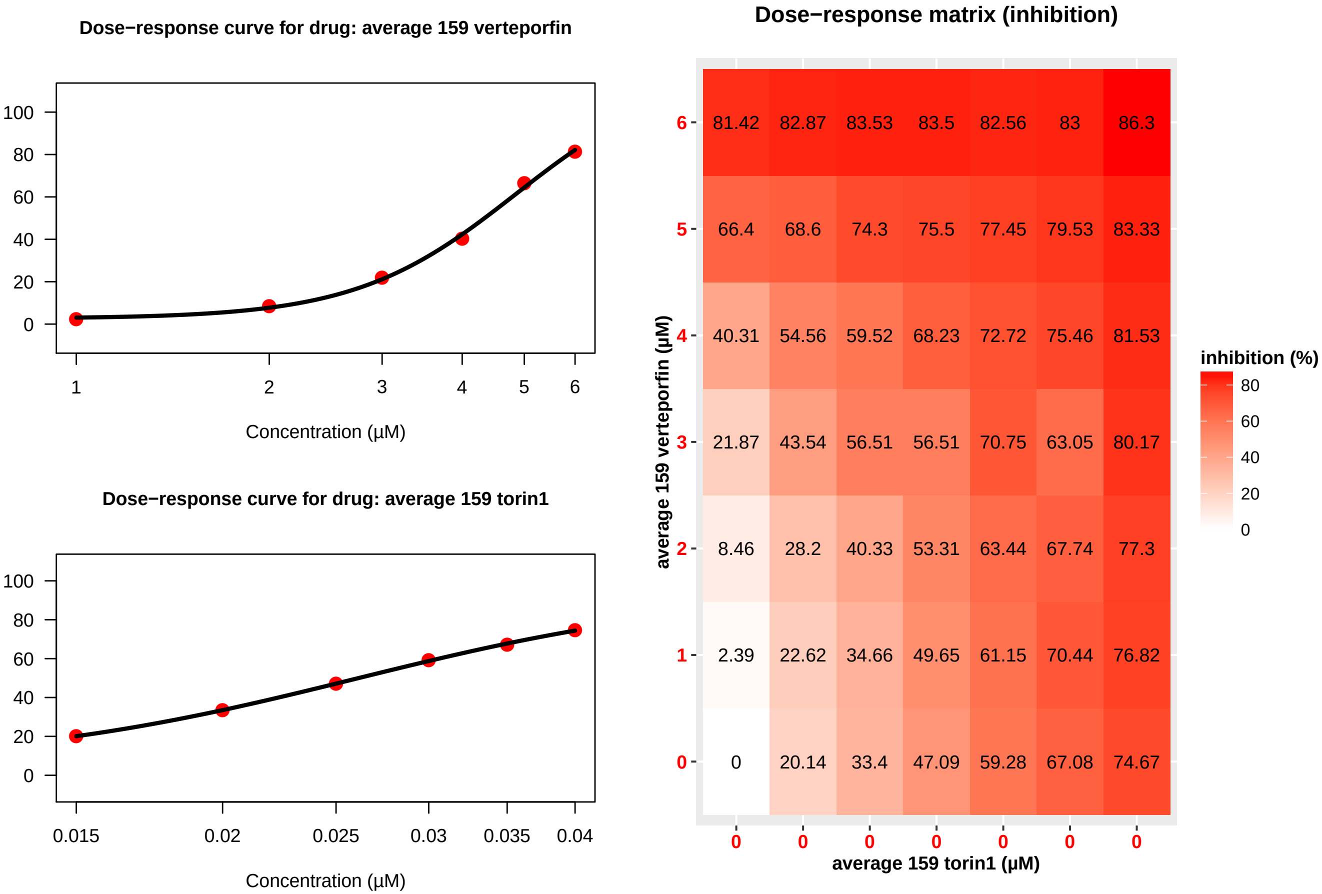

Replicate1 159 torin1 & Replicate 1 159 verteporfin

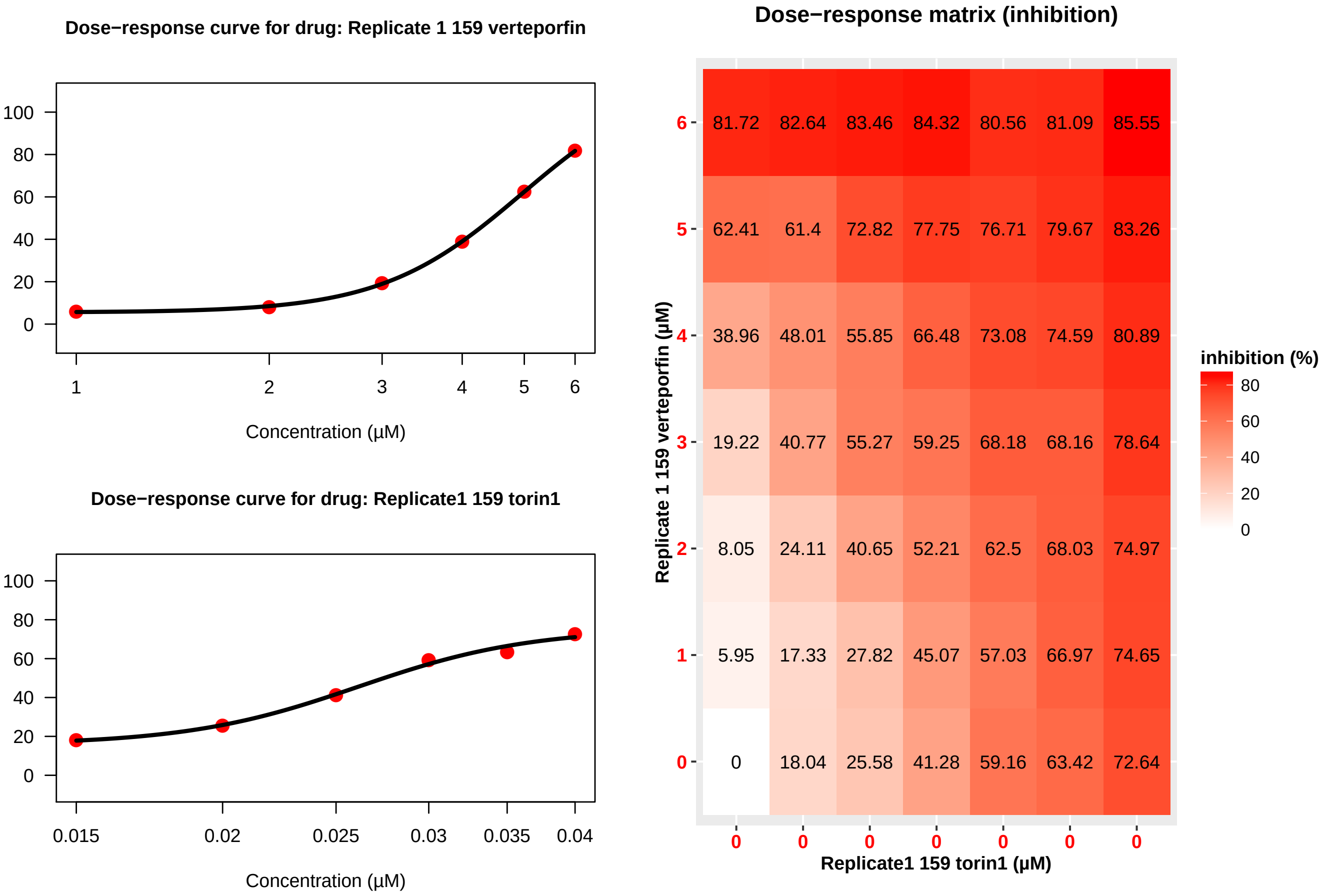

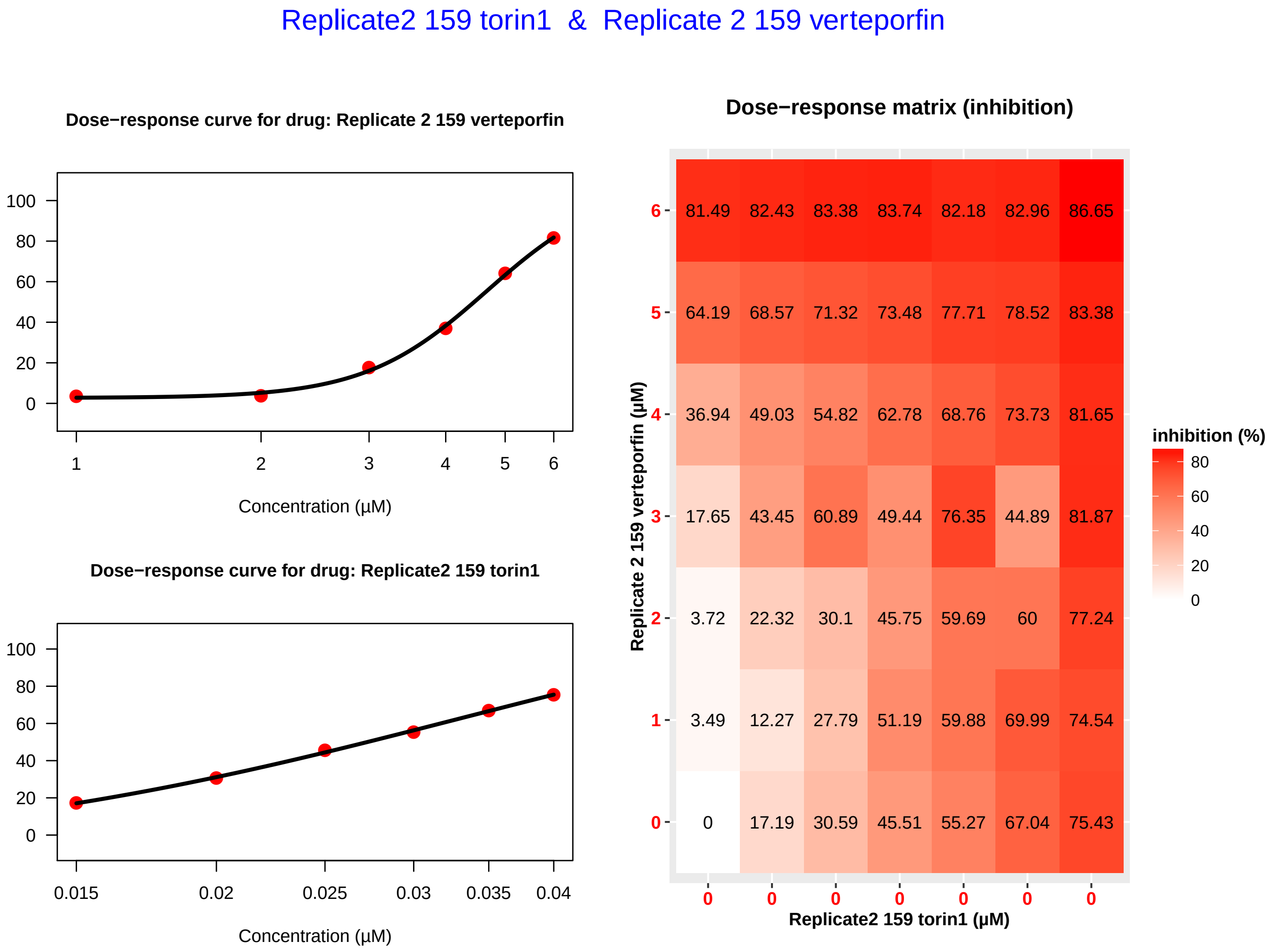
Replicate2 159 torin1 & Replicate 2 159 verteporfin
Dose−response curve for drug: Replicate 2 159 verteporfin
100
80
60
40
20
0
1
2
3
4
5
6
Concentration (µM)
Dose−response curve for drug: Replicate2 159 torin1
100
80
60
40
20
0
0.015
0.02
0.025
0.03
0.035
0.04
Concentration (µM)
Dose−response matrix (inhibition)
Replicate 2 159 verteporfin (µM)
6
5
4
3
2
1
0
81.49 82.43 83.38 83.74 82.18 82.96 86.65
64.19 68.57 71.32 73.48 77.71 78.52 83.38
36.94 49.03 54.82 62.78 68.76 73.73 81.65
17.65 43.45 60.89 49.44 76.35 44.89 81.87
3.72 22.32 30.1 45.75 59.69 60 77.24
3.49 12.27 27.79 51.19 59.88 69.99 74.54
0 17.19 30.59 45.51 55.27 67.04 75.43
0 0 0 0 0 0 0
Replicate2 159 torin1 (µM)
inhibition (%)
80
60
40
20
0

Replicate 3 159 torin1 & Replicate 3 159 verteporfin

Dose−response curve for drug: Replicate 3 159 verteporfin

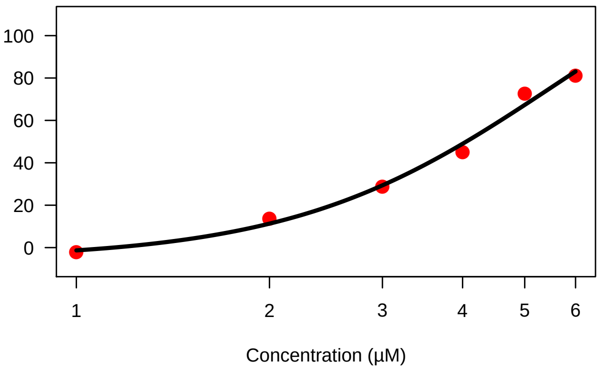

Dose−response curve for drug: Replicate 3 159 torin1

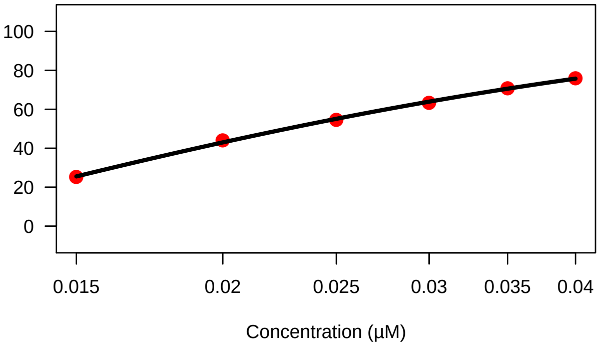

Dose−response matrix (inhibition)

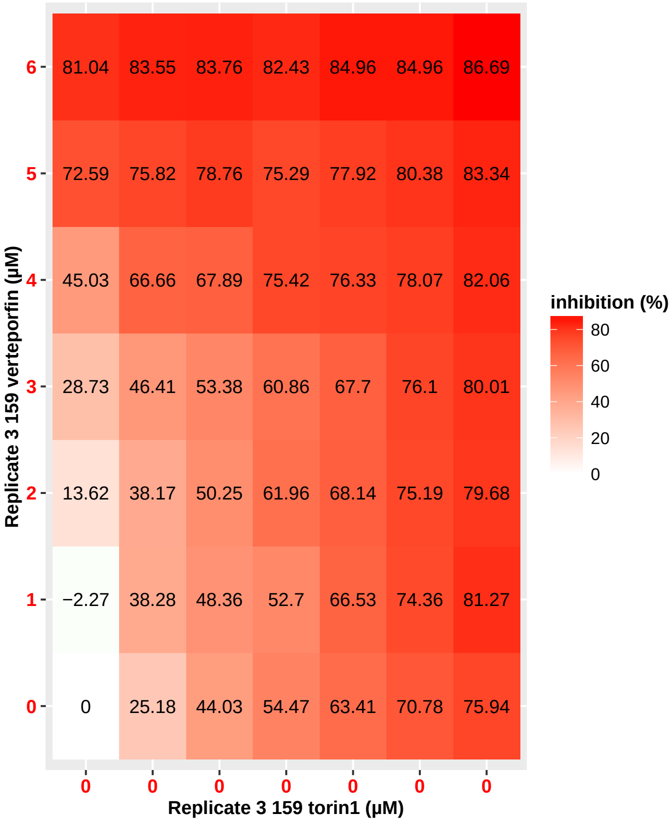

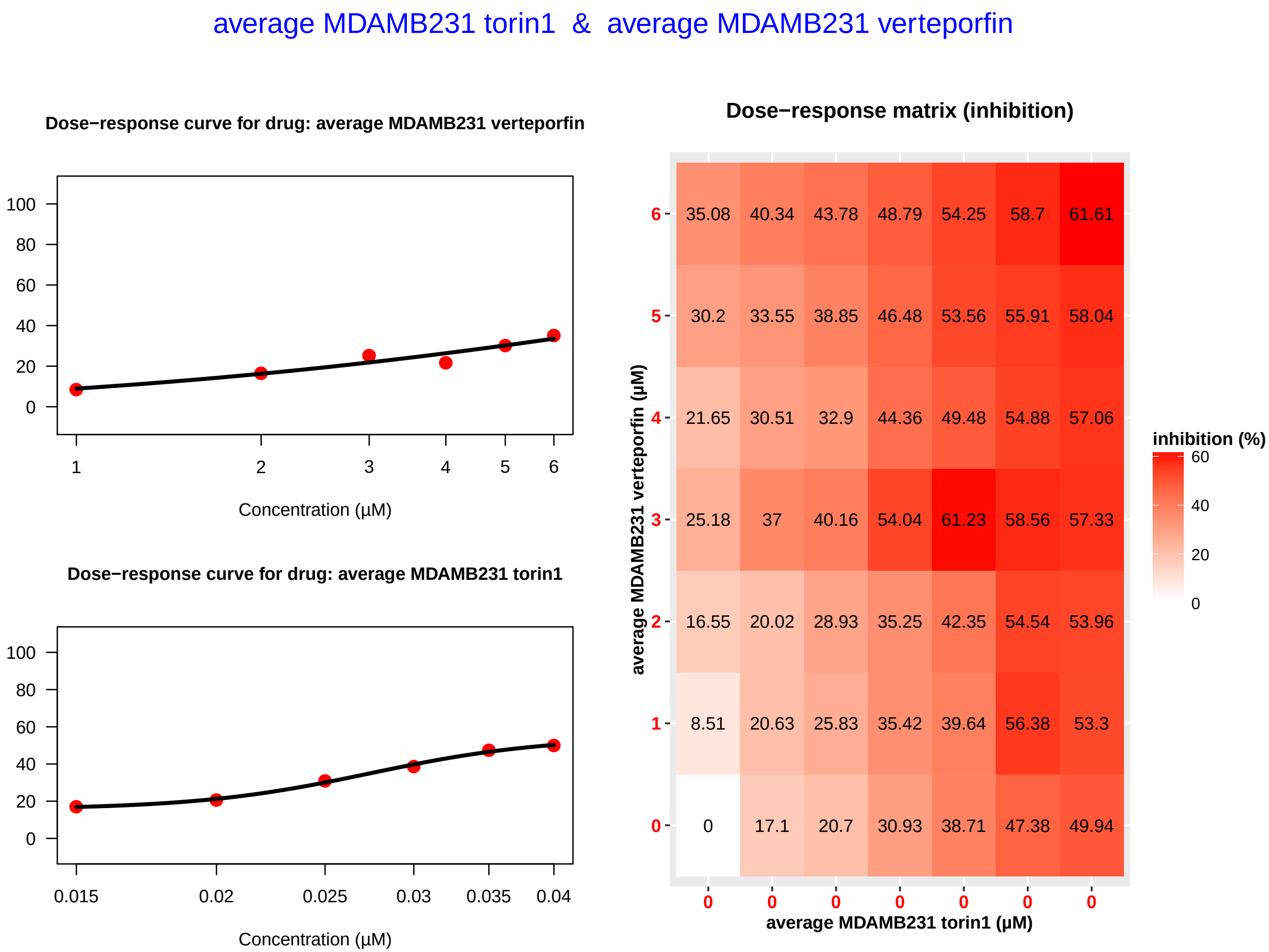
average MDAMB231 torin1 & average MDAMB231 verteporfin
Dose−response curve for drug: average MDAMB231 verteporfin
100
80
60
40
20
0
1
2
3
4
5
6
Concentration (µM)
Dose−response curve for drug: average MDAMB231 torin1
100
80
60
40
20
0
0.015
0.02
0.025
0.03
0.035
0.04
Concentration (µM)
Dose−response matrix (inhibition)
average MDAMB231 verteporfin (µM)
6 35.08 40.34 43.78 48.79 54.25 58.7 61.61
5 30.2 33.55 38.85 46.48 53.56 55.91 58.04
4 21.65 30.51 32.9 44.36 49.48 54.88 57.06
3 25.18 37 40.16 54.04 61.23 58.56 57.33
2 16.55 20.02 28.93 35.25 42.35 54.54 53.96
1 8.51 20.63 25.83 35.42 39.64 56.38 53.3
0 0 17.1 20.7 30.93 38.71 47.38 49.94
0 0 0 0 0 0 0
average MDAMB231 torin1 (µM)
inhibition (%)
60
40
20
0

Replicate 1 MDAMB231 torin1 & Replicate 1 MDAMB231 verteporfin

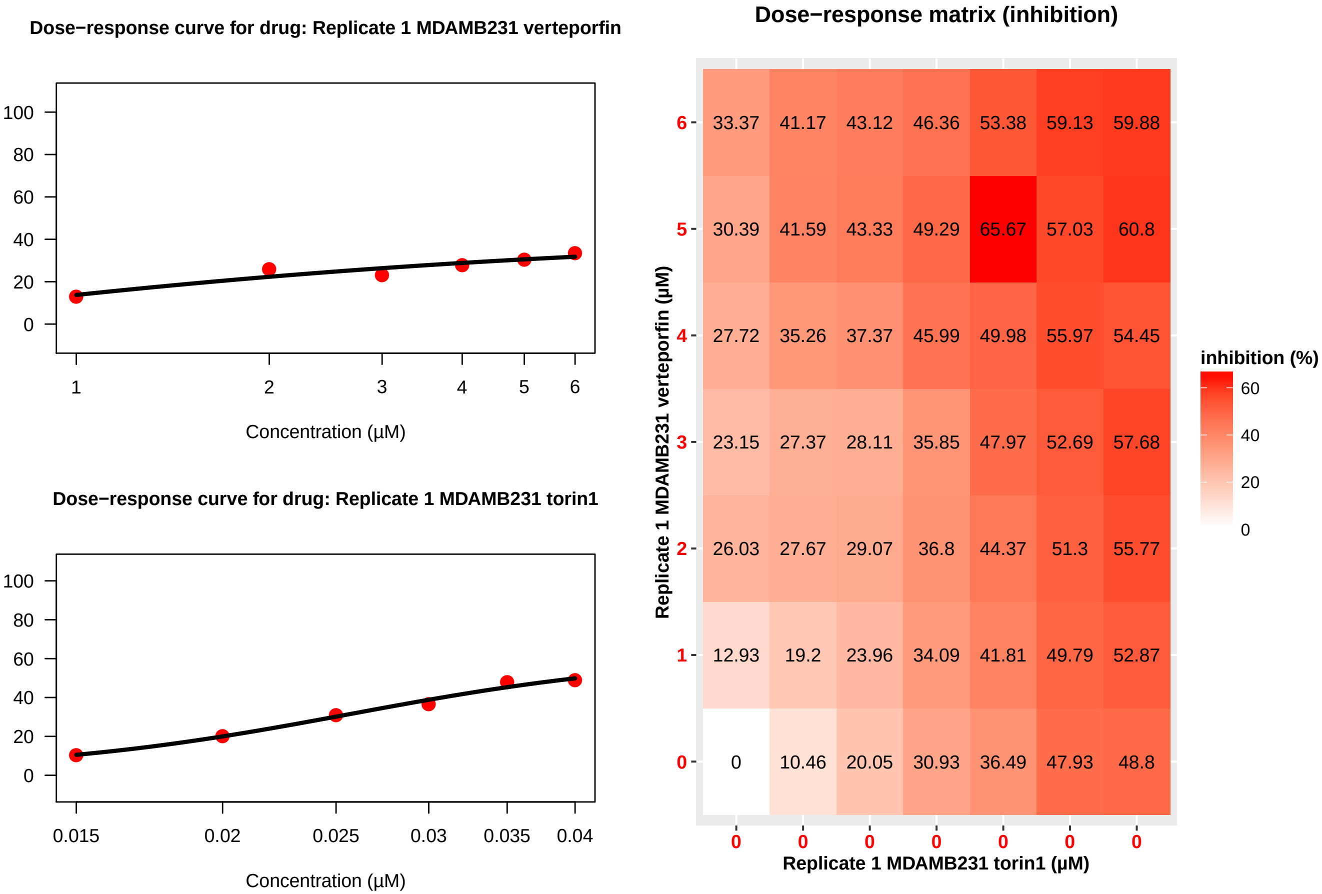

Replicate 2 MDAMB231 torin1 & Replicate 2 MDAMB231 verteporfin

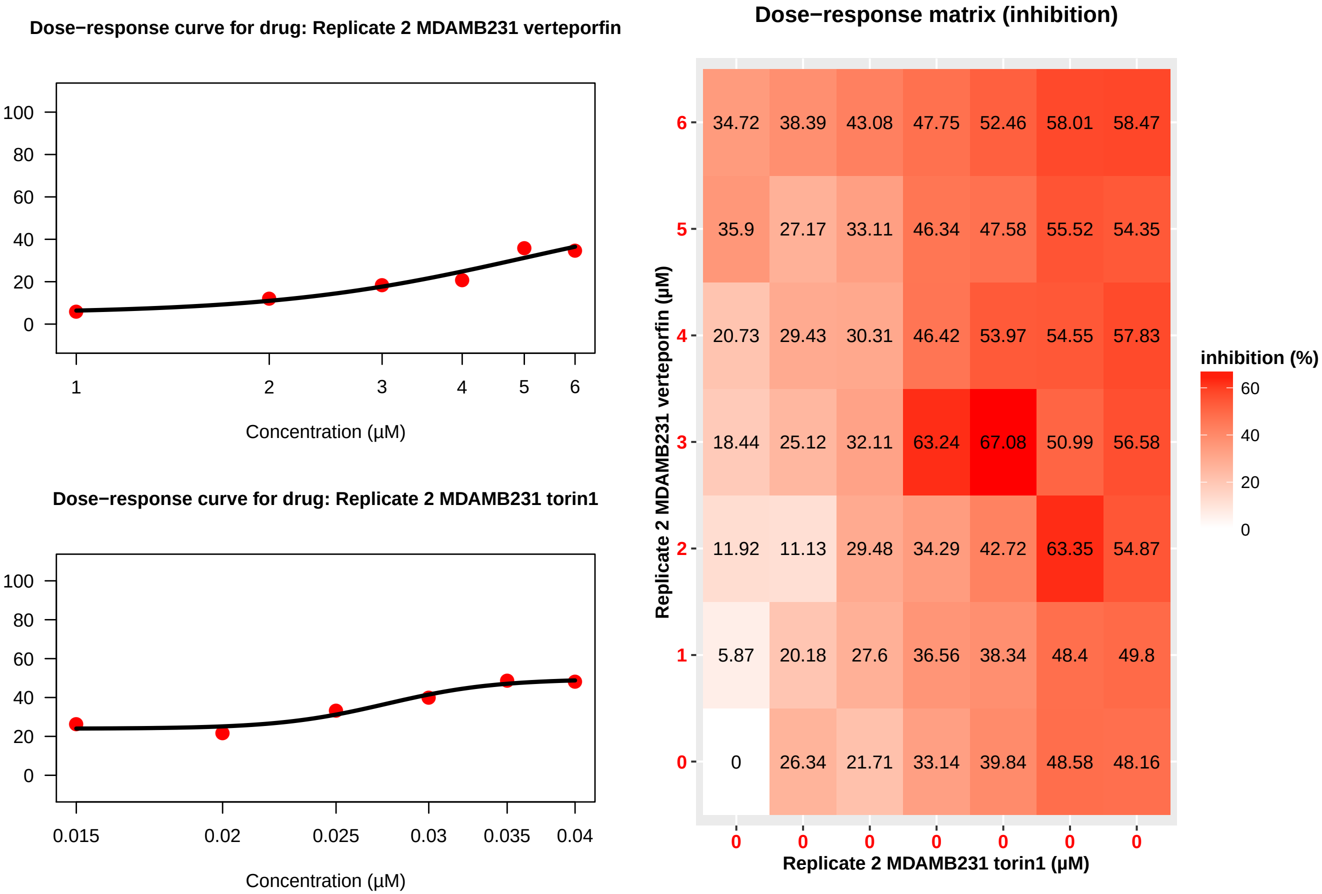

Replicate 3 MDAMB231 torin1 & Replicate 3 MDAMB231 verteporfin

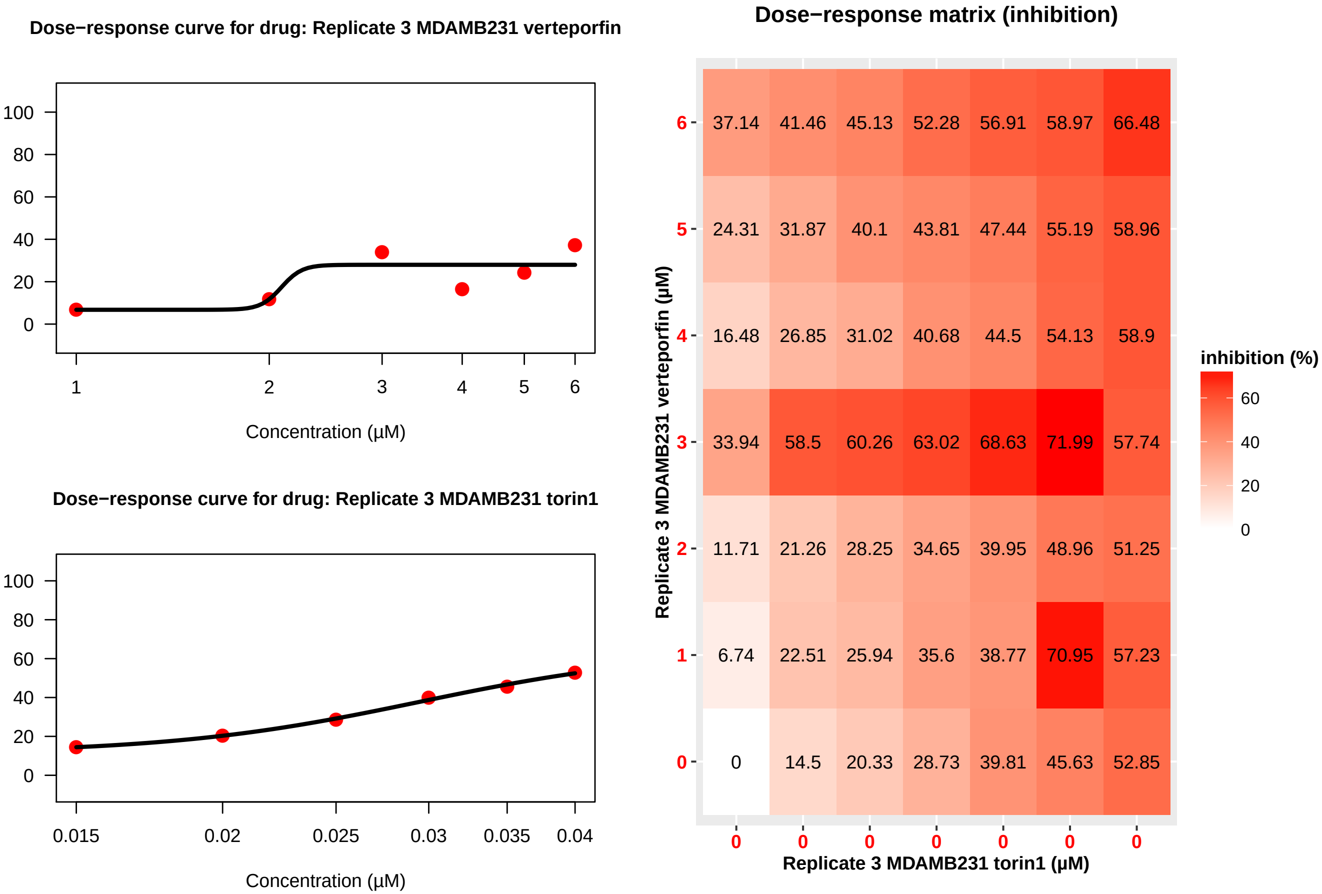

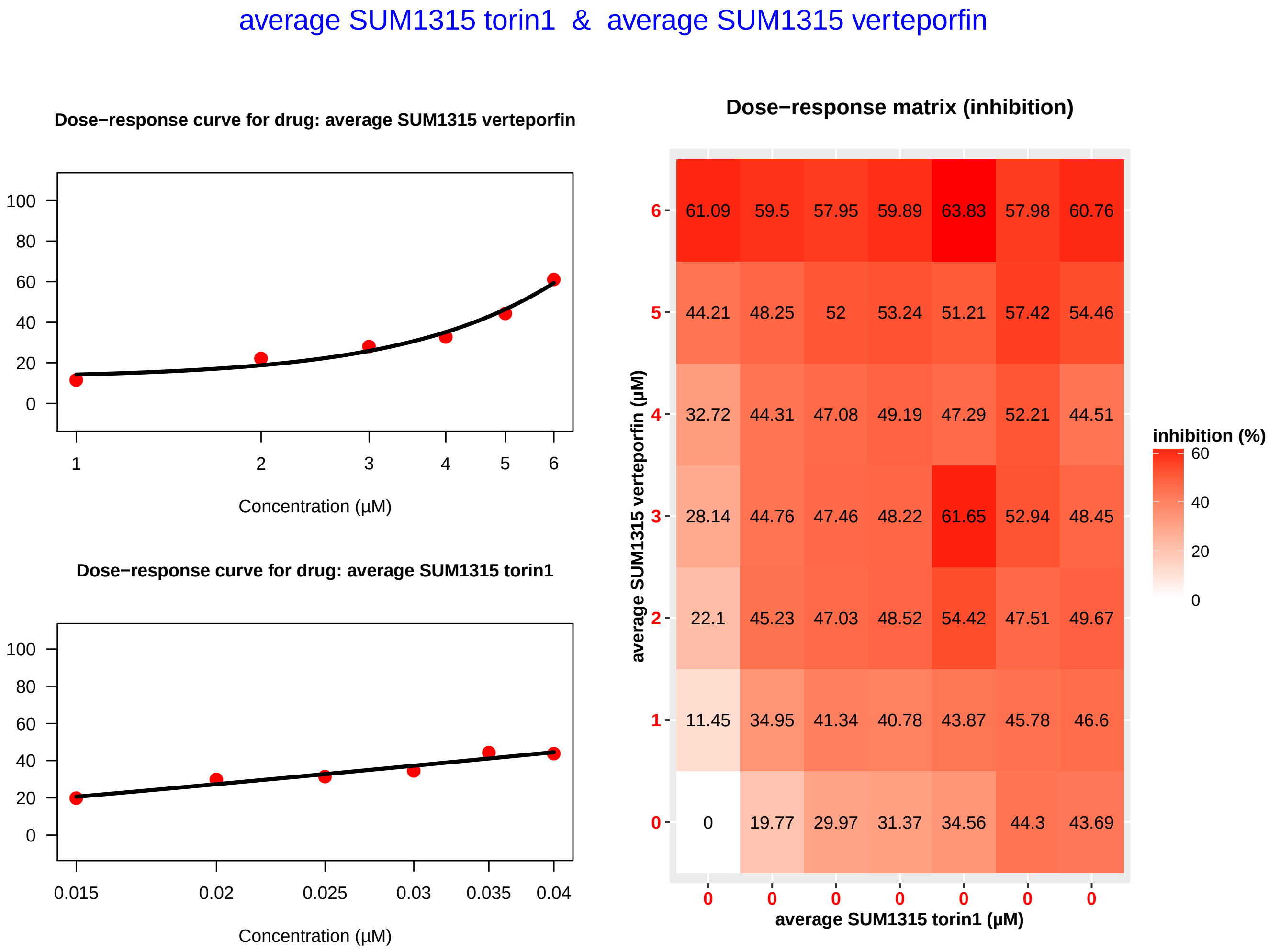
average SUM1315 torin1 & average SUM1315 verteporfin
Dose−response curve for drug: average SUM1315 verteporfin
100
80
60
40
20
0
1
2
3
4
5
6
Concentration (µM)
Dose−response curve for drug: average SUM1315 torin1
100
80
60
40
20
0
0.015
0.02
0.025
0.03
0.035
0.04
Concentration (µM)
Dose−response matrix (inhibition)
average SUM1315 verteporfin (µM)
6
61.09 59.5 57.95 59.89 63.83 57.98 60.76
5
44.21 48.25 52 53.24 51.21 57.42 54.46
4
32.72 44.31 47.08 49.19 47.29 52.21 44.51
3
28.14 44.76 47.46 48.22 61.65 52.94 48.45
2
22.1 45.23 47.03 48.52 54.42 47.51 49.67
1
11.45 34.95 41.34 40.78 43.87 45.78 46.6
0
0 19.77 29.97 31.37 34.56 44.3 43.69
0 0 0 0 0 0 0
average SUM1315 torin1 (µM)
inhibition (%)
60
40
20
0

Replicate 1 SUM1315 torin1 & Replicate 1 SUM1315 verteporfin

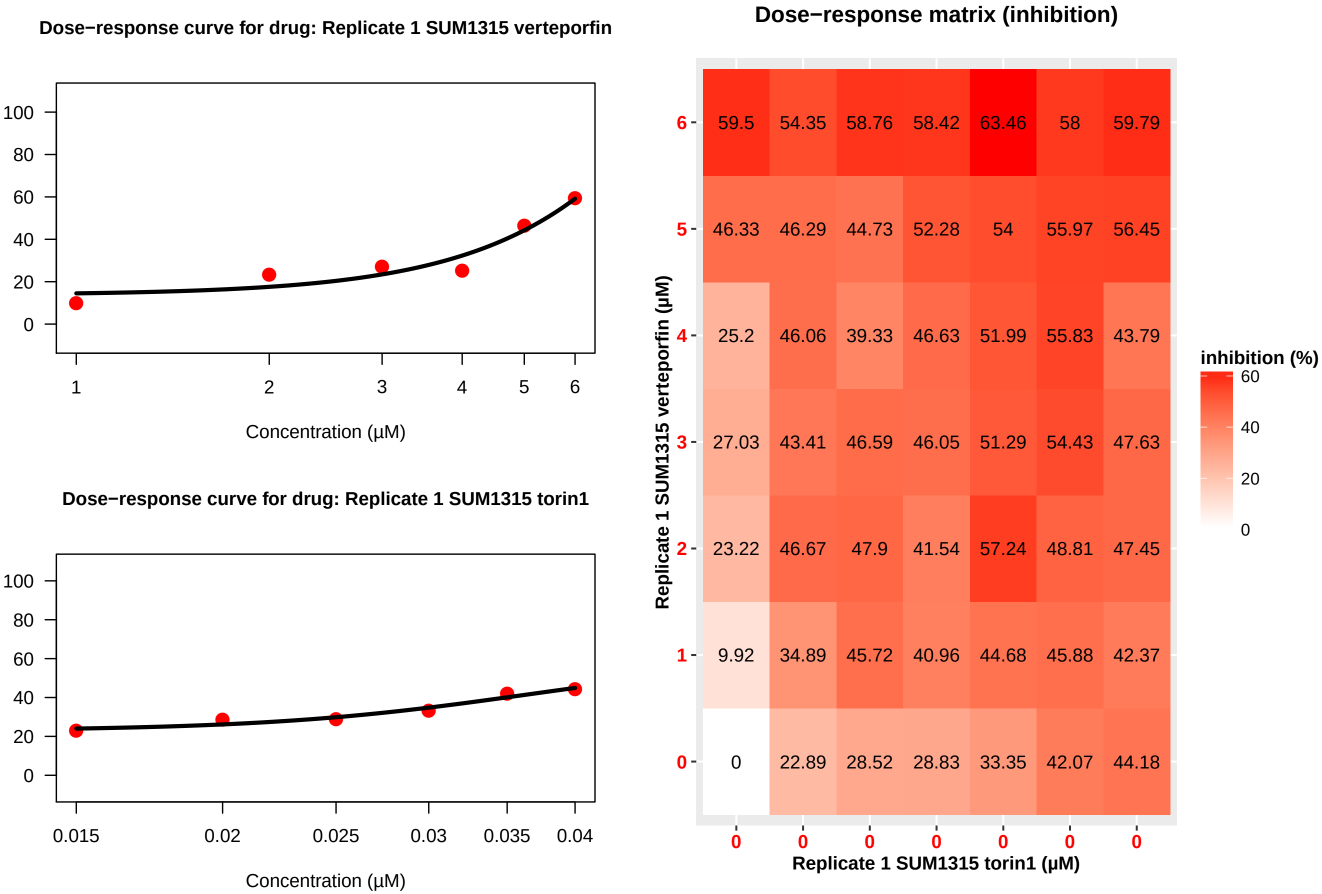

Replicate 2 SUM1315 torin1 & Replicate 2 SUM1315 verteporfin

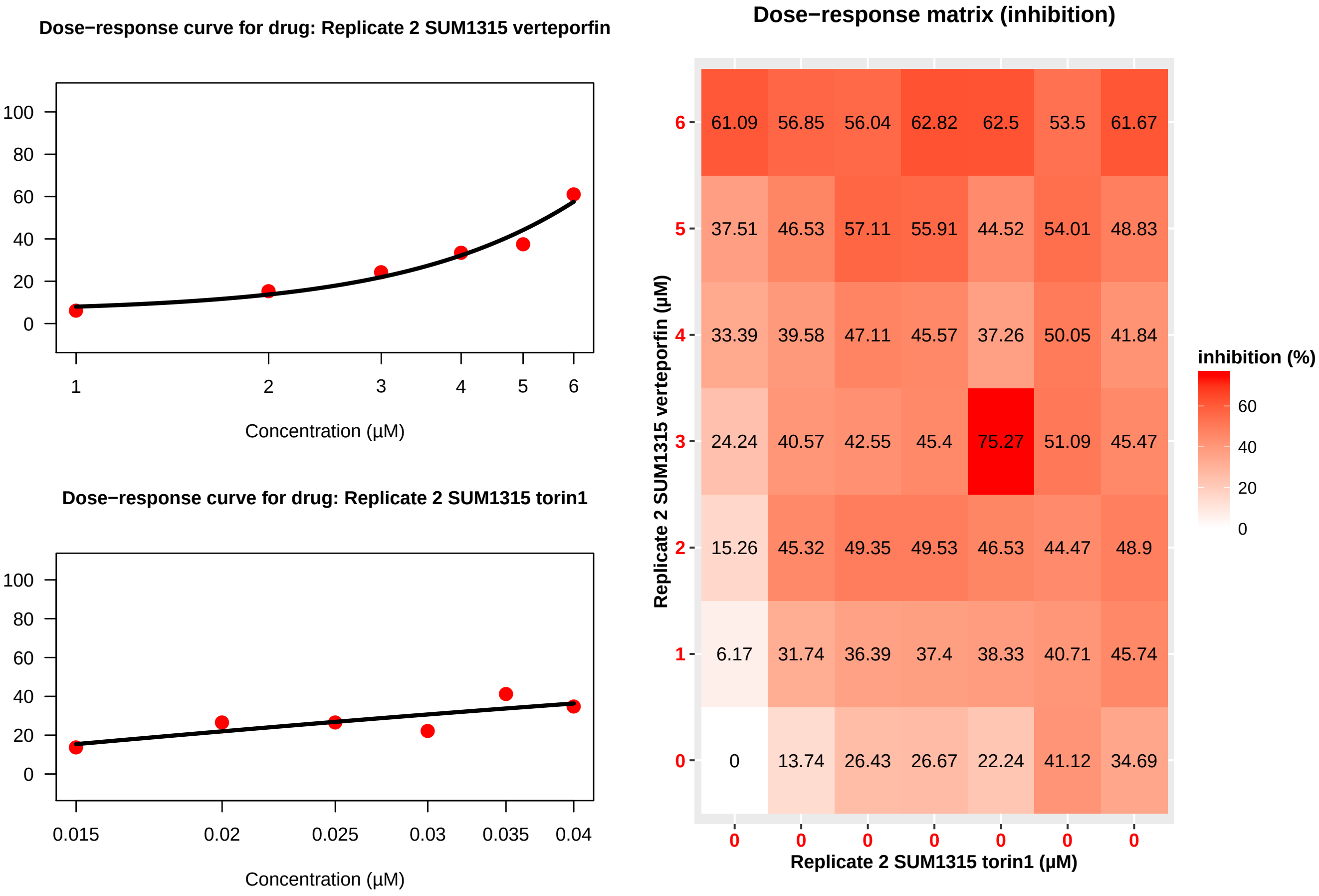

Replicate 3 SUM1315 torin1 & Replicate 3 SUM1315 verteporfin

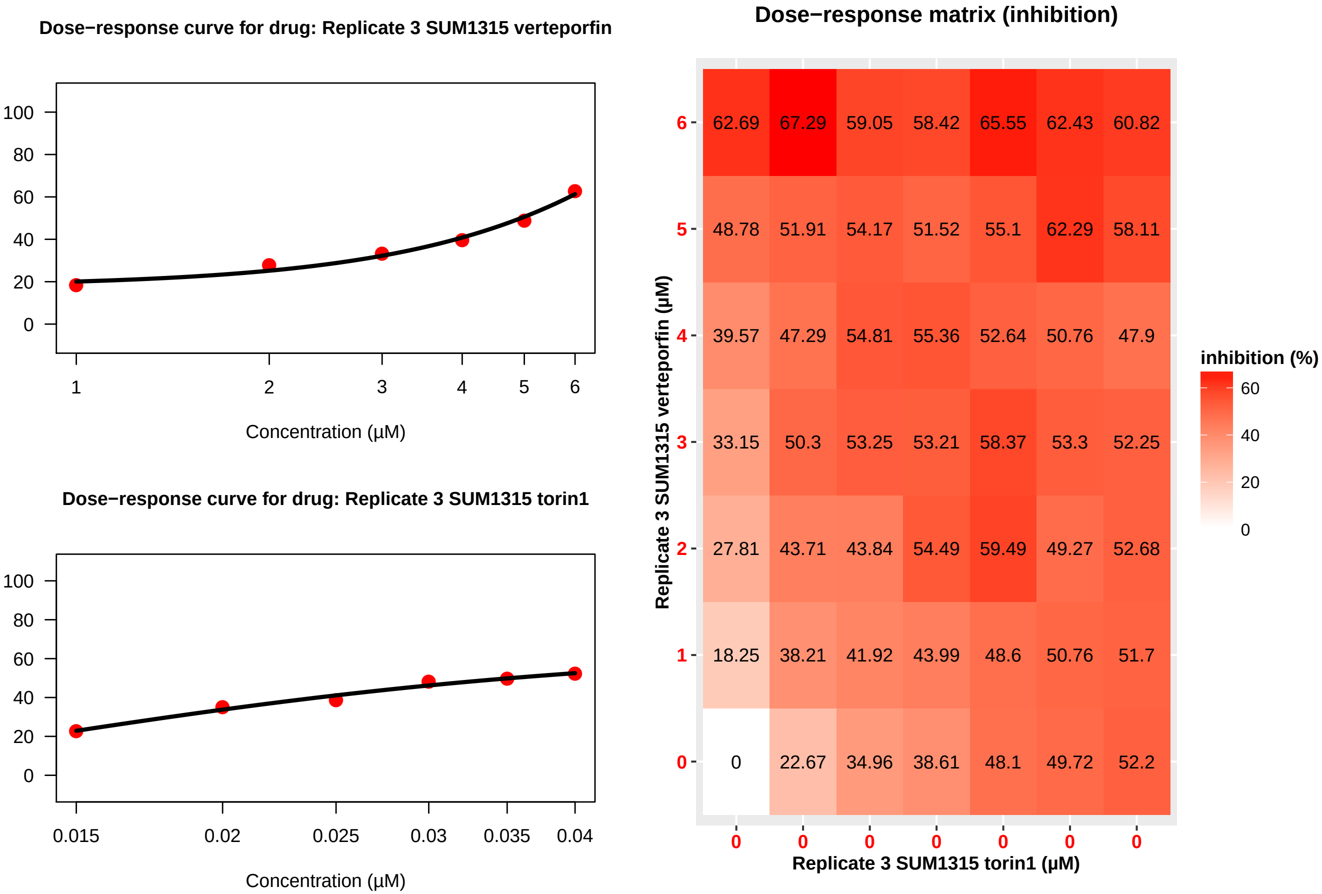

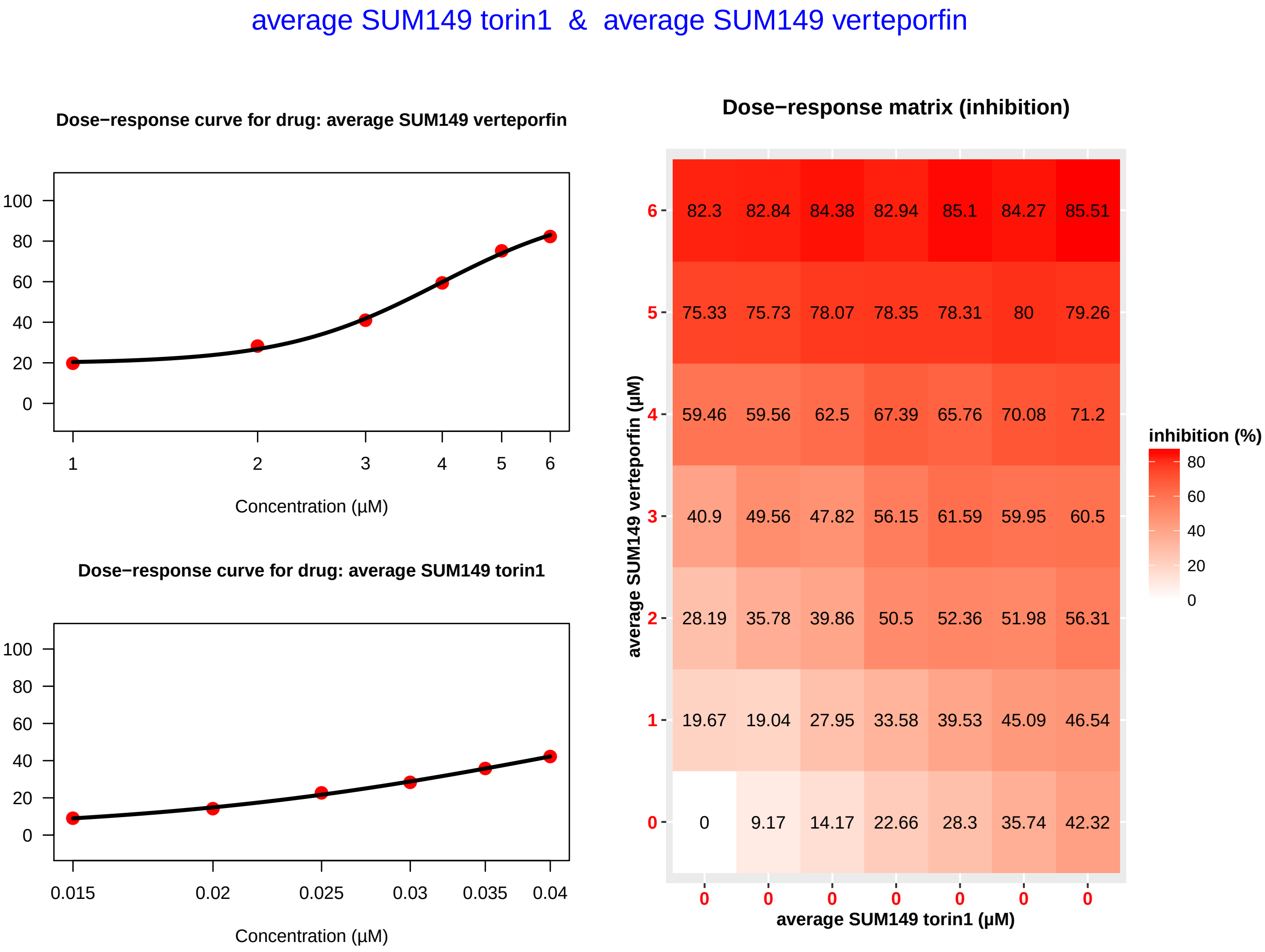
average SUM149 torin1 & average SUM149 verteporfin
Dose−response curve for drug: average SUM149 verteporfin
Concentration (µM)
Dose−response curve for drug: average SUM149 torin1
Concentration (µM)
Dose−response matrix (inhibition)
average SUM149 verteporfin (µM)
average SUM149 torin1 (µM)
inhibition (%)
82.3 82.84 84.38 82.94 85.1 84.27 85.51
75.33 75.73 78.07 78.35 78.31 80 79.26
59.46 59.56 62.5 67.39 65.76 70.08 71.2
40.9 49.56 47.82 56.15 61.59 59.95 60.5
28.19 35.78 39.86 50.5 52.36 51.98 56.31
19.67 19.04 27.95 33.58 39.53 45.09 46.54
0 9.17 14.17 22.66 28.3 35.74 42.32

Replicate 1 SUM149 torin1 & Replicate 1 SUM149 verteporfin

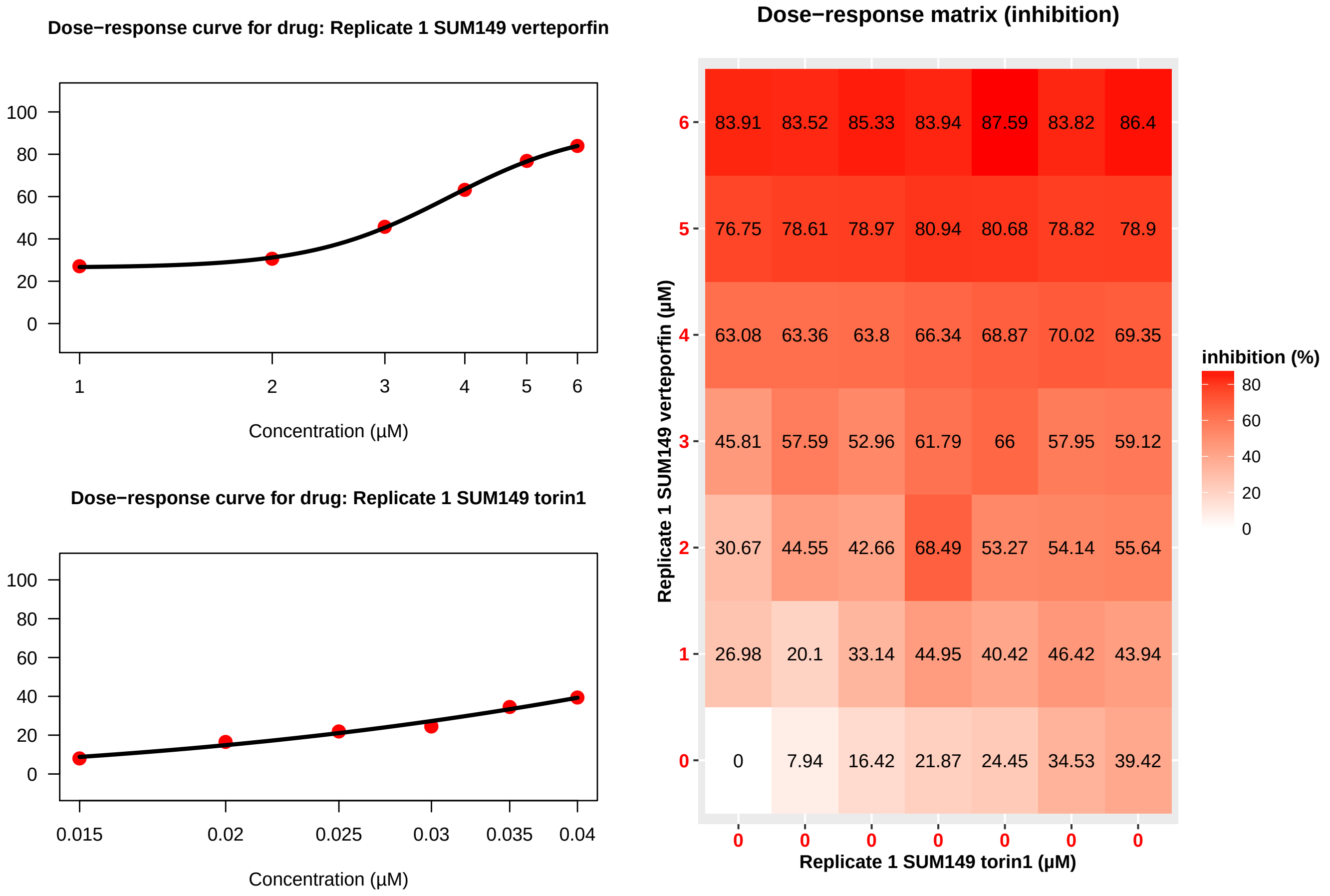

Replicate 2 SUM149 torin1 & Replicate 2 SUM149 verteporfin

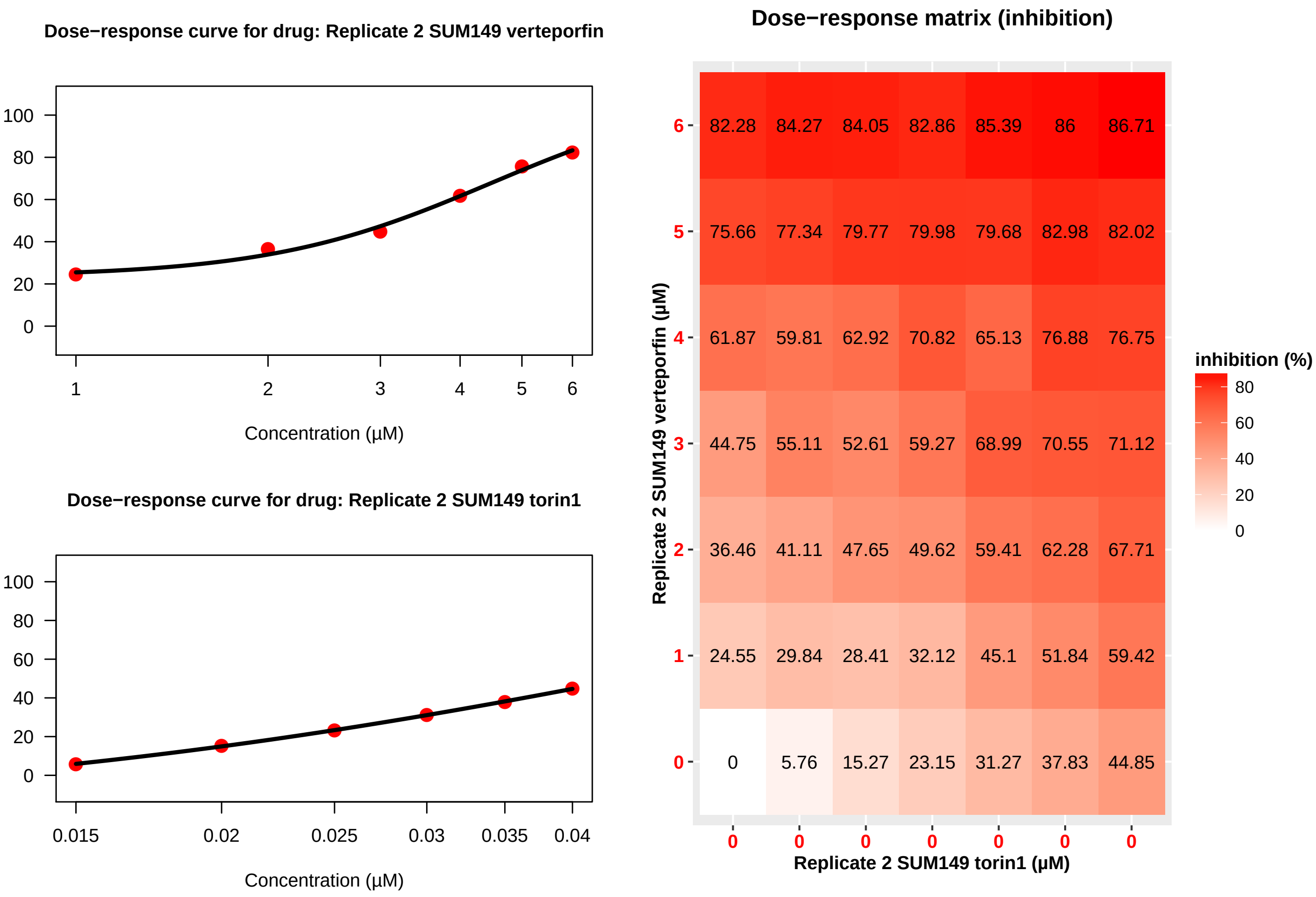

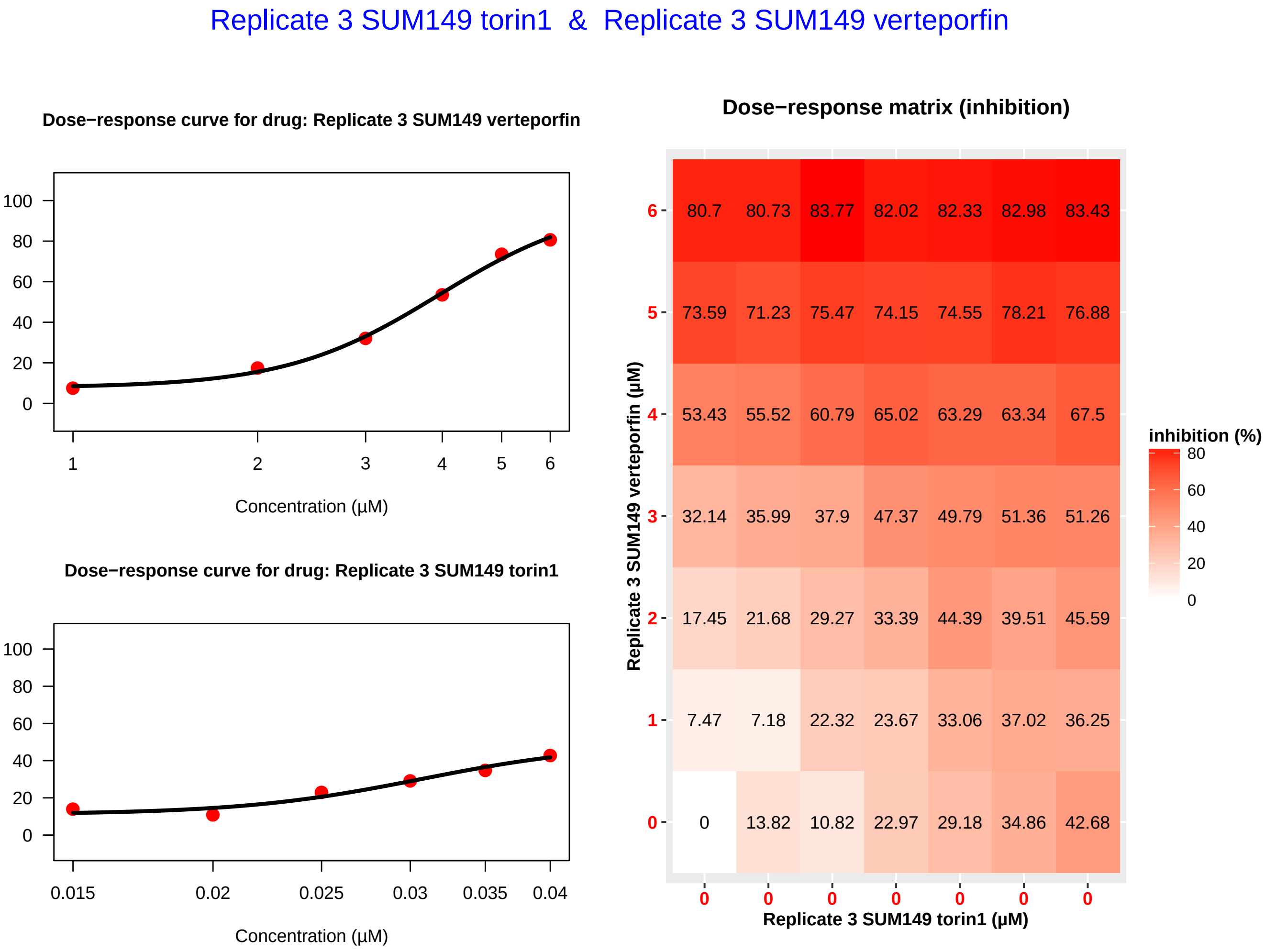
Replicate 3 SUM149 torin1 & Replicate 3 SUM149 verteporfin
Dose−response curve for drug: Replicate 3 SUM149 verteporfin
100
80
60
40
20
0
1
2
3
4
5
6
Concentration (µM)
Dose−response curve for drug: Replicate 3 SUM149 torin1
100
80
60
40
20
0
0.015
0.02
0.025
0.03
0.035
0.04
Concentration (µM)
Dose−response matrix (inhibition)
6
80.7 80.73 83.77 82.02 82.33 82.98 83.43
5
73.59 71.23 75.47 74.15 74.55 78.21 76.88
4
53.43 55.52 60.79 65.02 63.29 63.34 67.5
3
32.14 35.99 37.9 47.37 49.79 51.36 51.26
2
17.45 21.68 29.27 33.39 44.39 39.51 45.59
1
7.47 7.18 22.32 23.67 33.06 37.02 36.25
0
0 13.82 10.82 22.97 29.18 34.86 42.68
0 0 0 0 0 0 0
Replicate 3 SUM149 verteporfin (µM)
Replicate 3 SUM149 torin1 (µM)
inhibition (%)
80
60
40
20
0

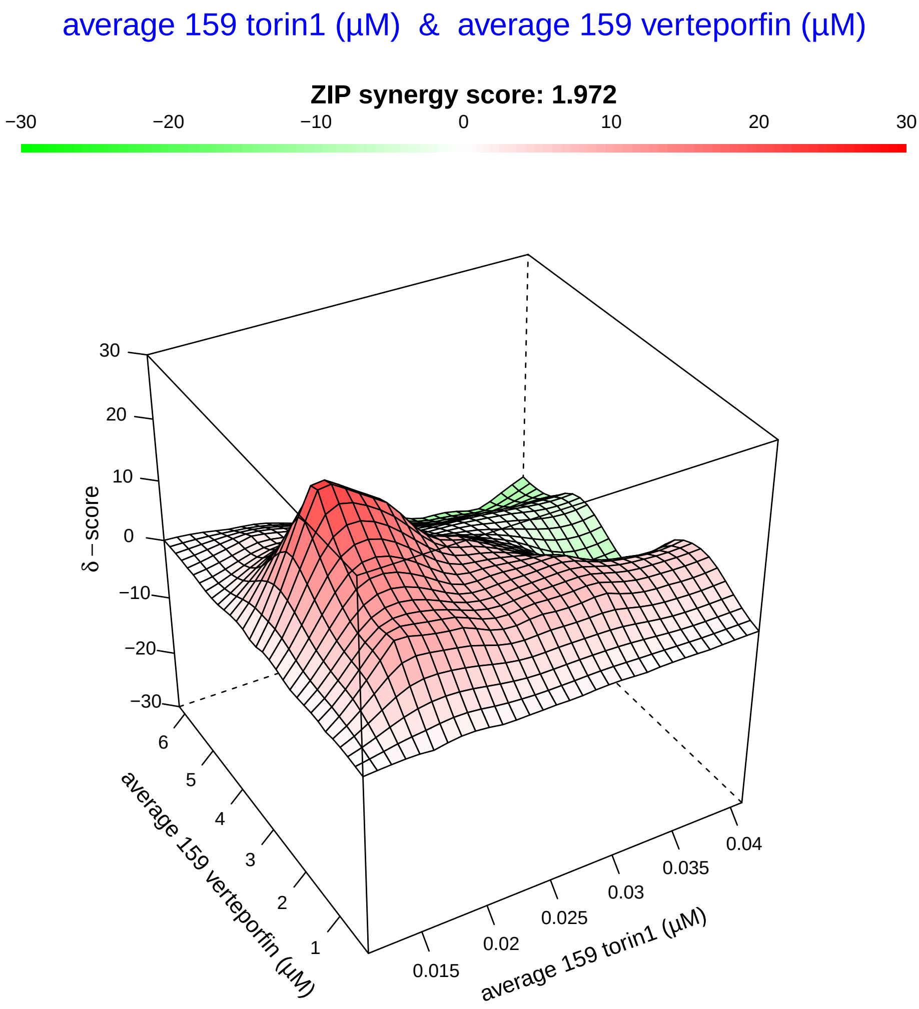
average 159 torin1 (µM) & average 159 verteporfin (µM)
ZIP synergy score: 1.972
−30
−20
−10
0
10
20
30
δ − score
average 159 verteporfin (µM)
average 159 torin1 (µM)
0.015
0.02
0.025
0.03
0.035
0.04
1
2
3
4
5
6

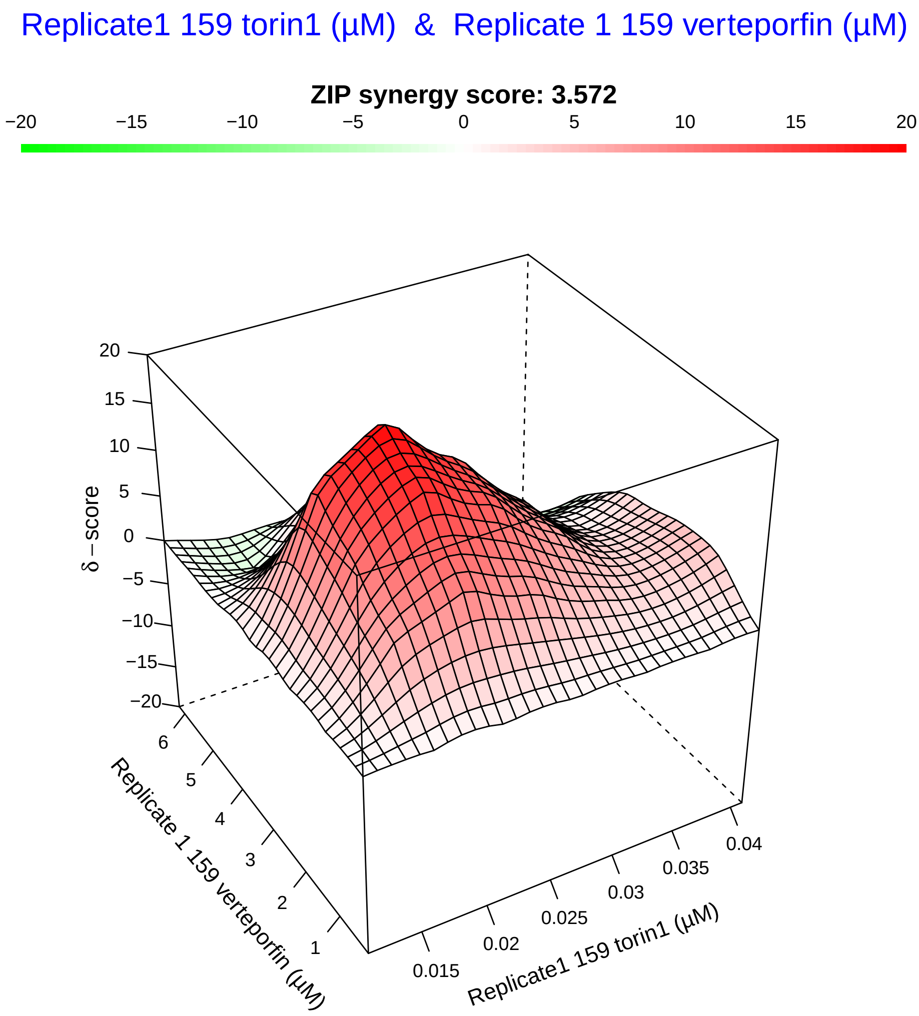
Replicate1 159 torin1 (µM) & Replicate 1 159 verteporfin (µM)
ZIP synergy score: 3.572
−20 −15 −10 −5 0 5 10 15 20
δ − score
20 15 10 5 0 −5 −10 −15 −20
Replicate 1 159 verteporfin (µM)
1 2 3 4 5 6
Replicate1 159 torin1 (µM)
0.015 0.02 0.025 0.03 0.035 0.04

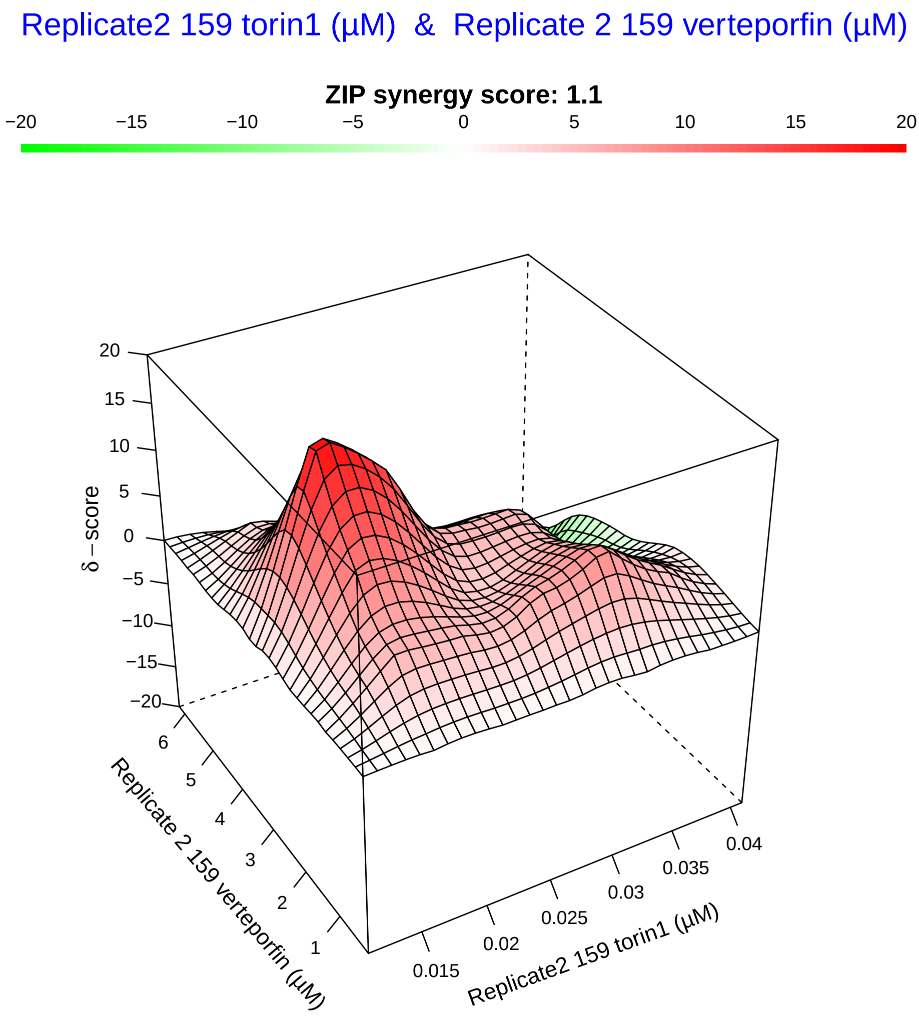

Replicate2 159 torin1 (µM) & Replicate 2 159 verteporfin (µM)
ZIP synergy score: 1.1
−20
−15
−10
−5
0
5
10
15
20
δ − score
Replicate 2 159 verteporfin (µM)
1
2
3
4
5
6
Replicate2 159 torin1 (µM)
0.015
0.02
0.025
0.03
0.035
0.04

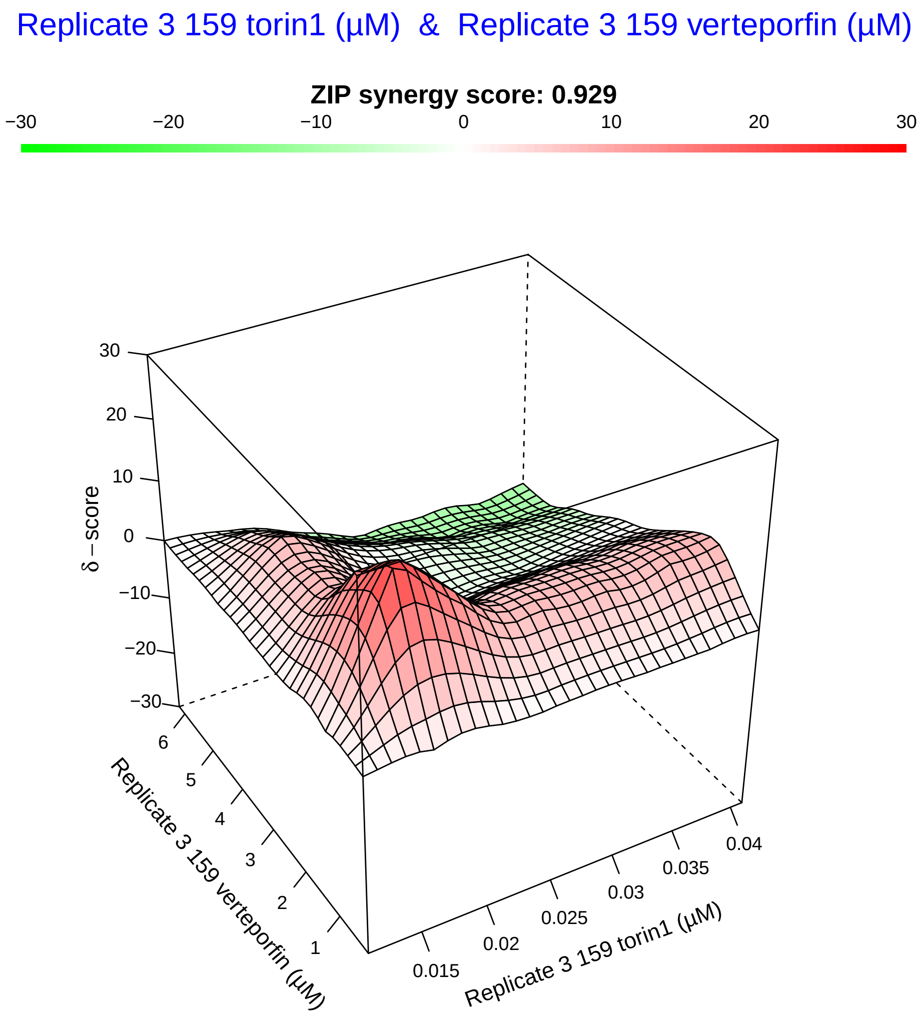
Replicate 3 159 torin1 (µM) & Replicate 3 159 verteporfin (µM)
ZIP synergy score: 0.929
−30
−20
−10
0
10
20
30
δ − score
Replicate 3 159 verteporfin (µM)
1
2
3
4
5
6
Replicate 3 159 torin1 (µM)
0.015
0.02
0.025
0.03
0.035
0.04

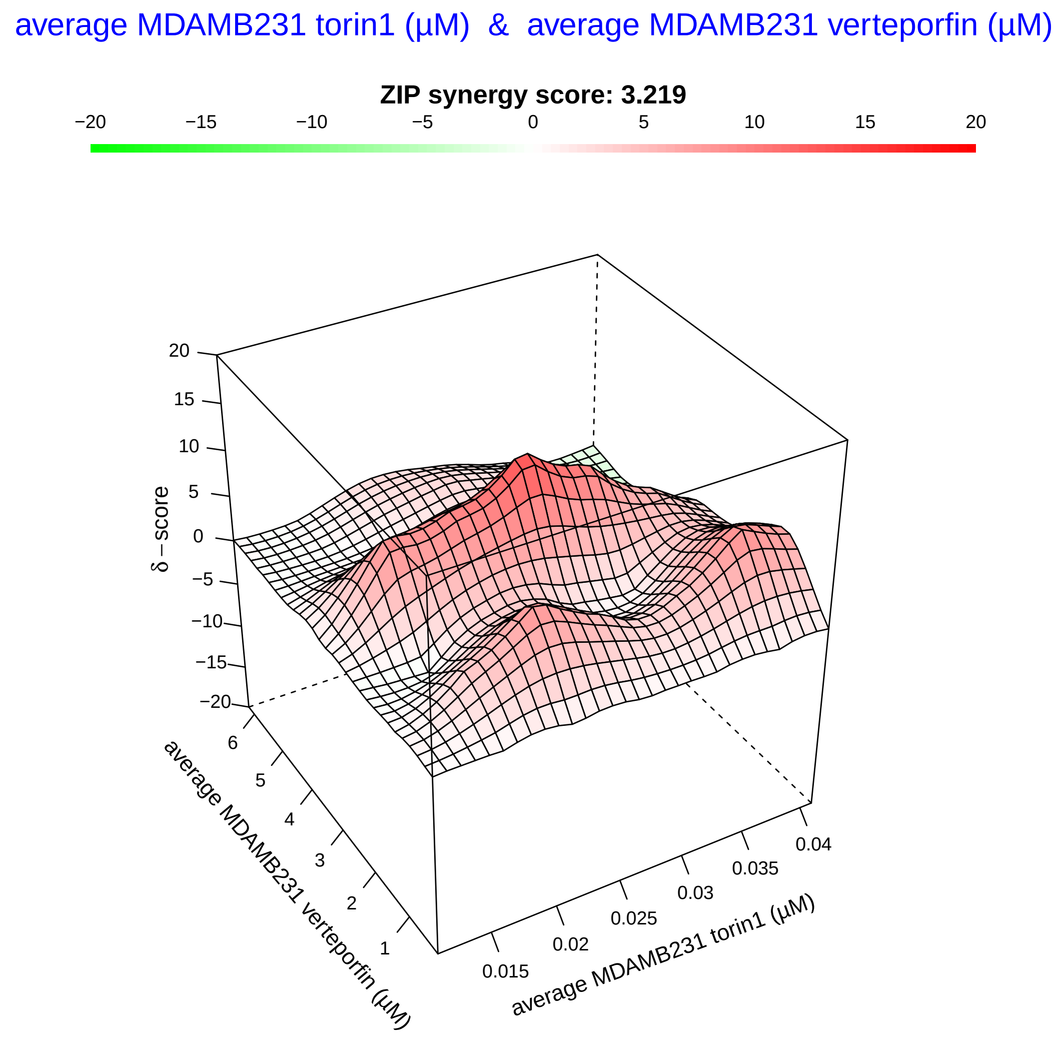
average MDAMB231 torin1 (µM) & average MDAMB231 verteporfin (µM)
ZIP synergy score: 3.219
−20
−15
−10
−5
0
5
10
15
20
δ − score
average MDAMB231 verteporfin (µM)
1
2
3
4
5
6
average MDAMB231 torin1 (µM)
0.015
0.02
0.025
0.03
0.035
0.04

Replicate 1 MDAMB231 torin1 (µM) & Replicate 1 MDAMB231 verteporfin (µM)

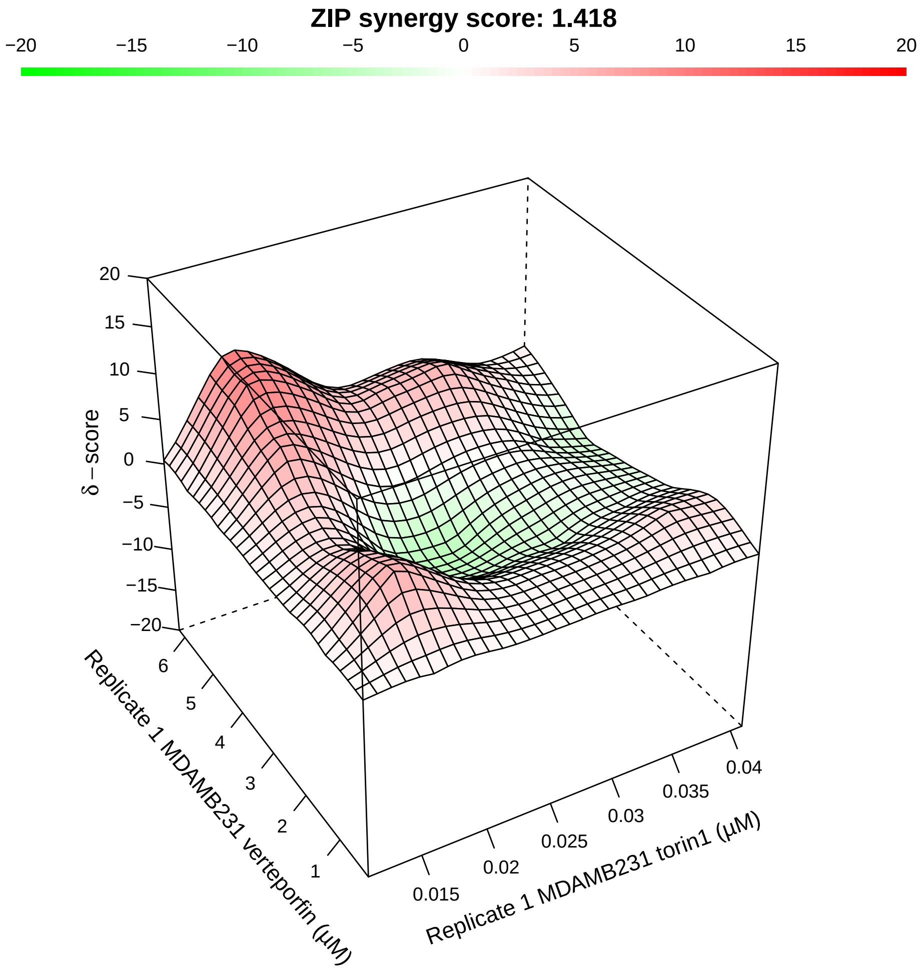

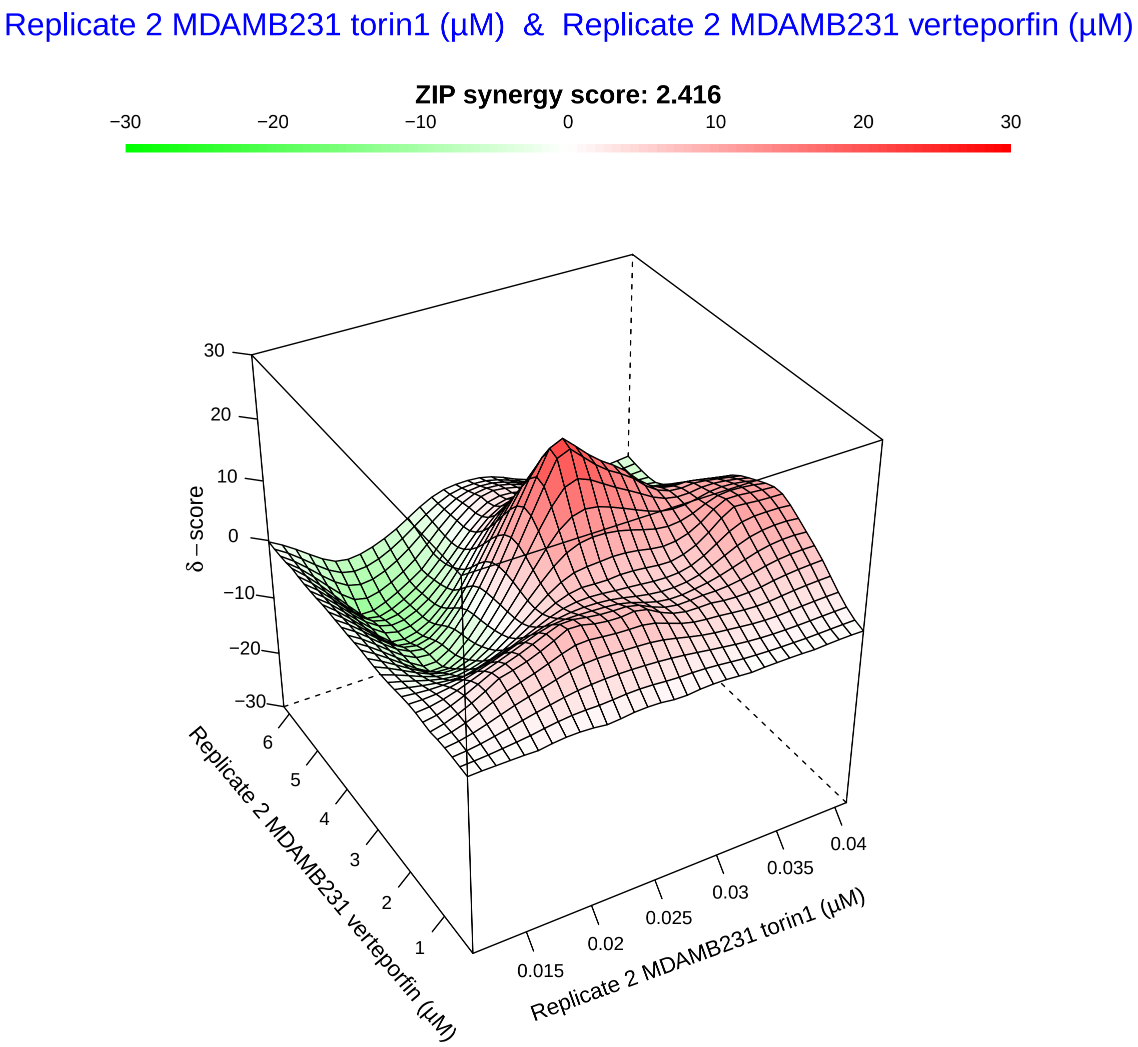
Replicate 2 MDAMB231 torin1 (µM) & Replicate 2 MDAMB231 verteporfin (µM)
ZIP synergy score: 2.416
−30
−20
−10
0
10
20
30
δ − score
Replicate 2 MDAMB231 verteporfin (µM)
1
2
3
4
5
6
Replicate 2 MDAMB231 torin1 (µM)
0.015
0.02
0.025
0.03
0.035
0.04

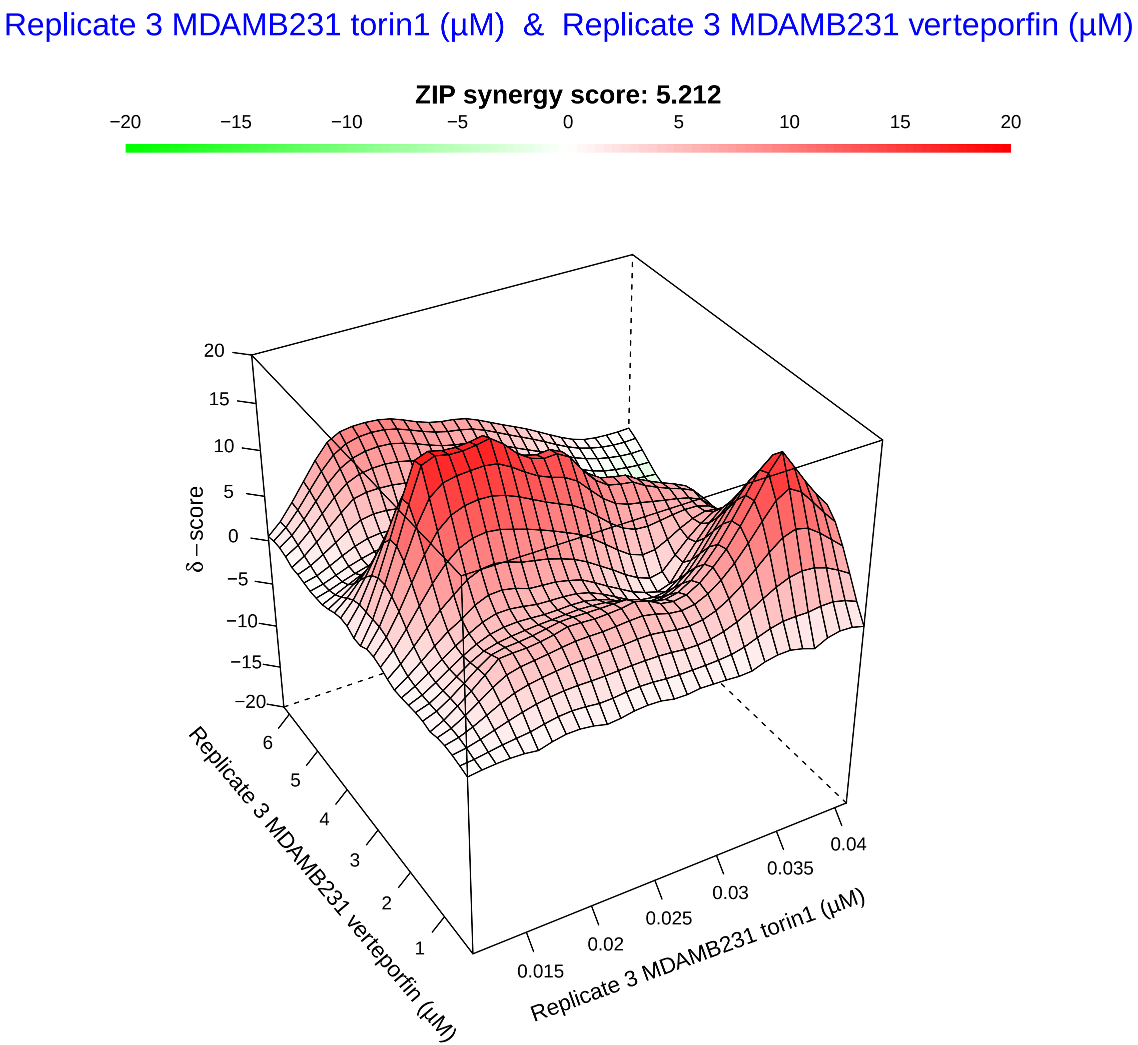

Replicate 3 MDAMB231 torin1 (µM) & Replicate 3 MDAMB231 verteporfin (µM)
ZIP synergy score: 5.212
−20
−15
−10
−5
0
5
10
15
20
δ − score
Replicate 3 MDAMB231 verteporfin (µM)
1
2
3
4
5
6
Replicate 3 MDAMB231 torin1 (µM)
0.015
0.02
0.025
0.03
0.035
0.04

average SUM1315 torin1 (µM) & average SUM1315 verteporfin (µM)

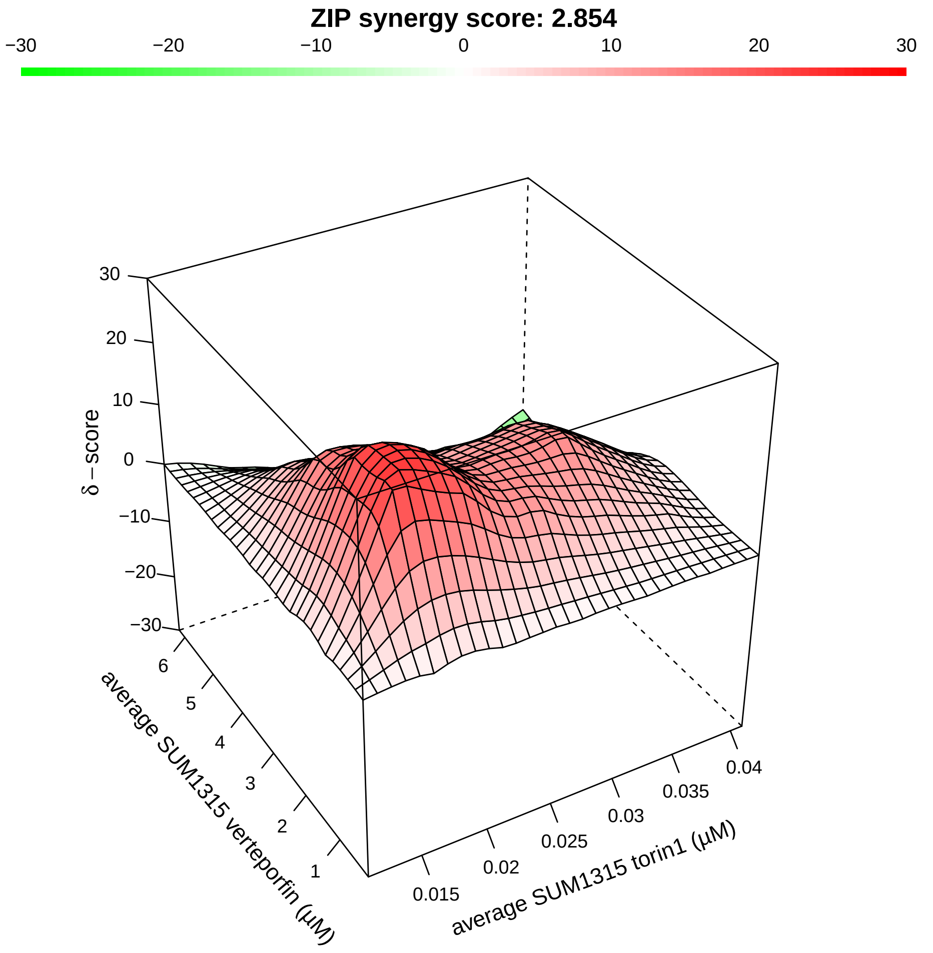

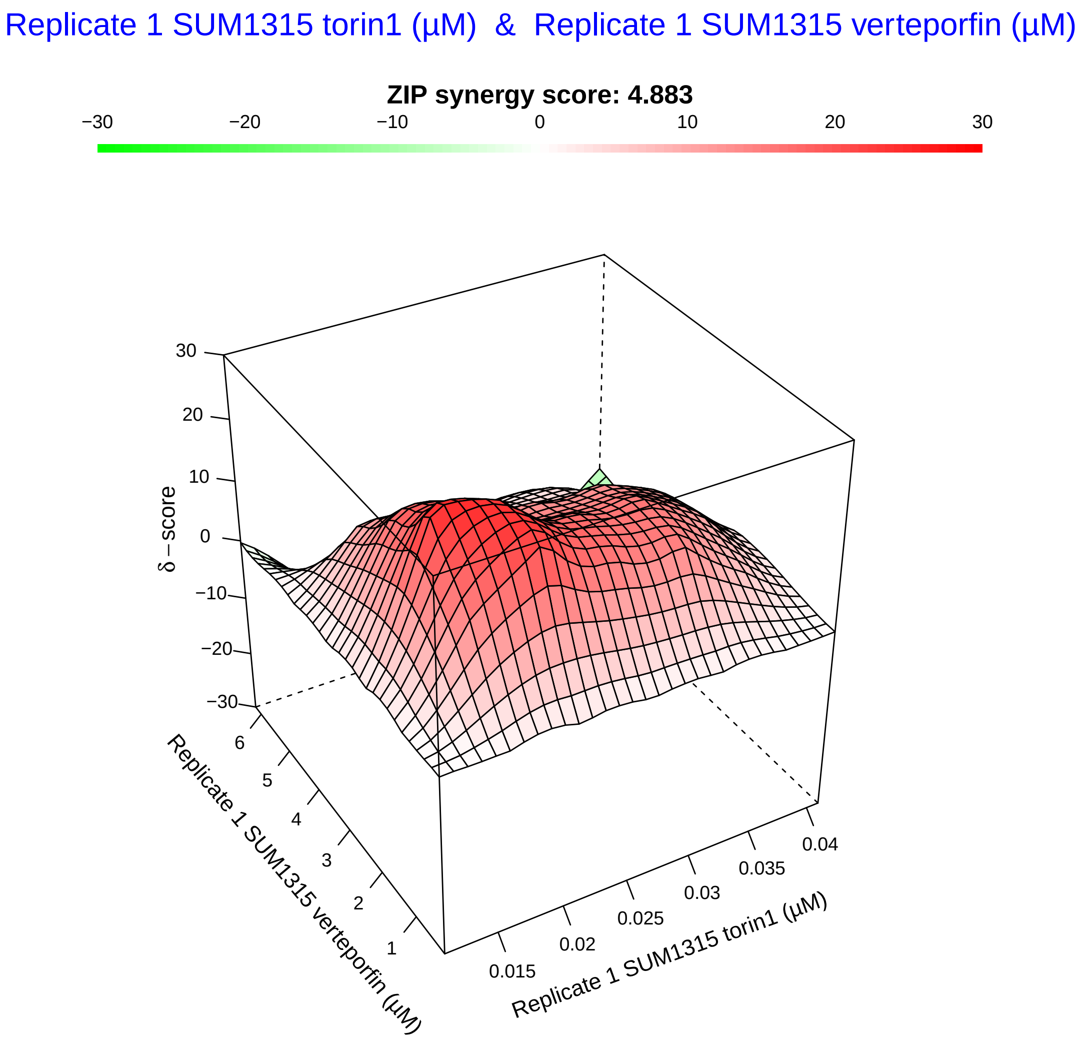
Replicate 1 SUM1315 torin1 (µM) & Replicate 1 SUM1315 verteporfin (µM)
ZIP synergy score: 4.883
−30
−20
−10
0
10
20
30
δ − score
30
20
10
0
−10
−20
−30
Replicate 1 SUM1315 verteporfin (µM)
6
5
4
3
2
1
Replicate 1 SUM1315 torin1 (µM)
0.015
0.02
0.025
0.03
0.035
0.04

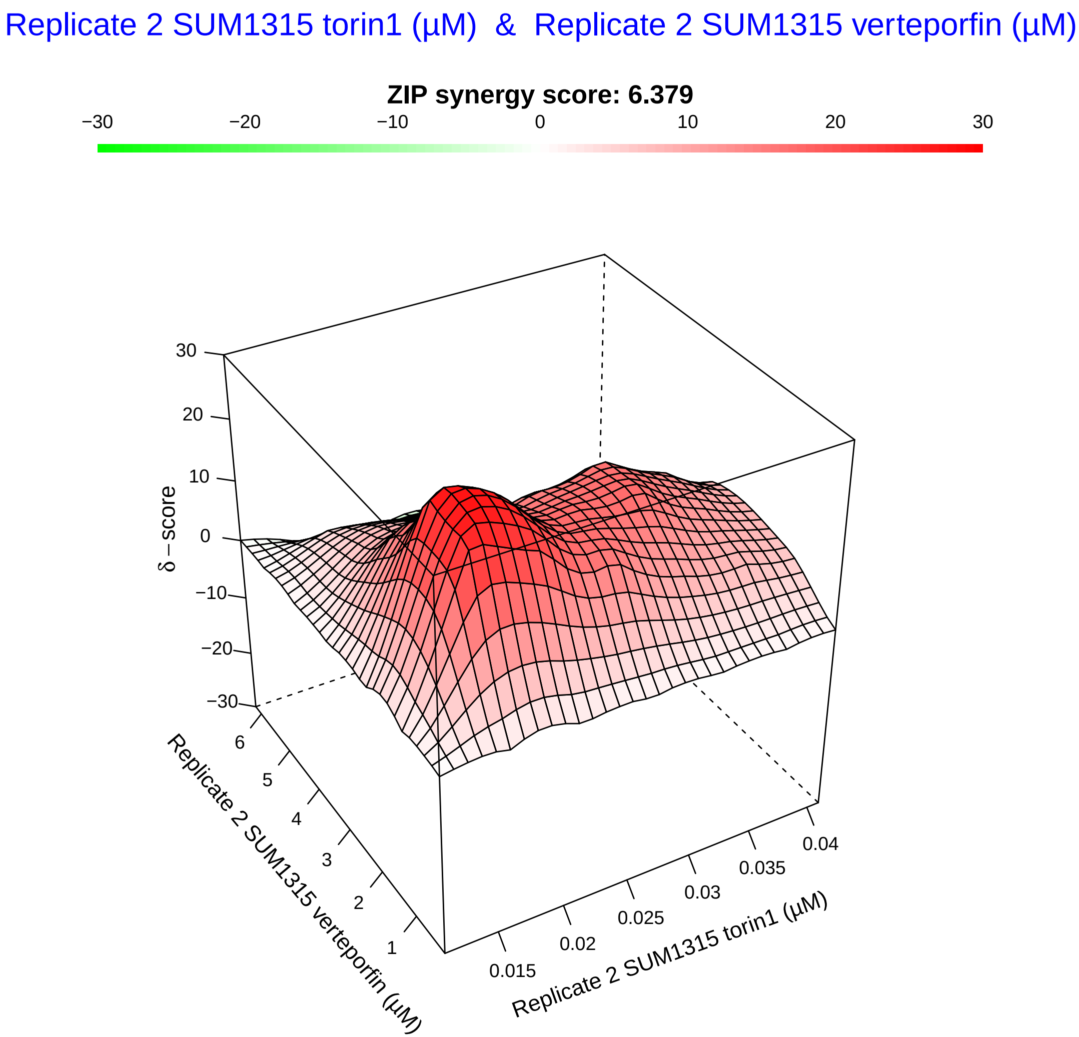

Replicate 2 SUM1315 torin1 (µM) & Replicate 2 SUM1315 verteporfin (µM)
ZIP synergy score: 6.379
−30
−20
−10
0
10
20
30
δ − score
30
20
10
0
−10
−20
−30
Replicate 2 SUM1315 verteporfin (µM)
6
5
4
3
2
1
Replicate 2 SUM1315 torin1 (µM)
0.015
0.02
0.025
0.03
0.035
0.04

Replicate 3 SUM1315 torin1 (µM) & Replicate 3 SUM1315 verteporfin (µM)

ZIP synergy score: −2.948

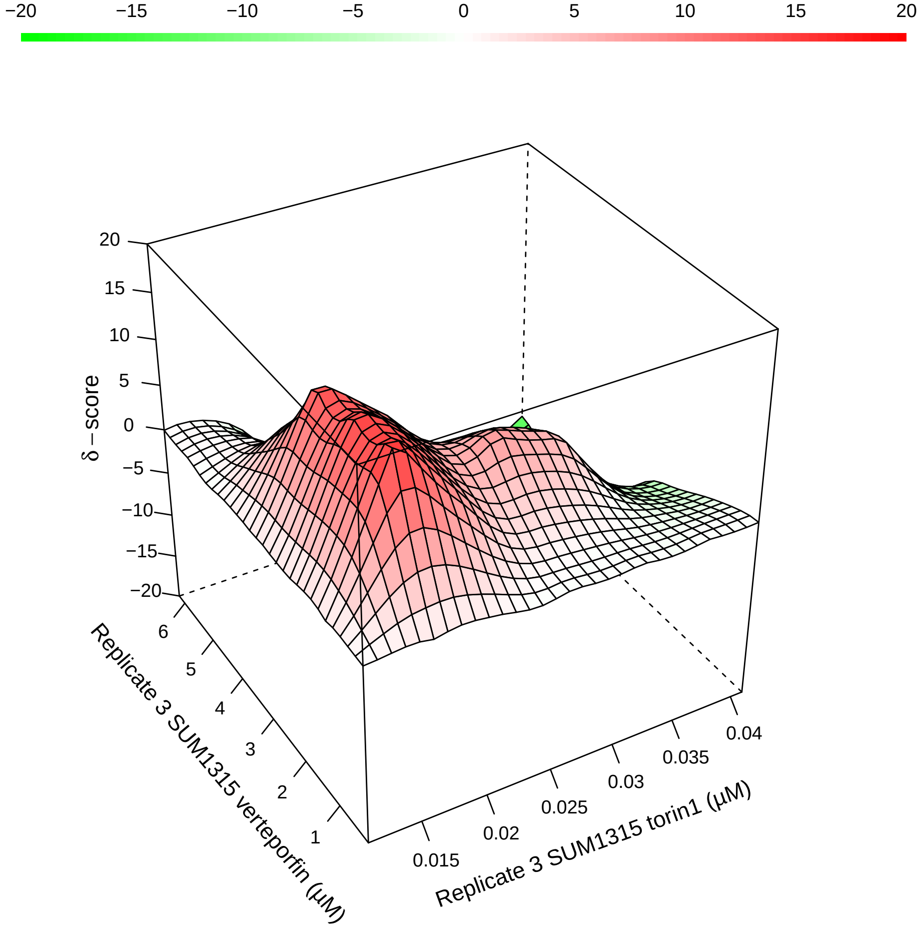

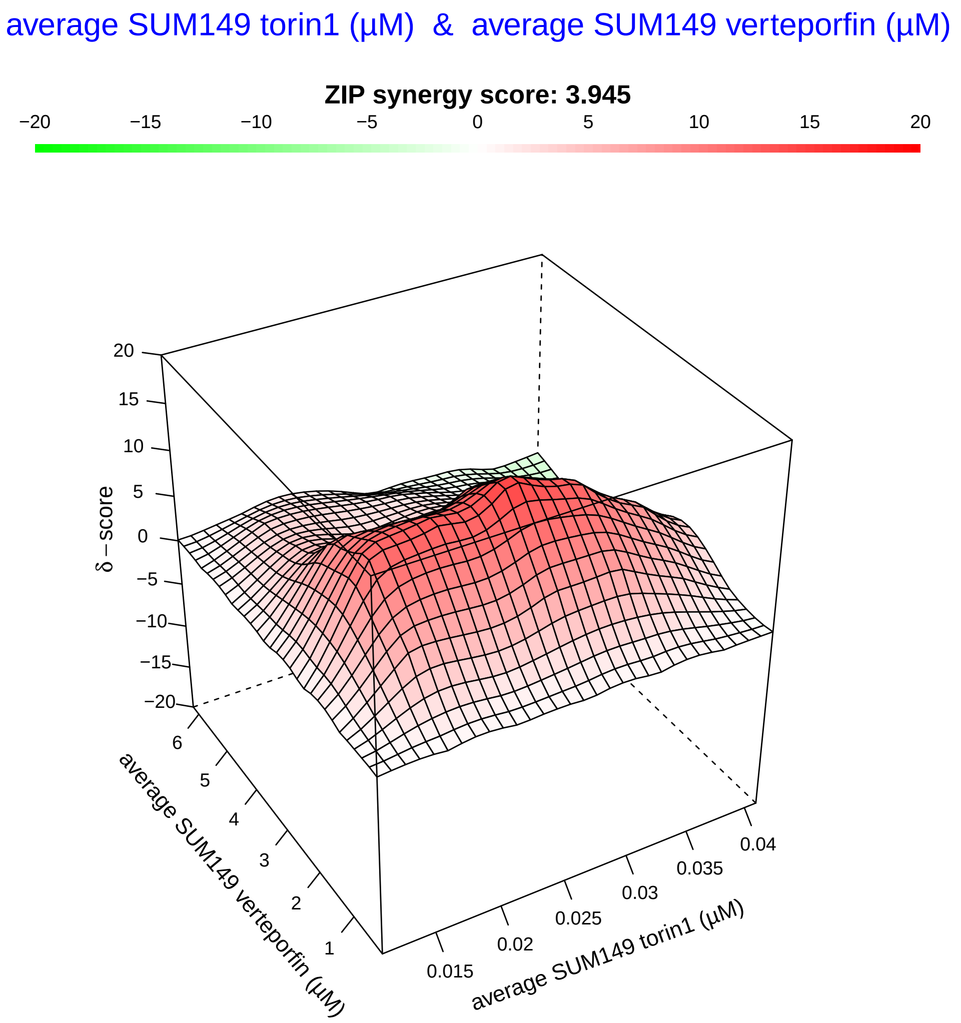
average SUM149 torin1 (μM) & average SUM149 verteporfin (μM)
ZIP synergy score: 3.945
−20
−15
−10
−5
0
5
10
15
20
δ − score
average SUM149 verteporfin (μM)
1
2
3
4
5
6
average SUM149 torin1 (μM)
0.015
0.02
0.025
0.03
0.035
0.04

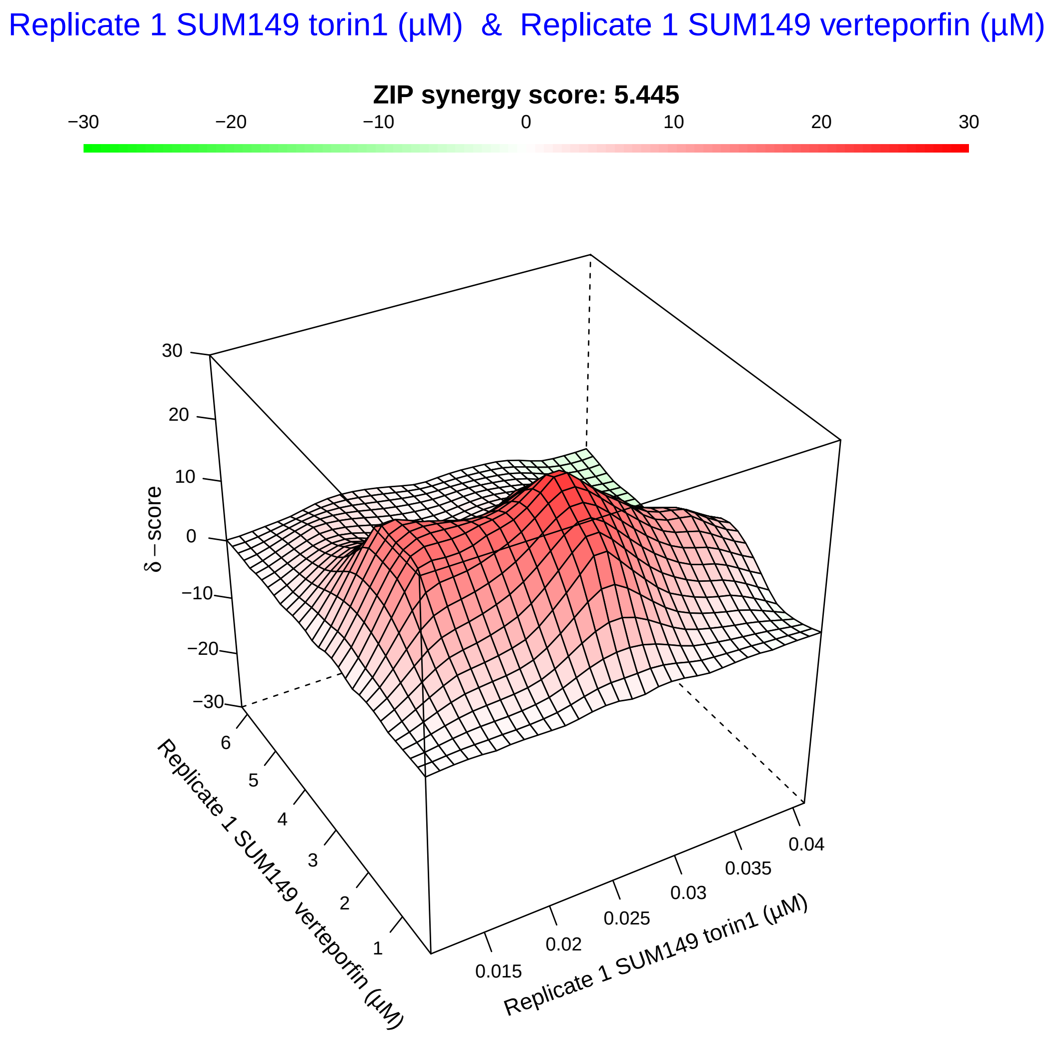

Replicate 1 SUM149 torin1 (µM) & Replicate 1 SUM149 verteporfin (µM)
ZIP synergy score: 5.445
−30
−20
−10
0
10
20
30
δ − score
30
20
10
0
−10
−20
−30
Replicate 1 SUM149 verteporfin (µM)
6
5
4
3
2
1
Replicate 1 SUM149 torin1 (µM)
0.015
0.02
0.025
0.03
0.035
0.04

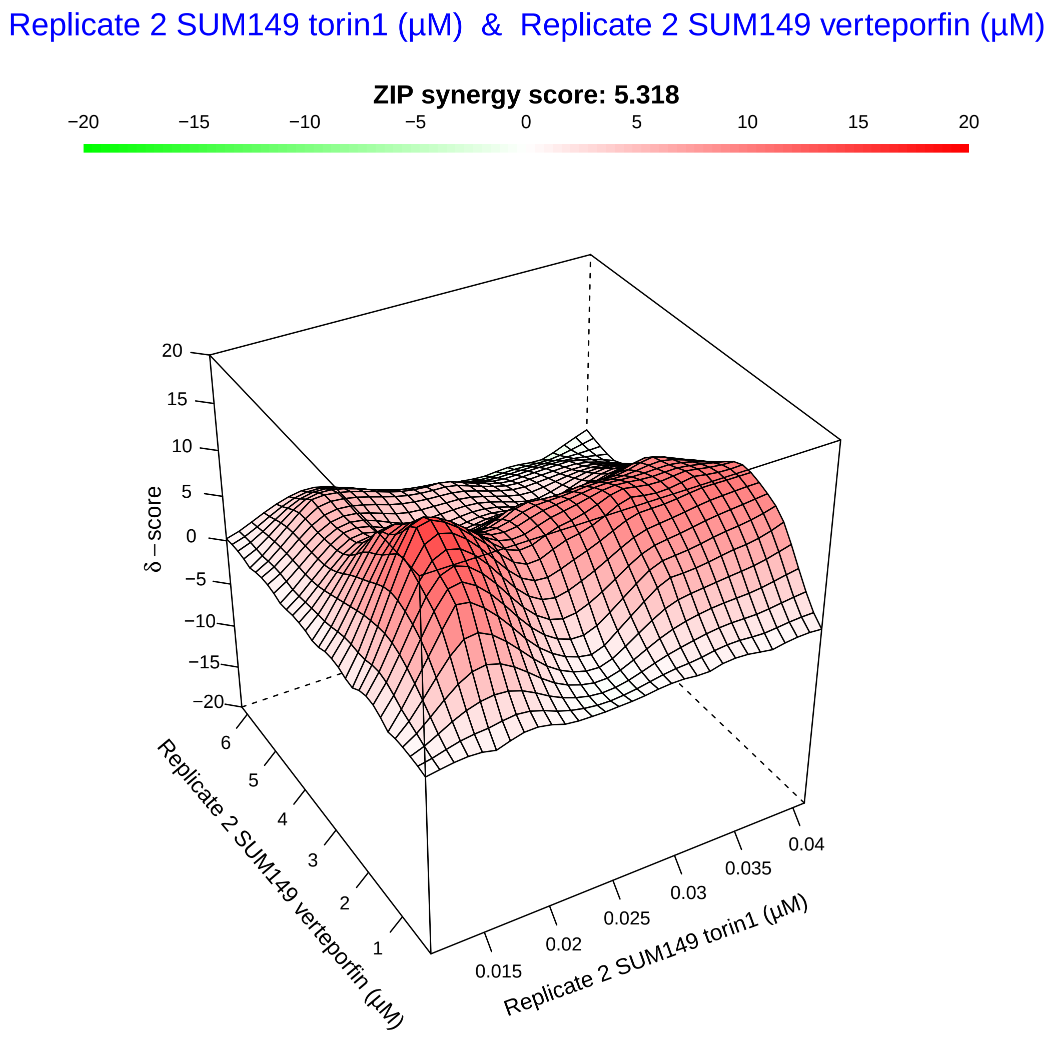

Replicate 2 SUM149 torin1 (µM) & Replicate 2 SUM149 verteporfin (µM)
ZIP synergy score: 5.318
−20
−15
−10
−5
0
5
10
15
20
δ − score
Replicate 2 SUM149 verteporfin (µM)
1
2
3
4
5
6
Replicate 2 SUM149 torin1 (µM)
0.015
0.02
0.025
0.03
0.035
0.04

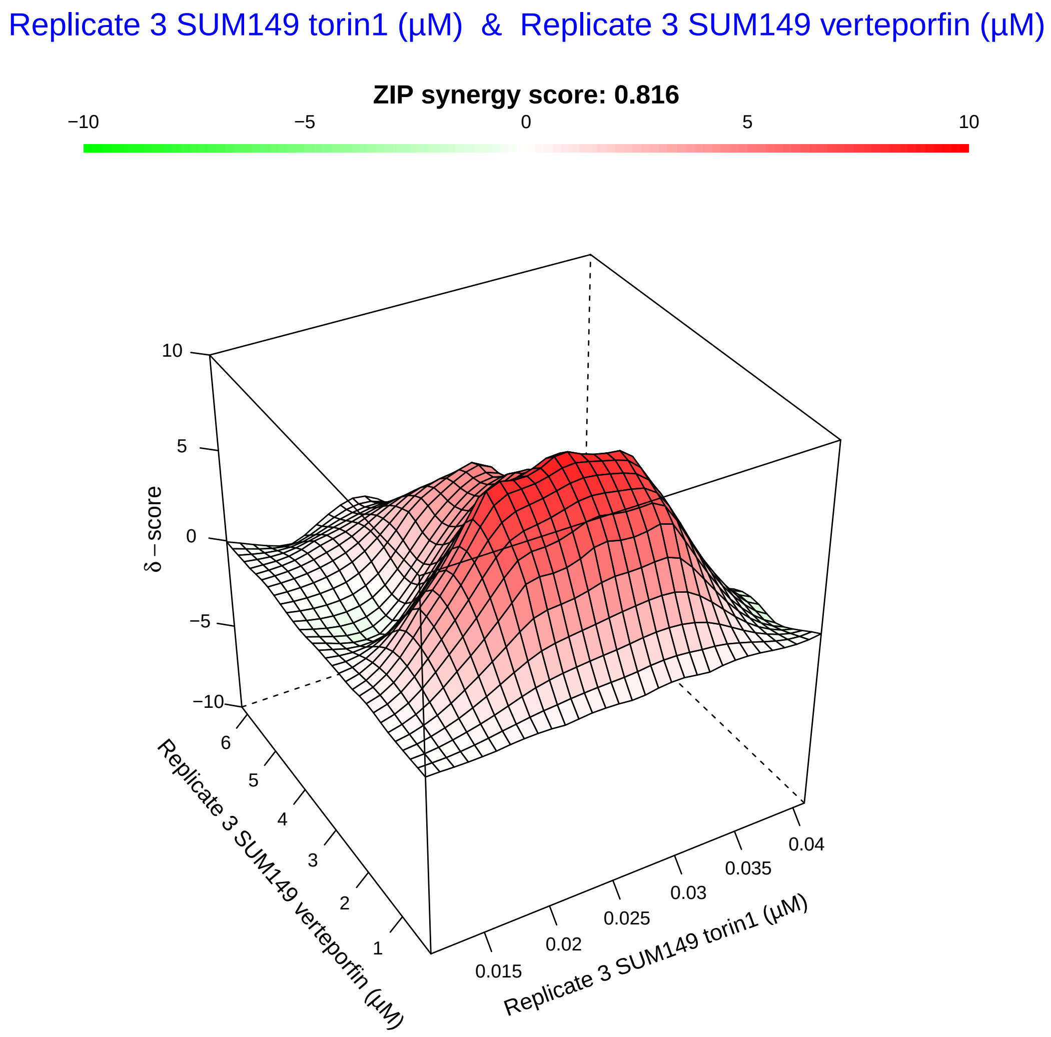
Replicate 3 SUM149 torin1 (µM) & Replicate 3 SUM149 verteporfin (µM)
ZIP synergy score: 0.816
−10
−5
0
5
10
δ − score
Replicate 3 SUM149 verteporfin (µM)
1
2
3
4
5
6
Replicate 3 SUM149 torin1 (µM)
0.015
0.02
0.025
0.03
0.035
0.04
